# Competitive ligand exchange of Fe^3+^ catecholate complexes with novel biobased chelators: a DFT-metadynamics investigation

**DOI:** 10.1039/d6ra06887a

**Published:** 2026-11-11

**Authors:** Mojgan Heshmat, Pavlo Kostetskyy, Daan S. van Es

**Affiliations:** a Wageningen Food & Biobased Research Bornse Weilanden 9 6708 WG Wageningen The Netherlands mojgan.heshmat@wur.nl; b Archer Daniels Midland Company 1001 N Brush College Road Decatur IL 62521 USA

## Abstract

The complexation of metal ions by polyphenolic compounds is a ubiquitous phenomenon in both natural and engineered systems, encompassing a vast multidimensional landscape of metal–ligand interactions. An important challenge in a range of applications, including textile care, is the efficient removal of stains formed by polyphenolic compounds coordinated to metal ions at the molecular level. In this study, we investigate the ligand-exchange process between a catecholate ligand coordinated to an Fe^3+^ center – a key molecular origin of persistent colored stains – and six candidate chelating ligands using *ab initio* molecular dynamics (AIMD) simulations. The selected ligands were chosen for their potential derivation from bio-based feedstocks, offering promising routes toward sustainable and environmentally benign stain-removal technologies. Our results reveal that effective metal-ion sequestration is governed not only by the thermodynamic stability of the resulting metal–ligand complexes but also by the kinetics of ligand exchange. Furthermore, we elucidate how the electronic structure of the incoming ligand influences the energetics of the exchange process and demonstrate the critical role of protonation state in determining both exchange accessibility and the relative stabilities of reactant and product complexes. Collectively, these findings provide molecular-level insights into Fe^3+^ chelation and ligand substitution mechanisms, advancing our understanding of structure–property relationships in metal binding. The knowledge generated in this work provides molecular-level insights into ligand exchange in a model Fe^3+^–catecholate system and may guide the development of novel bio-based chelating agents for stain-removal applications.

## Abstract 2

Breaking a stain begins with breaking a metal–ligand partnership. The challenge is finding bio-based chelators that can do so both rapidly and robustly.

## Graphical abstract

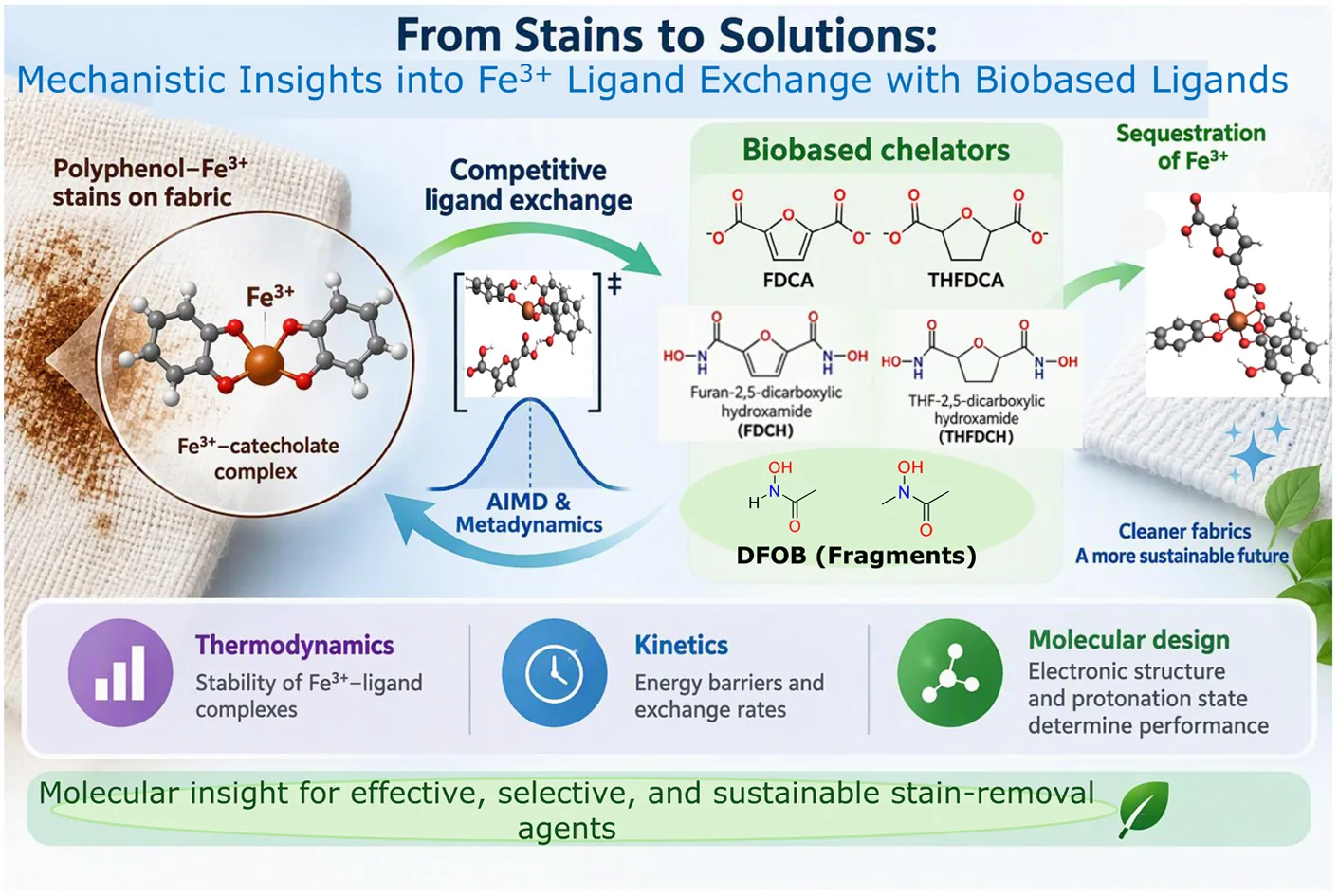

## Introduction

The complexation of metal ions by multidentate polyphenolic compounds, typically referred to as chelation, is a widely occurring phenomenon. This is observed in a number of naturally occurring and synthetic processes and can span an enormous multidimensional space of metal-catecholate combinations.^1–4^ These complexes typically involve covalent and non-covalent interactions between metal ions and the organic molecules, varying in the total number of coordinating groups bound to the metal center.^5,6^ Some of the important considerations when evaluating complexation include metal type and oxidation state, ligand identity, size, shape, and configuration, binding motif, solvation environment, solution pH, among others, all of which have an effect on the physical, chemical and optical properties once formed. Furthermore, for redox active metals autoxidation can occur with electron transfer leading to changes in metal oxidation state and ligand structure, which can lead to polymerisation.^7^ A key question in various applications is how to effectively remove staining from *e.g.* fabrics and fibres, caused by polyphenolic compounds bound to these substrates at the molecular level.

Experimental observations have shown that polyphenol stains are darkened, fixed, and made less water-soluble when bound to metal ions that can be found in aqueous solutions, including calcium, magnesium, iron, and copper. Chelators that can form stronger complexes with the metal ions can thus act as competitive ligands displacing the polyphenols, effectively removing types of stains from various surfaces.^8–10^ Various reaction conditions such as pH, temperature and time can facilitate the metal ion exchange between polyphenol ligands and external ligands. Catechol (1,2-benzenediol) is a compound that can be used as a proxy during stain removal testing for polyphenolic compounds, typically found in wine, fruit juices, coffee, and tea, which result in staining of fabrics.^5,6^ Catechol was selected as a tractable model for metal-binding polyphenolic motifs commonly encountered in stain-forming systems. While real stains may contain more complex mixtures of polyphenols, multiple coordination environments, and oxidation products, the present model enables controlled investigation of elementary Fe^3+^ ligand–exchange processes at the molecular level.

Carboxylic^11–13^ and hydroxamic^14–16^ acids, along with their conjugate bases are known to be good ligands for a range of metal ions of varying oxidation states. Hydroxamic acids are important because they occur in natural siderophores and other biologically active molecules. These ligands have found applications in a variety of industries including foods and beverages, agricultural, pharmaceutical, chemical, petroleum, mining, textile and other application. These species can be synthesized from a variety of renewable and non-renewable feedstocks through traditional heterogeneous and homogeneous catalytic means, as well as fermentation.^17–21^ Carboxylic acids span a wide range of species, varying in size, number of carboxylic acid functionalities, molecular backbone identity, and degree of branching.

There have been a number of experimental^22–24^ and computational^25–33^ studies published, with the aim of quantifying the thermodynamics of chelation of metal complexes and corresponding species distributions at equilibrium. Typically, electronic structure calculations are used as the computational tool for quantification of the kinetics and thermodynamics of chemical transformations.^34–41^ Contrary to existing thermodynamic studies, in-depth investigations of the kinetics of chelation are still relatively scarce.^42–44^ To this end, we investigate the kinetics of ligand exchange and examine how the electronic structures of the ligands influence the exchange process.

We are interested in developing new biobased and biodegradable chelators that can outperform the current, often fossil based products under mild conditions. Nature provides a broad range of metal specific chelator systems with very high complexation constants such as siderophore and hemoglobin.^42–44^ These are however often impractical under realistic application conditions, due to high costs per weight, difficulty of synthesis and (too) slow kinetics.^43,45^ Hence there is a growing interest in new effective bioinspired renewable chelators. Furan-2,5-dicarboxylic acid, or FDCA, is an emerging biobased building block that can be obtained from carbohydrate feedstocks.^46^ We note that through FDCA hydrogenation a 90 : 10 of the cis : trans mixture of THFDCA is obtained. Cis-Tetrahydrofuran-2,5-dicarboxylic acid disodium salt is a specialized, bio-based chemical precursor primarily utilized in green chemistry and materials science which is proved to be an efficient chelator for calcium and magnesium cations sequestration.^47^

In this work we model the exchange process between catecholate ligands bound to an Fe^3+^ metal center and six candidate ligands using *ab-initio* molecular dynamics (AIMD) simulations. The ligands, shown in Fig. 1, are THF-2,5-dicarboxylic(ate – disodium) (THFDCA), furan-2,5-dicarboxylic(ate – disodium) (FDCA), furan-2,5-dihydroxamic(ate – disodium) (FDCH), THF-2,5-dihydroxamic(ate – disodium) (THFDCH) and hydroxamate-desferrioxamine B (DFOB). DFOB is known as a strong Fe^3+^ scavenger.^5,6^ However, the rate at which DFOB can displace weaker ferric ion binding ligands is relatively slow^42–44^ which may be attributed to the flexibility of the structure. Increasing linker length increases conformational freedom of the free ligand, which raises its conformational entropy.^44^ To this end, we only consider a small part of the long chain (hydroxamic functionality) as depicted in Fig. 1. Some of the selected ligands have been tested experimentally for their stain removal performance, providing benchmark data and allowing for support of computational findings. The major findings in this study include: (i) not only the thermodynamic stability of substituting ligand-complex is important for an efficient binding but also the kinetics of exchange between various ligands and metal cations in the complex should be taken into account, (ii) the influence of ligand electronic structure on the energetics of exchange, and (iii) the impact of protonated and deprotonated states on the ease of exchange and relative ground state energies of the reactants. This study provides new insights into the influence of molecular identity and structure in chelation of Fe^3+^ metal ions and will assist in designing novel, effective and selective bio-based chelators. In order to optimise research efforts, computational chemistry can be used to screen a wide array of potentially effective chemical structures for their chelation efficacy and support the selection process towards development of candidate molecules.

**Fig. 1:**
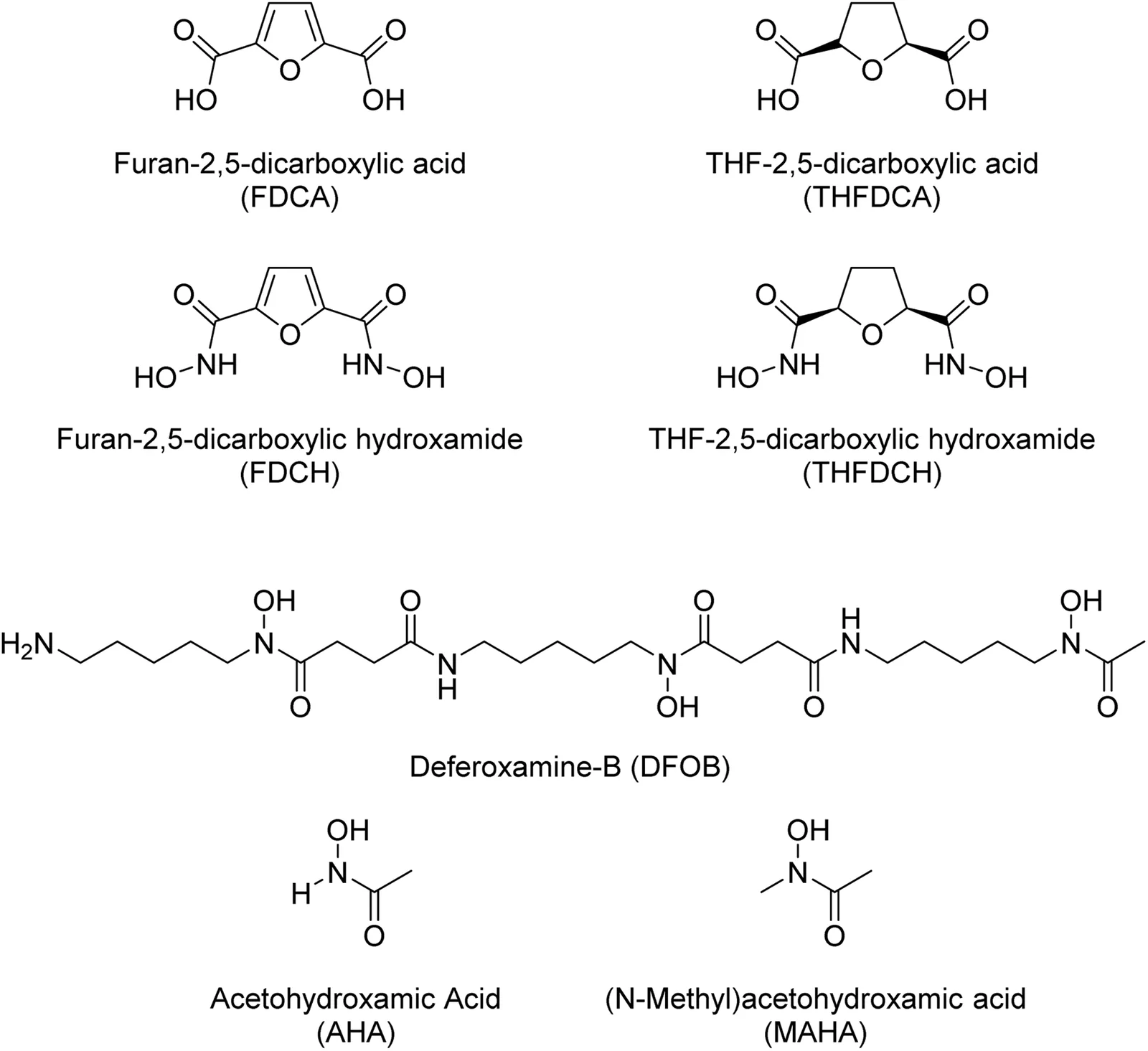
The selected biobased ligands for replacement of the catecholate from complexation with Fe^3+^. The impact of electronic structure of various ligands is investigated. For the THF variants only the cis isomer is considered (in a deprotonated form all H^+^ atoms are replaced with Na^+^).

## Computational methods

Several types of electronic structure calculations were performed in the current work. *Ab-initio* molecular dynamics (AIMD) simulations were performed using the CP2K software package,^48^ with the Gaussian and plane-wave (GPW) method.^49^ The valence orbitals were expanded in the DZVP-MOLOPT Gaussian basis set^50^ in combination with Goedecker, Teter and Hutter pseudopotentials were used with a plane wave cutoff energy of 280 Ry. We used the PBE density functional, also augmented with Grimme D3 dispersion correction.^51,52^ The criteria for self-consistent field (SCF) convergence was set to 5.0 × 10^−7^ and for the calculated free energy values to 1.0 × 10^−9^ kcal mol^−1^. The AIMD simulations were performed in a NVT ensemble, with the temperature controlled by a canonical sampling through velocity rescaling (CSVR) thermostat, set at a temperature of 298 K, and a period of 500 fs. The MD time step was set to 0.5 fs. To investigate the reaction mechanism at finite temperature, elucidate the reaction pathway and identify the transition state region between reactant and product states, we performed metadynamics (MTD) simulations using the PLUMED plug-in in combination with CP2K.^53^ The simulations were conducted without periodic boundary conditions, and electrostatic interactions were treated using the aperiodic Poisson solver implemented in CP2K. A sufficiently large simulation cell of 25 Å was employed to eliminate finite-size effects. The simulations were initiated from the optimized molecular structures and conducted until several transition state re-crossing events could be observed. To prevent sampling from unnecessary regions of the Free Energy Surface (FES) harmonic walls with a force constant *K* = 250 kcal mol^−1^ Å^−2^ were used for the collective variables (CVs), with the Gaussian bias potentials added every 25 steps. Multiple test simulations were run to set the computational parameters, including the height and width of the Gaussian bias potentials and the quadratic walls. The Gaussian width is set to 0.4 and Gaussian height to 0.35 with a pace of 25 (a plumed input as well as FES convergence plots are represented in the SI Fig. S1). The CVs were defined as catecholate O⋯Fe^3+^ interatomic distance in the reactant complex and external ligand O⋯Fe^3+^ bond distance for the product complex, as shown in Fig. 2. One of the Fe^3+^⋯O bonds in the reactant complex is cleaved and one Fe^3+^⋯O bond in the product complex is formed alongside each MD trajectory. To aid visualization of the MD trajectories and individual snapshots, we used the VMD software package.^54,55^

**Fig. 2:**
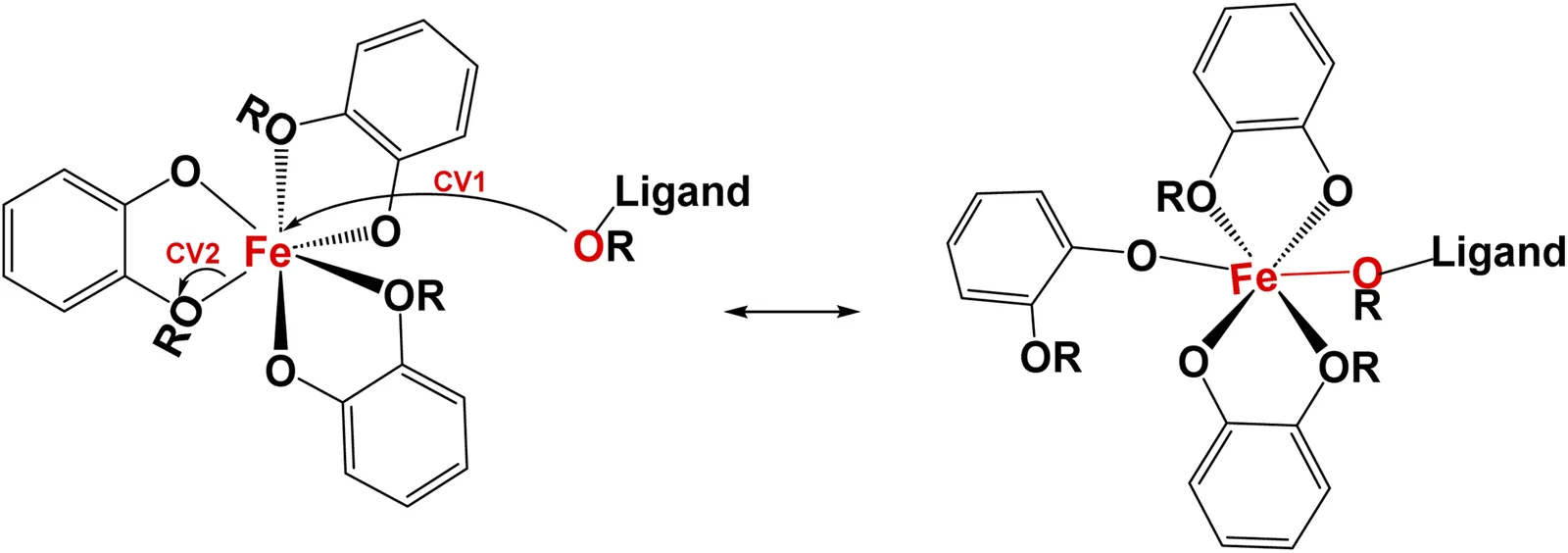
The schematic representation of the mechanism that shows Fe^3+^⋯O(catecholate) bond cleavage by a nucleophilic attack of an external ligand and Fe^3+^⋯O(Ligand) bond formation (R = H^+^ or Na^+^). The CVs are selected collective variables for this transformation.

The reported free-energy surfaces correspond to a projection onto the selected Fe–O distance coordinates. While additional slow variables may influence the quantitative shape of the free-energy landscape, particularly other unbiased Fe–O(catecholate) distances may vary alongside the MTD trajectories (the corresponding distances against the MD steps are included in the SI Fig. S2). Nevertheless, we acknowledge that hidden-variable effects cannot be completely excluded. In particular, changes in Fe^3+^ coordination numbers, solvent reorganization and ligand rotational motions may contribute to the free-energy barrier. Consequently, the reported barriers should be interpreted as free-energy estimates within the chosen collective-variable space. We included fully protonated and fully deprotonated states of the molecular complex systems. For charge neutrality sodium cations in deprotonated cases were used. Because protonation and deprotonation alter the net charge of the system, explicit Na^+^ counterions were introduced to maintain charge neutrality where appropriate. The role of the counterions is primarily electrostatic, and no direct coordination of Na^+^ to the Fe center was observed during the simulations. The fully protonated and fully deprotonated states were selected as chemically meaningful reference systems that allow isolation of protonation effects on the ligand-exchange process and quantify the effect of protonation on the reaction mechanism and free-energy landscape. The actual protonation distribution depends on the pH of the solution and the p*K*_a_ values of the relevant acidic groups. Consequently, partially protonated species are expected to coexist with the fully protonated and fully deprotonated forms over a significant pH range. The reported experimentally measured p*K*a values of catechol, carboxylic acids, and hydroxamic acids are catechol = 9.25–9.45, THFDCA = 3.04, FDCA = 2.76, hydroxamic acids = 8.5–9.5).

To obtain more insights about the interaction between catecholate-Fe^3+^ at the reactant state and also external ligand with the catecholate-Fe^3+^ at the transition state, energy decomposition analysis (EDA) and frontier molecular orbital (FMO) analysis were performed using the ADF software package and static DFT calculations.^56,57^ For each reaction the lowest energy structures of ground and transition states were obtained. The calculations were carried out with the PBE functional, augmented with Grimme's D3 dispersion correction^50–52^ and the TZP basis set.^56^ The optimized geometries have been verified to have zero imaginary frequency modes and the transition state one imaginary frequency. The transition states were searched on the basis of potential energy scans using linear transit (LT) calculations by employing the same reaction coordinates (distances) used in MTD calculations, starting from and ending at fully converged stable minima in a series of steps. In each LT calculation, all degrees of freedom are fully optimized except the reaction coordinate, which is scanned stepwise. The spin multiplicity of all molecular complexes was set at six which is correlated to a high spin d^5^ Fe^3+^ cation supported by previously reported studies.^58–62^

Independent simulations with 12 explicit water molecules in each system at BLYP-D3 DZVP-MOLOPT were performed to investigate the reproducibility of the ligand ranking. In the protonated systems the protons interact with water oxygens and several proton-transfers from catecholate and ligands to water molecules coupled with the main CVs can be detected (Fig. S4 in SI). Note that the aqueous environment plays a critical role in determining hydrogen-bonding patterns, proton-transfer events, and the resulting reaction energetics. The explicit water molecules were included to capture local hydrogen-bonding interactions, solvent-assisted structural rearrangements, and possible proton-transfer events occurring in the immediate vicinity of the reacting complex. Despite quantitative changes in the free-energy profiles upon inclusion of explicit water molecules, the principal relative ligand trends were retained, supporting the qualitative robustness of the ligand ranking. Nevertheless, we acknowledge that alternative counterion distributions may influence local electrostatic environments and consequently affect quantitative barrier heights, which is again for all the investigated systems. The present model provides only a partial representation of the aqueous environment.

The calculated differences between the protonated and deprotonated systems reflect the combined effects of protonation state, charge redistribution, and Na^+^ counterion stabilization within the specific model systems investigated. In Table 1 the specification of every simulated system is listed.

**Table 1:** Specification of the simulated chemical systems

Ligand	Protonation state	Total ligand charge	Number of Na^+^ on ligand	Catecholate charge/compensated by protonation or Na^+^	Total system charge	System label
THFDCA	Fully protonated	0 (2H^+^)	0	−3/3H^+^(monoanion)	0	5H^+^
THFDCA	Fully deprotonated	−2	2	−3/3Na^+^(dianion)	0	0H^+^
FDCA	Fully protonated	0 (2H^+^)	0	−3/3H^+^(monoanion)	0	5H^+^
FDCA	Fully deprotonated	−2	2	−3/3Na^+^(dianion)	0	0H^+^
THFDCH	Fully protonated	0 (2H^+^)	0	−3/3H^+^(monoanion)	0	5H^+^
THFDCH	Fully deprotonated	−2	2	−3/3Na^+^(dianion)	0	0H^+^
FDCH	Fully protonated	0 (2H^+^)	0	−3/3H^+^(monoanion)	0	5H^+^
FDCH	Fully deprotonated	−2	2	−3/3Na^+^(dianion)	0	0H^+^
AHA	Fully protonated	0 (1H^+^)	0	−3/3H^+^(monoanion)	0	4H^+^
AHA	Fully deprotonated	−1	1	−3/3Na^+^(dianion)	0	0H+
MAHA	Fully protonated	0 (1H^+^)	0	−3/3H^+^(monoanion)	0	4H^+^
MAHA	Fully deprotonated	−1	1	−3/3Na^+^(dianion)	0	0H^+^

## Results and discussion

Our motivation for this work is understanding various aspects of the polyphenol demetallation (*i.e.* ligand exchange) reaction correlated to the electronic structure of the ligands. We start with the reactant state that is the molecular complex between Fe^3+^ and catecholate monoanion in protonated and dianion in fully deprotonated states. The product state is the molecular complex between the external ligand and Fe^3+^. To avoid conflating different energetic descriptors, we distinguish throughout the following discussion between kinetic, thermodynamic, and electronic-structure quantities. Kinetic accessibility is evaluated from the finite-temperature activation free energy (Δ*G*^≠^) obtained from the metadynamics free-energy surface. The thermodynamic driving force is evaluated from the reaction free-energy difference (Δ*G*) between the relevant reactant and product basins. These quantities describe distinct aspects of the ligand-exchange process and are therefore considered independently. The EDA calculations provide a third, complementary level of analysis: the individual interaction-energy components characterize electronic interactions within selected static configurations and are used to rationalize structural/electronic differences between the systems analysed. They are not interpreted as contributions to either Δ*G*^≠^ or Δ*G*.

As shown in Fig. 1, the compounds chosen as selective ligands of the Fe^3+^ ions *via* substitution of a catecholate moiety vary in size, functional group identity, and degree of protonation. The nucleophilic (electron donating) part of the external ligand molecule is either a carboxylic(protonated)/carboxylate(deprotonated) group, *i.e.*, THFDCA and FDCA or a hydroxamic(protonated)/hydroxamate(deprotonated) functionality, *i.e.*, THF-2,5-dihydroxamic(ate) (THFDCH), furan-2,5-dihydroxamic(ate) (FDCH), acetohydroxamic(ate) (AHA), *N*-methyl-acetohydroxamic(ate) (MAHA) (an active site of the DFOB) as shown in Fig. 1. This series has been selected for a comparison between different characteristics of the donating O-based ligands in the procedure of catecholate substitution. This means if COOH(Na) is a more appropriate candidate or –C=ON(R)OH(Na) group performs a better catecholate substitution. Furthermore, the presence of a flat and rigid hetero aromatic furan ring *versus* a more flexible THF ring can have an impact due to the different characteristics of the electronic structures of these rings. The presence of a methyl substituent in the hydroxamate-type ligand is also investigated in case of one single unit of DFOB, in order to probe for steric, inductive and conjugative effects. Hydroxamate anions in the form of long chain DFOB (Fig. 1) are known as a common iron cation sequestering agent.^5,6^ However, for extracting Fe^3+^ from the catecholate ligands experiments confirmed that due to the high flexibility and conformational freedom of the long chain (entropic stabilization), DFOB has a poor performance under conditions relevant for stain removal.^44,45^

We have selected both types of THF and furan rings as well as the linear hydroxamate to investigate the impact of the electronic and structural properties on the complexation with Fe^3+^ and compare it with the carboxylate groups. Disodium THF-2,5-dicarboxylate (THFDCA) anions have shown favorable performance in the stain removal problem in industrial applications.^42,43^ On the other hand, hydroxamic(ate) THFDCH and/or FDCH showed reversibility in exchange chelation.^44^ The reactant complex in all cases is a catecholate-Fe^3+^ molecular complex.

### Protonation effects

To investigate the influence of protonation state, we consider protonated catecholate (monoanion) and external ligands as well as fully deprotonated molecular system with Na^+^ substituents to maintain charge neutrality of the system (Fig. 2). We note that in a basic solution free Fe^3+^ precipitates as iron hydroxide, however, in this study the Fe^3+^ is not a free cation as the reagent and is always complexed to the catecholate anions. In Fig. 3–8 we have depicted the schematic representation of the nucleophilic substitution path (upper part) applied for bond cleavage and formation, variation of the CVs *versus* the MD-steps (middle part), and the calculated FES (lower part) for each transformation in substitution of the catecholate with each ligand showed in Fig. 1.

**Fig. 3:**
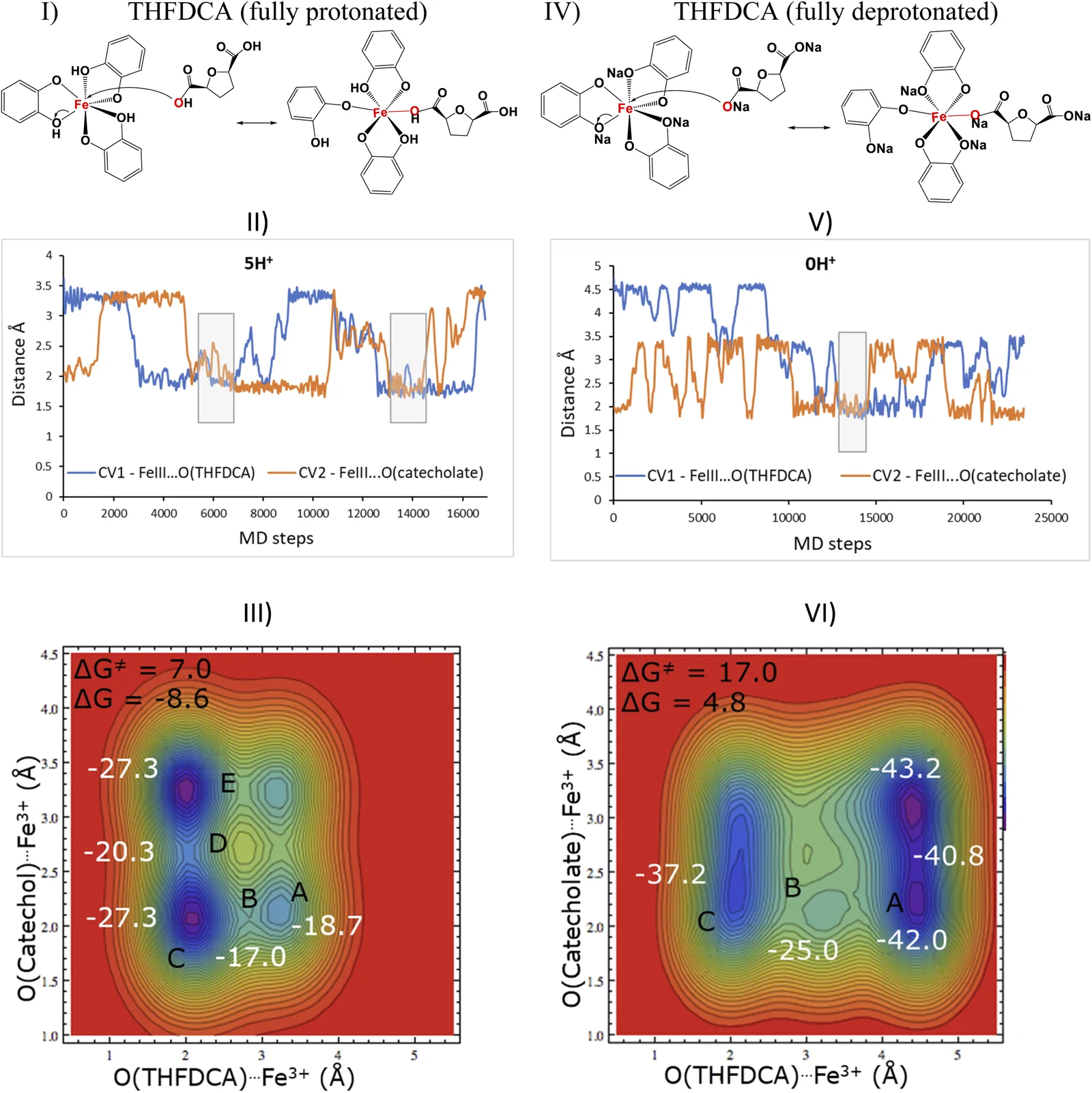
Top, Schematic representation of the Fe^3+^⋯O(catecholate) bond cleavage and formation of Fe^3+^⋯O(THFDCA) bond (I and IV), middle, the variation of the CVs (corresponding distances in Å) against the MD steps (II and V), down, the simulated free energy surface at 298 K and 1 atm for the bond cleavage/formation depicted in the upper part fully protonated and fully deprotonated systems (III and VI).

**Fig. 4:**
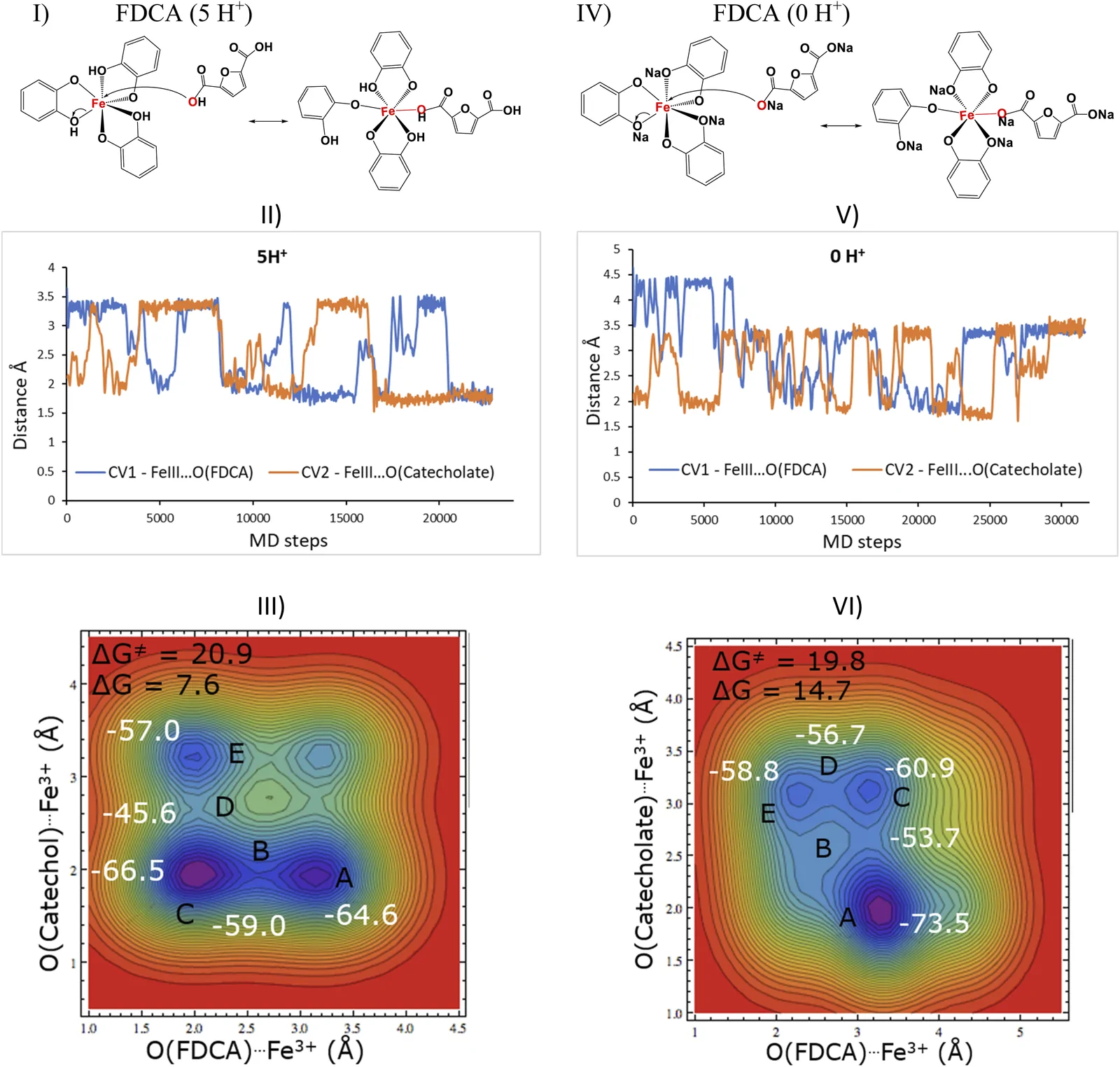
Top, schematic representation of the Fe^3+^⋯O(catecholate) bond cleavage and formation of Fe^3+^⋯O(FDCA) bond (I and IV), middle, the variation of the CVs (corresponding distances in Å) against the MD steps (II and V), down, the simulated free energy surface at 298 K and 1 atm for the bond cleavage/formation depicted in the upper part for fully protonated (III) and fully deprotonated (VI) systems.

**Fig. 5:**
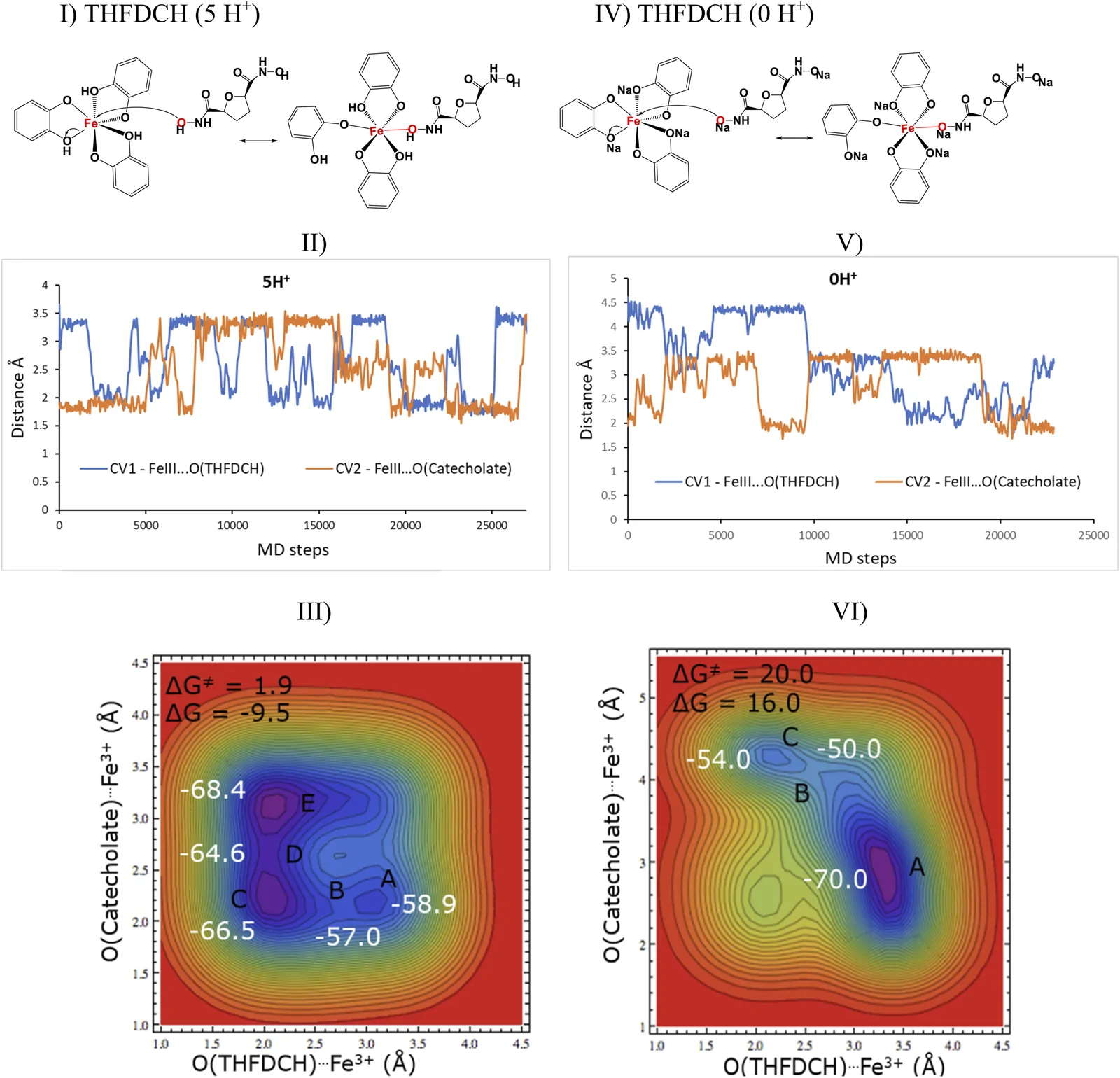
Top, schematic representation of the Fe^3+^⋯O(catecholate) bond cleavage and formation of Fe^3+^⋯O(THF-2,5-dihydroxamic(ate)) bond (I and IV), middle, the variation of the CVs (corresponding distances in Å) against the MD steps (II and V), down, the simulated free energy surface at 298 K and 1 atm for the bond cleavage/formation depicted in the upper part for fully protonated (left) and fully deprotonated (right) systems (III and VI).

**Fig. 6:**
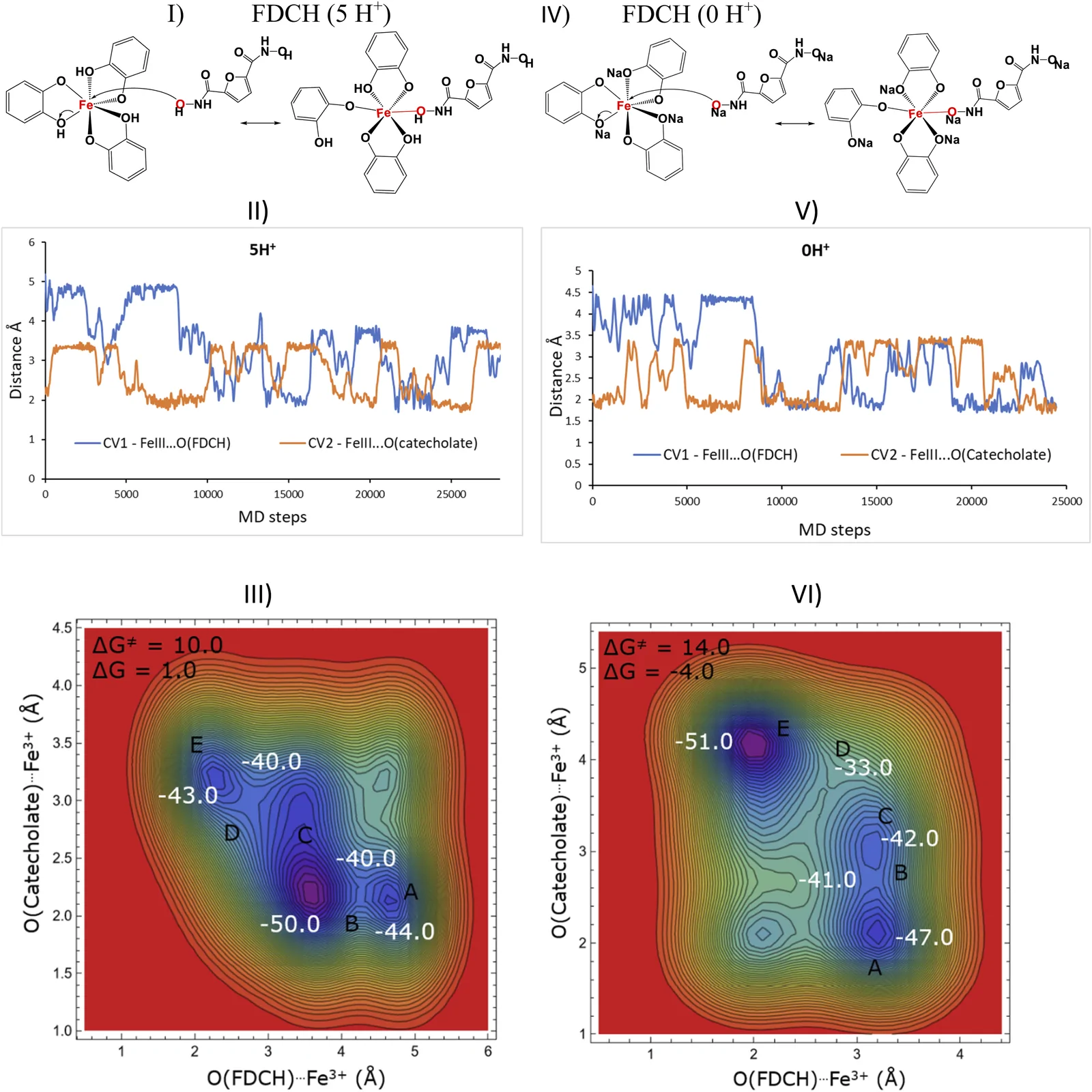
Top, schematic representation of the Fe^3+^⋯O(catecholate) bond cleavage and formation of Fe^3+^⋯O(furan-2,5-dihydroxamic(ate)) bond (I and IV), middle, the variation of the CVs (corresponding distances in Å) against the MD steps (II and V), down, the simulated free energy surface at 298 K and 1 atm for the bond cleavage/formation depicted in the upper part for fully protonated (III) and fully deprotonated (VI) systems.

**Fig. 7:**
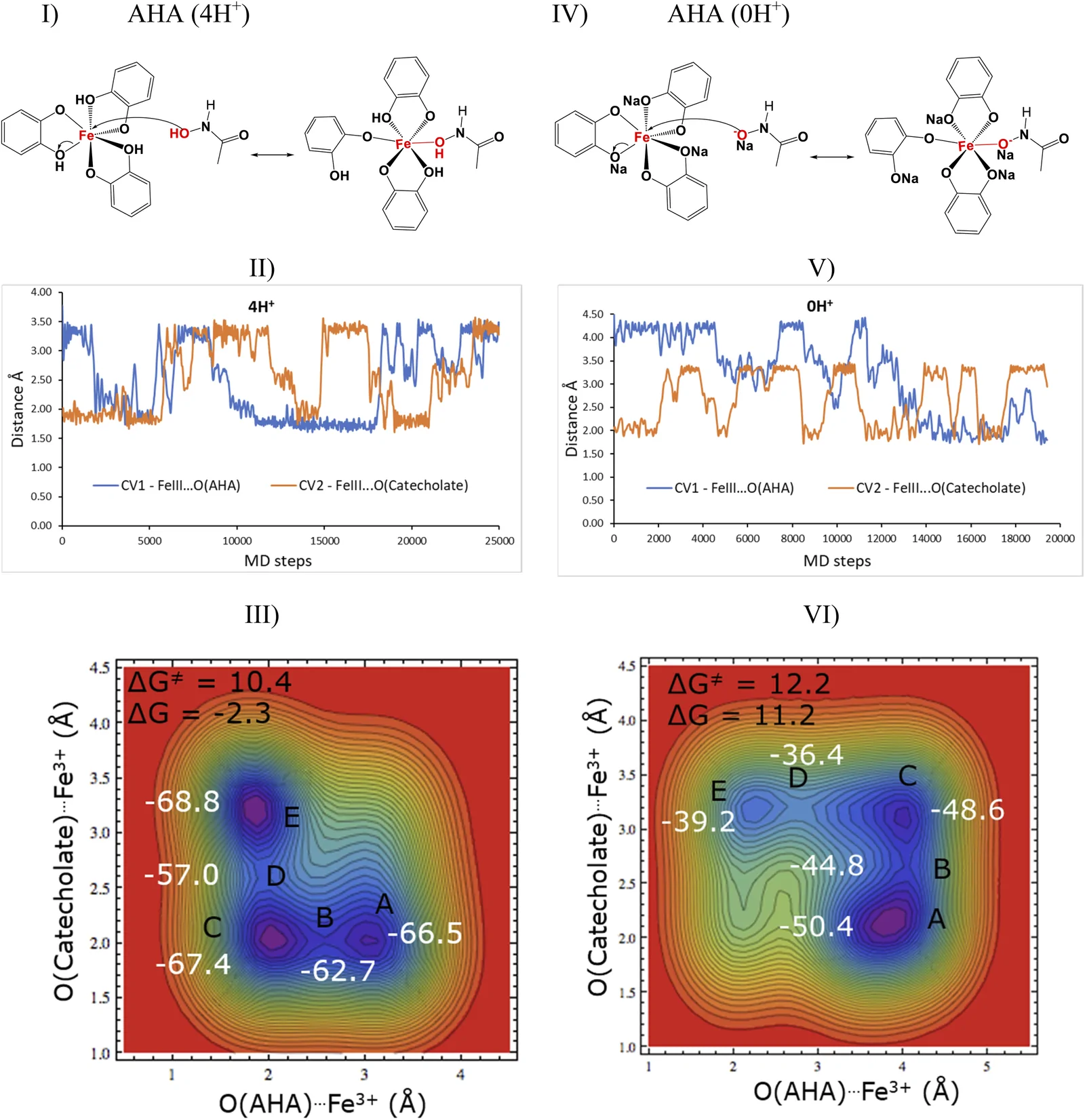
Up, schematic representation of the Fe^3+^⋯O(catecholate) bond cleavage and formation of Fe^3+^⋯O(AHA) bond (I and IV), middle, the variation of the CVs (corresponding distances in Å) against the MD steps (II and V), down, the simulated free energy surface at 298 K and 1 atm for the bond cleavage/formation depicted in the upper part for fully protonated (left) and fully deprotonated (right) systems (III and VI).

**Fig. 8:**
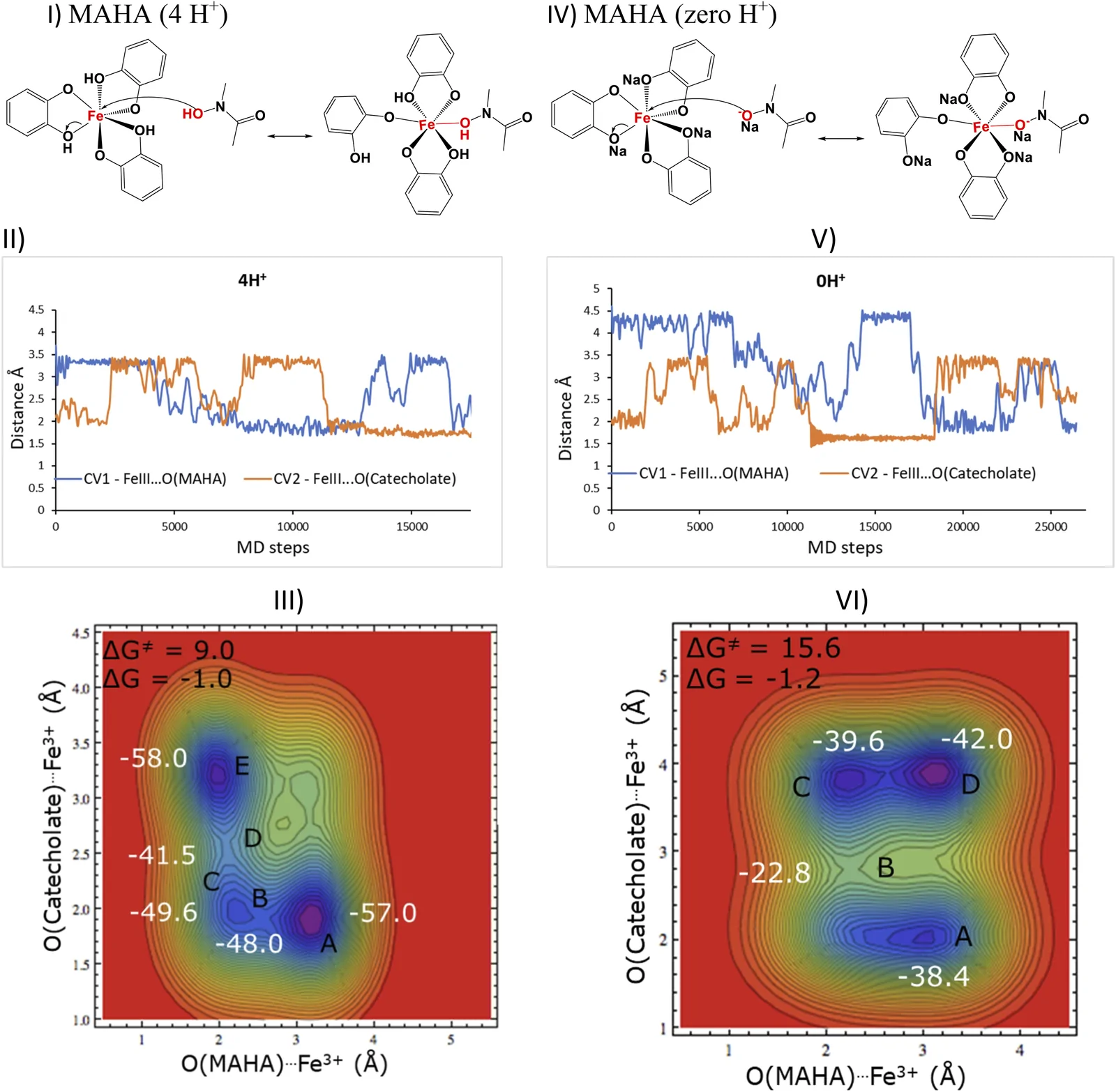
Top, schematic representation of the Fe^3+^⋯O(catecholate) bond cleavage and formation of Fe^3+^⋯O(methyl-hydroxamic(ate)) bond (I and IV), middle, the variation of the CVs (corresponding distances in Å) against the MD steps (II and V), down, the simulated free energy surface at 298 K and 1 atm for the bond cleavage/formation depicted in the upper part for fully protonated (III) and fully deprotonated (VI) systems.

The selected two cases of protonation state in each chemical system are shown in Fig. 3–8 (I–III and IV–VI, protonated and deprotonated, respectively). This means that either all the conjugate base anions are protonated (with nH^+^ in I–III) or are associated to Na^+^ cations (0H^+^ in VI–IV). Hence, the free energy difference between fully protonated and fully deprotonated can be compared. In each MTD simulation CV1 is the distance between Fe^3+^⋯O(external Ligand) and CV2 is the distance between Fe^3+^⋯O(catecholate). We follow a nucleophilic substitution mechanism, *i.e.*, the (catecholate)O⋯Fe^3+^⋯O(external ligand) bond cleavage and bond formation occur alongside each MD step. As shown in the middle part of the Fig. 3–8, multiple transitions between the reactant and product states can be observed that indicate convergence of the obtained FES. The numbers on each FES show the energies of the corresponding states in kcal mol^−1^ and the A–E labels indicate each state (reactant complex, transition state, intermediate, product complex) on the FES.

In Fig. 3(I–VI), the substitution reaction of catecholate with THFDCA is depicted for both fully protonated (I–III) and deprotonated (IV–VI) cases. As depicted in Fig. 3(III) the FES for the THFDCA, two steps for substituting one catecholate⋯Fe^3+^ with the O^−^(THFDCA) are identified. Starting from a long Fe^3+^⋯O(THFDCA) and a short Fe^3+^⋯O(catecholate) (state A), first a bond between Fe^3+^-O(THFDCA) (state B) forms. This is a ternary species between two ligands (catecholate and THFDCA, highlighted regions in 3II and 3V), which was reported in previous studies.^43^ Then the Fe^3+^⋯O(catecholate) distance increases and this bond cleavages as the second step. As observed in the FES the second step has a higher barrier transition state (state C) than the bond formation between THFDCA and Fe^3+^ and hence is the rate determining step (7.0 kcal mol^−1^*vs.* 1.7 kcal mol^−1^). The product state (E) is thermodynamically favorable in this reaction (−8.6 kcal mol^−1^). In Fig. 3-VI, we show the fully deprotonated FES for the substitution reaction of catecholate with THFDCA. Reactant state A is more stable than the product state C (Δ*G* of 4.8 kcal mol^−1^), which is rather a flat area, and the transition state barrier (17.0 kcal mol^−1^ state B) is higher than the corresponding transition state of fully protonated case (Fig. 3(III), 7.0 kcal mol^−1^). Therefore, within the fully protonated model, the barrier of the catecholate-THFDCA substitution is significantly lower (10.0 kcal mol^−1^) and results in more favorable thermodynamics of reaction relative to the deprotonated complex (Fig. 3IV–VI). As observed in the middle part of Fig. 3, bond formation (blue line, CV1) occurs earlier in case of fully protonated system (3II) *versus* fully deprotonated system (3V), *i.e.* MD steps *ca.* 3000 *vs. ca* 11 600.

In Fig. 4, we have depicted the catecholate and FDCA exchange reaction for both fully protonated and deprotonated reaction conditions (I–VI). As can be observed in Fig. 4III, starting from the state A, similar to the pathway that was observed for fully protonated THFDCA, there is a two-step pathway with a low barrier for Fe^3+^⋯O(FDCA) bond formation (5.6 kcal mol^−1^) and a high barrier of 20.9 kcal mol^−1^ for Fe^3+^⋯O(catecholate) bond cleavage, which is the rate determining step. In case of FDCA (5H^+^) the ligand exchange reaction is not as favorable as the THFDCA (5H^+^) and has a Δ*G* of +7.6 kcal mol^−1^*vs.* −8.6 kcal mol^−1^, respectively. Fig. 4(II) shows formation of a stable bond between FDCA and Fe^3+^ occurs later than THFDCA, *i.e.*, at 9000 *vs.* 3000 MD steps, respectively. The less favorable kinetics and thermodynamics of the FDCA-catecholate exchange *vs.* THFDA can be due to either weaker nucleophilicity of the FDCA with respect to THFDCA or higher steric hindrance effects at the transition state structure. This is investigated by energy decomposition analysis (EDA) and frontier molecular orbitals (FMO) later on in this work (*vide infra*). Fig. 4(VI) shows the deprotonated system has an entirely different pathway *versus* protonated structure that is starting from the reactant state A and passing through a *ca.* 20 kcal mol^−1^ transition state B and intermediate C forms the product state E. At the intermediate C the initial Fe^3+^⋯O(catecholate) is cleaved but the Fe^3+^⋯O(FDCA) not formed, yet, and through a rather small barrier (4.2 kcal mol^−1^) the product is formed (state E). This transformation is thermodynamically less favorable than the fully protonated system (14.7 *vs.* 7.6 kcal mol^−1^, respectively). However, comparison of the energetics exhibits similar kinetics in both protonated and deprotonated systems (20.9 *vs.* 19.8 kcal mol^−1^). The unfavorable thermodynamics of this transformation results in a reversible FDCA-catecholate exchange which may not be desired in experiment, due to the lower stability of the product molecular complex.

In Fig. 5 we perform the same analysis for the THFDCH ligand to estimate the possibility of Fe^3+^⋯O(catecholate) bond cleavage with this external ligand as well. As illustrated in Fig. 5(III) the FES is very flat, *i.e.*, only a barrier of 1.9 kcal mol^−1^ and a stable thermodynamic with a Δ*G* of −9.5 kcal mol^−1^-that means THFDCH acid can easily replace catecholate, with a favorable reaction free energy.^44^ In the deprotonated case *i.e.* the conjugate base of THFDCH, the deprotonated system has a high barrier of exchange (20 kcal mol^−1^) and a shallow FES at the product side (state C, Δ*G* = 16.0 kcal mol^−1^) which may indicate the reversible catecholate–Fe^3+^ bond formation.

In Fig. 6 we have shown the Fe^3+^⋯O(catecholate) bond cleavage with FDCH external ligand. As observed by comparison of the left and right parts, two different paths are identified, *i.e.*, in the fully protonated case (III) bond cleavage and bond formation occur almost simultaneously with a barrier of 10.0 kcal mol^−1^ (transition state D). However, in case of fully deprotonated system (VI) it appears that the bond cleavage between the catecholate ligand and Fe^3+^ takes place first with a TS barrier of 6.0 kcal mol^−1^ (A to C).Then bond formation between Fe^3+^⋯O(external ligand) which needs 9.0 kcal mol^−1^ (C to E) occurs. The overall barrier is 14.0 kcal mol^−1^. Similar to the FDCA the barrier for fully deprotonated system is almost similar to the fully protonated case However, in comparison to the FDCA the barrier is lower. Additionally, thermodynamics of exchange is more exergonic than FDCA, (1.0 and −4.0 (in FDCH) *vs.* 7.6 and 14 kcal mol^−1^ for protonate and deprotonated cases, respectively. The late formation of the Fe^3+…^O(FDCH) with respect to the corresponding Fe^3+…^O(THFDCH), *i.e.*, *ca.* 2400 *vs.* 15 000 MD steps, is similar to THFDCA/FDCA.

In Fig. 7, the analysis for AHA (acetohydroxamic(ate) acid) is shown. The barrier and reaction free energy are 10.4/−2.3 kcal mol^−1^ for the protonated and 16.0/11.2 kcal mol^−1^, respectively, for deprotonated system. The simulated FESs show a preference for the catecholate demetallation at the fully protonated regime, which is not favorable thermodynamically at the fully deprotonated case.

In Fig. 8 the external ligand is MAHA, to mimic one of the active sites of the long DFOB chain (Fig. 1). The fully protonated (I) and fully deprotonated (IV) systems are analyzed. Starting from the reactant state A in fully protonated system (III), a bond between the Fe^3+^⋯O(hydroxamic) at state C (with a barrier of 9.0 kcal mol^−1^) is formed and then Fe^3+^⋯O(catecholate) bond at the state D is cleaved with a barrier of 8.1 kcal mol^−1^. The thermodynamics of the exchange is slightly in favor of hydroxamic O⋯Fe^3+^ (−1.0 kcal mol^−1^). In the right panel of Fig. 8 we have analyzed the deprotonated MAHA system. As seen, the energetics in 8-III and 8-VI are not similar, *i.e.*, first, in the fully deprotonated 8-VI the barrier is higher (15.6 *vs.* 9.0), and second, bond formation and cleavage are simultaneously. At the state C the hydroxamate bond is formed and the catecholate is cleaved.

### Ligand comparison

The above analysis discussed based on the FESs and the ligand exchange energetics is summarized in Fig. 9. Fig. 9 shows that in the above mentioned molecular systems the overall barriers of the deprotonated molecular systems are higher than the protonated molecular systems, *i.e.*, up to 20 kcal mol^−1^ for the state B in Fig. 9. Visualization of the MD trajectories indicates that formation of the intermolecular H-bonds in the protonated molecular systems stabilizes the intermediate and transition states and causes an overall lower energetics of the ligands exchange transformation. In some cases, such as THFDCH and FDCH the shallow FES (D to E) identifies the reaction reversibility. In FDCA, both protonated and deprotonated systems have similar high barriers for the exchange of catecholate. Furthermore, the less favorable thermodynamics in case of the fully deprotonated FDCA can also result in reversibility of the reaction. As Fig. 9A shows, FDCA has the highest energetics with respect to the other ligands, while THFDCA and THFDCH have the lowest barriers. AHA has a lower barrier than the MAHA (light blue *vs.* green, respectively, Fig. 9A). In the deprotonated systems (Fig. 9B), THFDCA, MAHA, and THFDCH (dark blue, green and pink, respectively) exhibit a concerted exchange pathway in which Fe–O(catecholate) bond cleavage and Fe–O(incoming ligand) bond formation occur within the same region of the free-energy surface, without a clearly resolved intermediate. We therefore describe these pathways as concerted interchange processes. In contrast, FDCH, FDCA, and AHA (blue, orange and purple) display a stepwise pathway involving a distinguishable intermediate. Only in case of MAHA and FDCH the overall free energy of the reaction is favorable amongst the deprotonated systems.

**Fig. 9:**
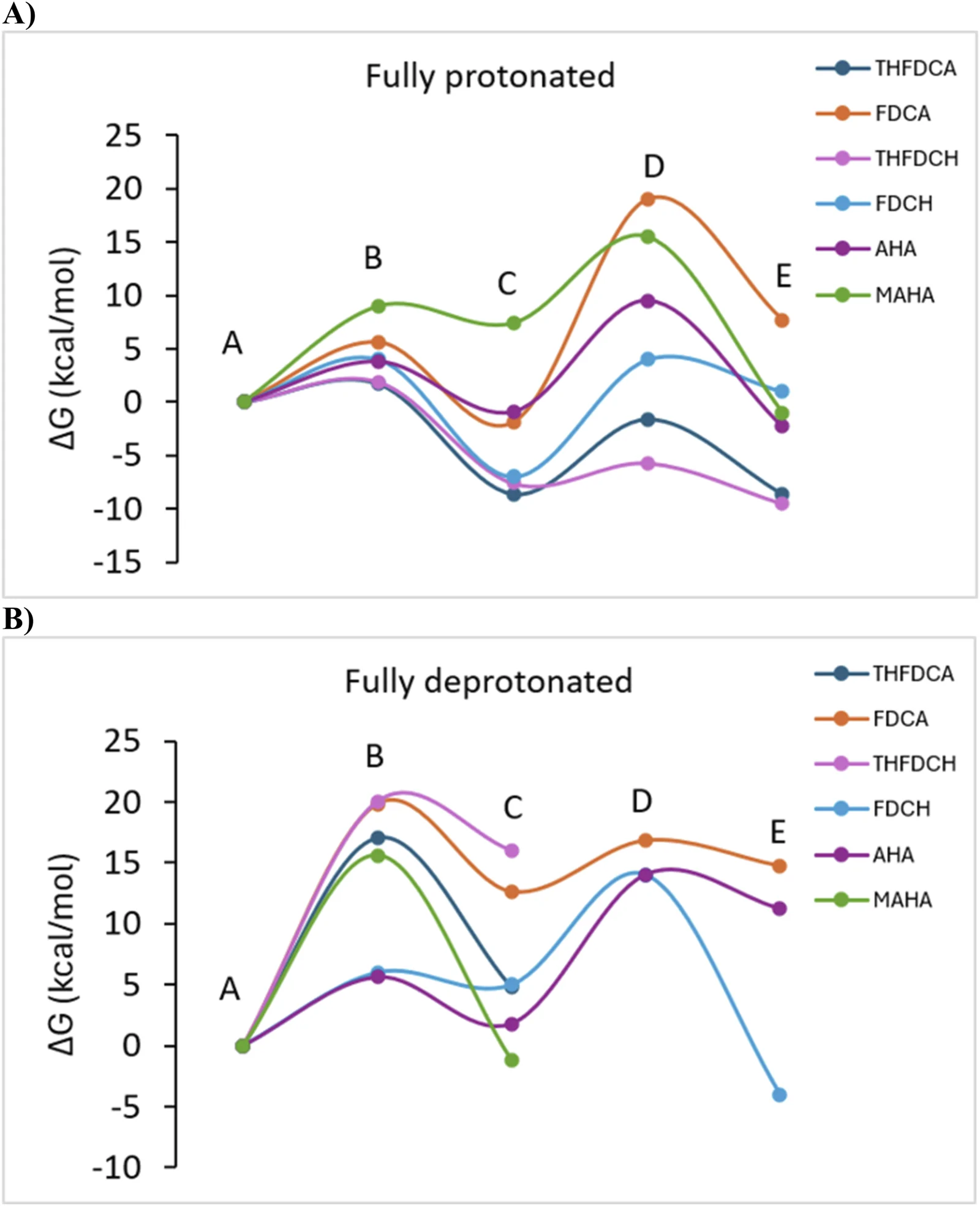
The free energy profiles comparing all ligands for exchange between the catecholate and external ligands (Fig. 2), (A) protonated and (B) deprotonated molecular systems.

To summarize, the initial molecular complex with catecholate⋯Fe^3+^ bonds has either OH covalent bonds with O-donators coordinating to Fe^3+^ (protonated system) or ionic Na^+^⋯^−^O with O^−^ donating to the iron center (deprotonated system). The calculated FESs at room temperature illustrate that the energetics of protonated and deprotonated forms of the ligands are different, *i.e.*, in general for the entire series of selected ligands the deprotonated systems indicate higher barrier and less favorable thermodynamics. In addition, there is a large difference between energetics of THFDCA/THFDCH and FDCA/FDCH to replace catecholate from Fe^3+^ complexation, which is in line with experiments.^45,46^Table 2 summarizes the comparison between computational predictions and available experimental observations.

**Table 2:** Comparison between computational predictions and available experimental observations

Ligand	Computational prediction	Experimental observation	Agreement
THFDCA	Low activation free-energy barrier; favorable exchange thermodynamics	High stain-removal performance reported experimentally	Consistent trend
FDCA	Higher barrier; less favorable exchange thermodynamics	Lower performance than THFDCA	Consistent trend
THFDCH	Low barrier; favorable product stability; possible reversibility	Reversible chelation reported experimentally	Qualitative agreement
FDCH	Intermediate barrier; relatively flat free-energy surface	Reversible ligand exchange observed experimentally	Qualitative agreement
AHA	Moderate barrier and weak thermodynamic driving force	Limited available experimental data	Not evaluated
MAHA	Moderate barrier; favorable deprotonated exchange thermodynamics	Limited available experimental data	Not evaluated

The available experimental measurements primarily reflect overall stain-removal performance under application-relevant conditions and do not directly provide activation free energies, transition-state structures, or detailed microscopic ligand–exchange pathways. Consequently, the present comparison should be regarded as a validation of relative trends rather than a quantitative validation of the calculated free-energy barriers or mechanistic assignments.

### Mechanistic insights

Fig. 9A indicates that state C in the FESs is a more stable intermediate state in THFDCA, THFDCH and FDCH, which both catecholate and external ligands are associated with the Fe^3+^ (both Fe^3+^⋯O distances are smaller than 3 Å). This association is stronger in THFDCA, THFDCH and FDCH than FDCA and AHA. In MAHA the state C has a shallow FES and a poor association state.

The calculated pathways reveal a continuum between associative and dissociative substitution rather than a uniform mechanism determined solely by protonation state. For pathways such as protonated THFDCA, coordination of the incoming ligand occurs before substantial Fe–O(catecholate) dissociation, producing a transient species in which both ligands interact with the Fe^3+^ center. These pathways exhibit associative character because formation of the Fe–O interaction with the incoming ligand precedes cleavage of the exchanging Fe–O(catecholate) bond consequently display predominantly associative character. Conversely, pathways in which Fe–O(catecholate) cleavage substantially precedes formation of the new Fe–O(ligand) interaction display predominantly dissociative character such as deprotonated FDCA (ionic environment). For systems in which Fe–O bond breaking and formation occur concertedly without a clearly resolved higher- or lower-coordinate intermediate, we use the more conservative description of an interchange pathway. Thus, the mechanistic assignments are based on the evolution of Fe–O distances and the Fe coordination environment along the calculated reaction coordinate. The effect of ionic environment on the type of dissociative mechanism was reported in the previous studies.^63^

### Influence of ligand electronic structures

To understand the electronic-structure rationale for the relative energetics of the specific static configurations of different performances of THFDCA and FDCA in exchange of catecholate, we performed electronic structure analyses including Energy Decomposition Analysis (EDA) and Frontier Molecular Orbital (FMO) analysis on the molecular structures at the ground and transition states of these ligands. Only THFDCA and FDCA were selected for the EDA because they constitute a structurally related pair that enables the electronic consequences of their structural difference to be examined while limiting changes in other molecular features. The purpose of this analysis is therefore a focused comparison rather than a systematic decomposition of the entire ligand series. We note that the metadynamics and static DFT calculations provide complementary but distinct descriptions of the ligand-exchange process. The metadynamics free-energy surfaces represent finite-temperature free energies projected onto the selected collective variables, and the corresponding barrier regions therefore comprise ensembles of configurations. By contrast, the constrained static DFT calculations provide static potential–energy profiles for selected structures along the ligand-exchange coordinate. Consequently, structures near the maxima of the constrained potential–energy profiles are used here as representative barrier-region configurations for structural and electronic analysis and are not assumed to be identical to the transition-state ensemble sampled in metadynamics. Similarly, the EDA performed for these static configurations provides insight into changes in electronic interactions along the exchange pathway but does not constitute a decomposition of the metadynamics activation free energy. In summary, metadynamics establishes the finite-temperature free-energy landscape, while static DFT and EDA provide complementary structural and electronic interpretation of representative configurations associated with the ligand-exchange barrier region. The initial molecular complex with six catecholate⋯Fe^3+^ bonds has either OH bonds with O-donators coordinating to Fe^3+^ (protonated system) or ionic Na^+^⋯^−^O with O^−^ donating to the iron center (deprotonated system). To understand which of the protonated or the deprotonated system has stronger Fe^3+^⋯^−^O bonds (that causes higher energy of bond-cleavage) catecholate-Fe^3+^ molecular complex is divided into two fragments one is Fe^3+^ and the other is the three catecholate ligands as one fragment. The EDA results are shown in Table 3. According to Table 3, there is very strong electrostatic interaction between Fe^3+^ cation and the catecholate anions due to the ionic interactions between these two fragments. This consequently results in a very strong orbital interaction caused by charge transfer between HOMO–LUMO of catecholate and Fe^3+^ fragments. The total interaction is 41.77 kcal mol^−1^ stronger in the deprotonated molecular complex that means in principle the Fe–O bonds are stronger than their corresponding analogues in the protonated system. The stronger interaction calculated for the deprotonated complex is consistent with greater resistance to Fe–O bond cleavage and therefore provides a qualitative electronic-structure rationale for its higher calculated ligand-exchange barrier. Fig. 10 shows the molecular complexes for protonated and deprotonated cases. The longer Fe^3+^⋯O bond distances in protonated molecular complex is an indication of weaker interaction between catecholate and iron fragments (2.05 Å *vs.* 1.90 Å).

**Table 3:** The EDA results for molecular complex of Fe^3+^ and catecholate for the molecular structures depicted in Fig. 10

Energy (kcal mol^−1^)	Fe^3+^⋯catecholate (protonated)	Fe^3+^⋯catecholate (deprotonated)
Pauli repulsion	294.82	295.00
Electrostatic interaction	−1124.59	−1069.68
Total steric interaction	−829.77	−774.68
Orbital interaction	−887.42	−982.13
Dispersion	−9.49	−11.65
Total interaction	−1726.70	−1768.47

**Fig. 10:**
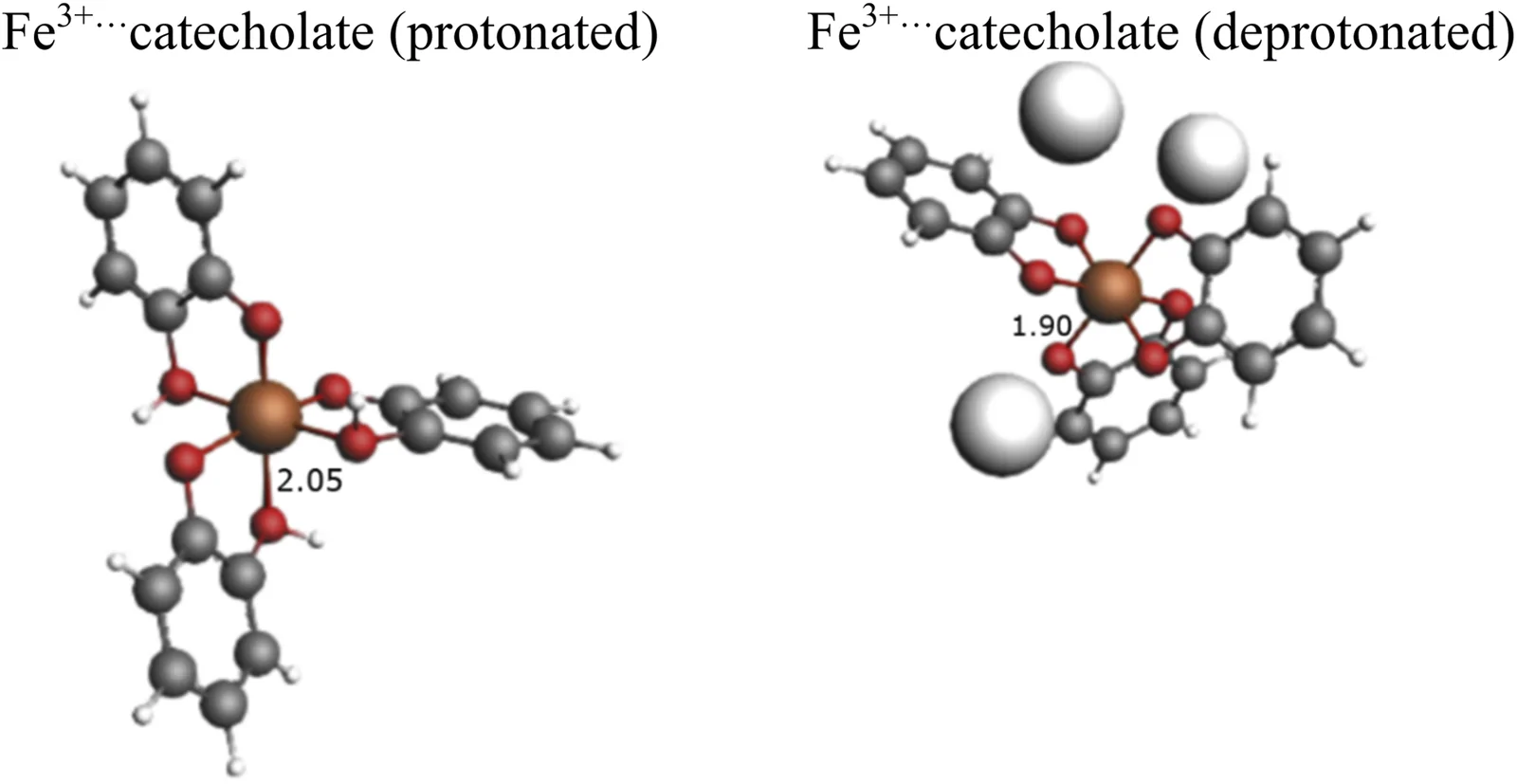
Molecular complexes of catecholate Fe^3+^ for protonated and deprotonated systems. In the deprotonated molecule the Na^+^ cations compensate the negative charge.

For better understanding of the large difference between the barriers of protonated THFDCA and FDCA systems the EDA is performed on the transition states obtained by linear transit (static DFT) calculations to form/cleave the corresponding bonds. The EDA results at the transition state structures are summarized in Table 4.

**Table 4:** The EDA results for the TS structures of protonated THFDCA and FDCA molecular systems (obtained by static DFT and linear transit calculations). The molecular complex is divided into two fragments including the external ligand and the entire catecholate-Fe^3+^ fragment

EDA at the TS structures (energies in kcal mol^−1^)	THFDCA-fully protonated	FDCA-fully protonated
TS barrier (calc. in gas phase)	10.0	19.31
Pauli repulsion	31.73	49.08
Electrostatic interaction	−26.99	−35.26
Total steric interaction	4.74	13.82
Orbital interaction	−20.96	−25.27
Dispersion	−11.17	−9.77
Total interaction	−27.39	−21.22
Total strain (deformation)	37.39	40.53

According to Table 4, the EDA of the TS structures suggests that the higher barrier observed for FDCA relative to THFDCA is associated with greater Pauli repulsion and a somewhat larger deformation energy in the FDCA system. These results are in agreement with a later TS for the FDCA (shorter distance) than the THFDCA molecular systems as shown in Fig. 11, *i.e.*, the Fe^3+^⋯O(THFDCA) is 2.93 Å and Fe^3+^⋯O(FDCA) is 2.79 Å. The stronger orbital interaction (covalent character) in FDCA transition state, *ca.* 4.31 kcal mol^−1^, also indicates a later TS.

**Fig. 11:**
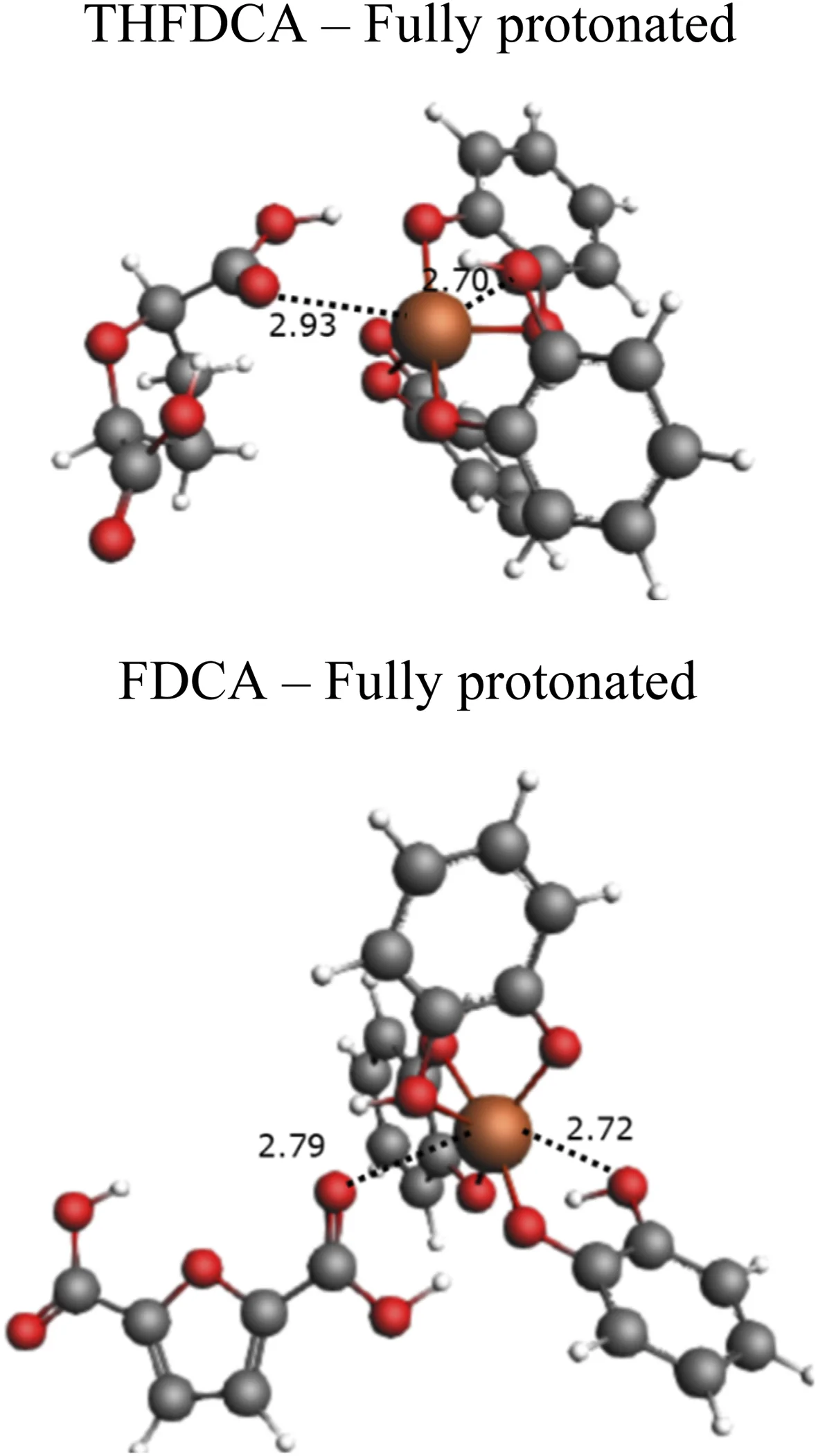
The TS structures of the fully protonated THFDCA and FDCA molecular system obtained by the linear transit calculations.

In Fig. 12 the main interacting FMOs of the catecholate-Fe^3+^ (LUMO) and the external ligands (HOMO) are depicted. The numbers show the energy levels of the FMOs and the number in parentheses show the HOMO–LUMO gaps. Fig. 12 shows the LUMO of catecholate-Fe^3+^ is mainly spread over the Fe–O bonds and the HOMO of the external ligands is localized on the COO and –C=O(N)–O nucleophilic sites. The highest HOMO–LUMO gap is correlated to the FDCA external ligand (that has the most unfavorable energetics amongst the other ligands).

**Fig. 12:**
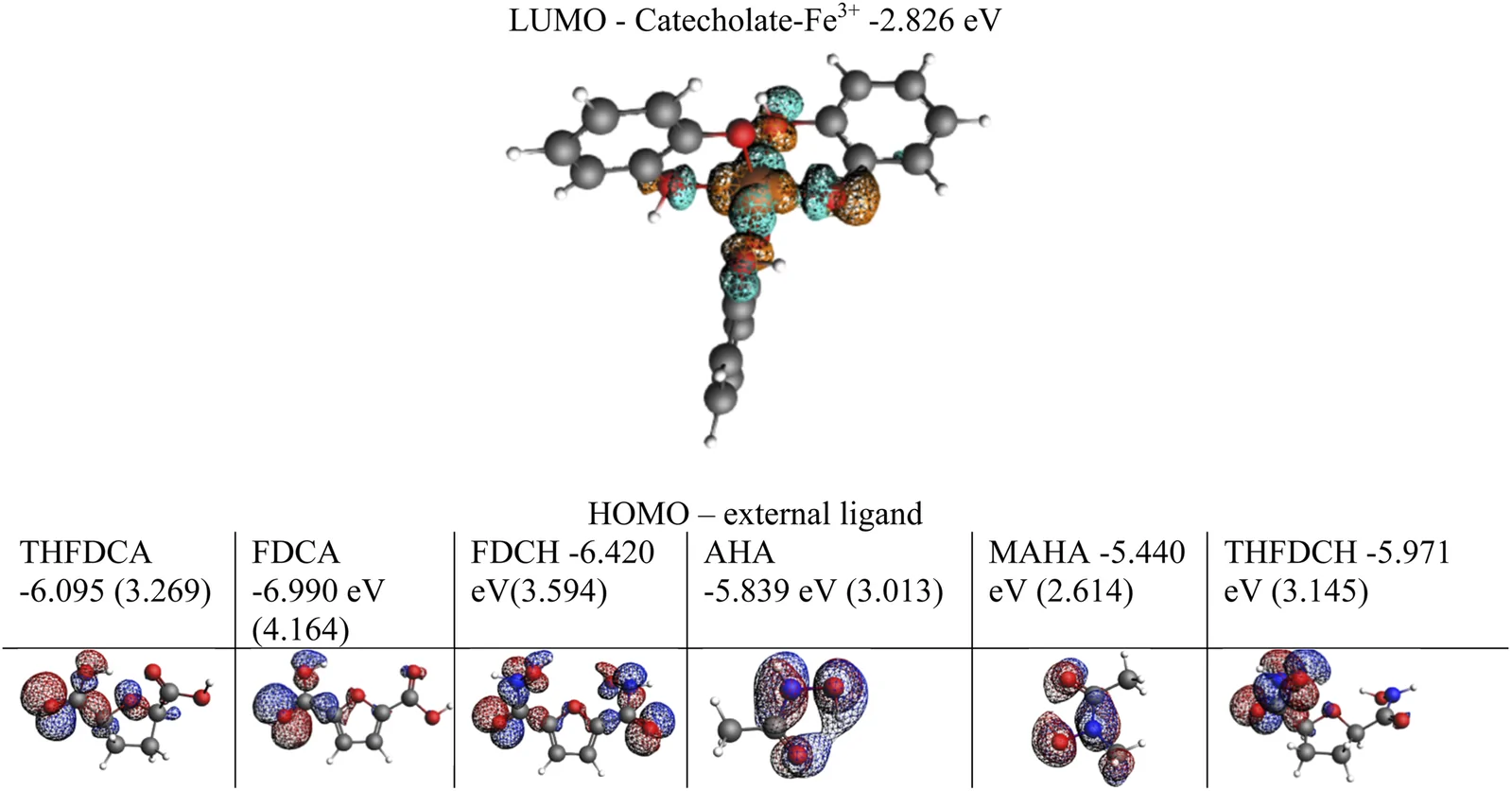
Catecholate-Fe^3+^ LUMO is depicted in comparison to the external ligands HOMOs (isosurface value 0.03). The numbers show the energy levels of the FMOs in eV and the number in parentheses show the HOMO–LUMO gaps.

## Conclusions

In this work, we investigated ligand-exchange reactions involving Fe^3+^–catecholate complexes and a series of bio-inspired competing ligands. These included furan dicarboxylic acid (FDCA), its hydrogenated analogue tetrahydrofuran dicarboxylic acid (THFDCA), and their corresponding hydroxamic acid derivatives (FDCH and THFDCH). In addition, two low-molecular-weight model compounds of the biological siderophore desferrioxamine B (DFOB), namely acetohydroxamic acid (AHA) and *N*-methyl acetohydroxamic acid (MAHA), were examined using *ab initio* molecular dynamics (AIMD) simulations.

The primary objective of this study was to elucidate how ligand-exchange kinetics are influenced by the electronic structure and protonation state of competing ligands—factors that have received comparatively limited attention in previous investigations. Our results demonstrate that, in addition to the thermodynamic stability of the resulting Fe^3+^–ligand complexes, the kinetics of ligand exchange play a crucial role in determining the overall replacement process.

The simulations indicate that protonation can favor pathways with greater associative character in several of the investigated systems, although the calculated pathways span a continuum between associative, dissociative, and interchange behavior. The calculated free-energy surfaces (FESs) indicate that protonation of either the catecholate ligand (forming the monoanionic species) or the incoming ligand generally results in a lower activation barrier, thereby facilitating ligand exchange. Furthermore, the increased lability of the protonated Fe^3+^–catecholate complex relative to its deprotonated counterpart further promotes the associative substitution pathway. We note that our mechanistic findings pertain to the investigated limiting protonation states as reference states rather than definitive representations of the dominant solution species.

The electronic structure of the competing ligands was also found to significantly affect ligand-exchange behavior. In particular, ligands with smaller HOMO–LUMO gaps facilitate catecholate displacement. Among the ligands investigated, THFDCA emerged as the most effective candidate within the present computational framework, consistent with the experimentally observed superior performance of THFDCA-based chelators.

Several ligands, including THFDCH and FDCH in their protonated forms, and FDCA and AHA in their deprotonated forms, exhibited relatively flat free-energy surfaces. Such behavior suggests that these compounds may serve as synergistic co-ligands, enhancing Fe^3+^ replacement when used in combination with other chelating agents.

Overall, this study provides new molecular-level insights into the roles of ligand identity, electronic structure, and protonation state in Fe^3+^ chelation and exchange processes. It should be emphasized that the present conclusions are derived from a simplified Fe^3+^–catecholate model system and do not explicitly address additional complexities encountered in real stain matrices, including competing coordination motifs, aggregation, polymerization, heterogeneous substrate interactions, and redox processes. Additionally, the concentration of the competitive ligands plays an important role in breaking the barrier of the first and second solvation shells, which in our simplified model has not been included. Consequently, the identified trends should be viewed as molecular-level hypotheses and design guidelines rather than direct predictions of detergent performance. The agreement between the calculated rankings and available experimental observations supports the utility of the computational approach for comparative ligand assessment. Nevertheless, the experimental data available at present do not constitute a direct validation of the absolute free-energy barriers or mechanistic details obtained from the simulations. These findings contribute to a deeper understanding of metal–ligand substitution mechanisms and may support the rational design and development of new, effective, and selective bio-based chelators for metal recovery and environmental applications. Future work will be required to examine more chemically complex Fe–polyphenol assemblies and competing processes that may be present in realistic applications.

## Conflicts of interest

There are no conflicts to declare.

## Supplementary Material

RA-OLF-D6RA06887A-s001

## Data Availability

The authors declare that the data supporting this article have been included as part of the supplementary information (SI). Supplementary information: tables of free energy barriers for PBE-D3 gas phase and BLYP-D3 with 12 explicit water molecules, convergence plots correlated to the FESs shown in the main text, FESs and variation of the CVs simulated at BLYP-D3 level, variation of the Fe–O distances to follow the Fe^3+^ coordination alongside the MTD trajectories, variation of the proton-oxygen distances alongside the trajectory of the molecular systems with explicit water molecules to prob proton transfers, XYZ coordinates of the molecular structures. See DOI: https://doi.org/10.1039/d6ra06887a.
