# Supplementary material for: Competitive ligand exchange of Fe3+ catecholate complexes with novel biobased chelators: a DFT-metadynamics investigation
Source: RSC Adv. 2026 Nov 11. Online ahead of print. doi: 10.1039/d6ra06887a (PMC13753976; doi:10.1039/d6ra06887a)
Supplement: Supplementary file 1 [file RA-OLF-D6RA06887A-s001.pdf]

Supplementary Information (SI) for RSC Advances.
This journal is © The Royal Society of Chemistry

Competitive Ligand Exchange of Fe^{3+} Catecholate Complexes with Novel Biobased Chelators: A DFT-Metadynamics Investigation

Mojgan Heshmat[a*], Pavlo Kostetskyy[b], Daan S. van Es[a*]

[a]Wageningen Food & Biobased Research, Bornse Weilanden 9, 6708 WG Wageningen, The Netherlands

[b]Archer Daniels Midland Company, 1001 N Brush College Road, Decatur, IL, 62521, USA

Metadynamic parameters for all ligand exchange trajectories discussed in the main text

```
UNITS LENGTH=A TIME=ps ENERGY=kcal/mol

# definition of CVs

d1: DISTANCE ATOMS=a,b

d2: DISTANCE ATOMS=a,b

METAD ARG=d1,d2  SIGMA=0.4,0.4 HEIGHT=0.35 PACE=25 LABEL=restraint

 UPPER_WALLS ARG=d1 AT=3.3  KAPPA=250.0 EXP=2 EPS=1 OFFSET=0 LABEL=uwall1

 UPPER_WALLS ARG=d2 AT=3.3  KAPPA=250.0 EXP=2 EPS=1 OFFSET=0 LABEL=uwall2

 PRINT ARG=d1,d2,restraint.bias  STRIDE=1  FILE=colvar.out
```

Table S1. Comparison of the average Fe-O distances in all molecular complexes and reaction barriers ($\Delta G^{\neq}$) of the rate determining steps of the competitive ligands calculated at two different levels of theory BLYP-D3 (only for optimized structural comparison), PBE-D3 as well as BLYP-D3 with 12 explicit water molecules. The average Fe-O distances calculated at BLYP-D3 is at most 0.05 Å larger than the calculated average at PBE-D3. However, addition of 12 water molecules to the molecular complex increases the average up to 0.2 Å.

Ligand	BLYP-D3	PBE-D3		BLYP-D3 - 12H2O	
	Avg. Fe-O Å	Avg. Fe-O Å	$\Delta G^{\neq}$	Avg. Fe-O Å	$\Delta G^{\neq}$
THFDCA (5H+)	1.97	1.93	7.0	2.09	11.2
THFDCA (0H+)	1.96	1.94	17.0	2.08	27.5
FDCA (5H+)	1.97	1.92	20.9	2.06	26.0
FDCA (0H+)	1.98	1.94	19.8	2.01	17.0
THFDCH (5H+)	2.01	2.01	1.9	2.08	9.0
THFDCH (0H+)	1.97	1.95	20.0	2.15	22.8
FDCH (5H+)	1.98	1.93	10.0	2.09	13.3
FDCH (0H+)	1.96	1.94	14.0	2.11	20.5
AHA (4H+)	1.97	1.93	10.4	2.07	14.0
AHA (0H+)	1.97	1.93	12.2	2.06	15.7
MAHA (4H+)	1.97	1.95	9.0	2.05	13.0
MAHA (0H+)	1.97	1.97	15.6	2.08	16.8

Corresponding structures to Table S1

THFDCA-5H+ PBE-D3

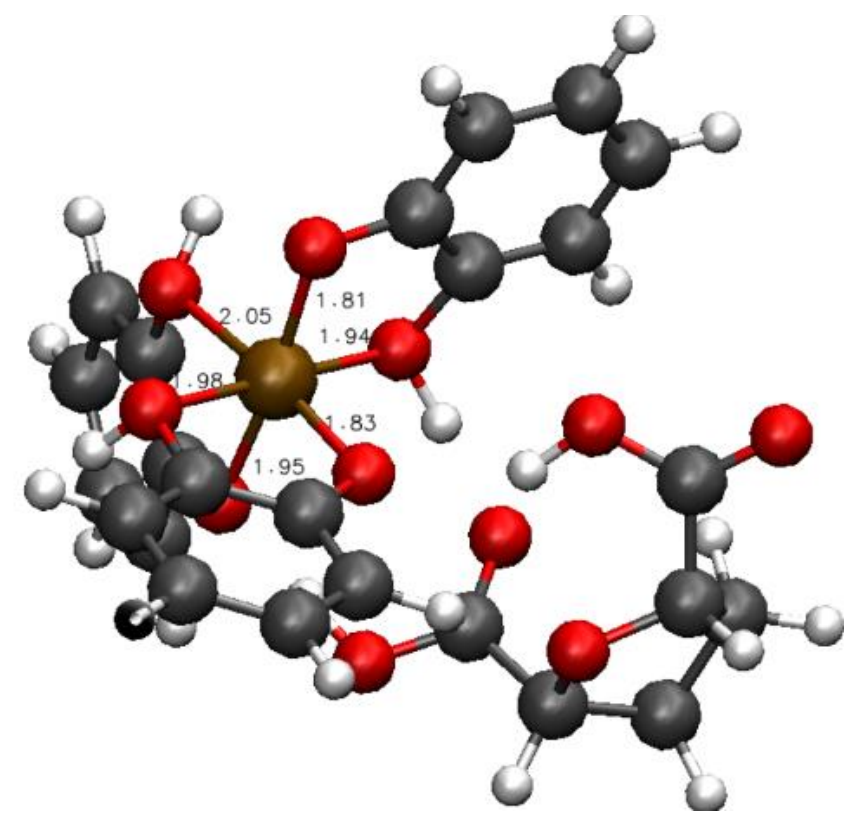

THFDCA-0H+ PBE-D3

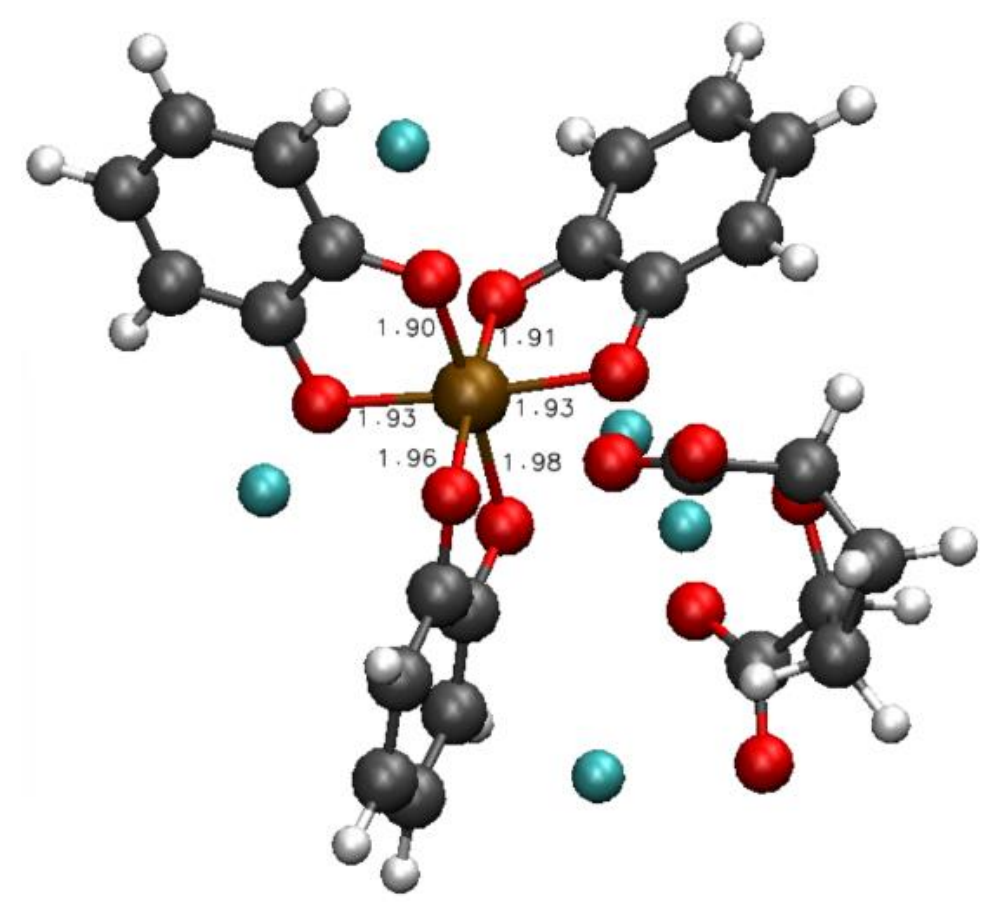

FDCA-5H+ PBE-D3

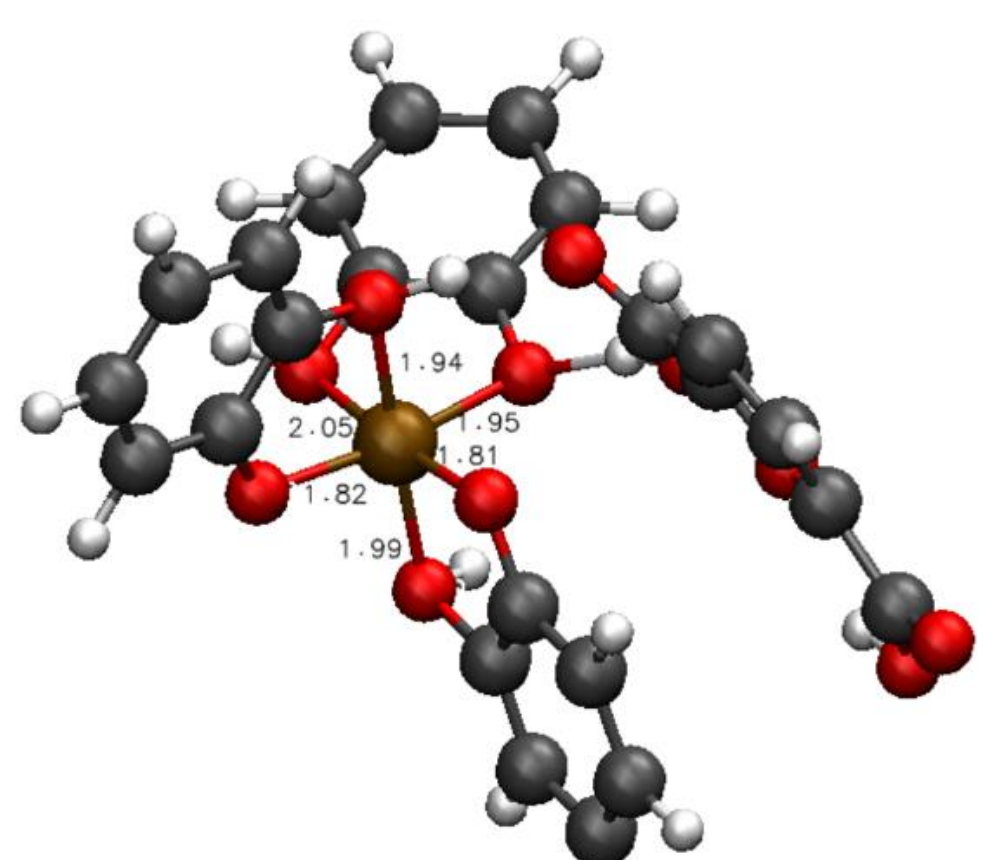

FDCA-0H+ PBE-D3

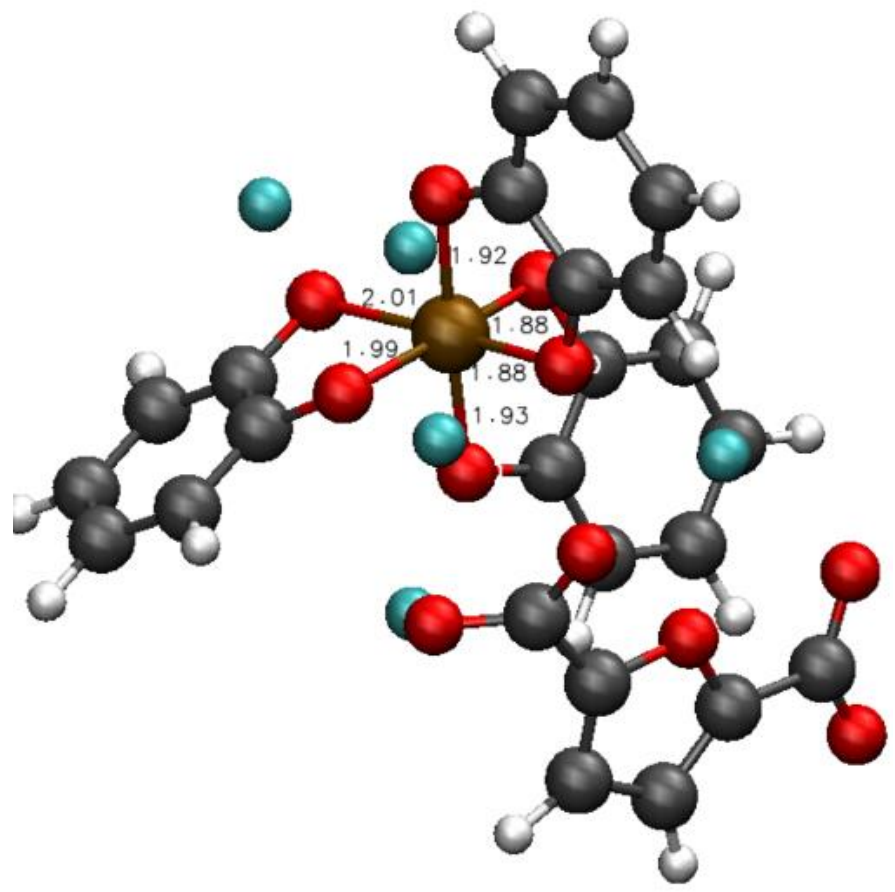

THFDCH-5H+ PBE-D3

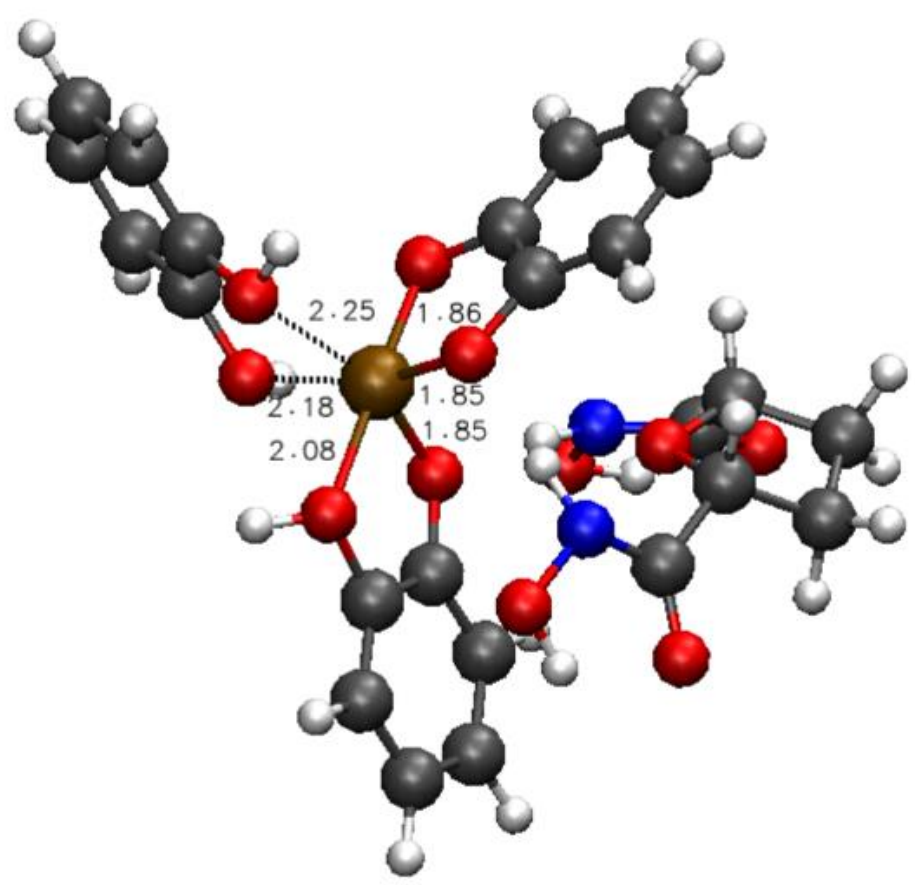

THFDCH-0H+ PBE-D3

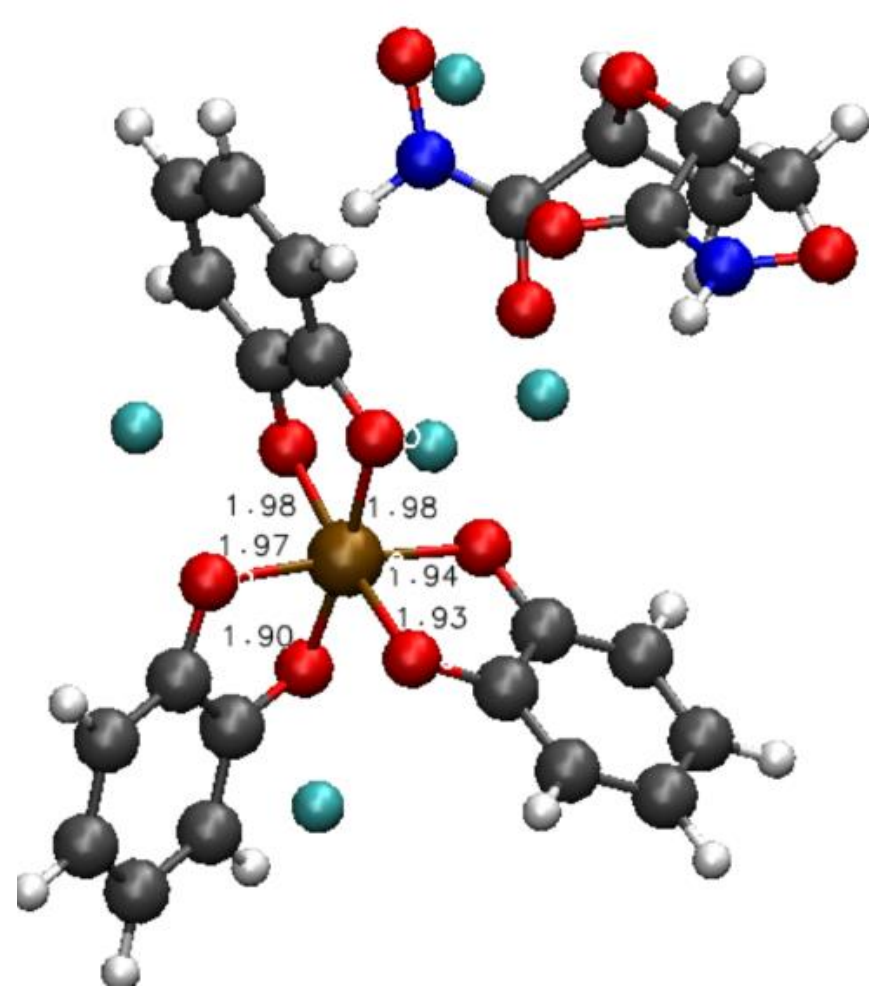

FDCH-5H+ PBE-D3

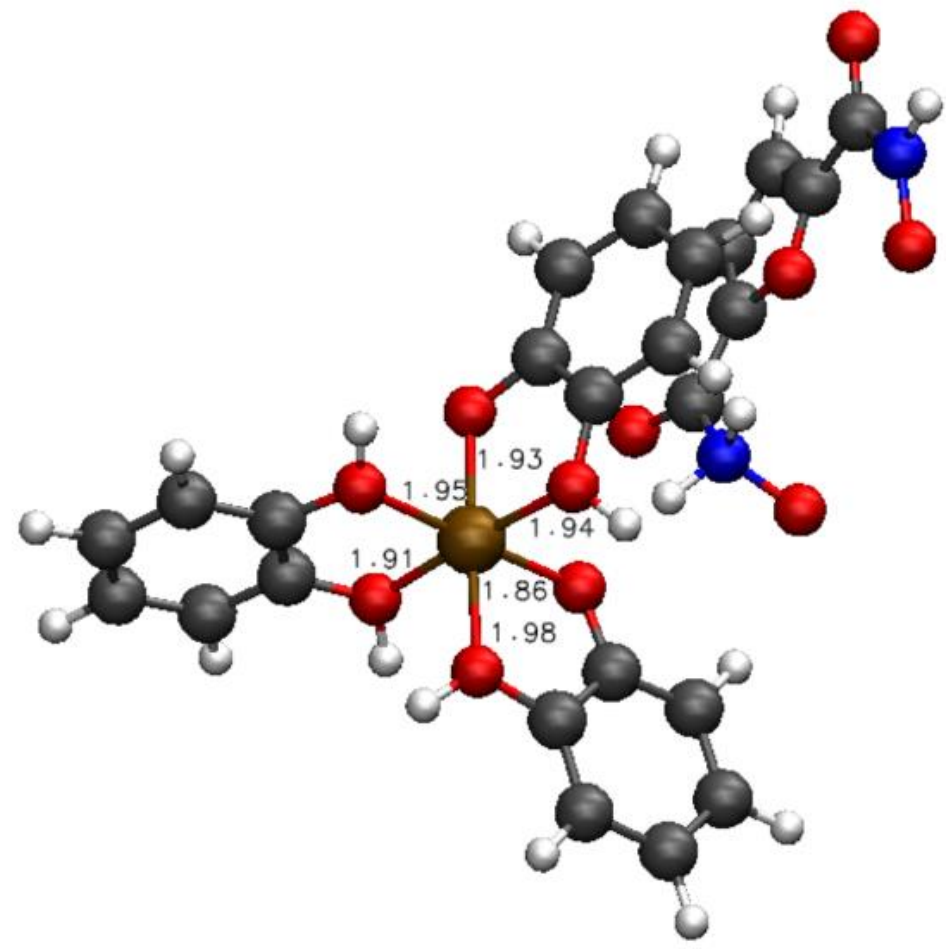

FDCH-0H+ PBE-D3

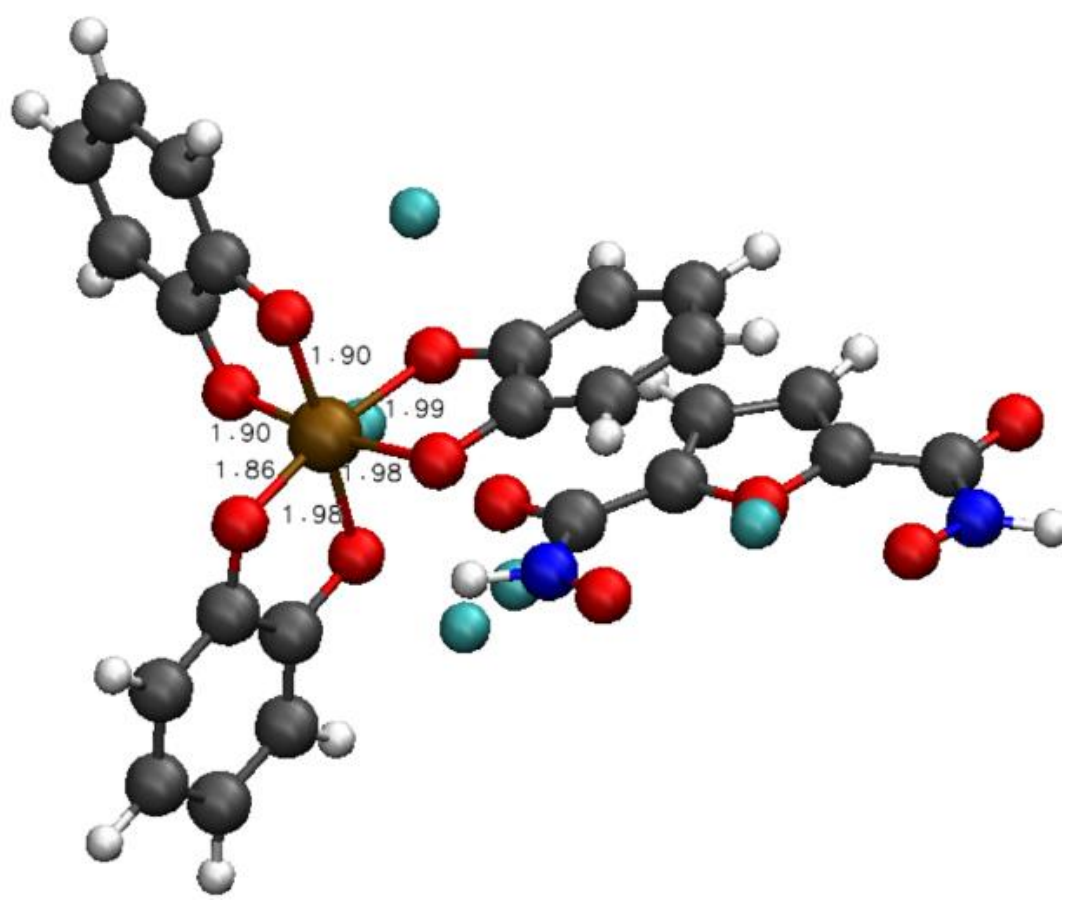

AHA-4H+ PBE-D3

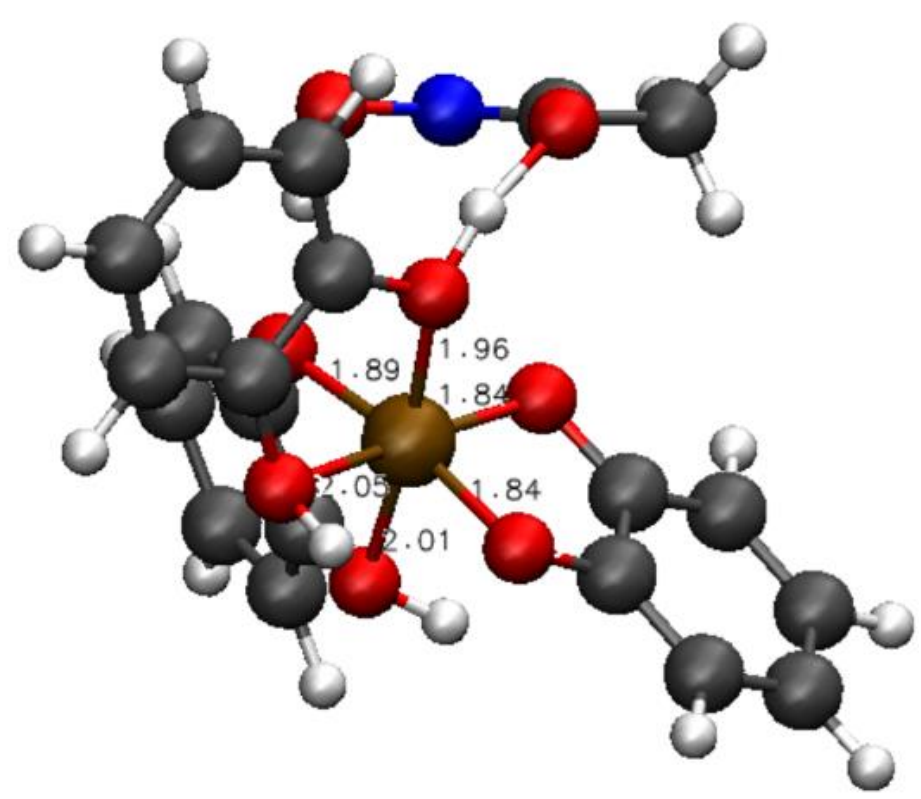

AHA-0H+ PBE-D3

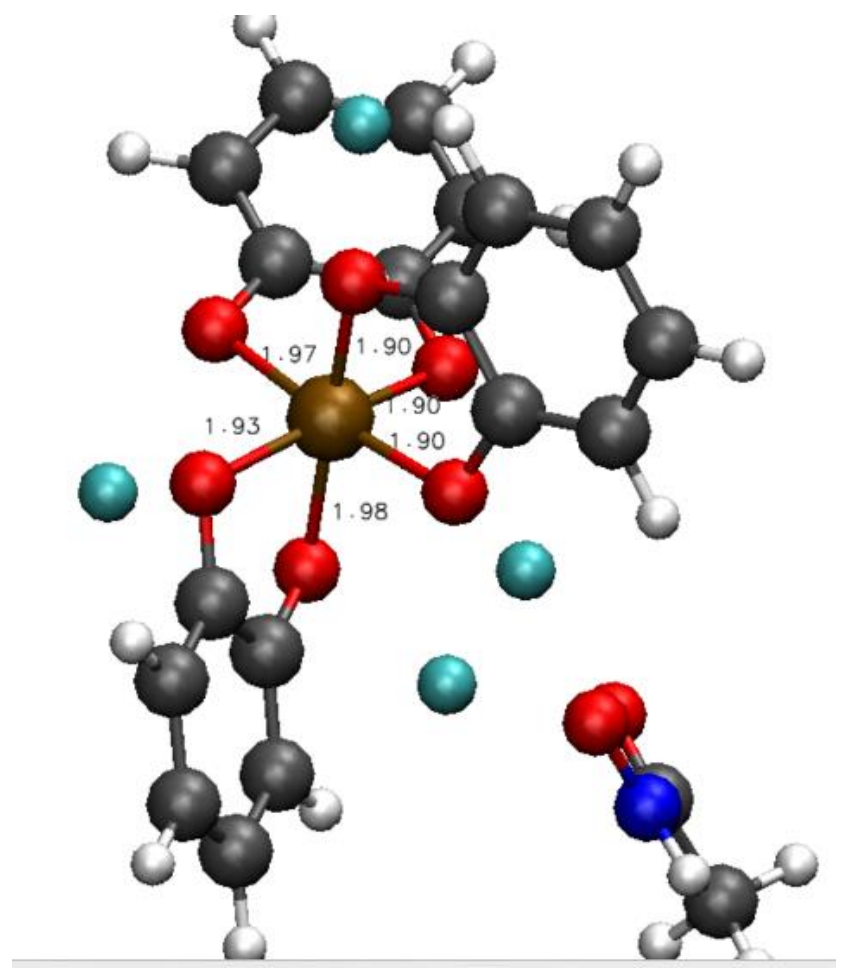

MAHA-4H+ PBE-D3

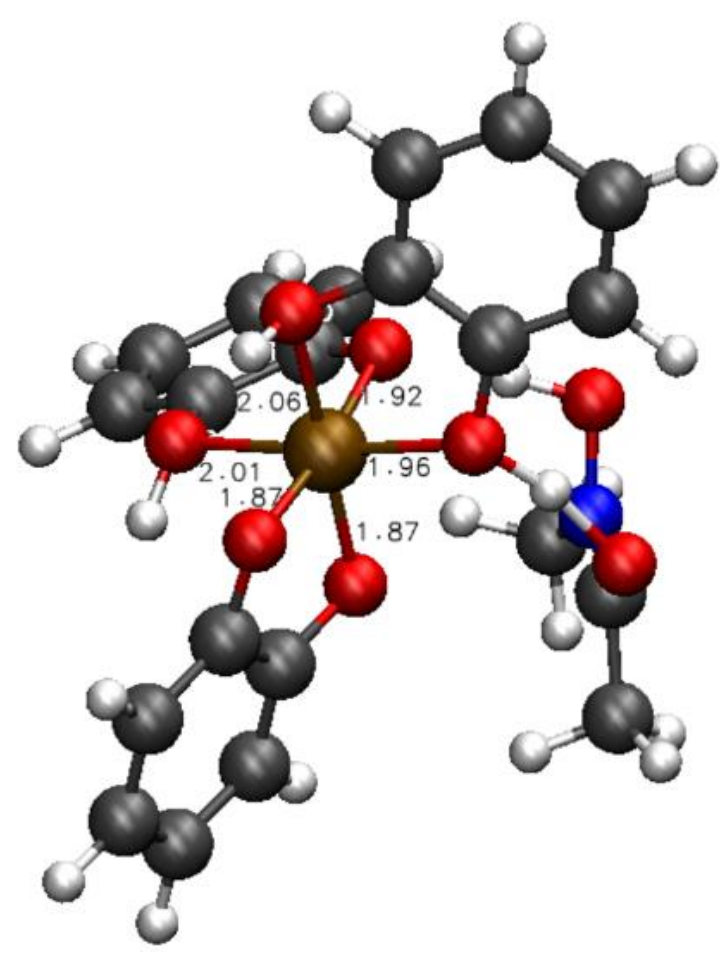

MAHA-0H+ PBE-D3

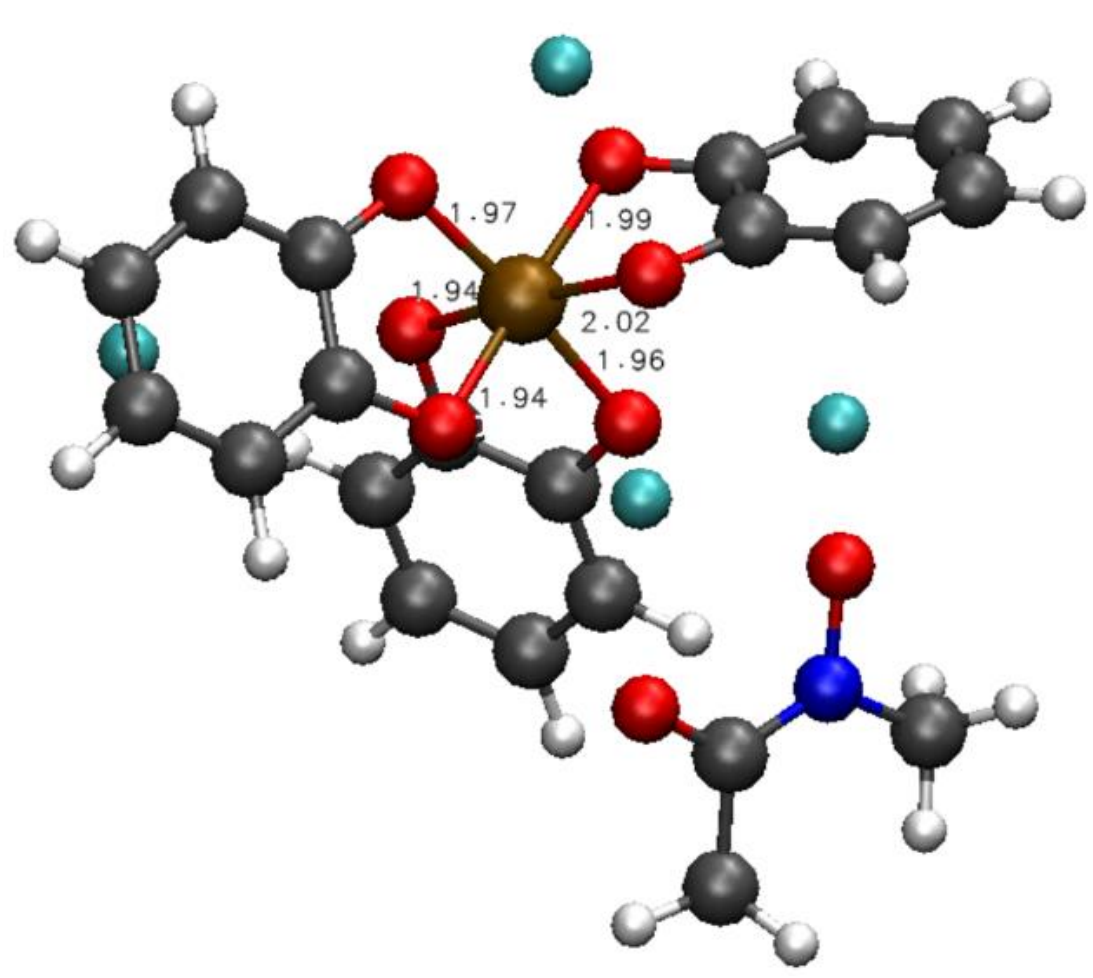

THFDCA-5H+ BLYP-D3

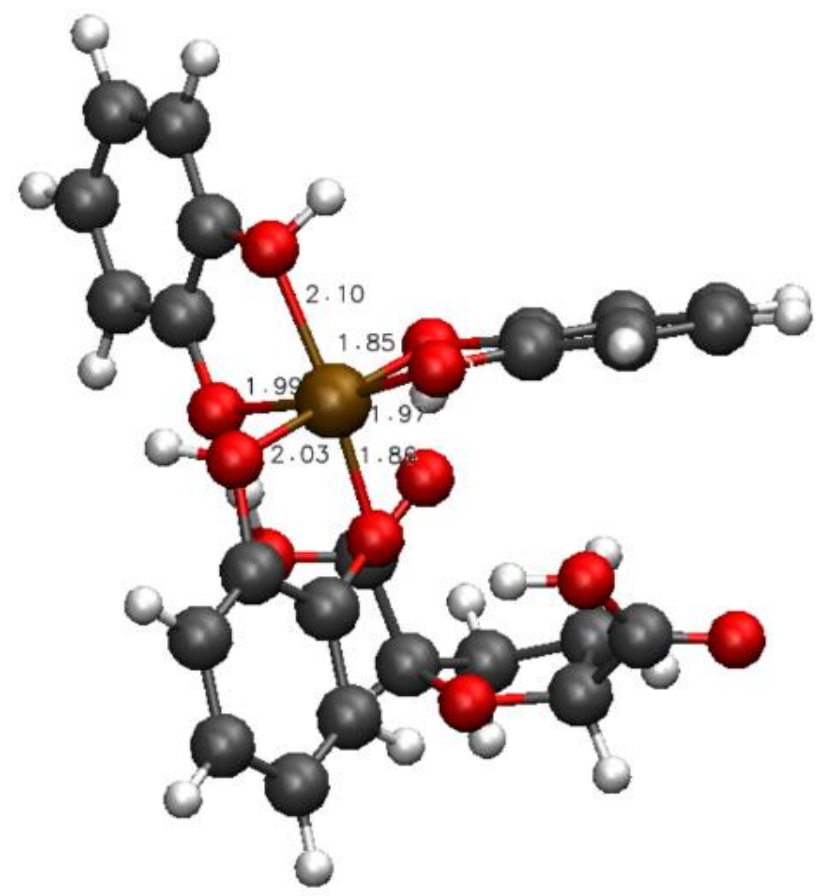

THFDCA-0H+ BLYP-D3

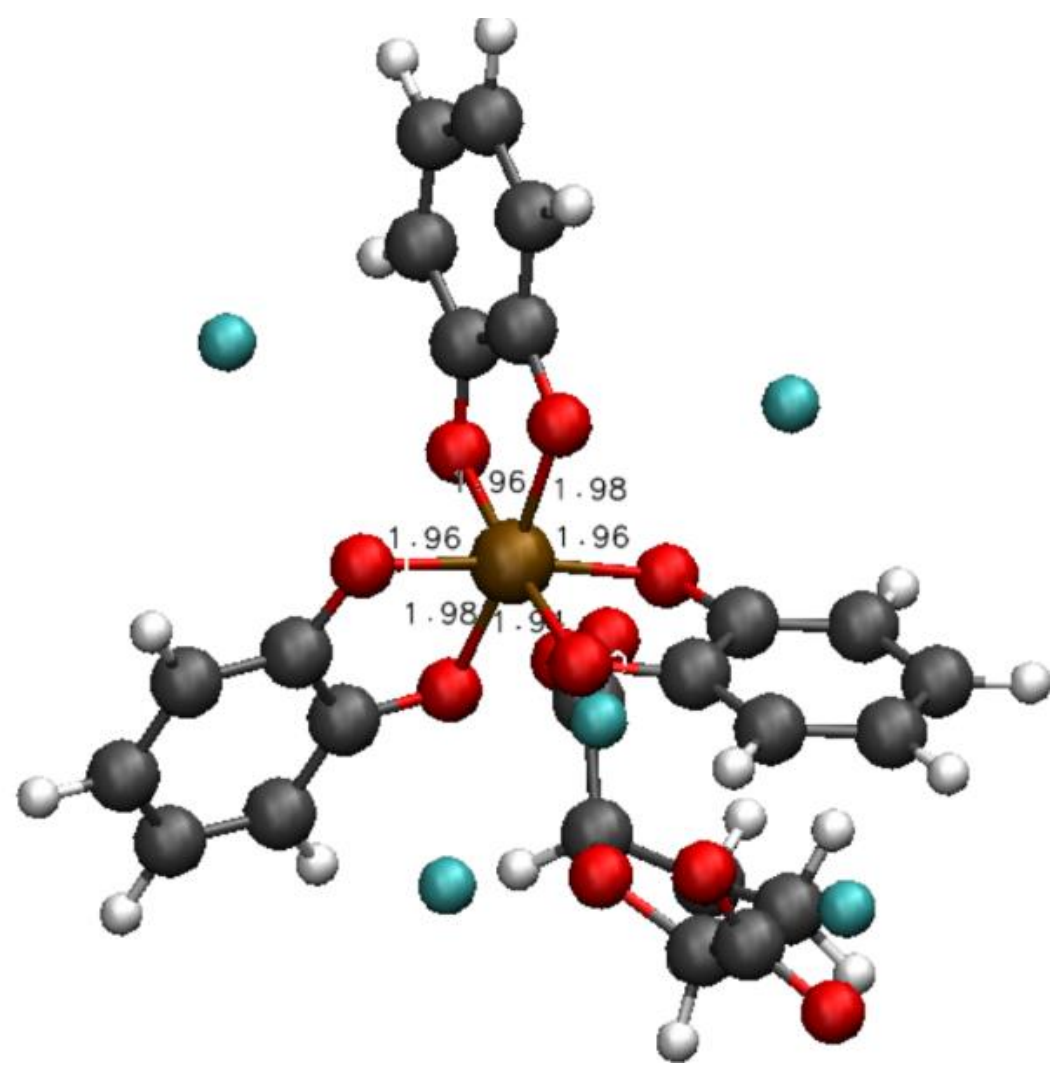

FDCA-5H+ BLYP-D3

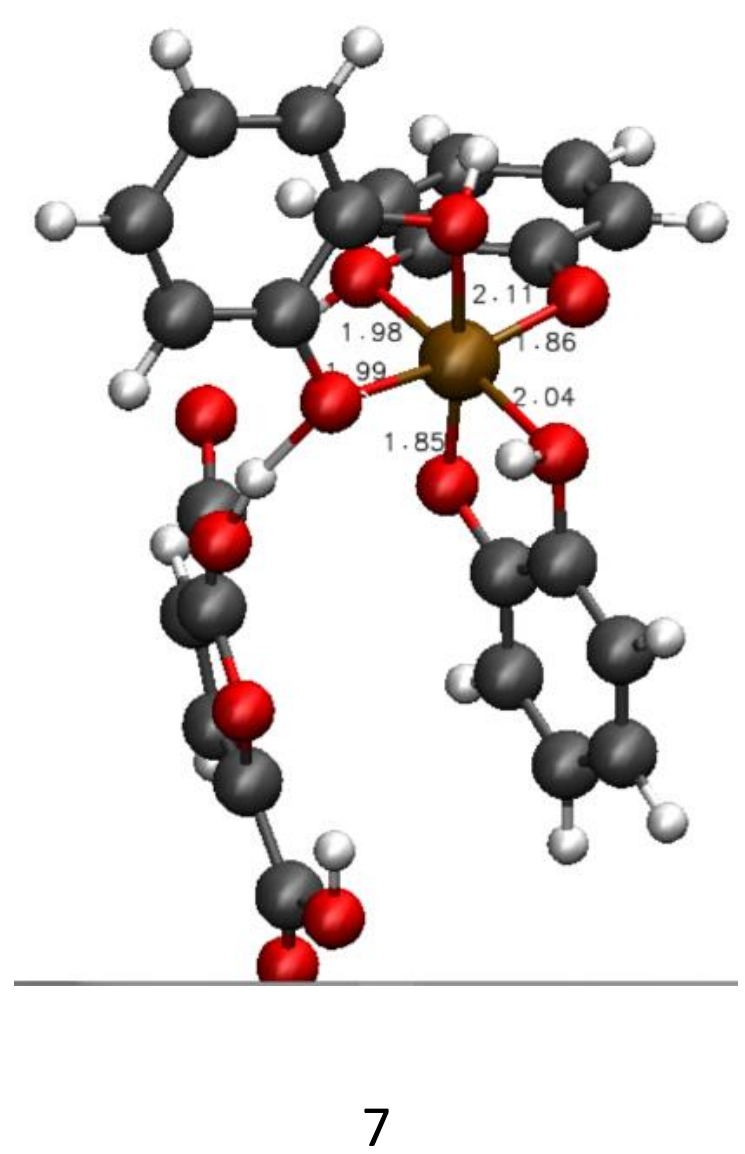

FDCA-0H+ BLYP-D3

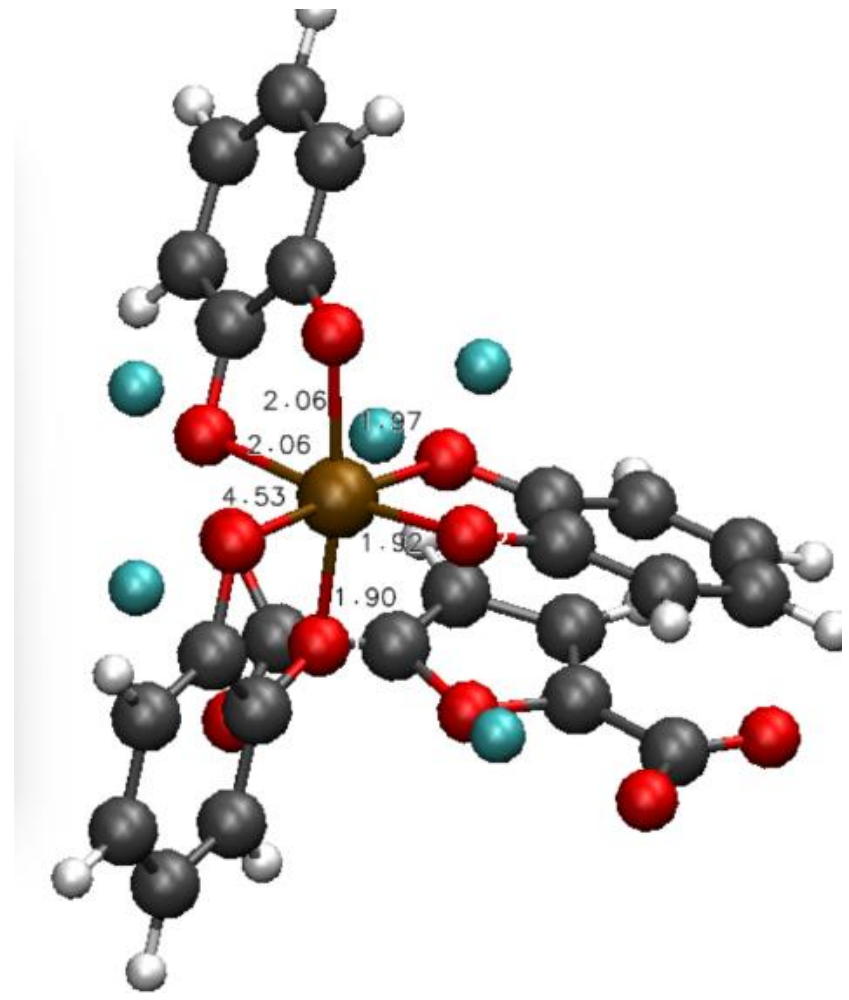

THFDCH-5H+ BLYP-D3

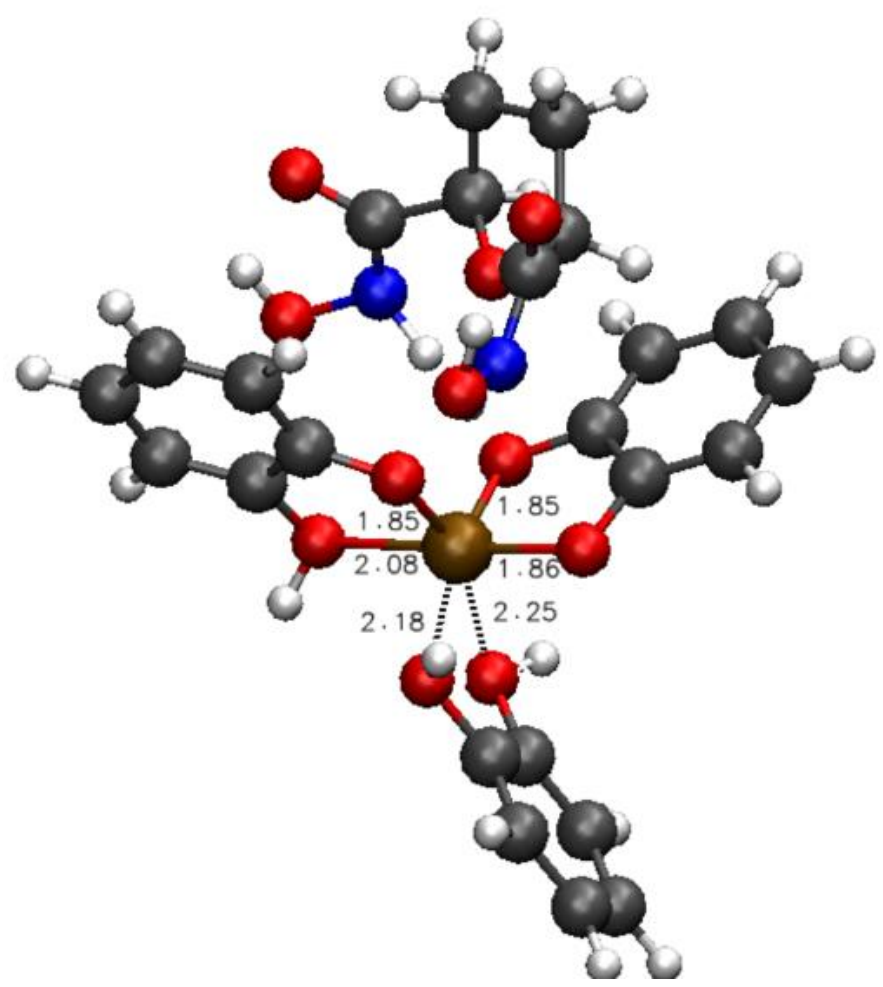

THFDCH-0H+ BLYP-D3

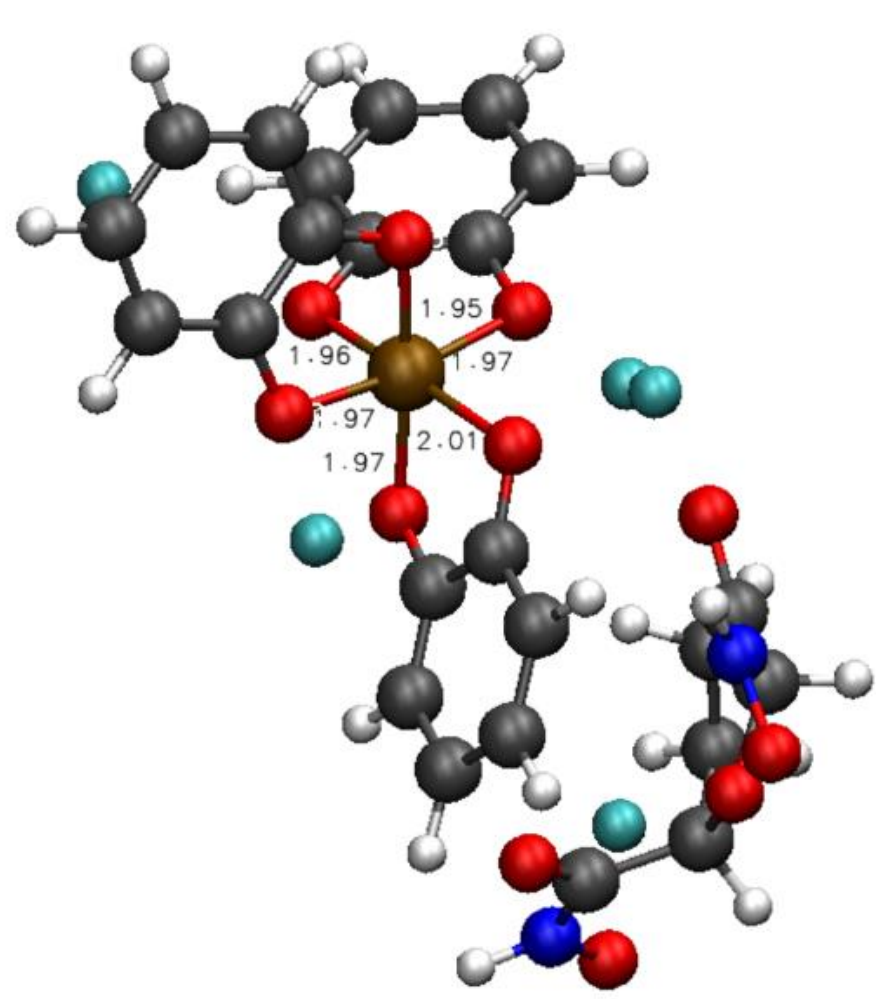

FDCH-5H+ BLYP-D3

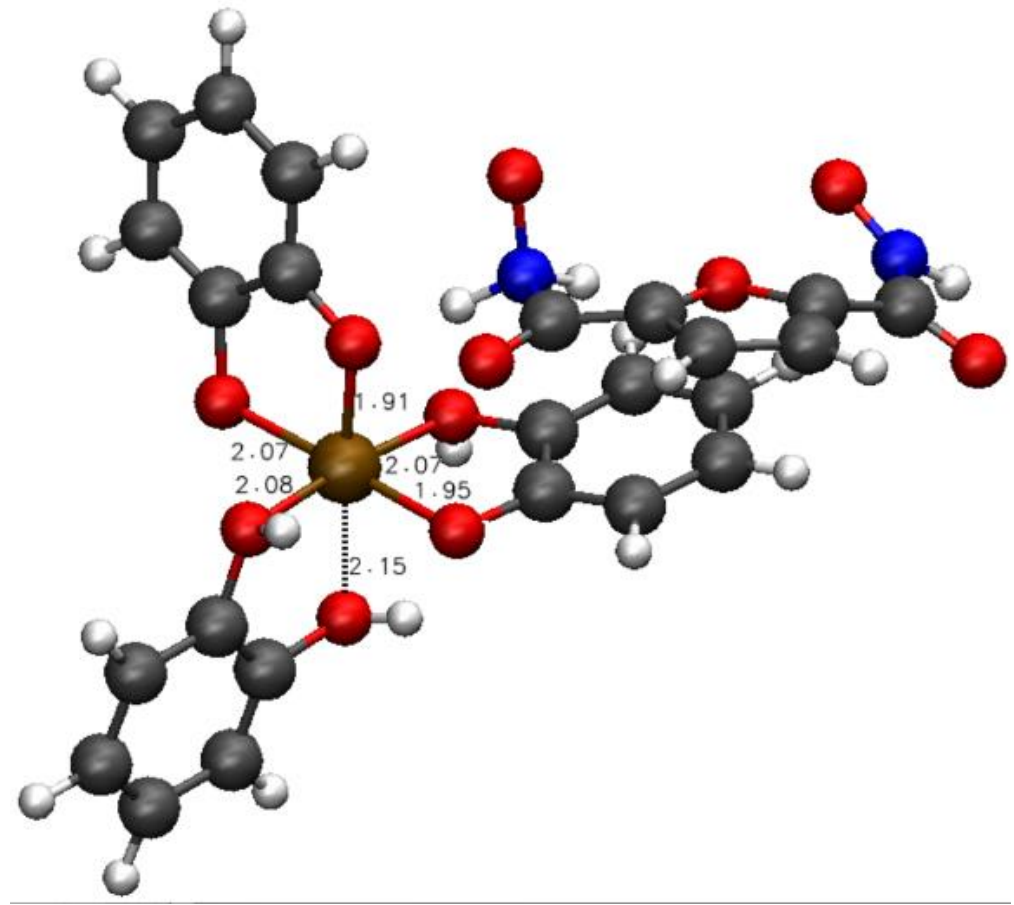

FDCH-0H+ BLYP-D3

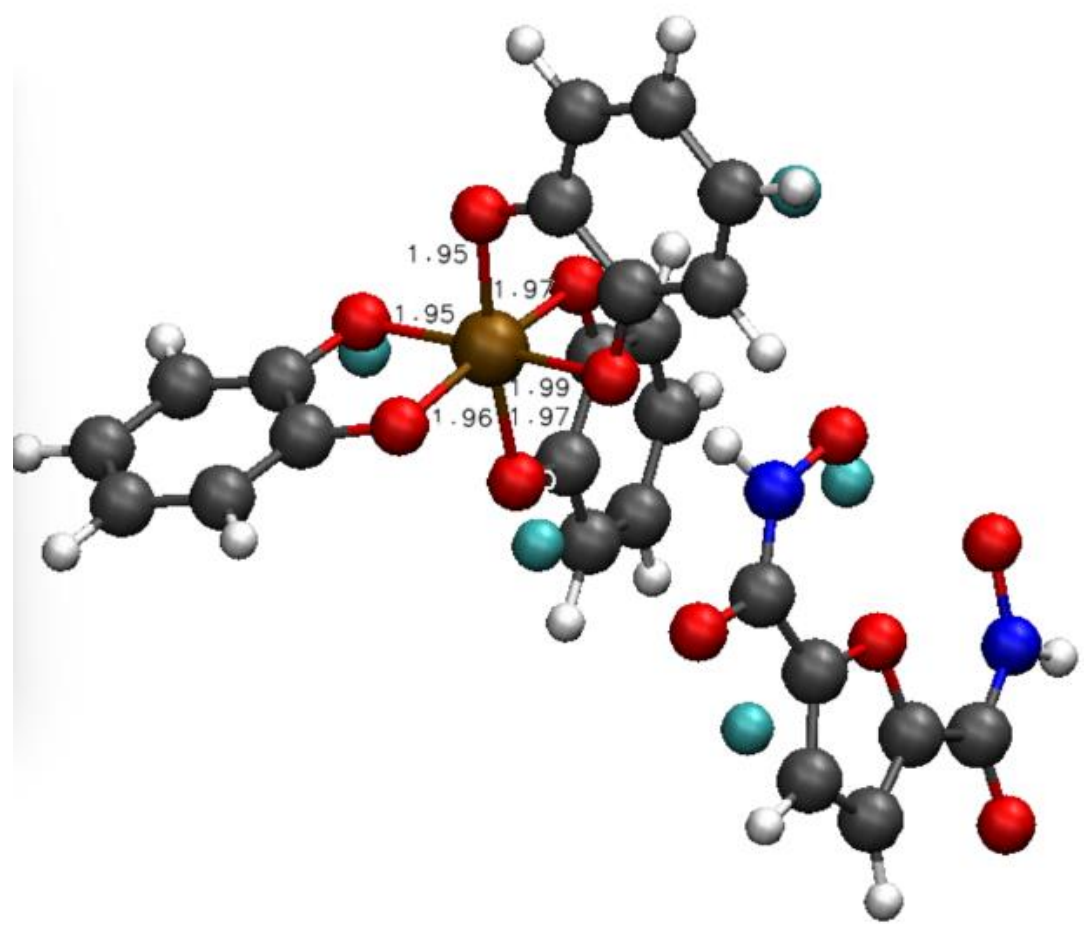

AHA-4H+ BLYP-D3

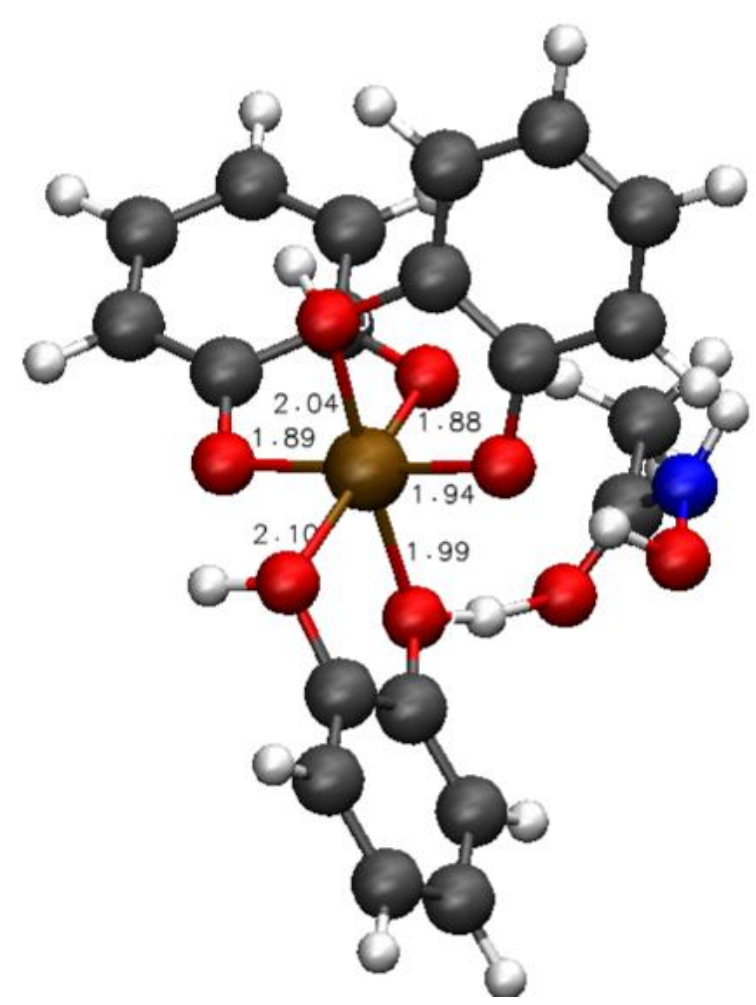

AHA-0H+ BLYP-D3

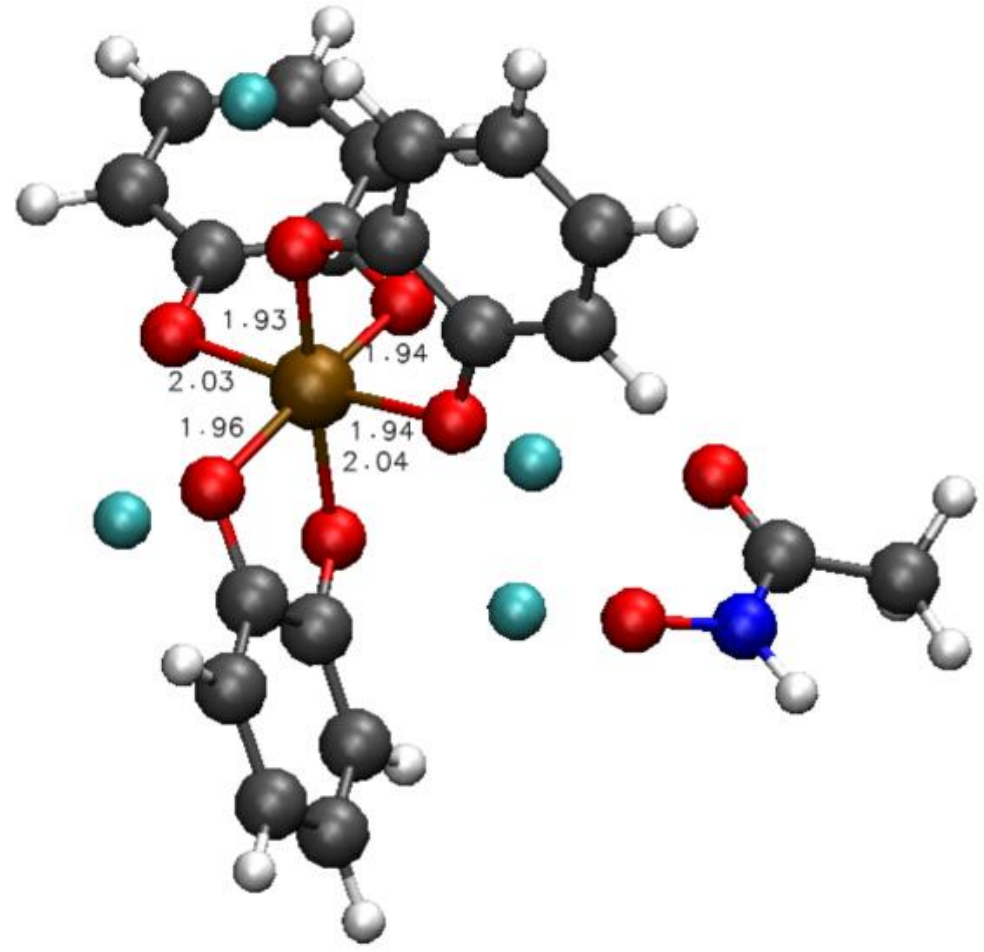

MAHA-4H+ BLYP-D3

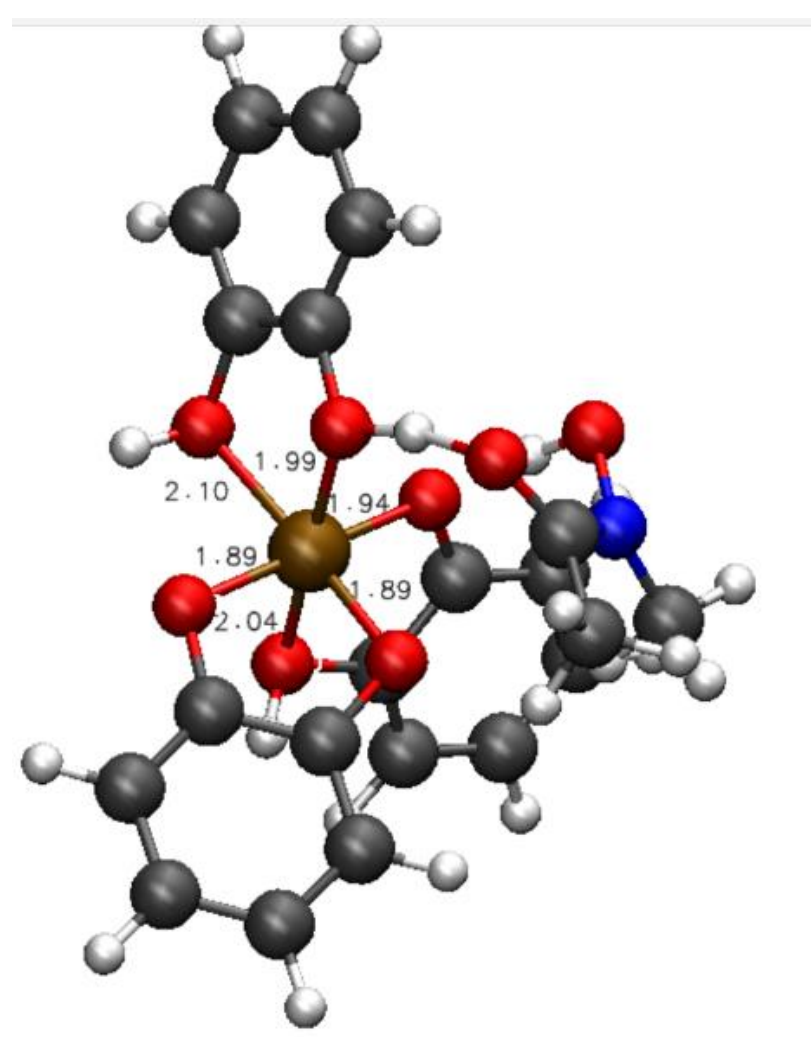

MAHA-0H+ BLYP-D3

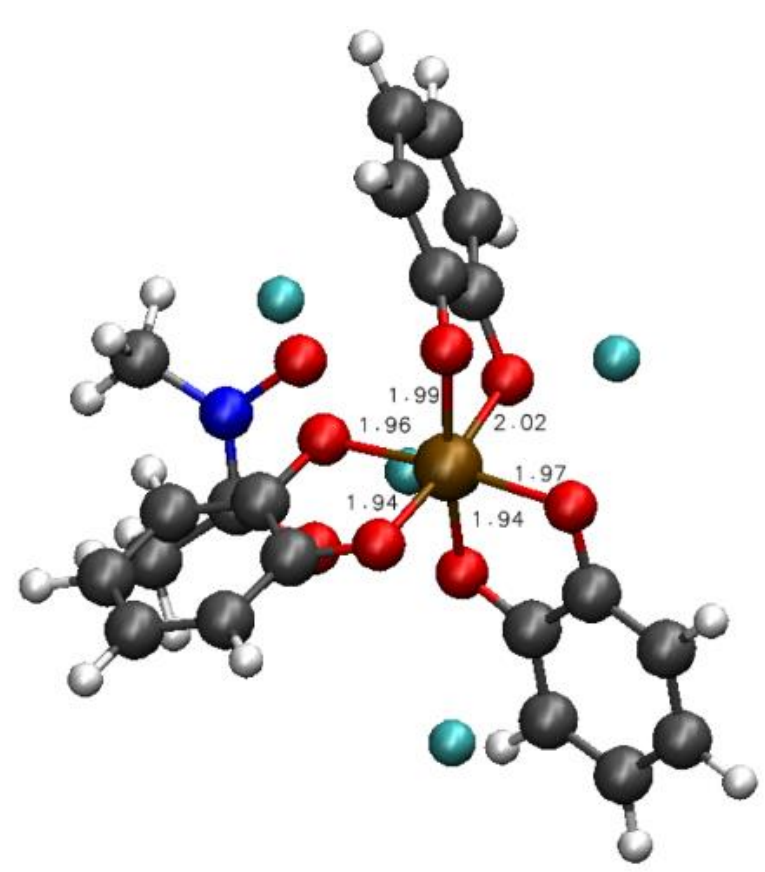

THFDCA-5H+ 12H2O BLYP-D3

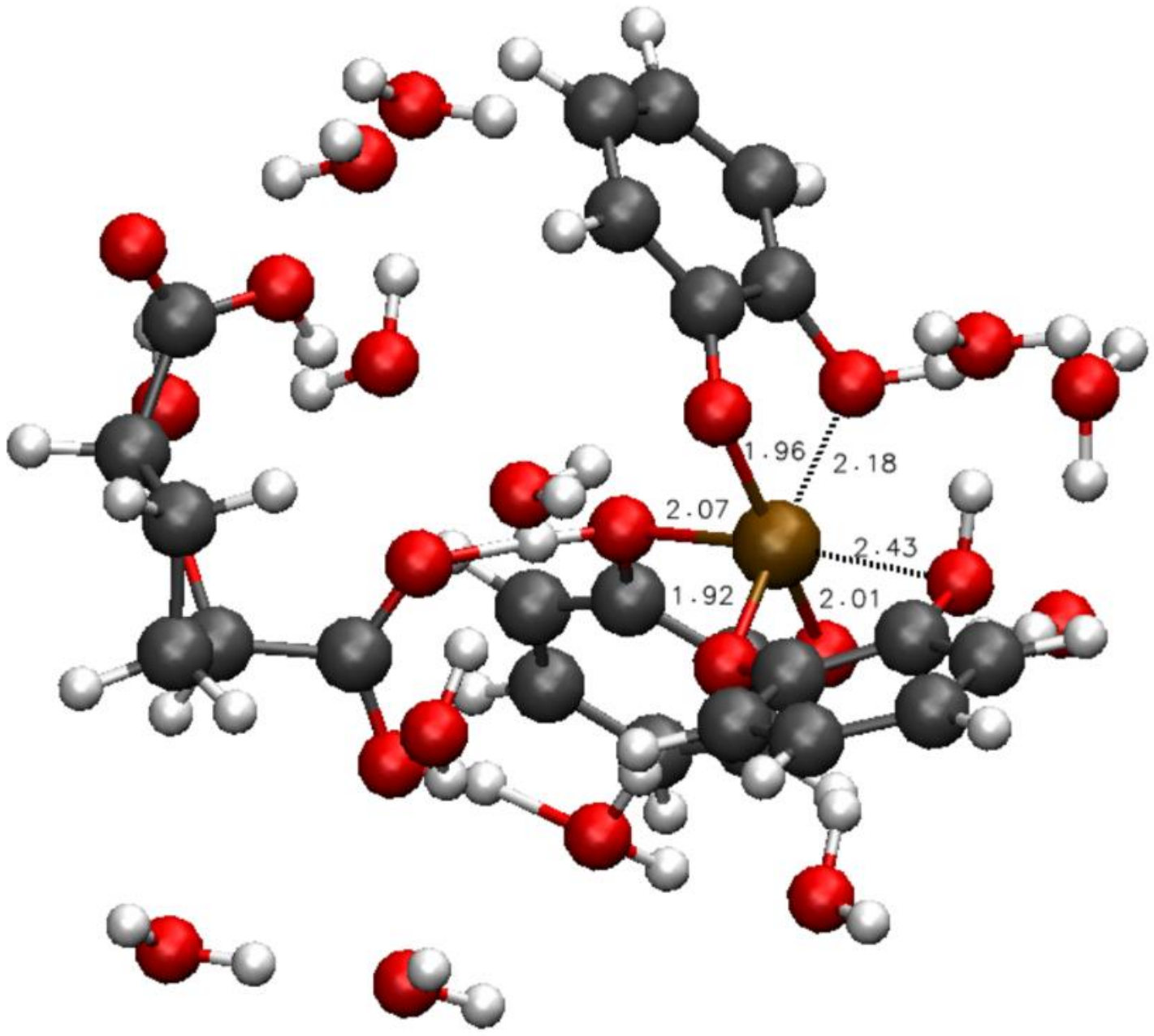

THFDCA-0H+ 12H2O BLYP-D3

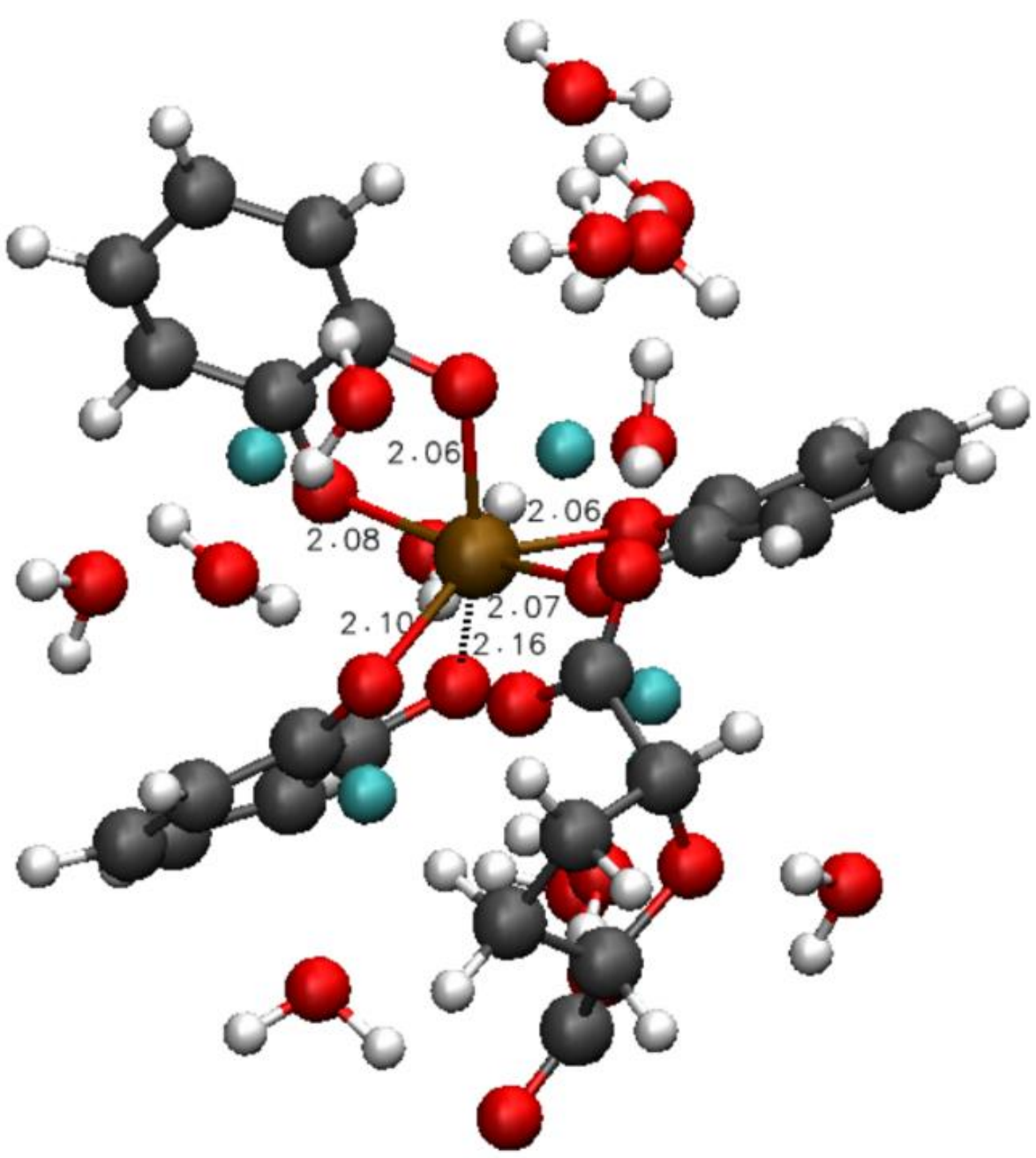

FDCA-5H+ 12H2O BLYP-D3

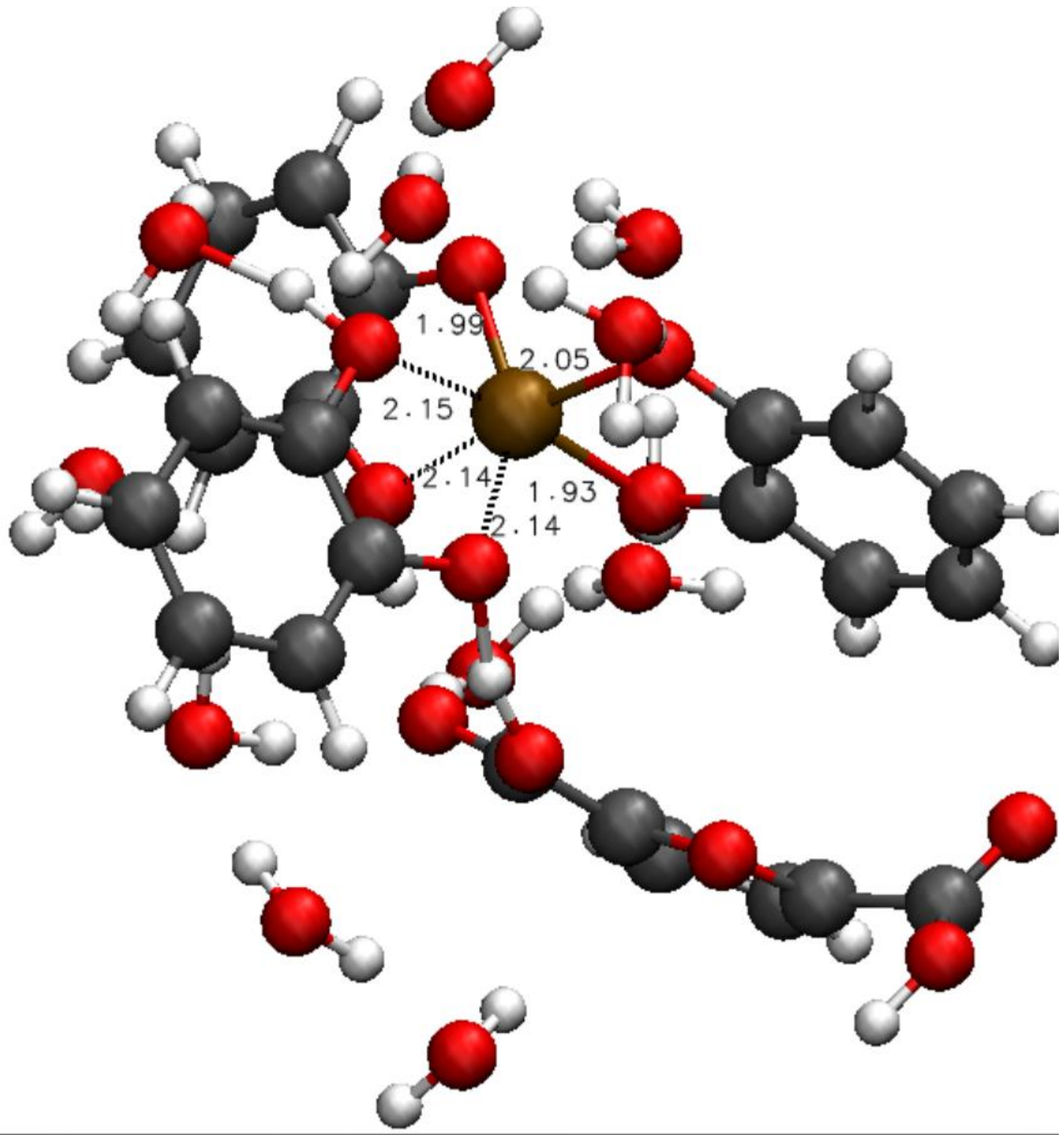

FDCA-0H+ 12H2O BLYP-D3

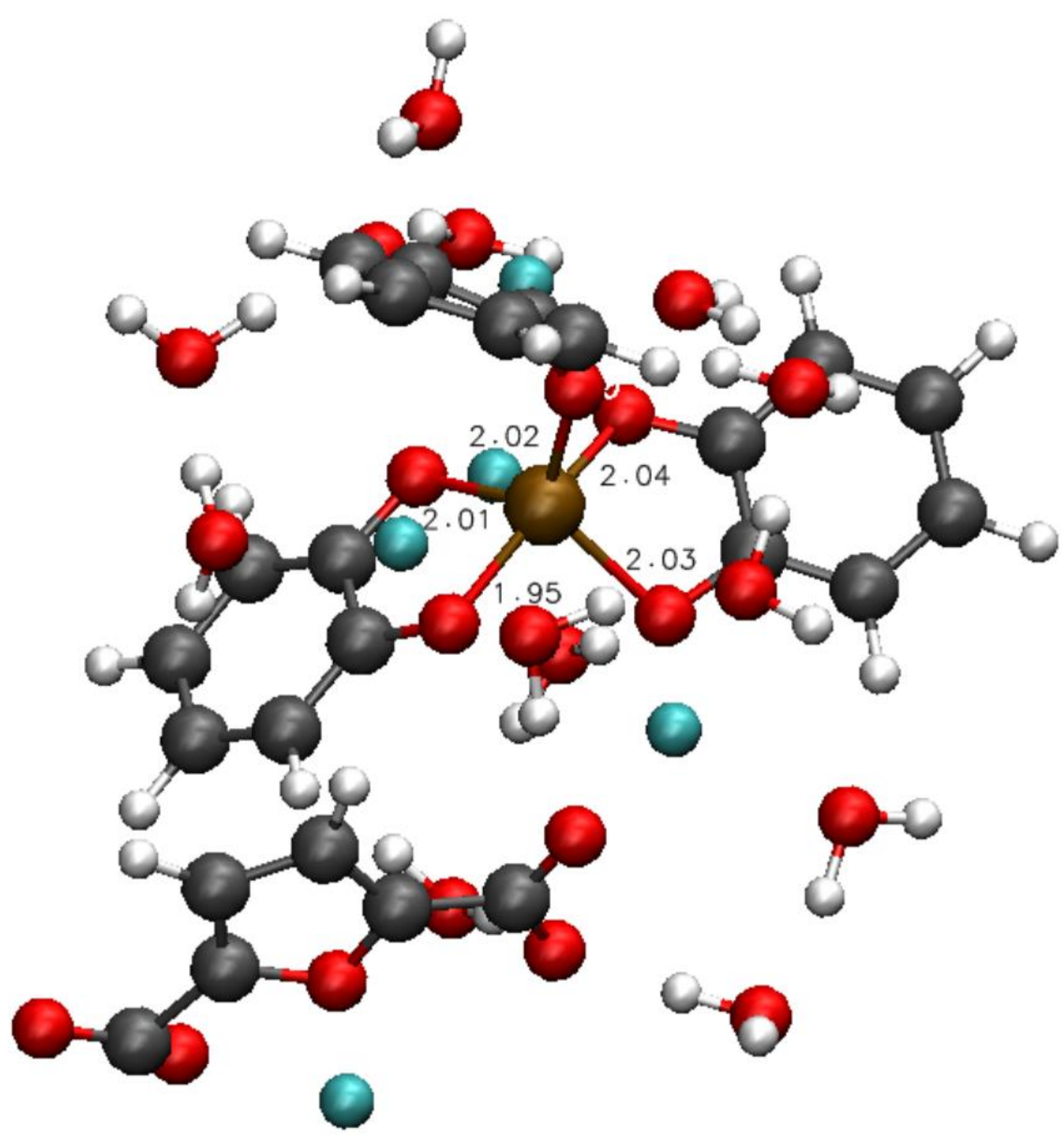

THFDCH-5H+ 12H2O BLYP-D3

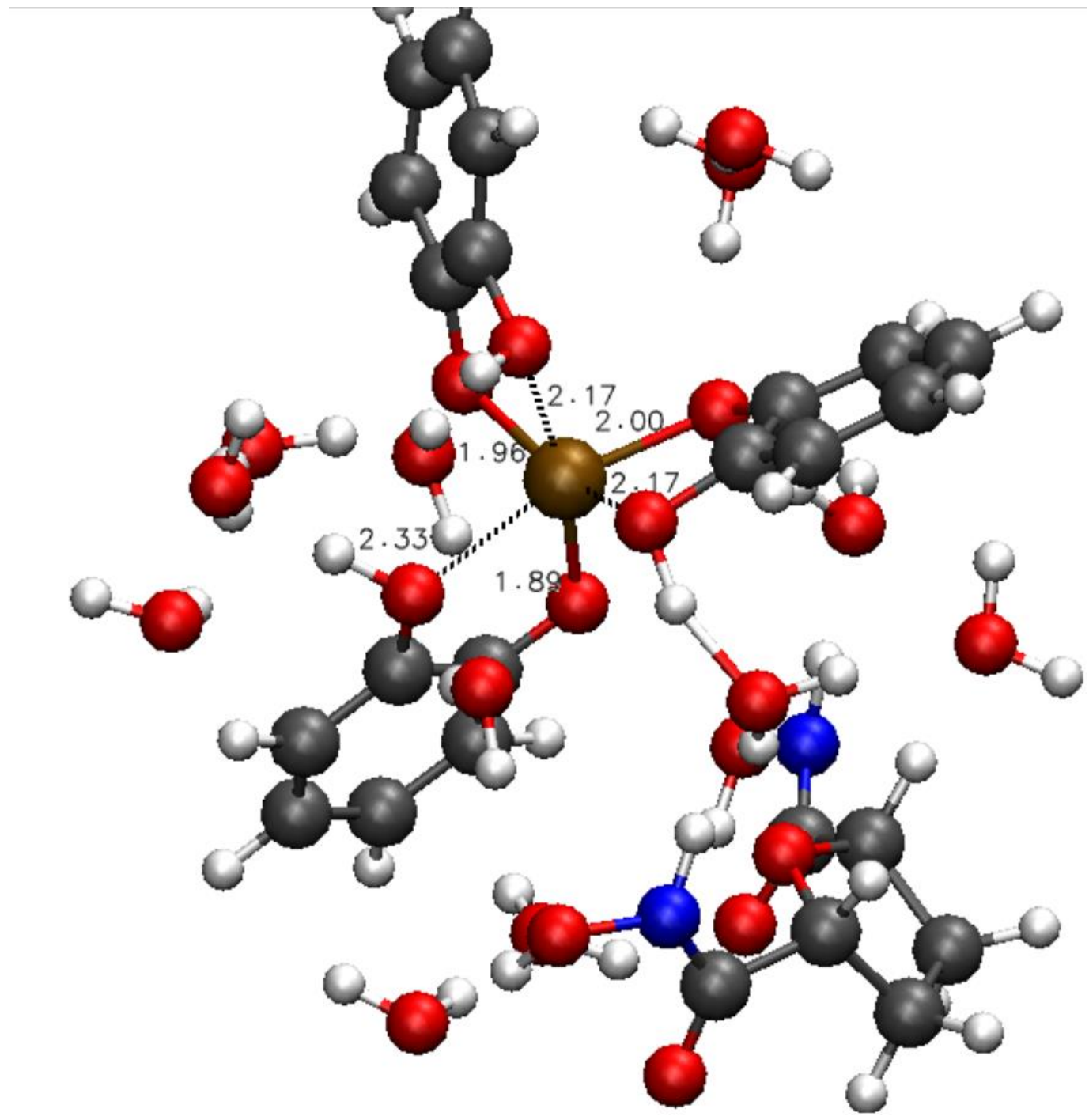

THFDCH-0H+ 12H2O BLYP-D3

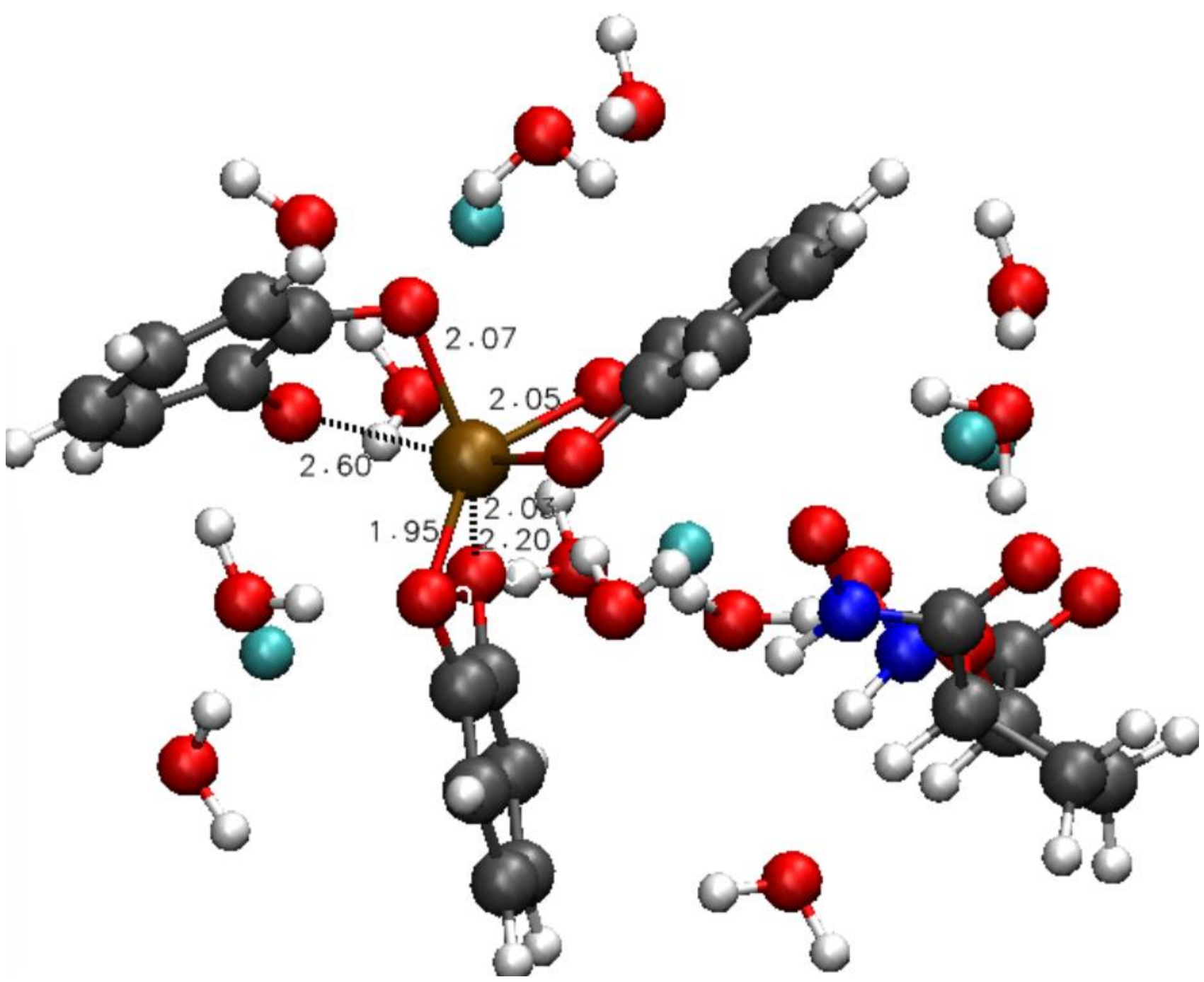

FDCH-5H+ 12H2O BLYP-D3

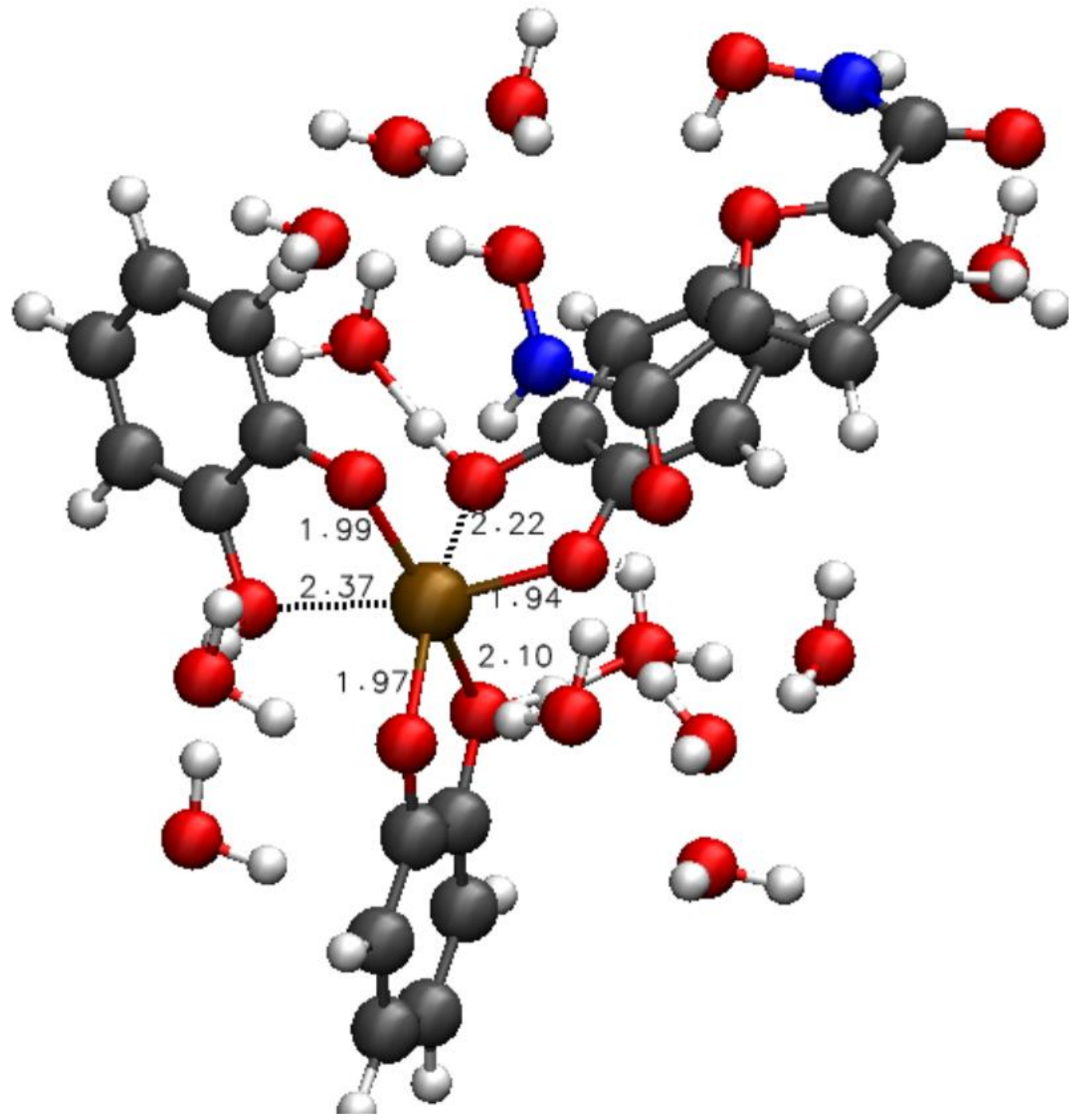

FDCH-0H+ 12H2O BLYP-D3

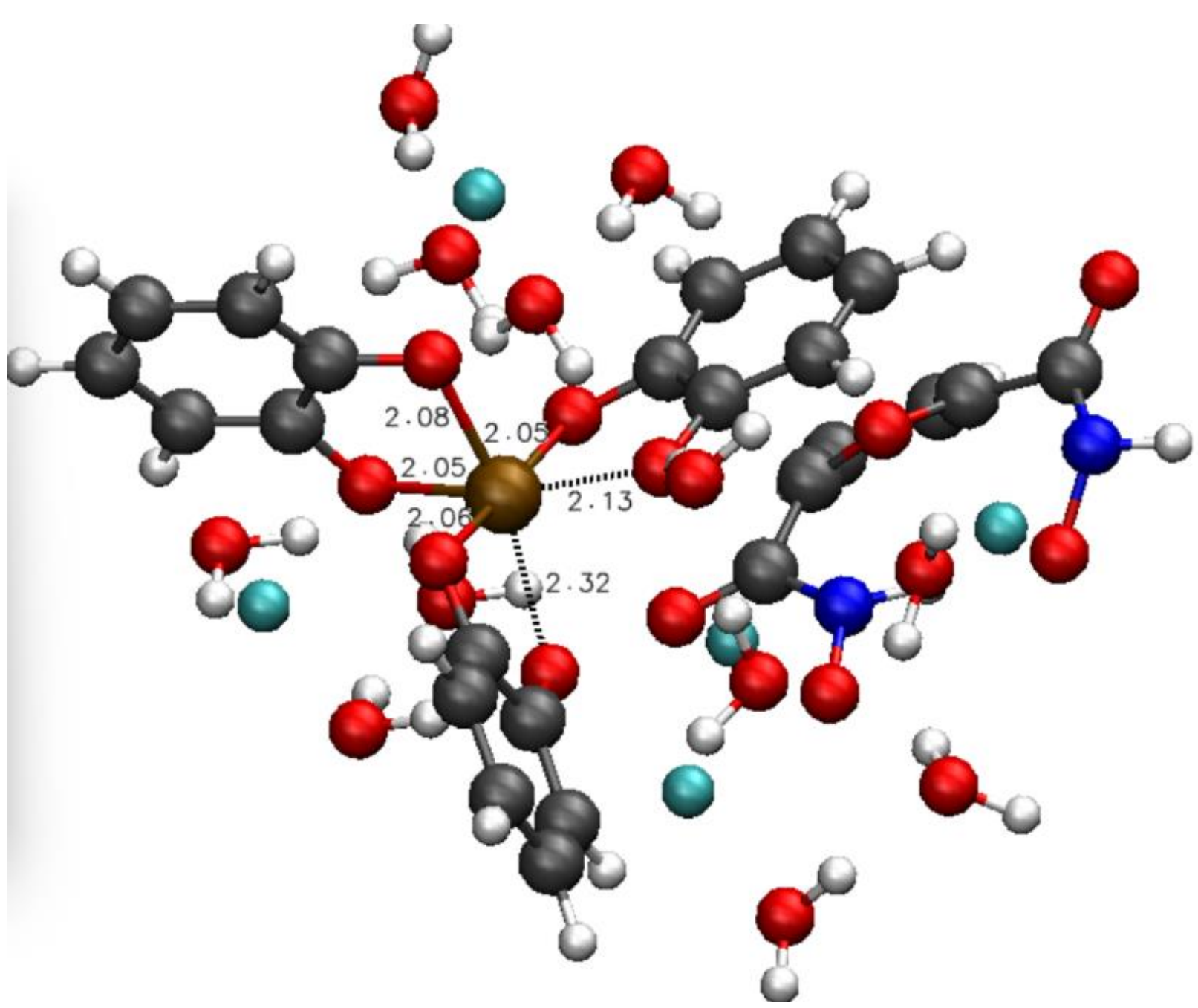

AHA-4H+ 12H2O BLYP-D3

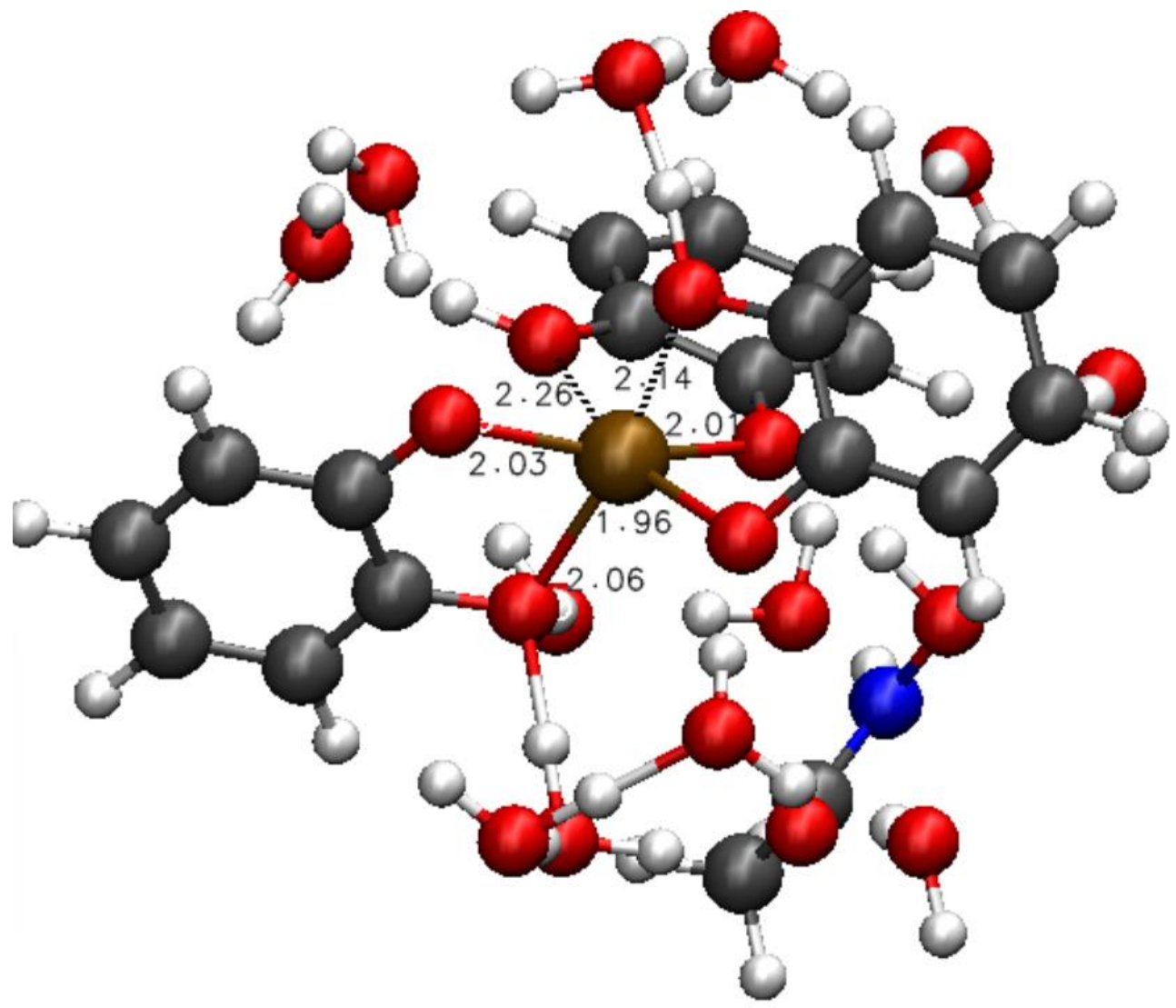

AHA-0H+ 12H2O BLYP-D3

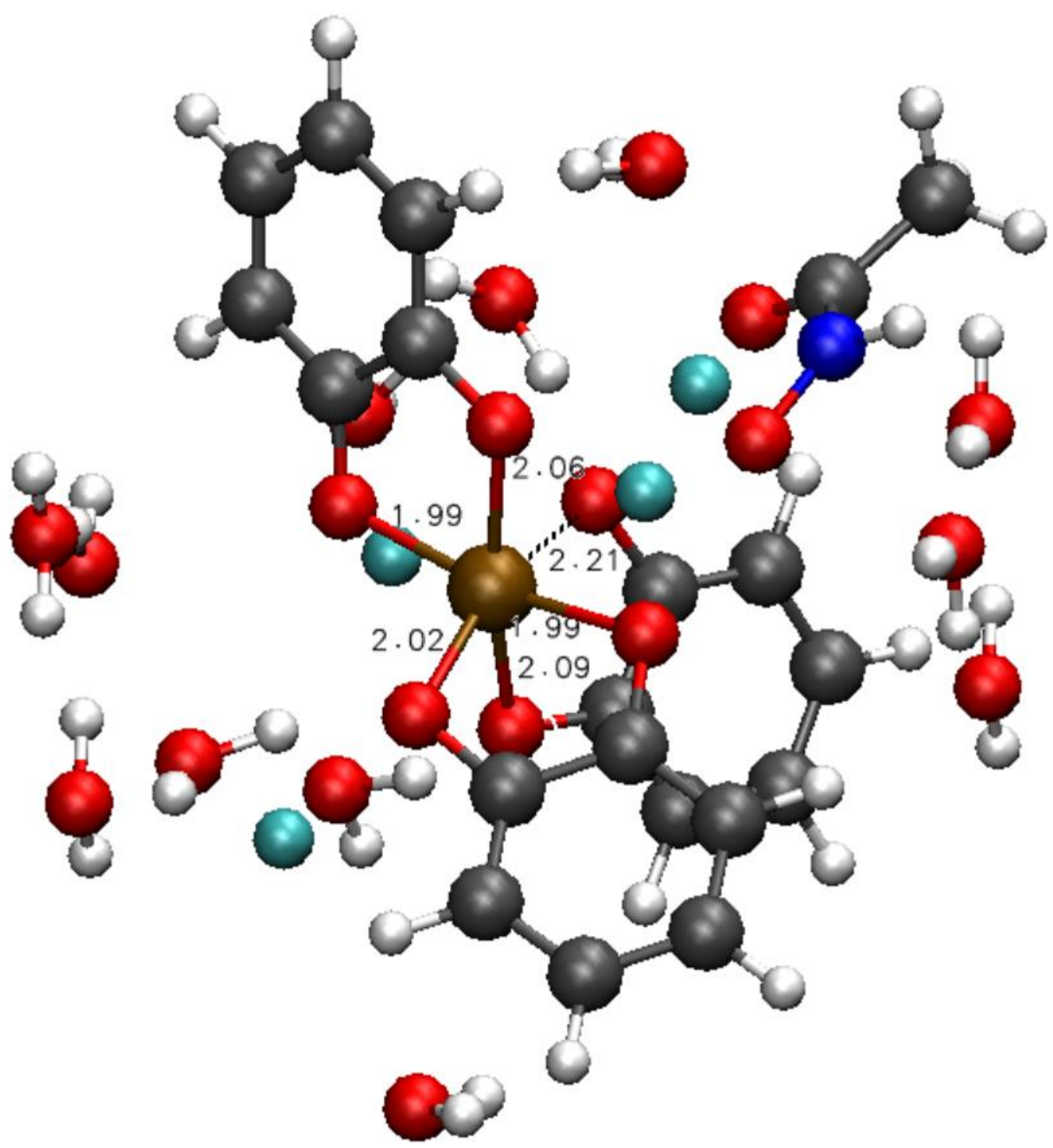

MAHA-4H+ 12H2O BLYP-D3

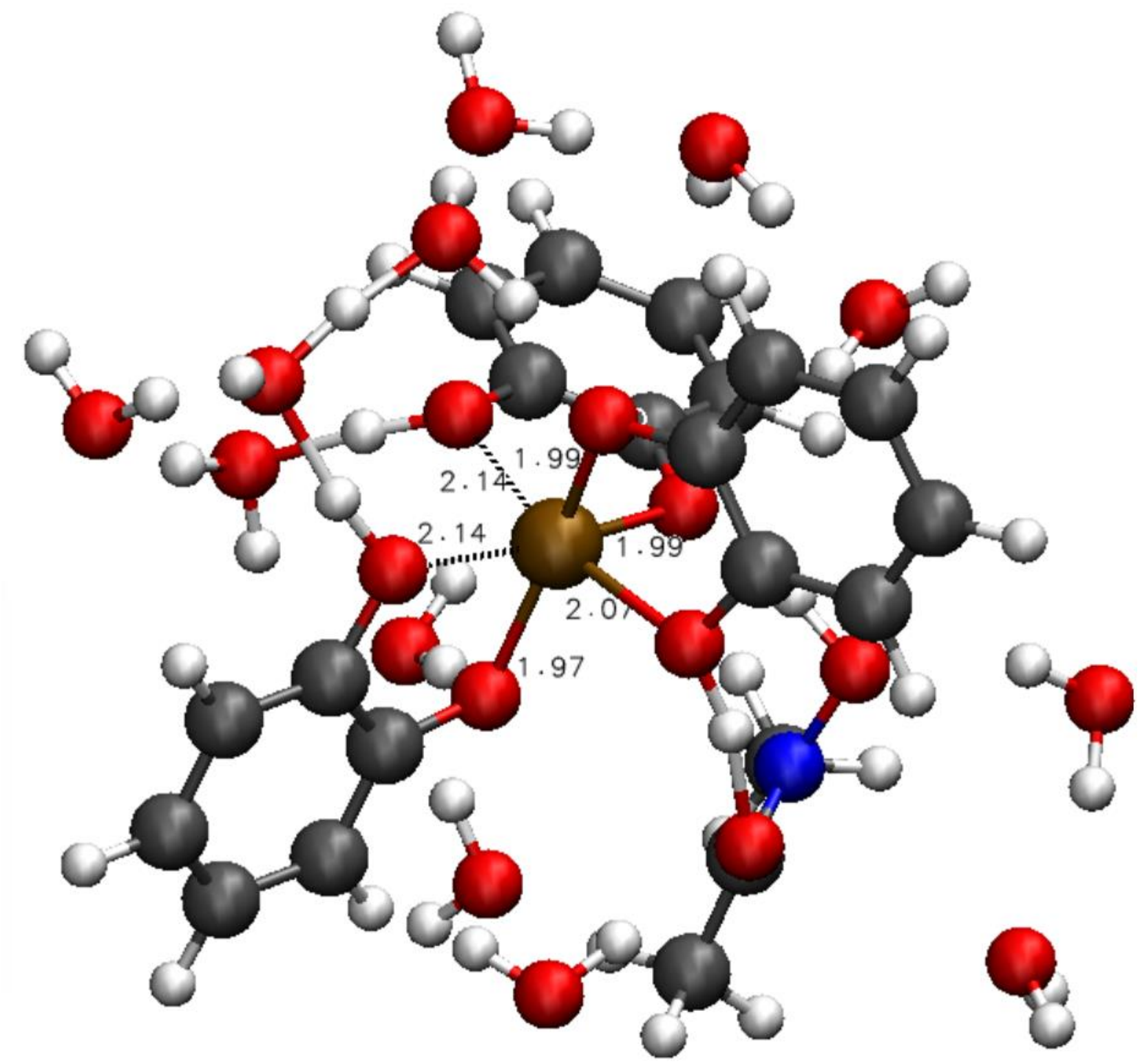

MAHA-0H+ 12H2O BLYP-D3

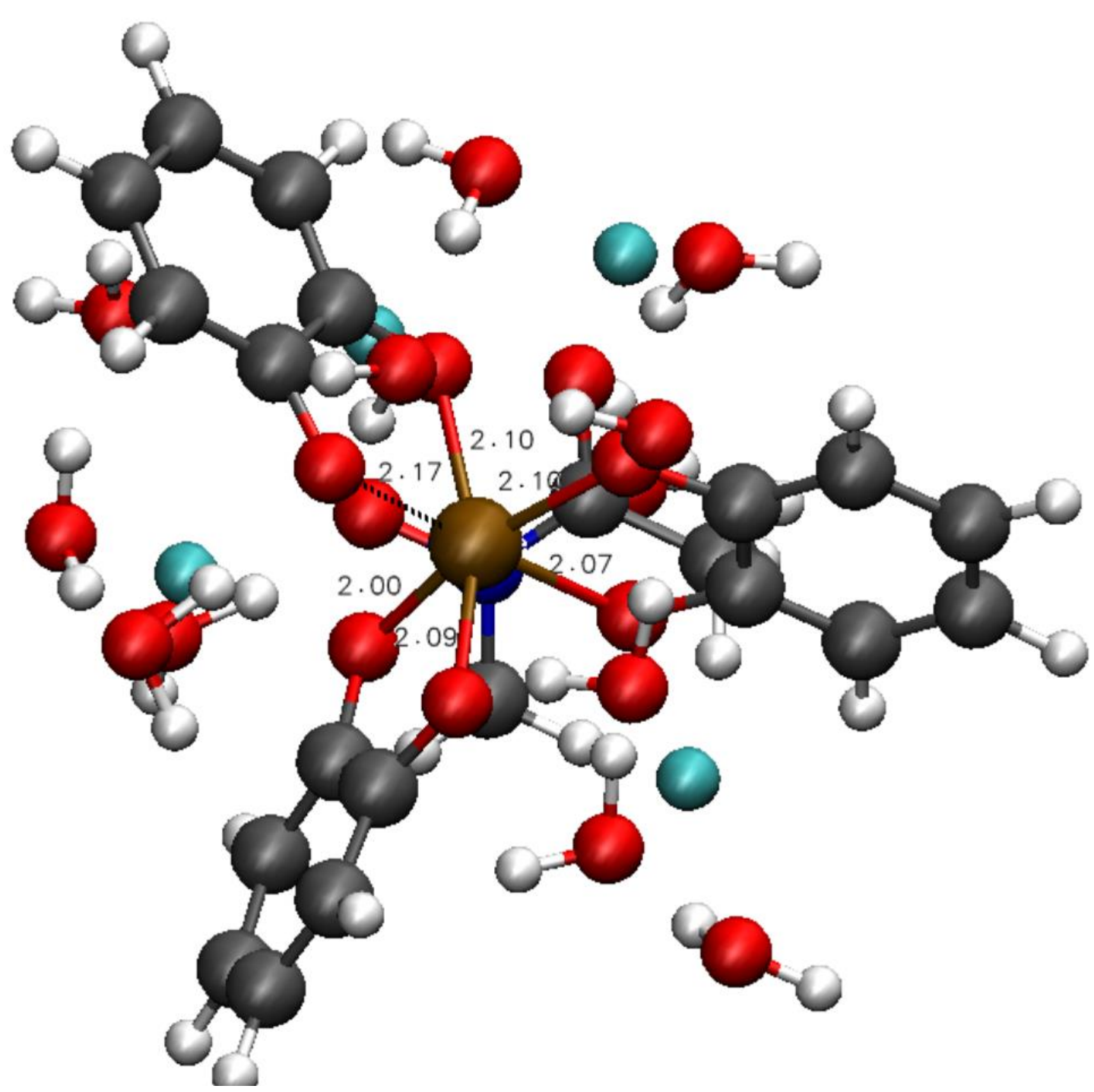

Figure S1. The gradual buildup of the metadynamics free energy profiles projected along the collective variable (relevant distance) in every FES showed in **the main text** (PBE-D3, DZVP-MOLOPT).

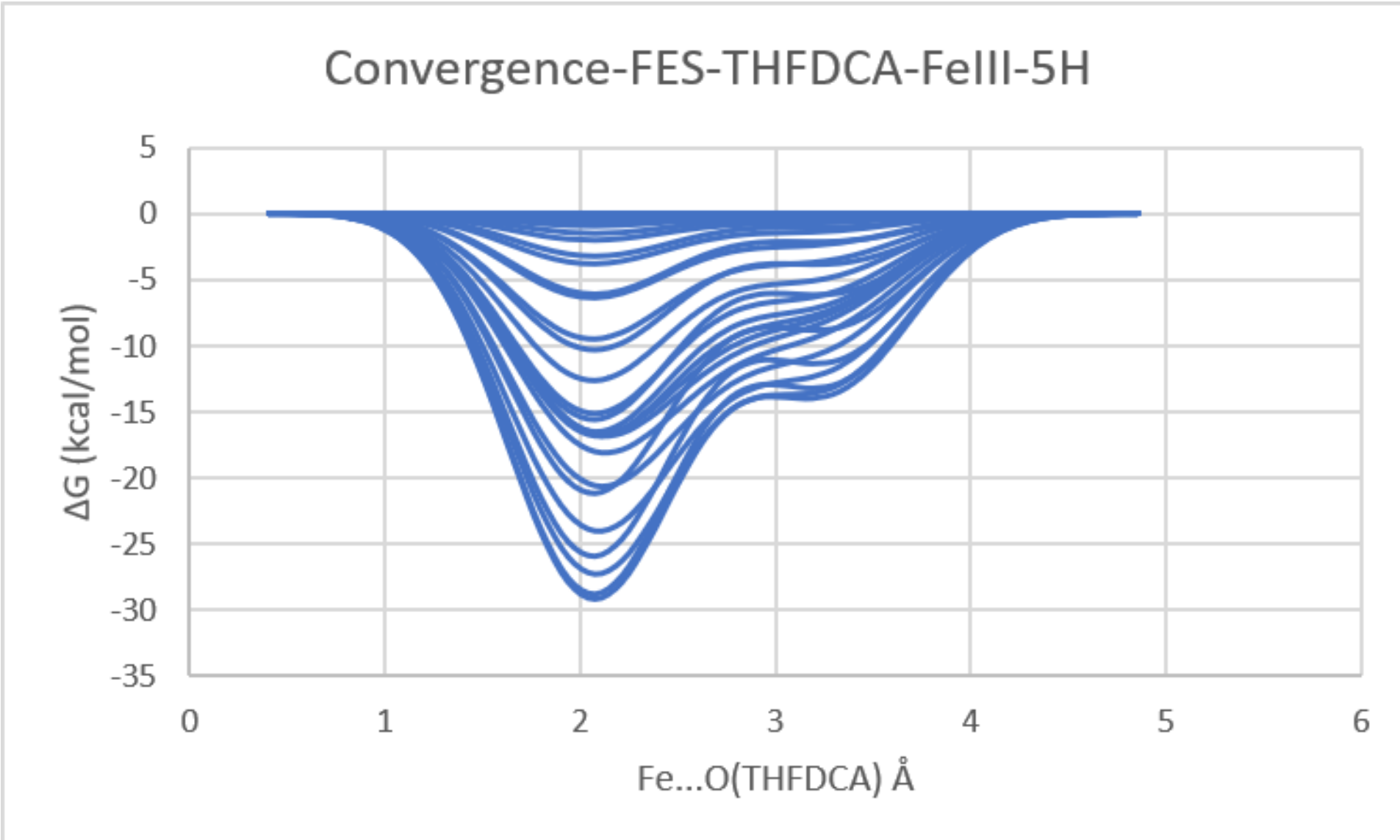

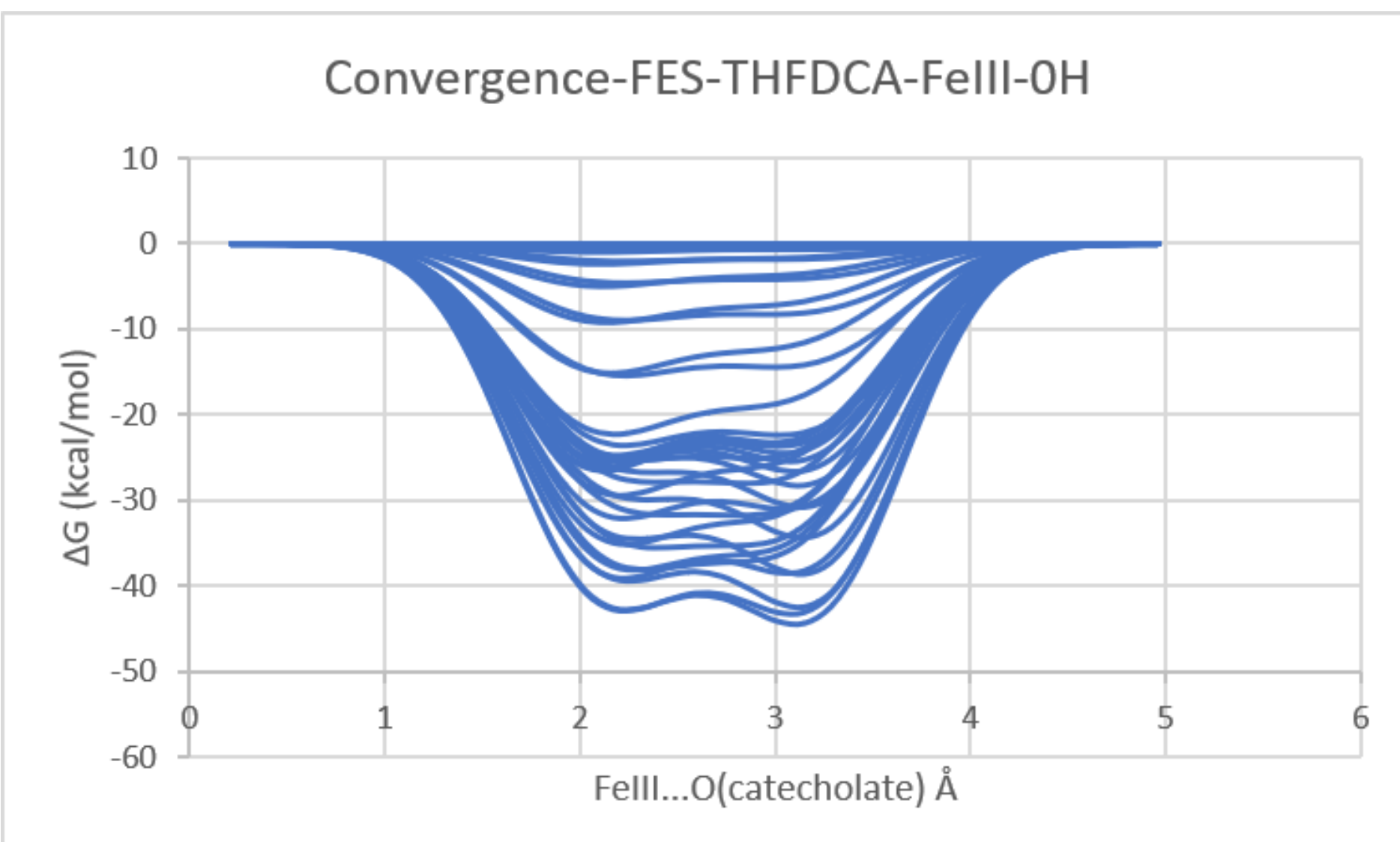

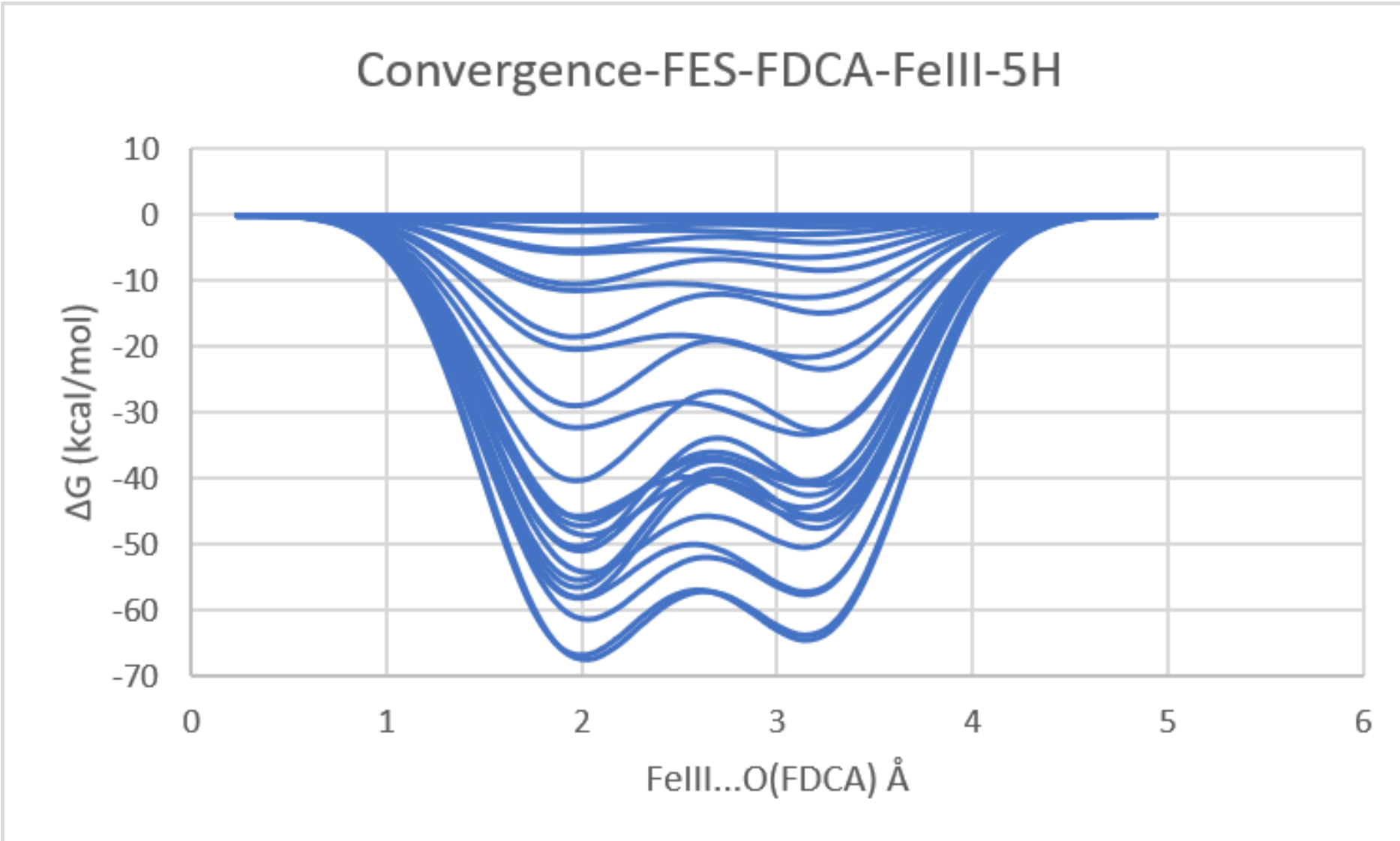
Convergence-FES-FDCA-FeIII-5H
ΔG (kcal/mol)
10
0
-10
-20
-30
-40
-50
-60
-70
0
1
2
3
4
5
6
FeIII...O(FDCA) Å

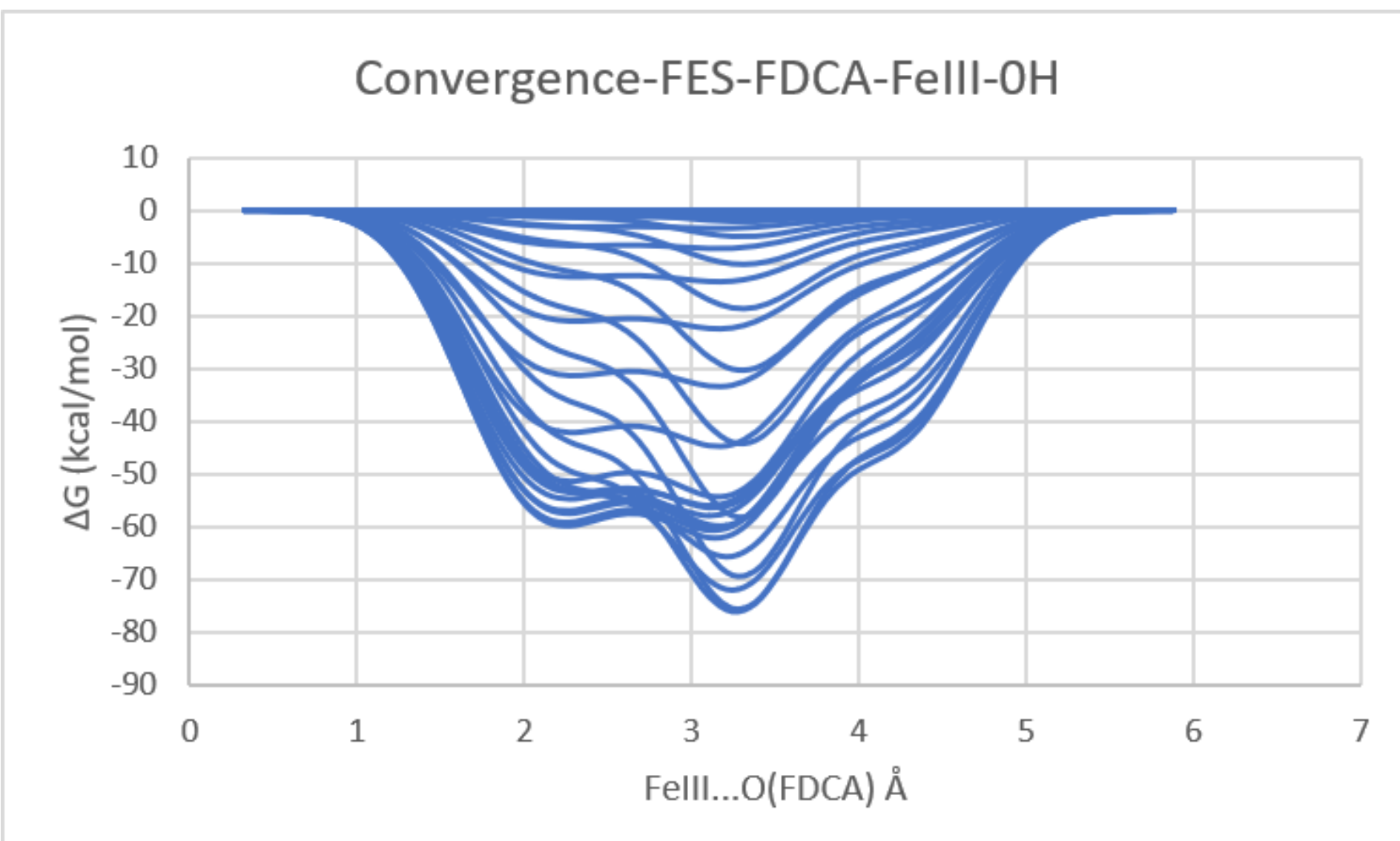
Convergence-FES-FDCA-FeIII-0H
ΔG (kcal/mol)
10
0
-10
-20
-30
-40
-50
-60
-70
-80
-90
0
1
2
3
4
5
6
7
FeIII...O(FDCA) Å

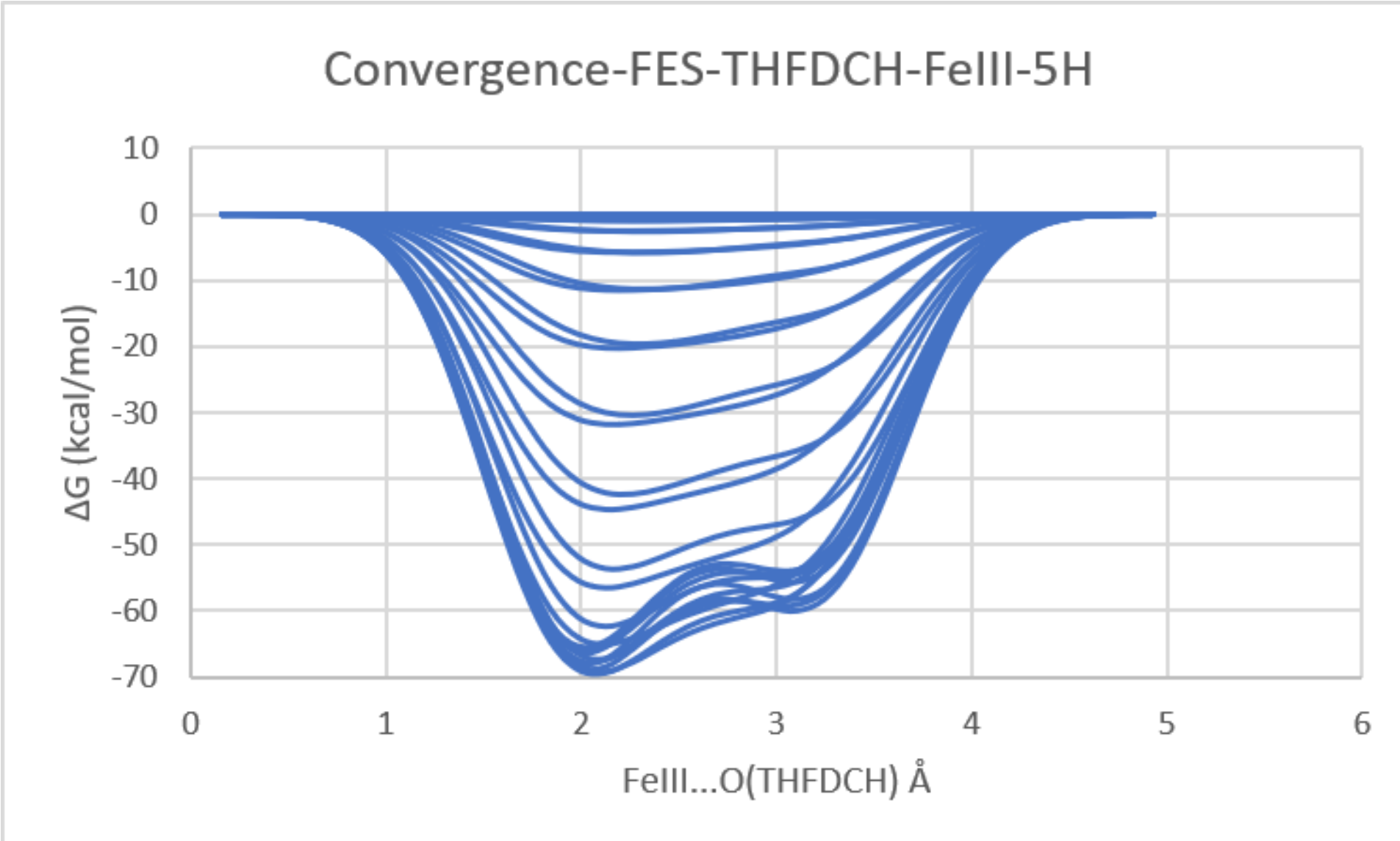
Convergence-FES-THFDCH-FeIII-5H
ΔG (kcal/mol)
10
0
-10
-20
-30
-40
-50
-60
-70
0
1
2
3
4
5
6
FeIII...O(THFDCH) Å

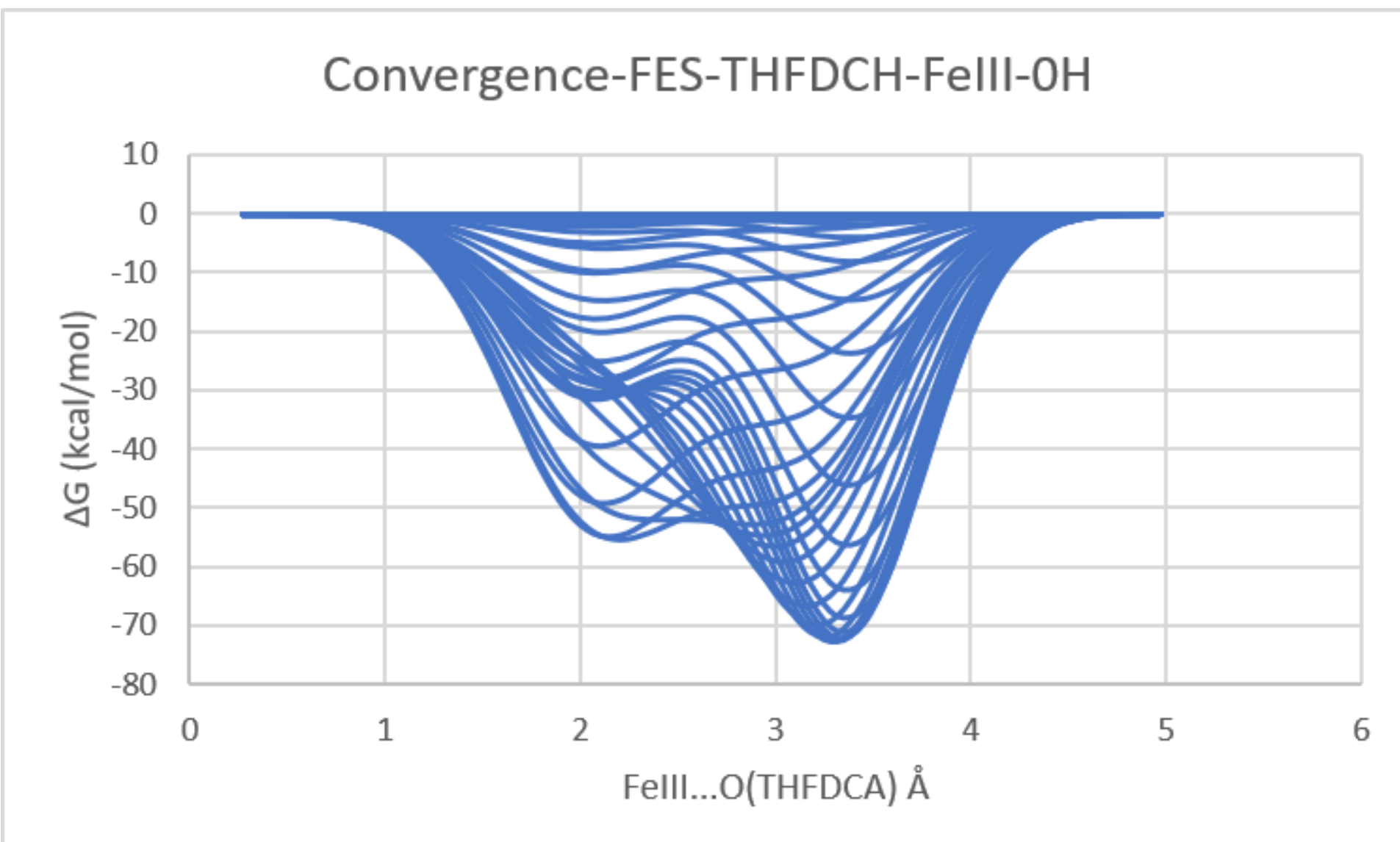
Convergence-FES-THFDCH-FeIII-0H
ΔG (kcal/mol)
10
0
-10
-20
-30
-40
-50
-60
-70
-80
0
1
2
3
4
5
6
FeIII...O(THFDCA) Å

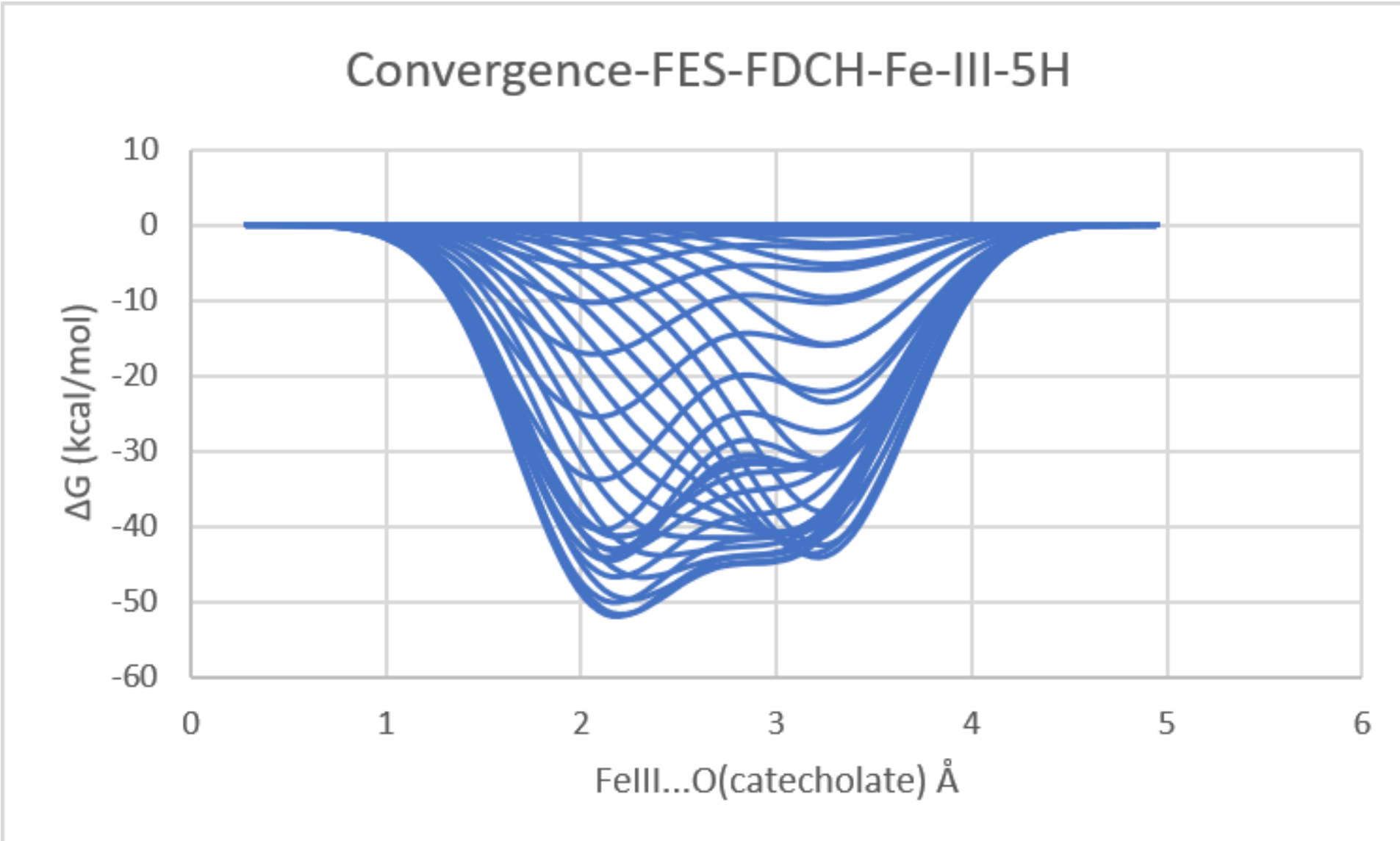
Convergence-FES-FDCH-Fe-III-5H
ΔG (kcal/mol)
10
0
-10
-20
-30
-40
-50
-60
0
1
2
3
4
5
6
FeIII...O(catecholate) Å

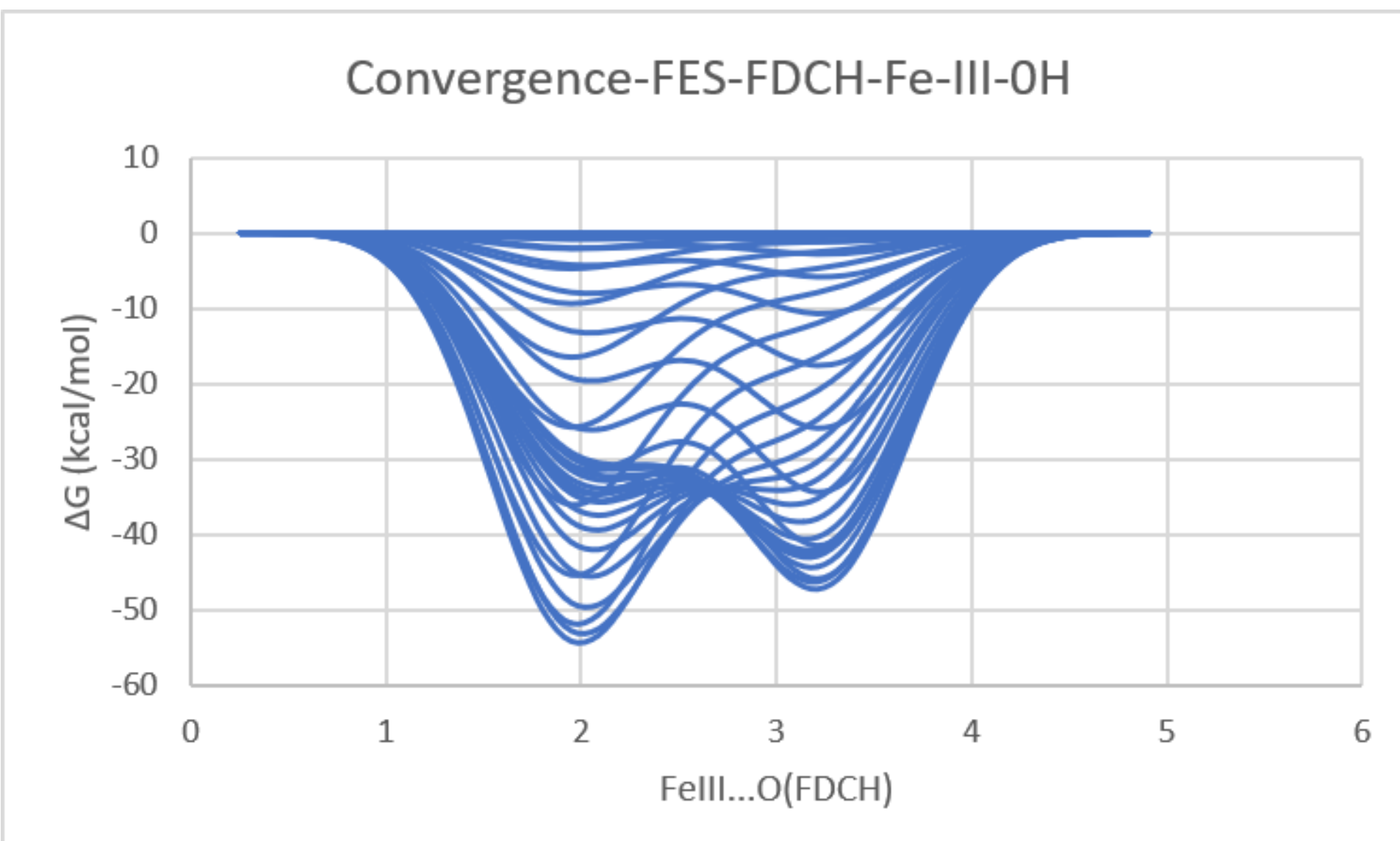
Convergence-FES-FDCH-Fe-III-0H
ΔG (kcal/mol)
10
0
-10
-20
-30
-40
-50
-60
0
1
2
3
4
5
6
FeIII...O(FDCH)

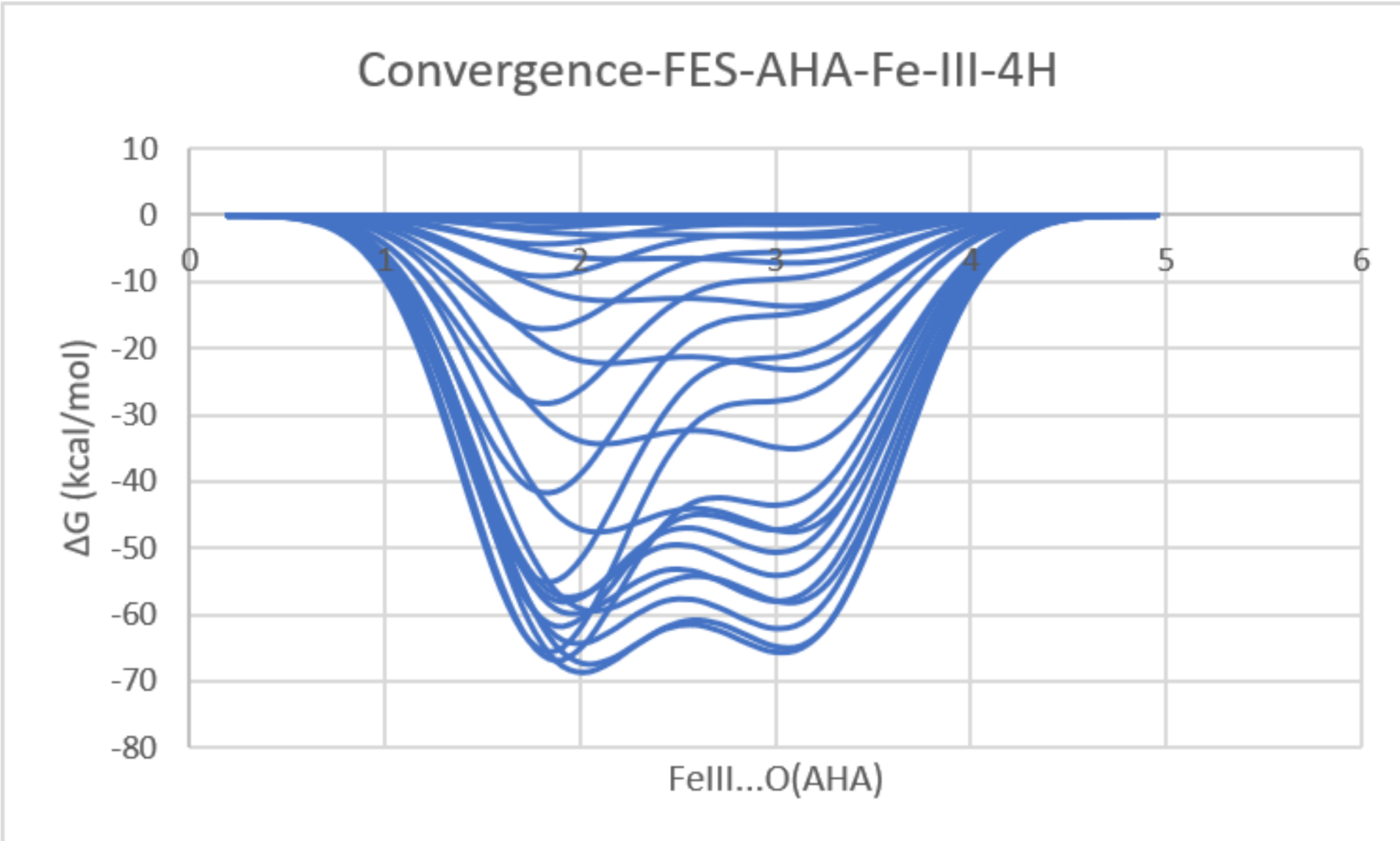

Convergence-FES-AHA-Fe-III-4H
10
0
-10
-20
-30
-40
-50
-60
-70
-80
0
1
2
3
4
5
6
ΔG (kcal/mol)
FeIII...O(AHA)

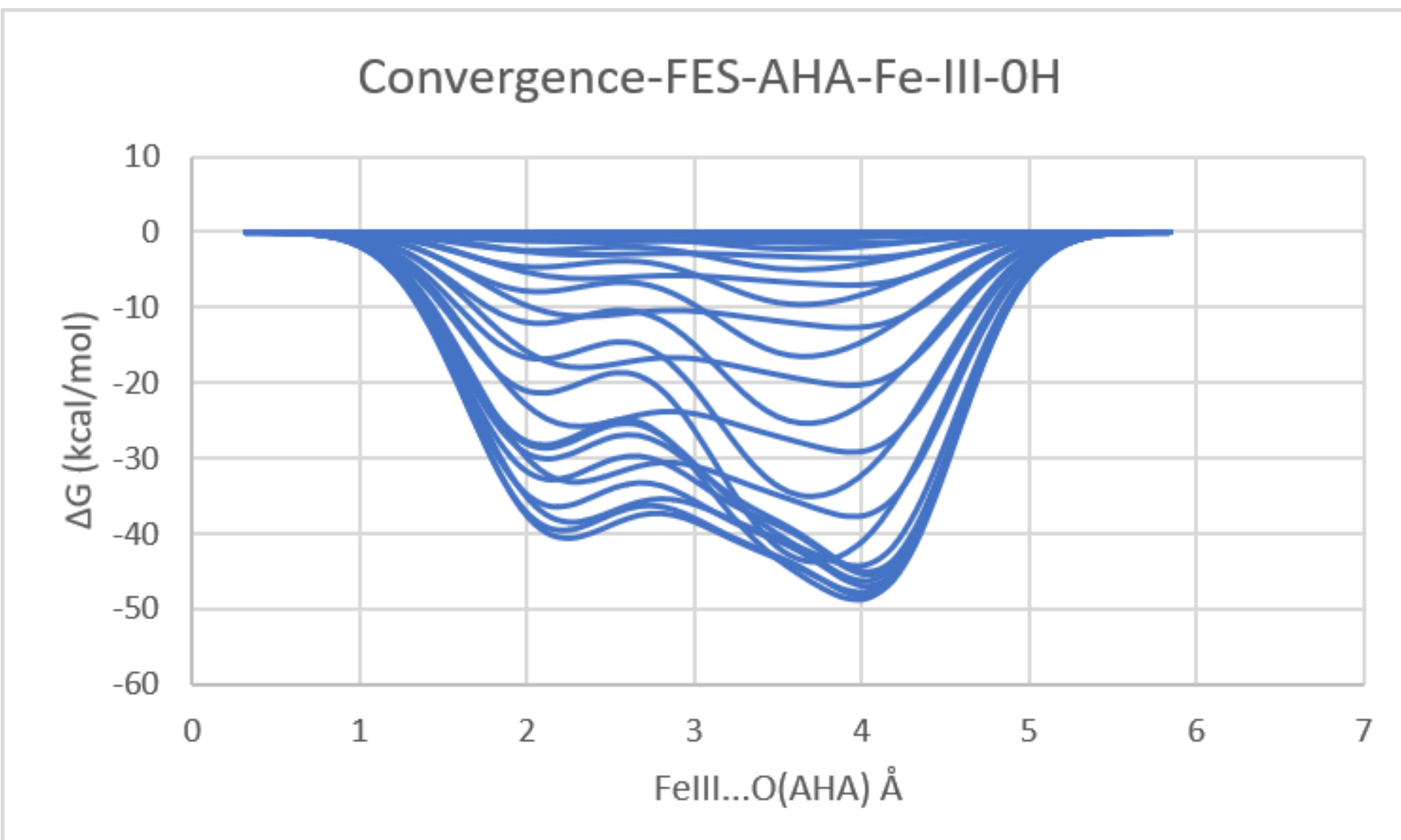

Convergence-FES-AHA-Fe-III-0H
10
0
-10
-20
-30
-40
-50
-60
0
1
2
3
4
5
6
7
ΔG (kcal/mol)
FeIII...O(AHA) Å

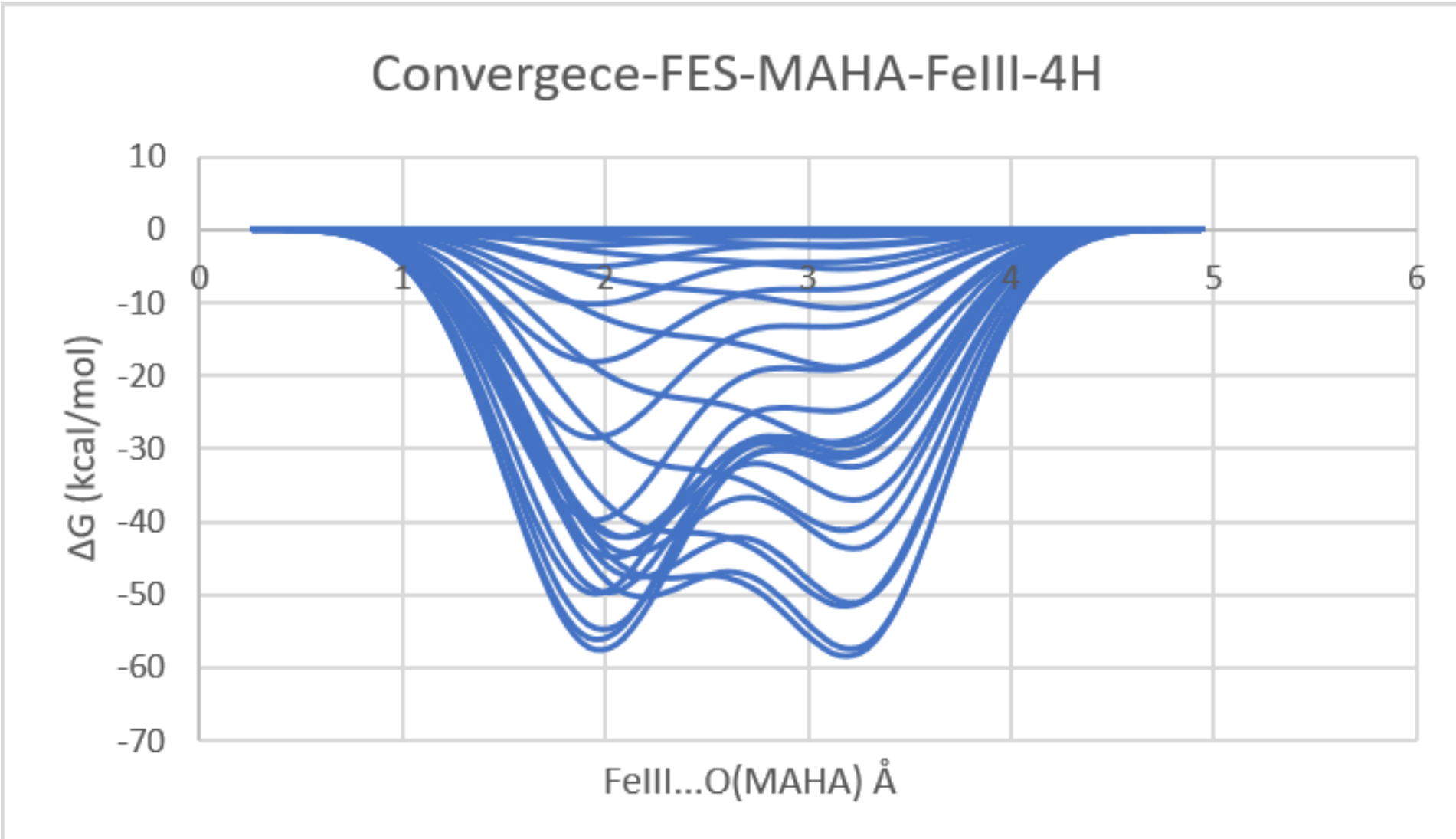
Convergece-FES-MAHA-FeIII-4H
10
0
-10
-20
-30
-40
-50
-60
-70
0
1
2
3
4
5
6
ΔG (kcal/mol)
FeIII...O(MAHA) Å

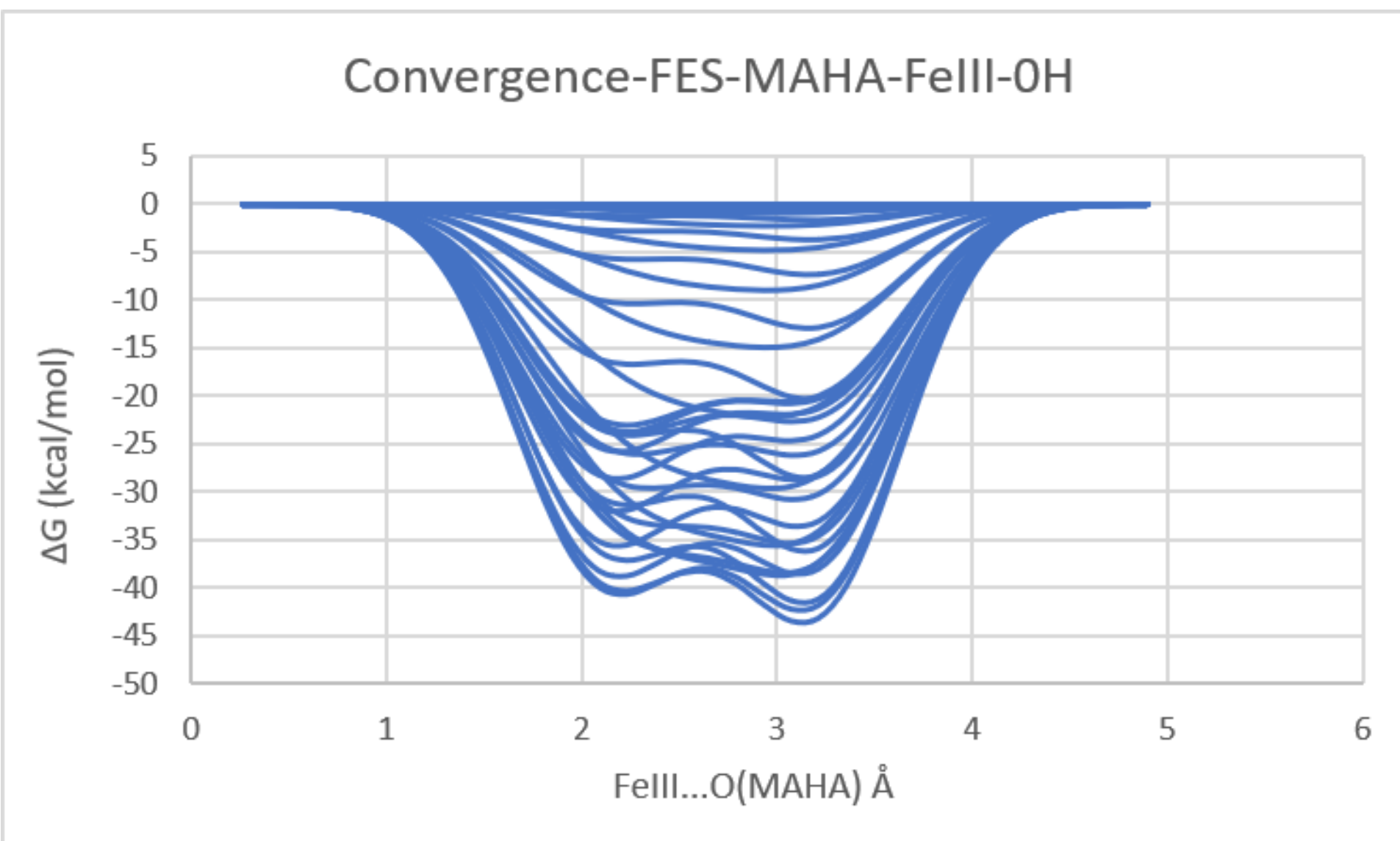
Convergence-FES-MAHA-FeIII-0H
5
0
-5
-10
-15
-20
-25
-30
-35
-40
-45
-50
0
1
2
3
4
5
6
ΔG (kcal/mol)
FeIII...O(MAHA) Å

Figure S2. The variation of all Fe-O distances correlated to the molecular systems discussed in the **main text** and simulated at PBE-D3 DZVP-MOLOPT. One of the Fe-O distances is the selected collective variable (CV2, highlighted in pink) and the remaining five Fe-O distances are unbiased and vary during the simulations.

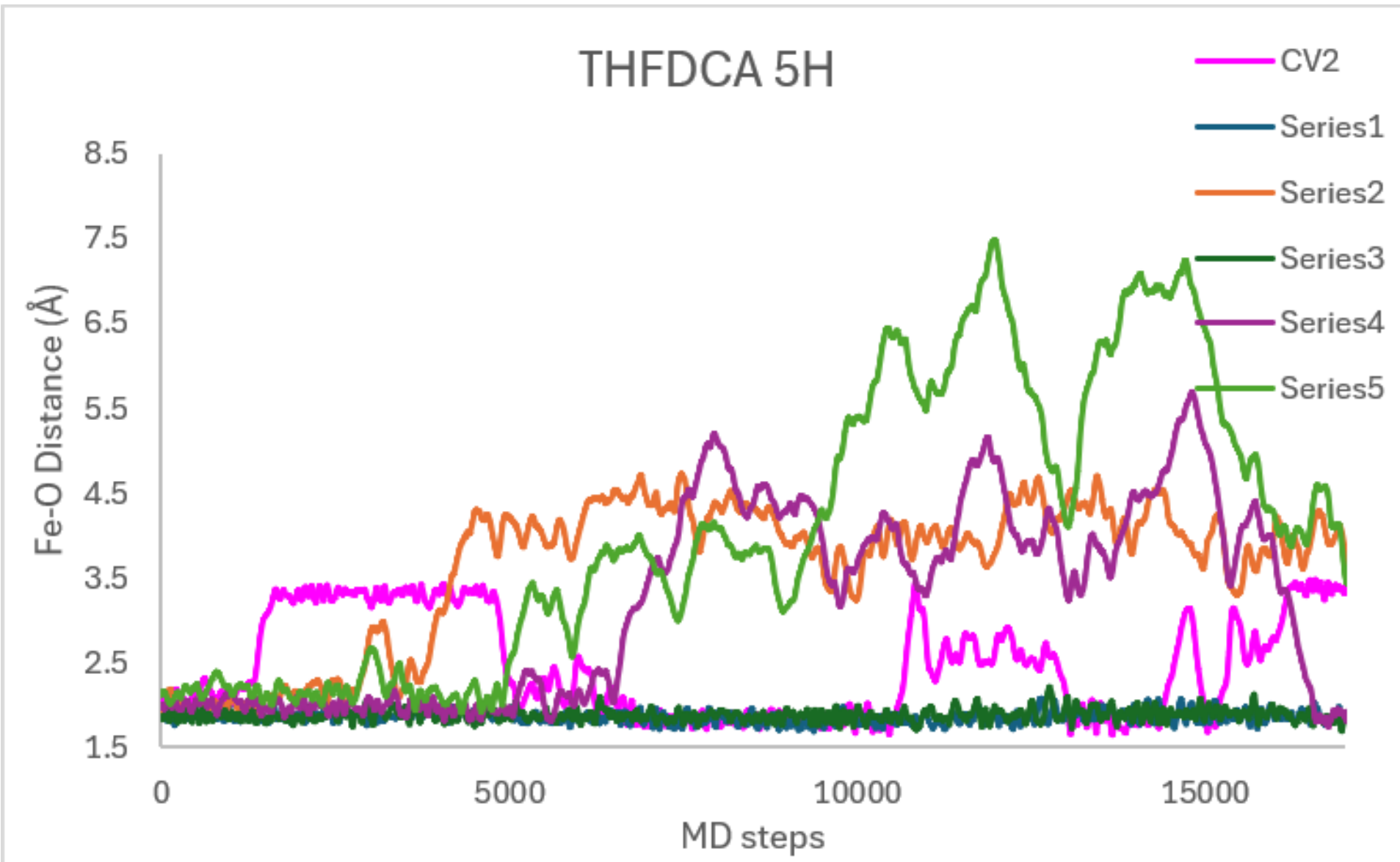

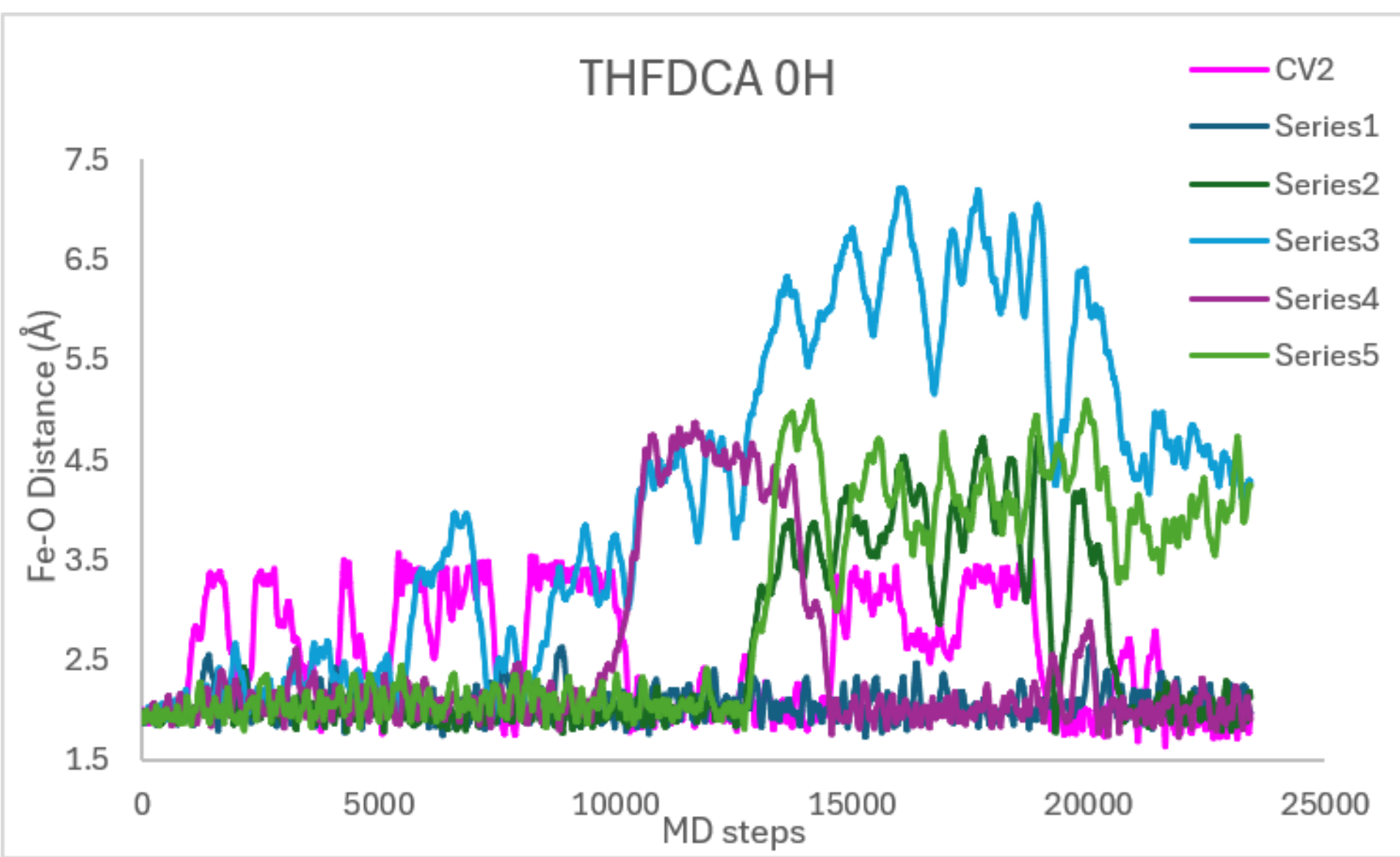

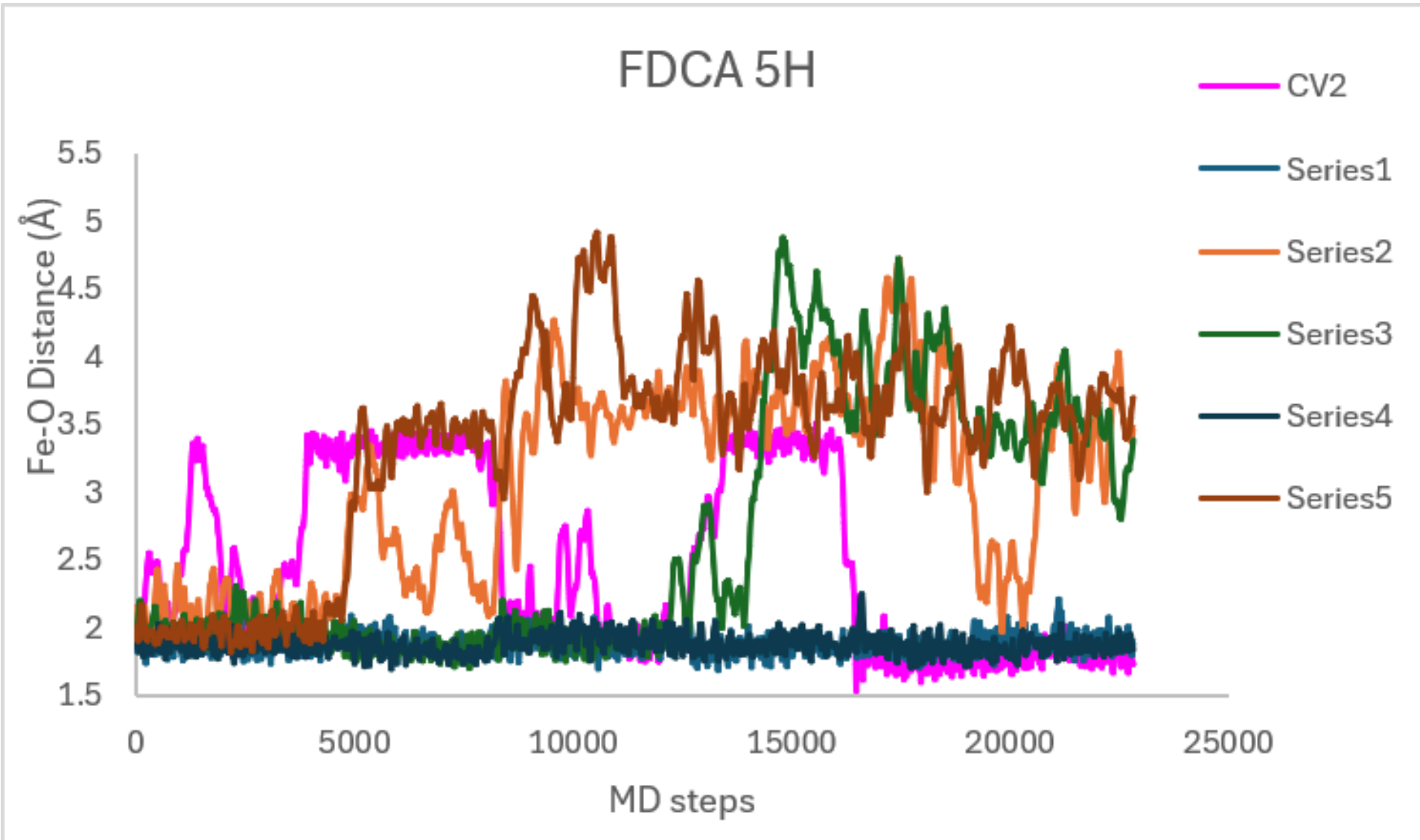
FDCA 5H
CV2
Series1
Series2
Series3
Series4
Series5
Fe-O Distance (Å)
5.5
5
4.5
4
3.5
3
2.5
2
1.5
0
5000
10000
15000
20000
25000
MD steps

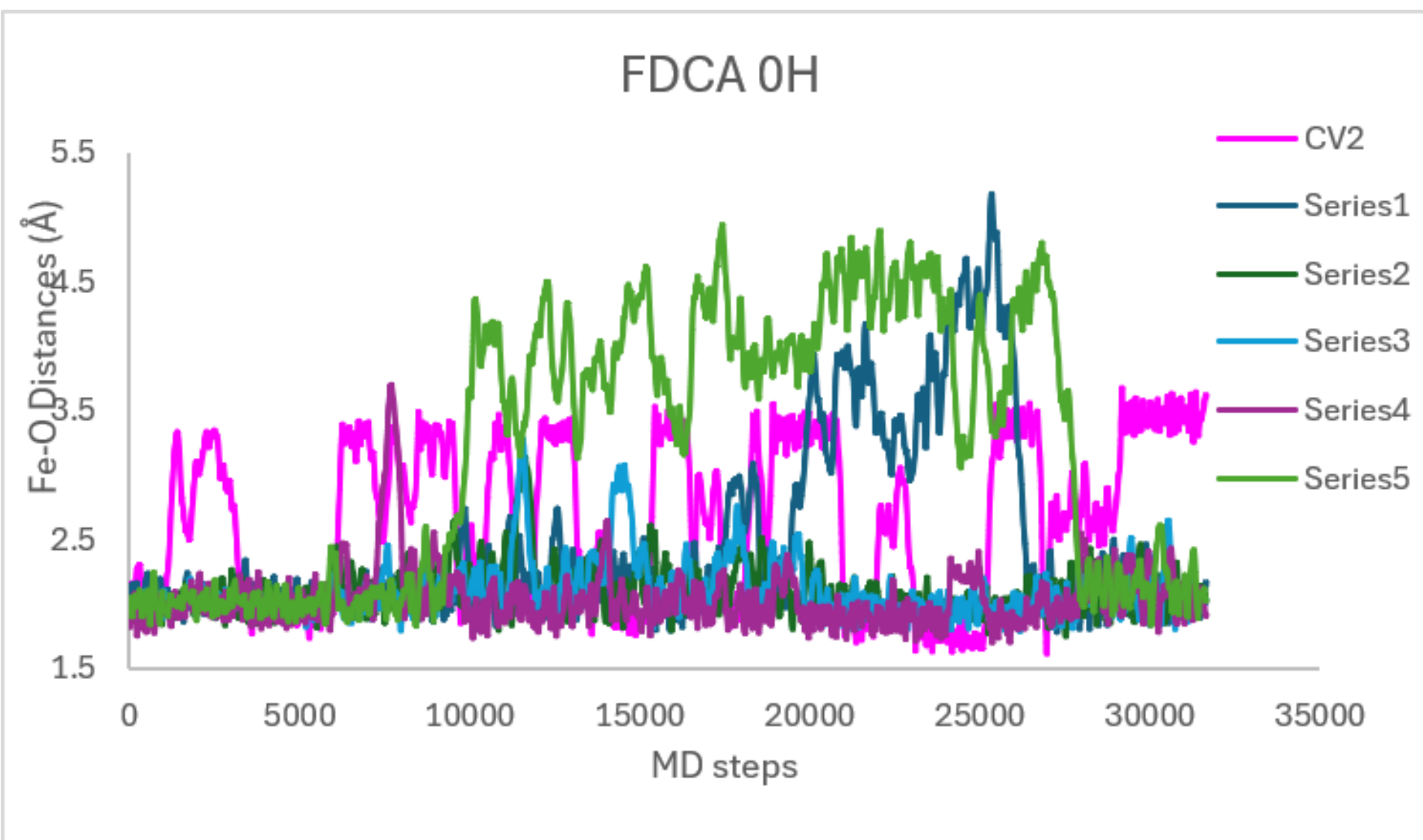
FDCA 0H
CV2
Series1
Series2
Series3
Series4
Series5
Fe-O Distances (Å)
5.5
4.5
3.5
2.5
1.5
0
5000
10000
15000
20000
25000
30000
35000
MD steps

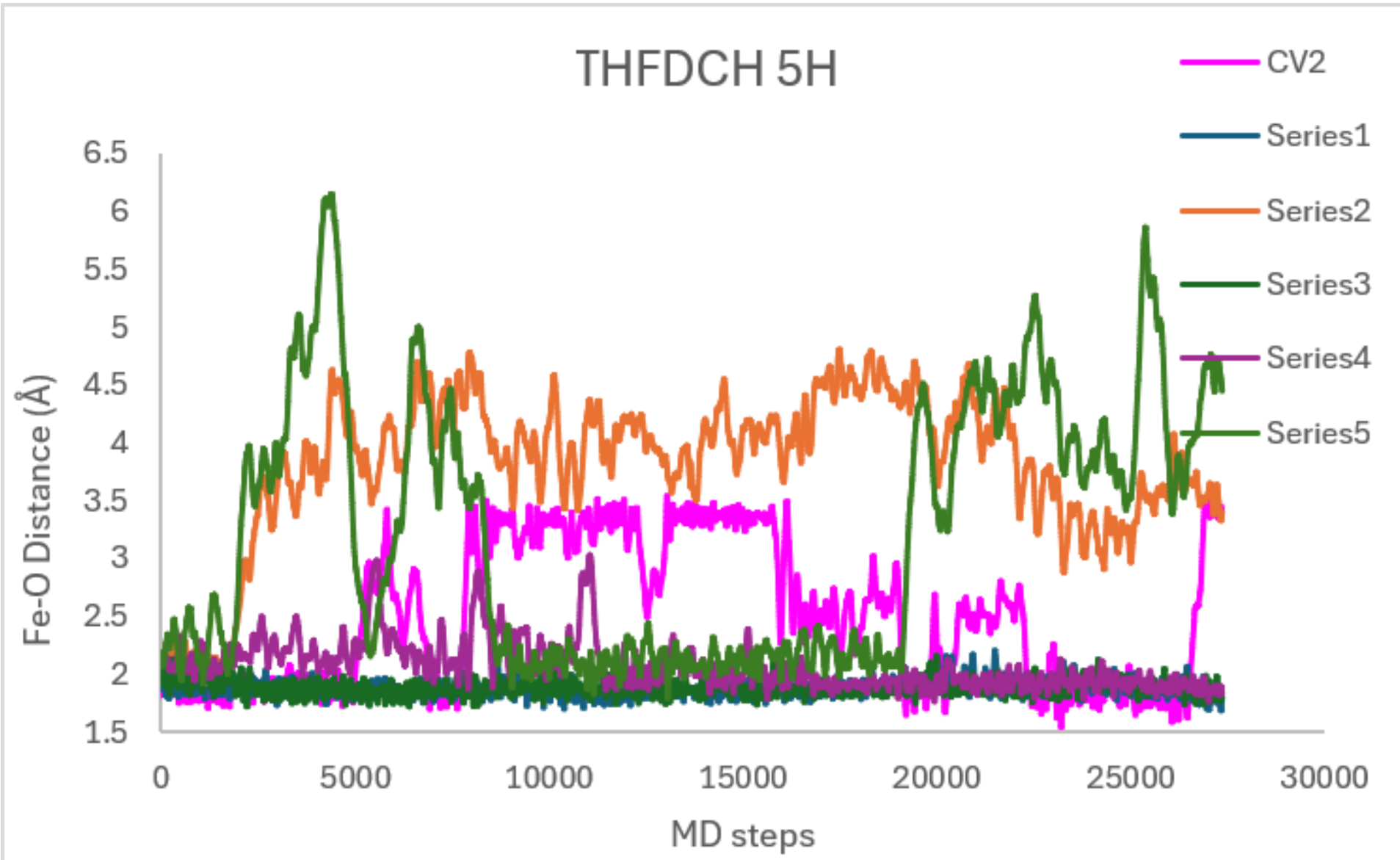
THFDCH 5H
Fe-O Distance (Å)
MD steps
CV2
Series1
Series2
Series3
Series4
Series5
6.5
6
5.5
5
4.5
4
3.5
3
2.5
2
1.5
0
5000
10000
15000
20000
25000
30000

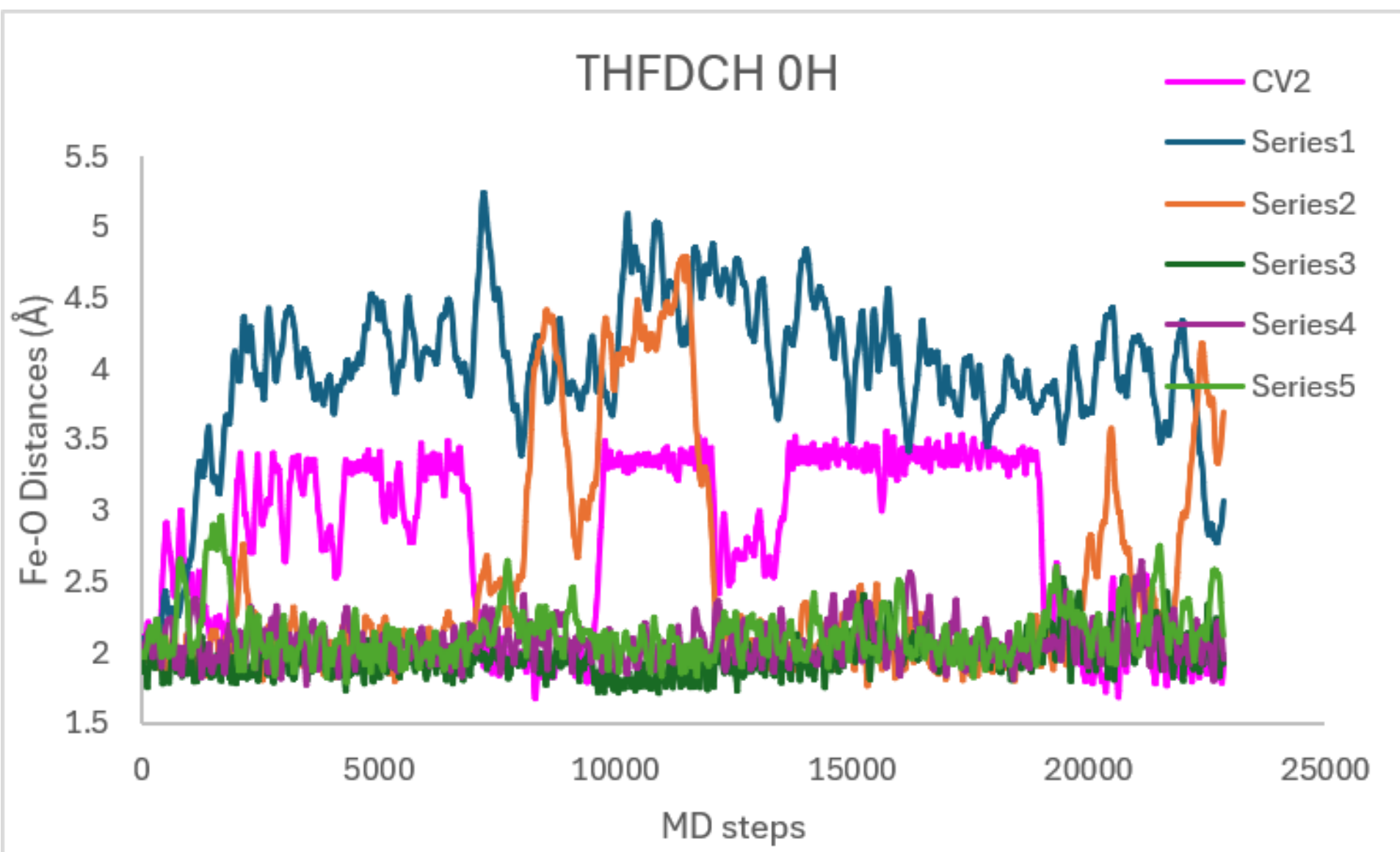
THFDCH 0H
Fe-O Distances (Å)
MD steps
CV2
Series1
Series2
Series3
Series4
Series5
5.5
5
4.5
4
3.5
3
2.5
2
1.5
0
5000
10000
15000
20000
25000

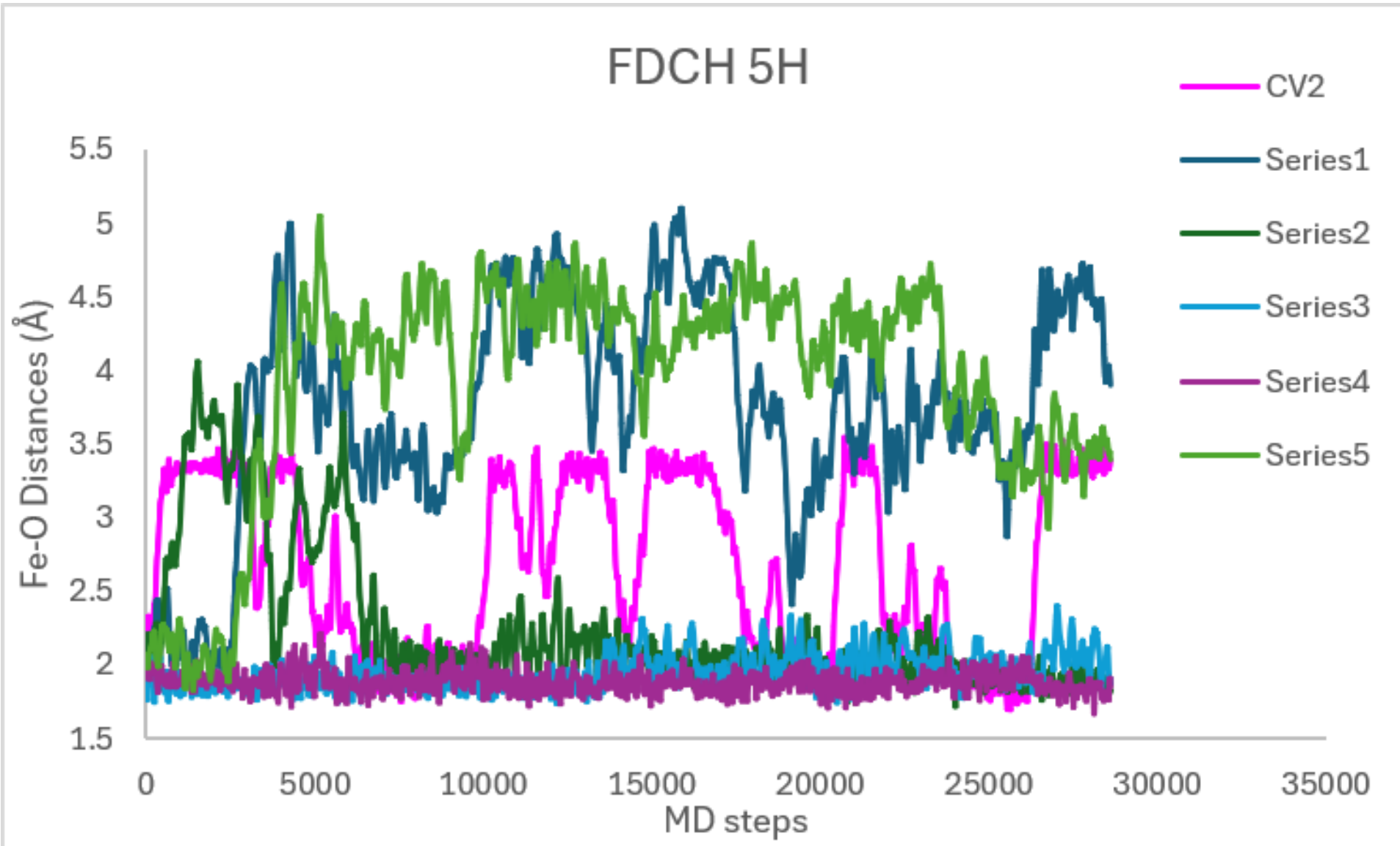

FDCH 5H
CV2
Series1
Series2
Series3
Series4
Series5
Fe-O Distances (Å)
5.5
5
4.5
4
3.5
3
2.5
2
1.5
0
5000
10000
15000
20000
25000
30000
35000
MD steps

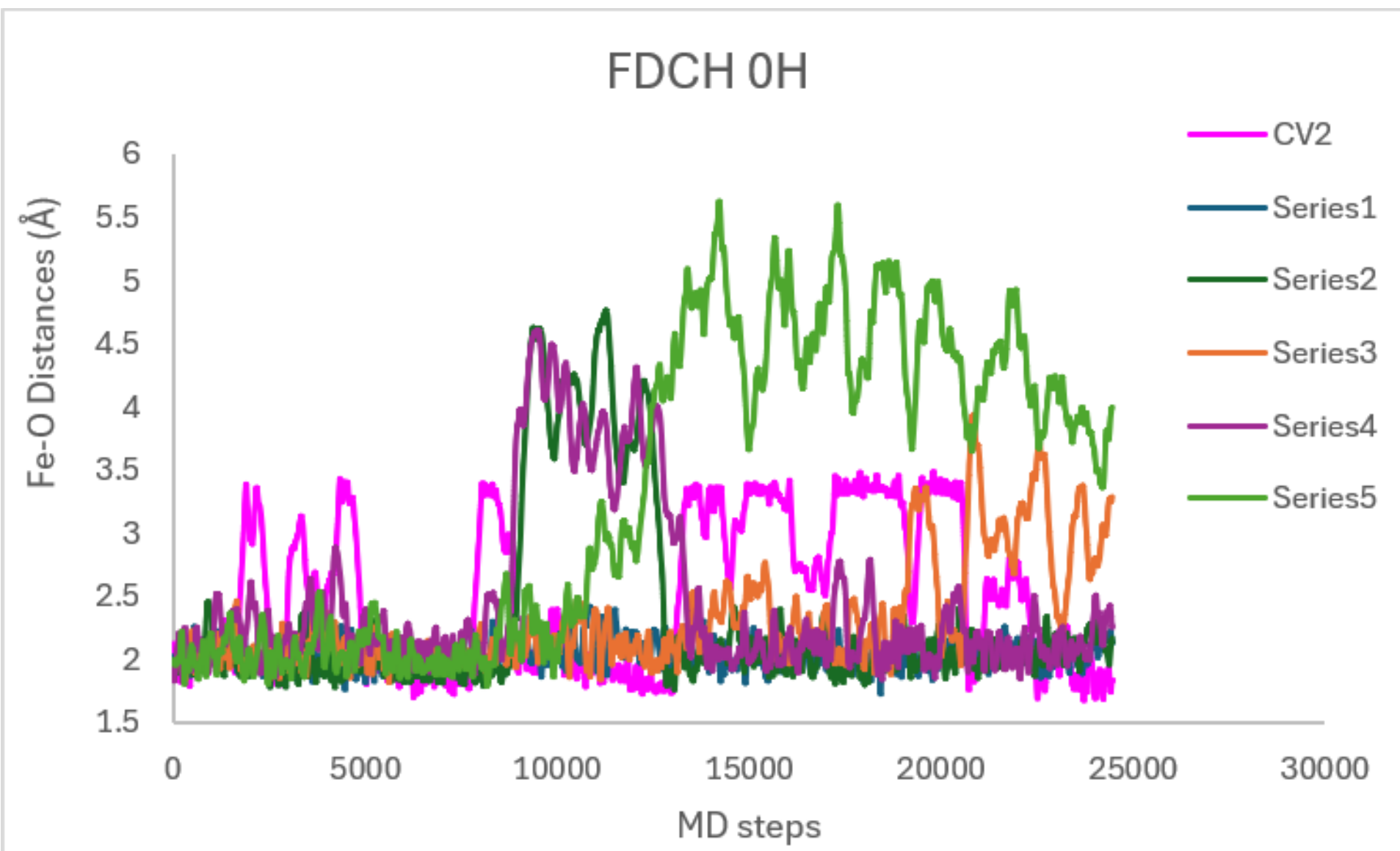

FDCH 0H
CV2
Series1
Series2
Series3
Series4
Series5
Fe-O Distances (Å)
6
5.5
5
4.5
4
3.5
3
2.5
2
1.5
0
5000
10000
15000
20000
25000
30000
MD steps

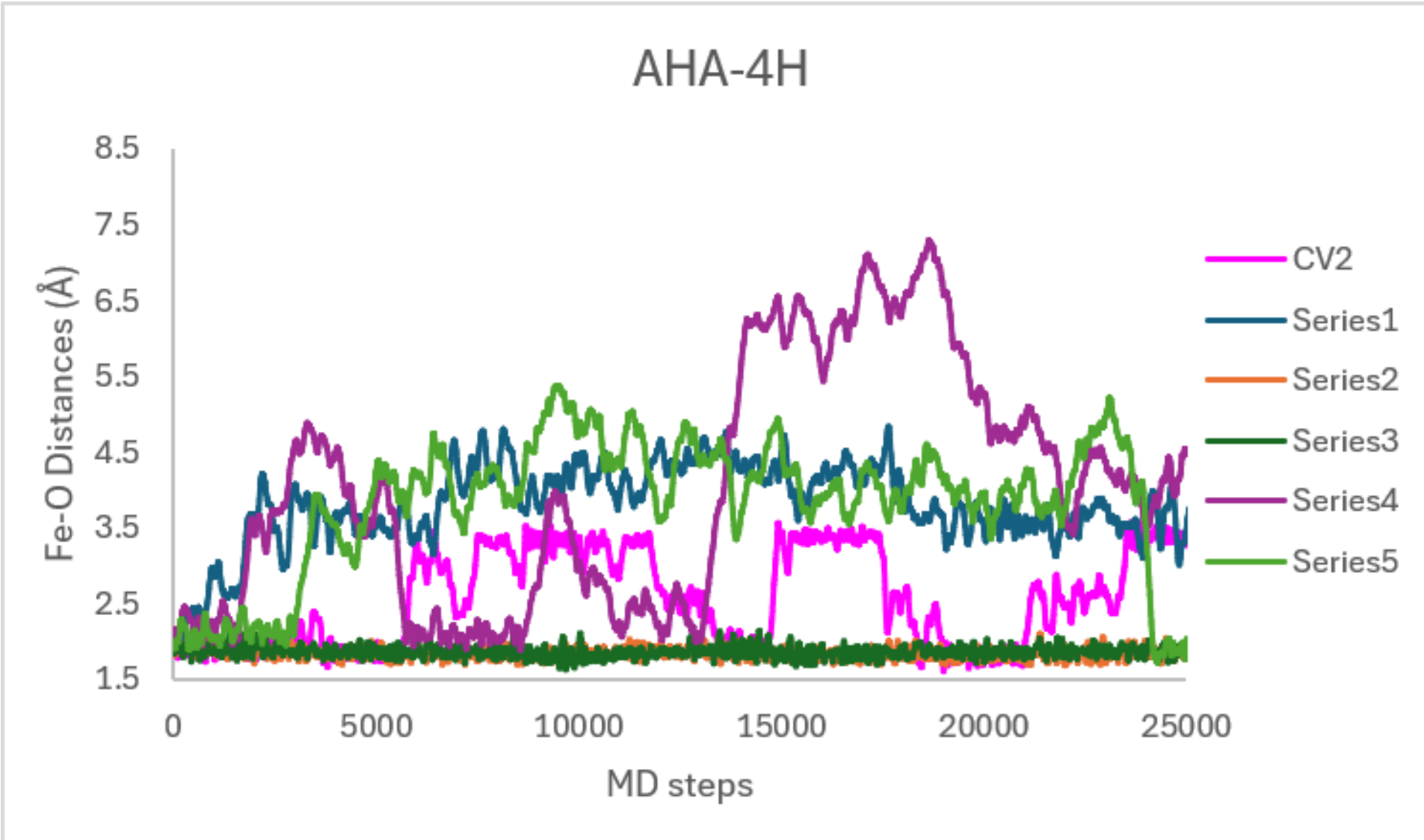
AHA-4H
Fe-O Distances (Å)
8.5
7.5
6.5
5.5
4.5
3.5
2.5
1.5
0
5000
10000
15000
20000
25000
MD steps
CV2
Series1
Series2
Series3
Series4
Series5

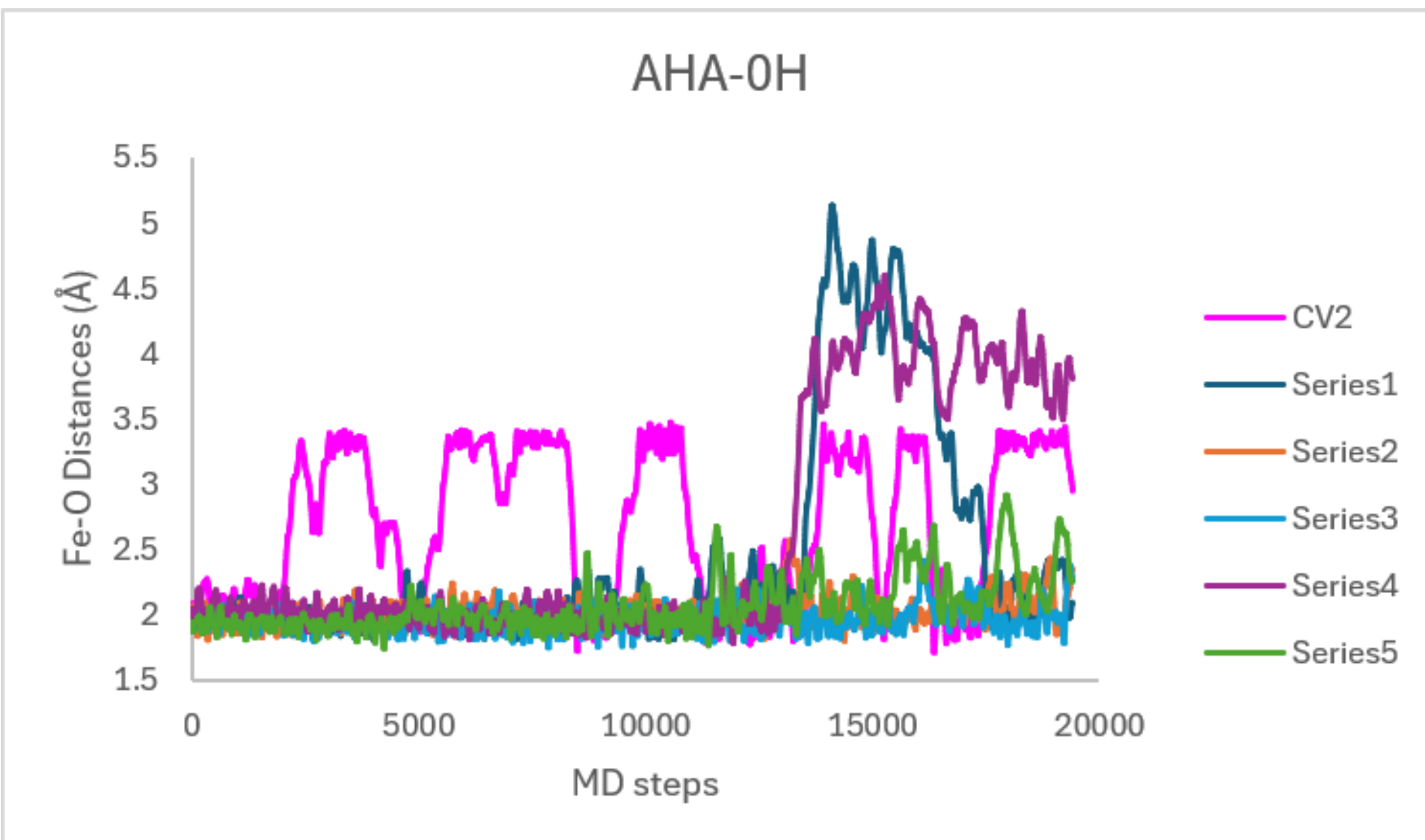
AHA-0H
Fe-O Distances (Å)
5.5
5
4.5
4
3.5
3
2.5
2
1.5
0
5000
10000
15000
20000
MD steps
CV2
Series1
Series2
Series3
Series4
Series5

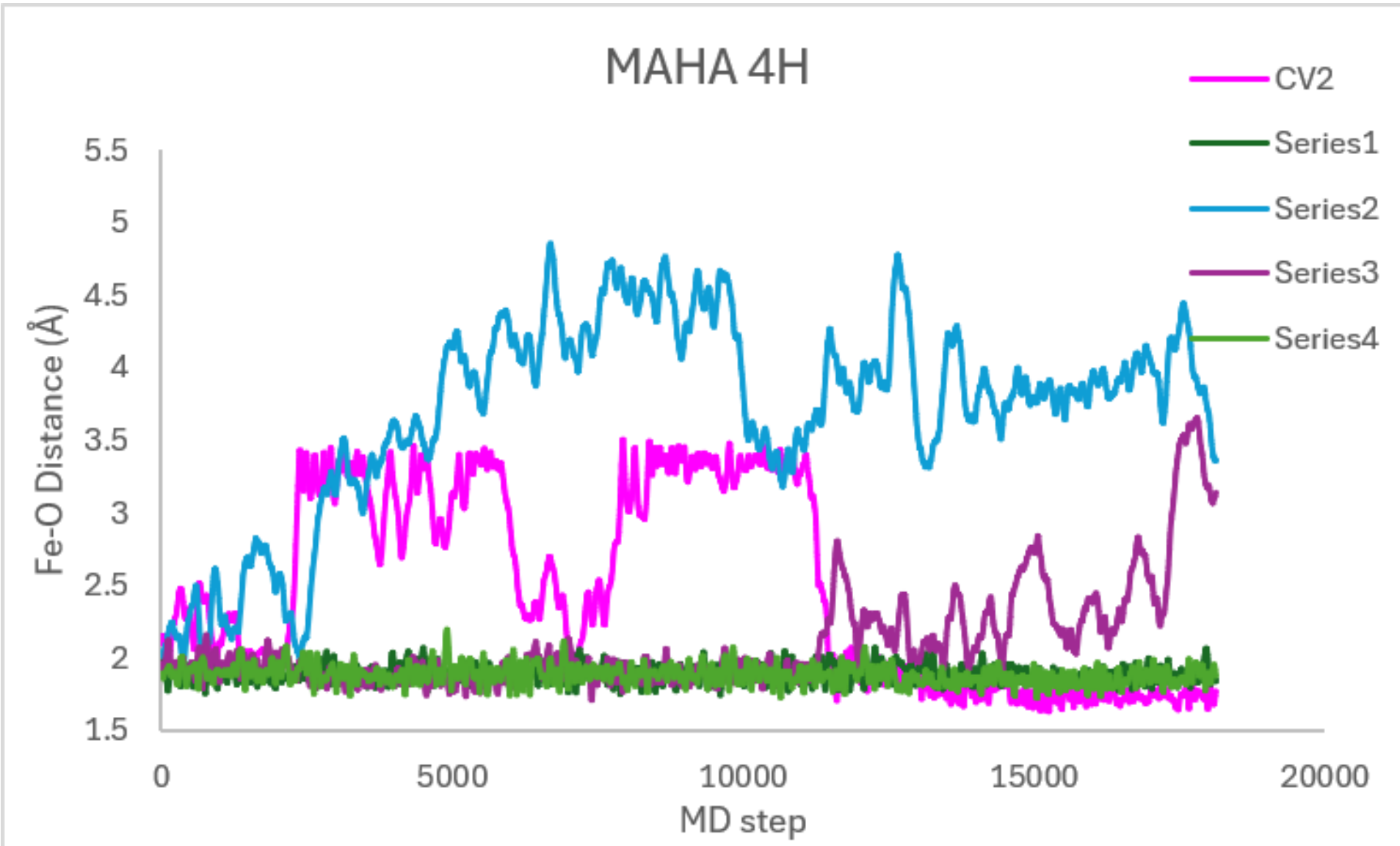
MAHA 4H
CV2
Series1
Series2
Series3
Series4
Fe-O Distance (Å)
5.5
5
4.5
4
3.5
3
2.5
2
1.5
0
5000
10000
15000
20000
MD step

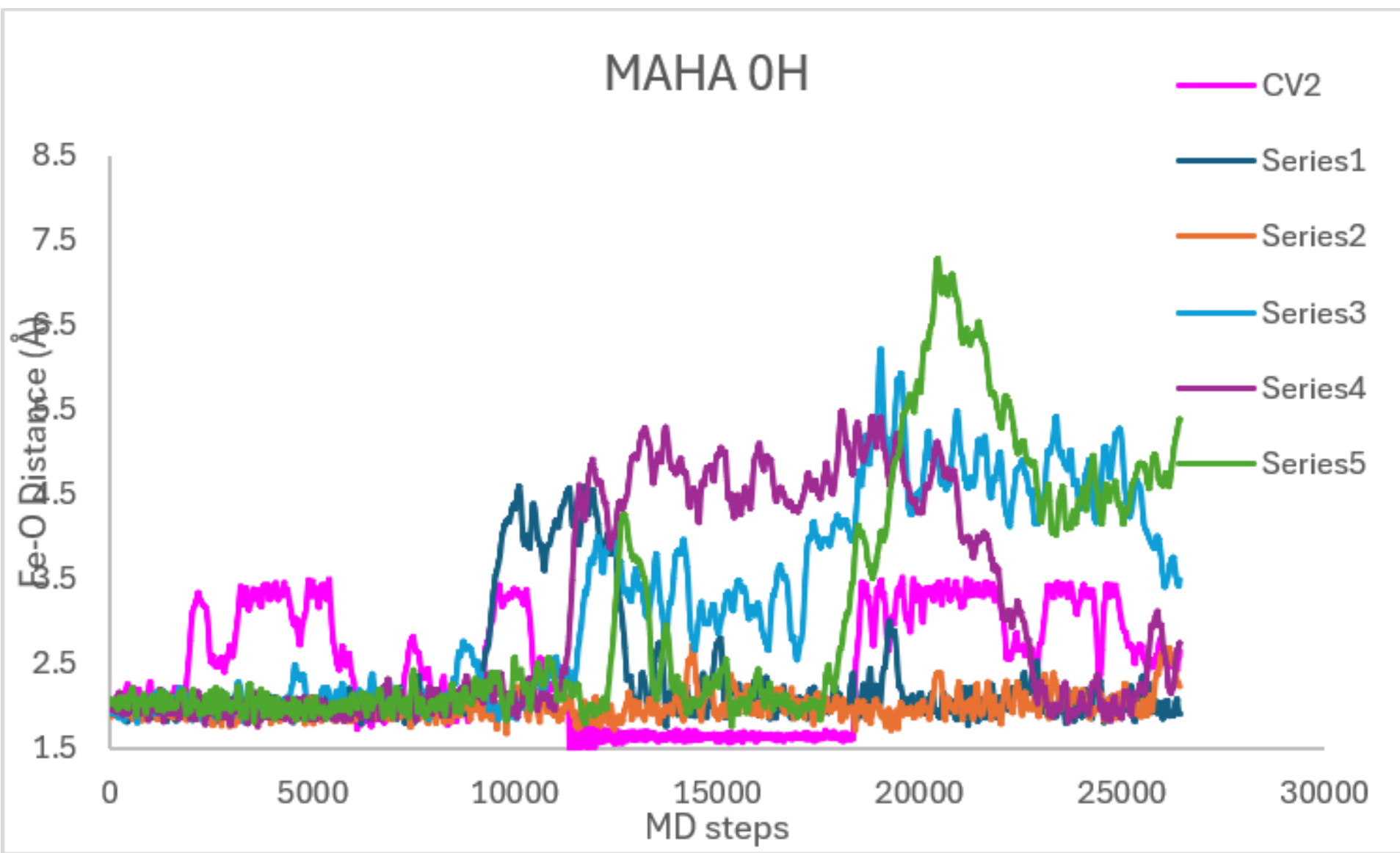
MAHA 0H
CV2
Series1
Series2
Series3
Series4
Series5
Fe-O Distance (Å)
8.5
7.5
1.5
2.5
0
5000
10000
15000
20000
25000
30000
MD steps

Figure S3. The variation of CVs (distances) and FES for the molecular systems including 12 explicit water molecules optimized at BLYP-D3 MOLOPT-DZVP level of theory

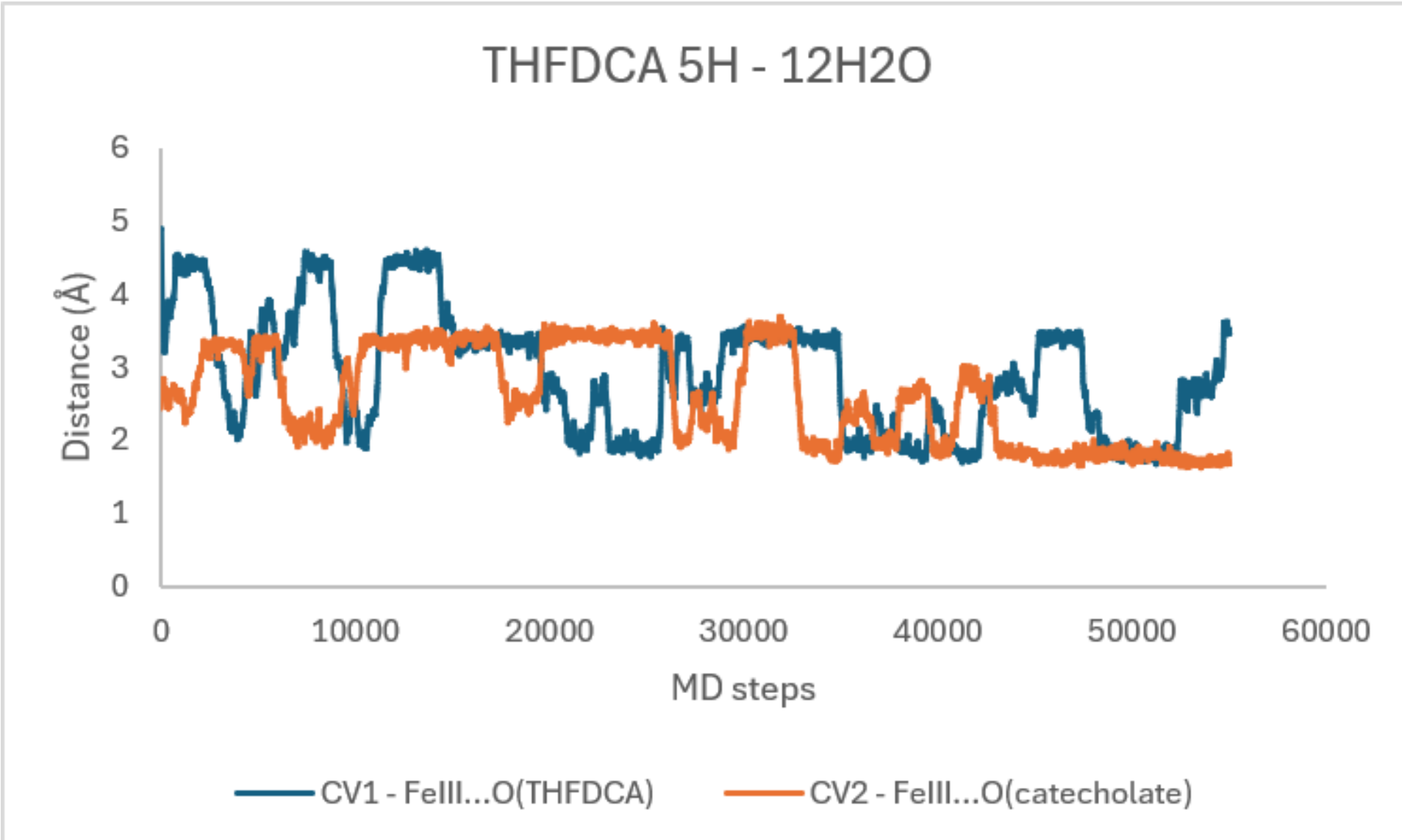

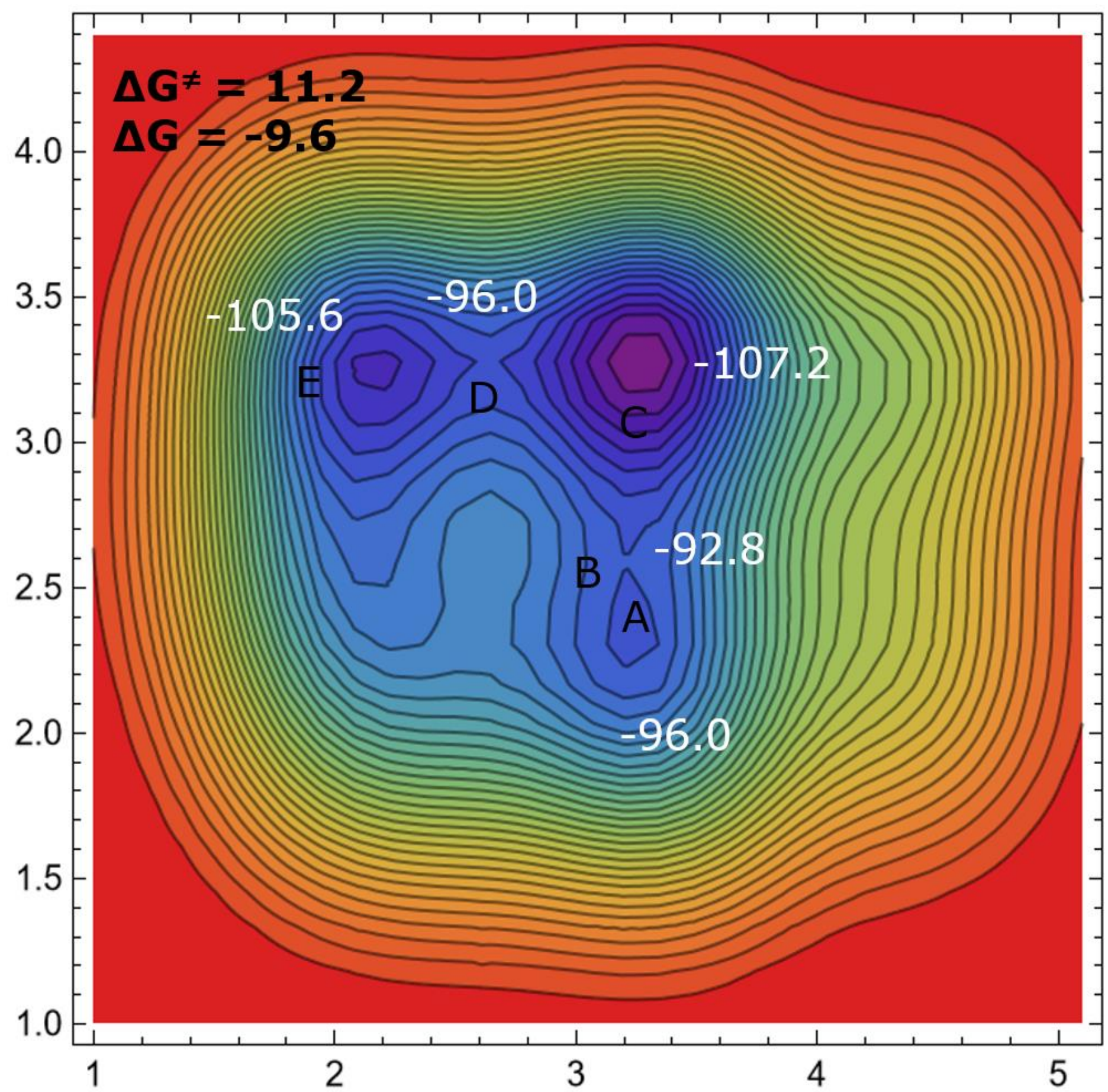

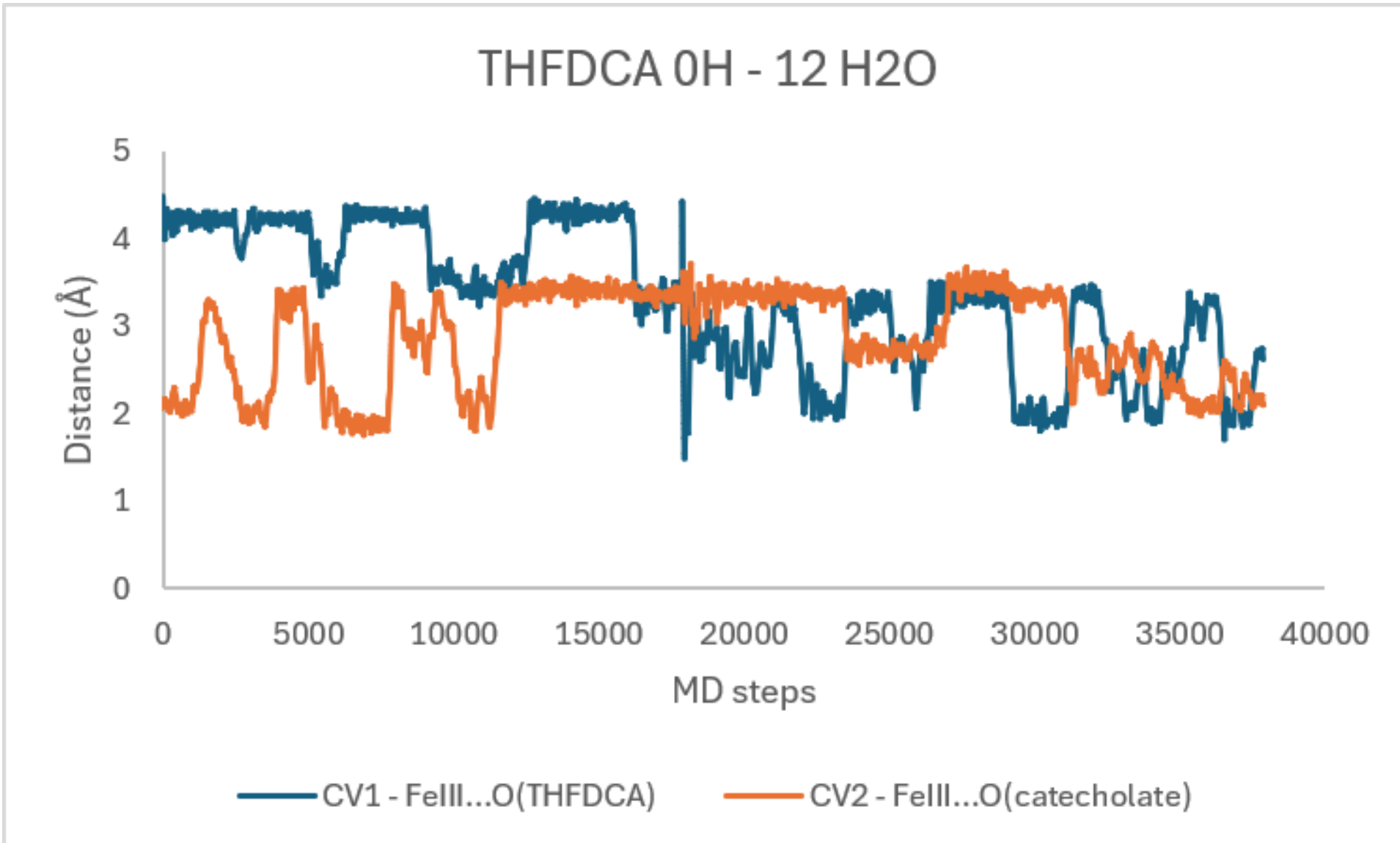
THFDCA 0H - 12 H2O
Distance (Å)
MD steps
CV1 - FeIII...O(THFDCA)
CV2 - FeIII...O(catecholate)

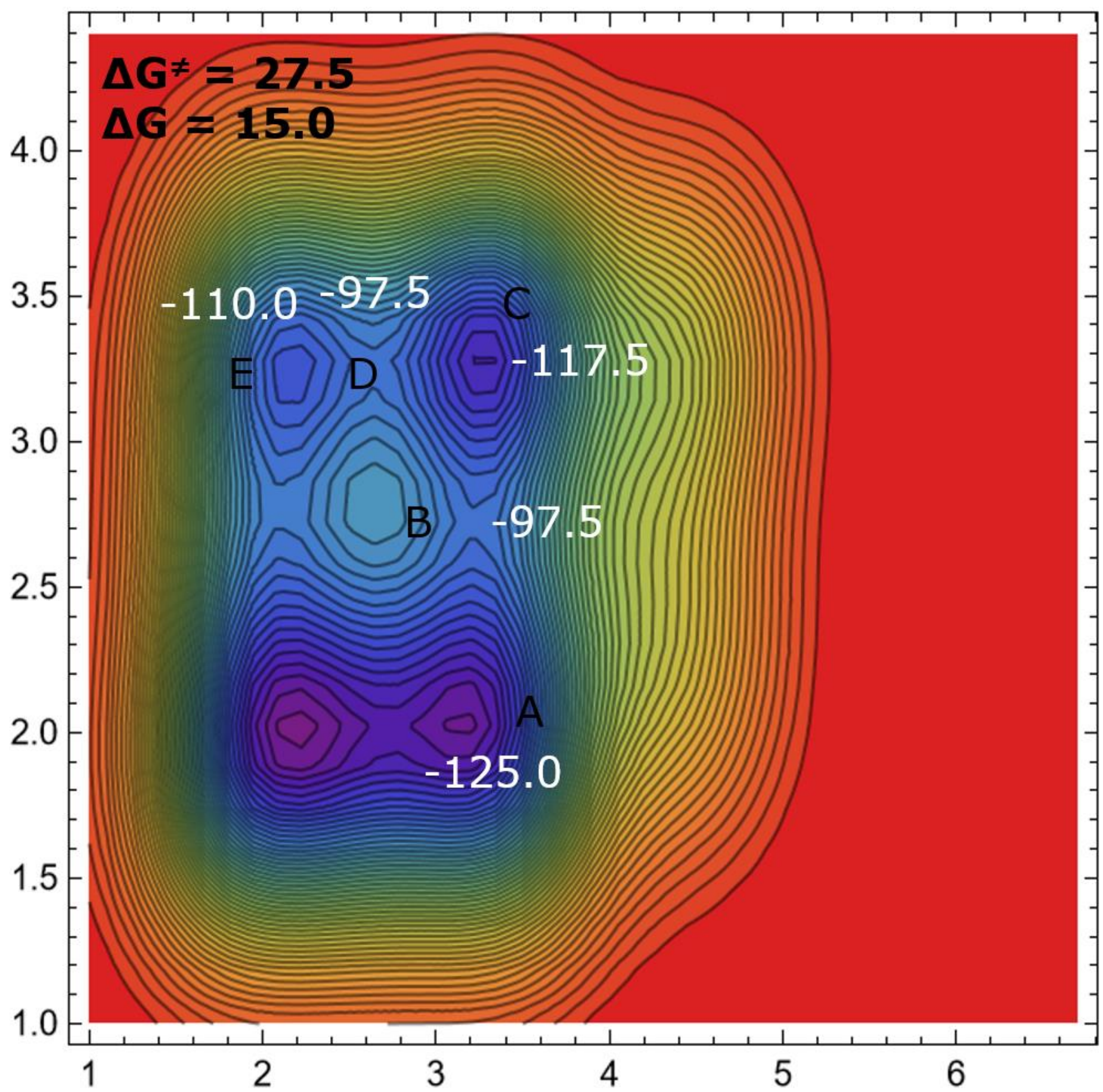
ΔG≠ = 27.5
ΔG = 15.0
-110.0
-97.5
C
E
D
-117.5
B
-97.5
A
-125.0

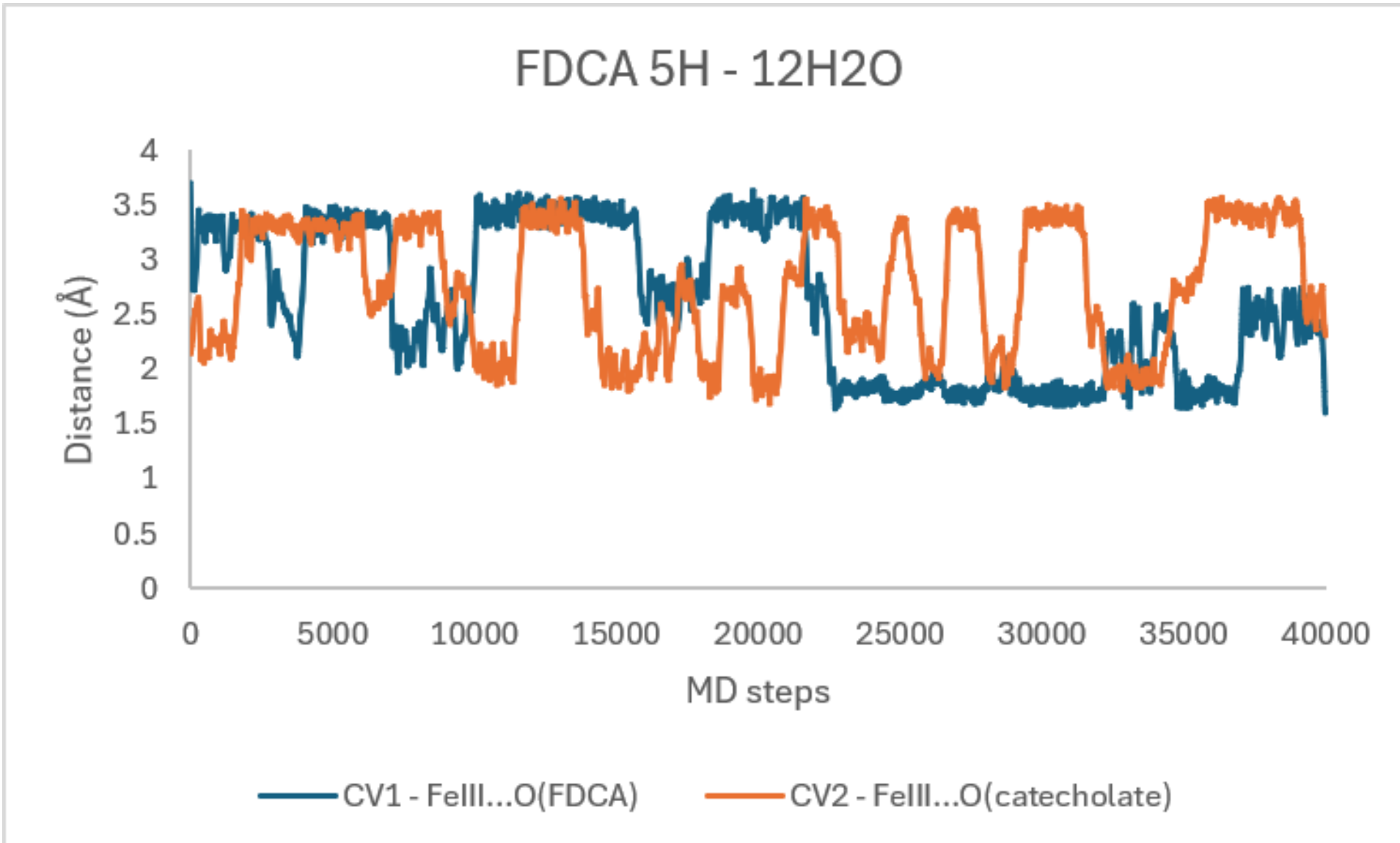
FDCA 5H - 12H2O
Distance (Å)
4
3.5
3
2.5
2
1.5
1
0.5
0
0
5000
10000
15000
20000
25000
30000
35000
40000
MD steps
CV1 - FeIII...O(FDCA)
CV2 - FeIII...O(catecholate)

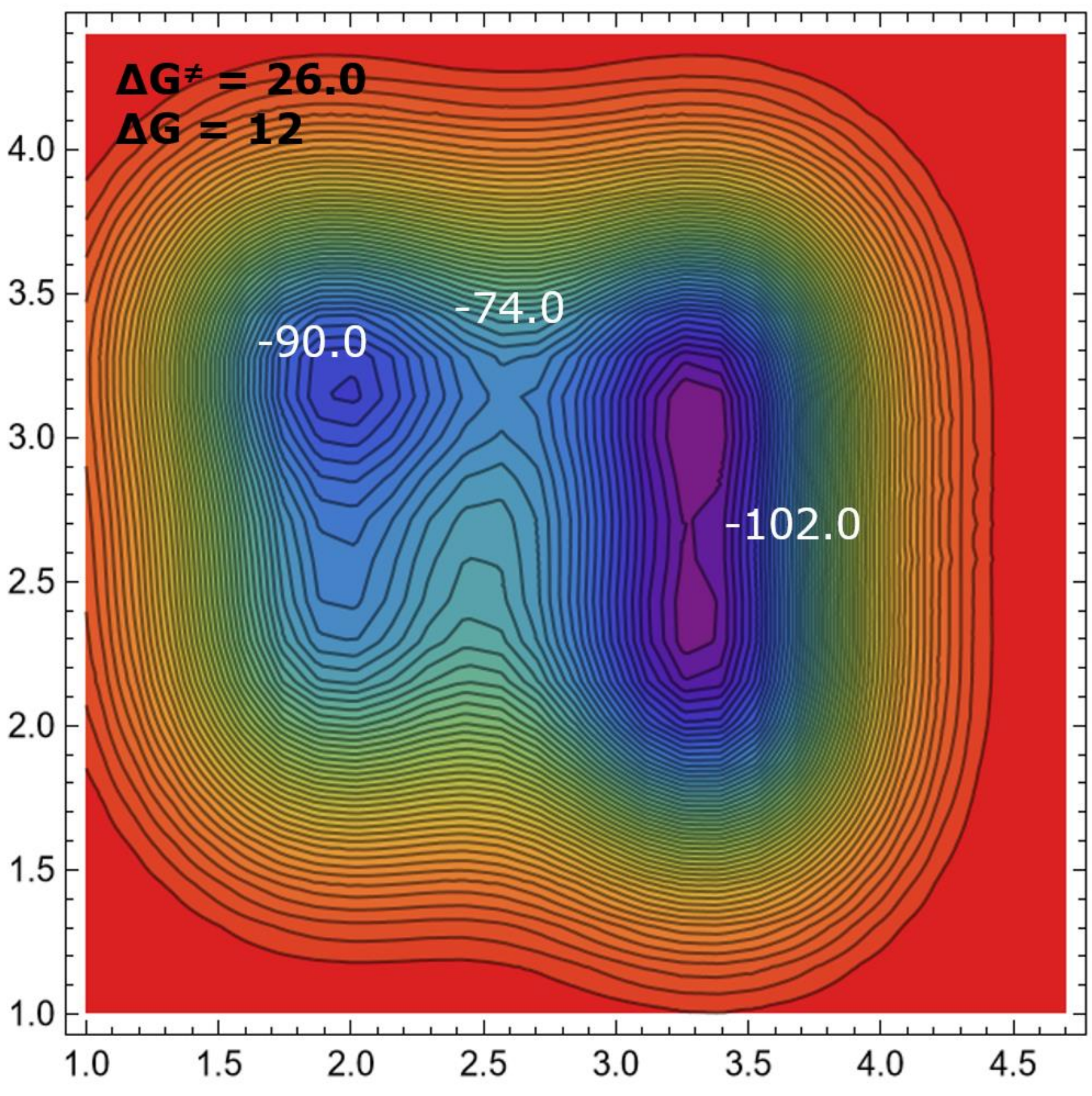
ΔG≠ = 26.0
ΔG = 12
-90.0
-74.0
-102.0
4.0
3.5
3.0
2.5
2.0
1.5
1.0
1.0
1.5
2.0
2.5
3.0
3.5
4.0
4.5

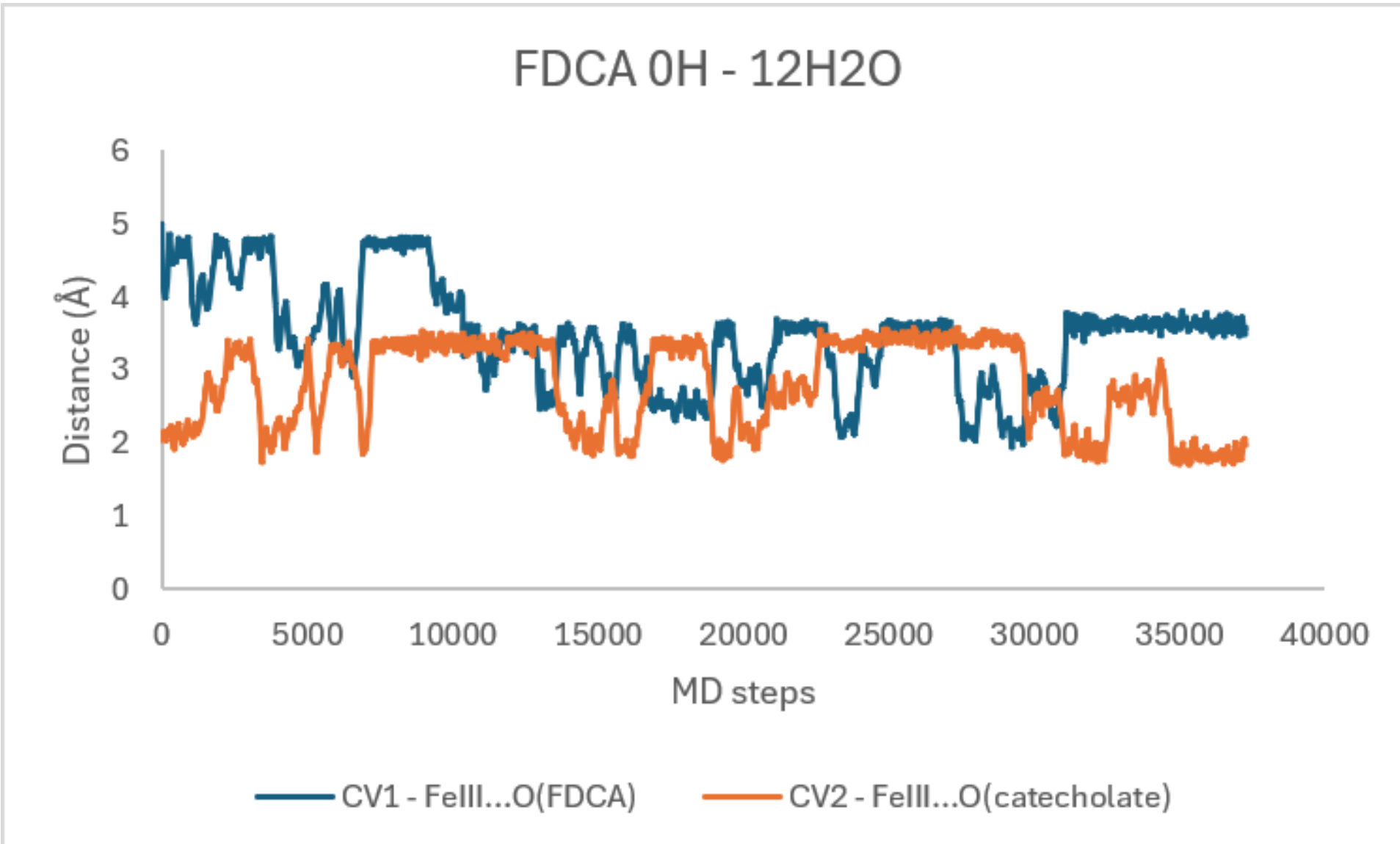

FDCA 0H - 12H2O
Distance (Å)
6
5
4
3
2
1
0
0
5000
10000
15000
20000
25000
30000
35000
40000
MD steps
CV1 - FeIII...O(FDCA)
CV2 - FeIII...O(catecholate)

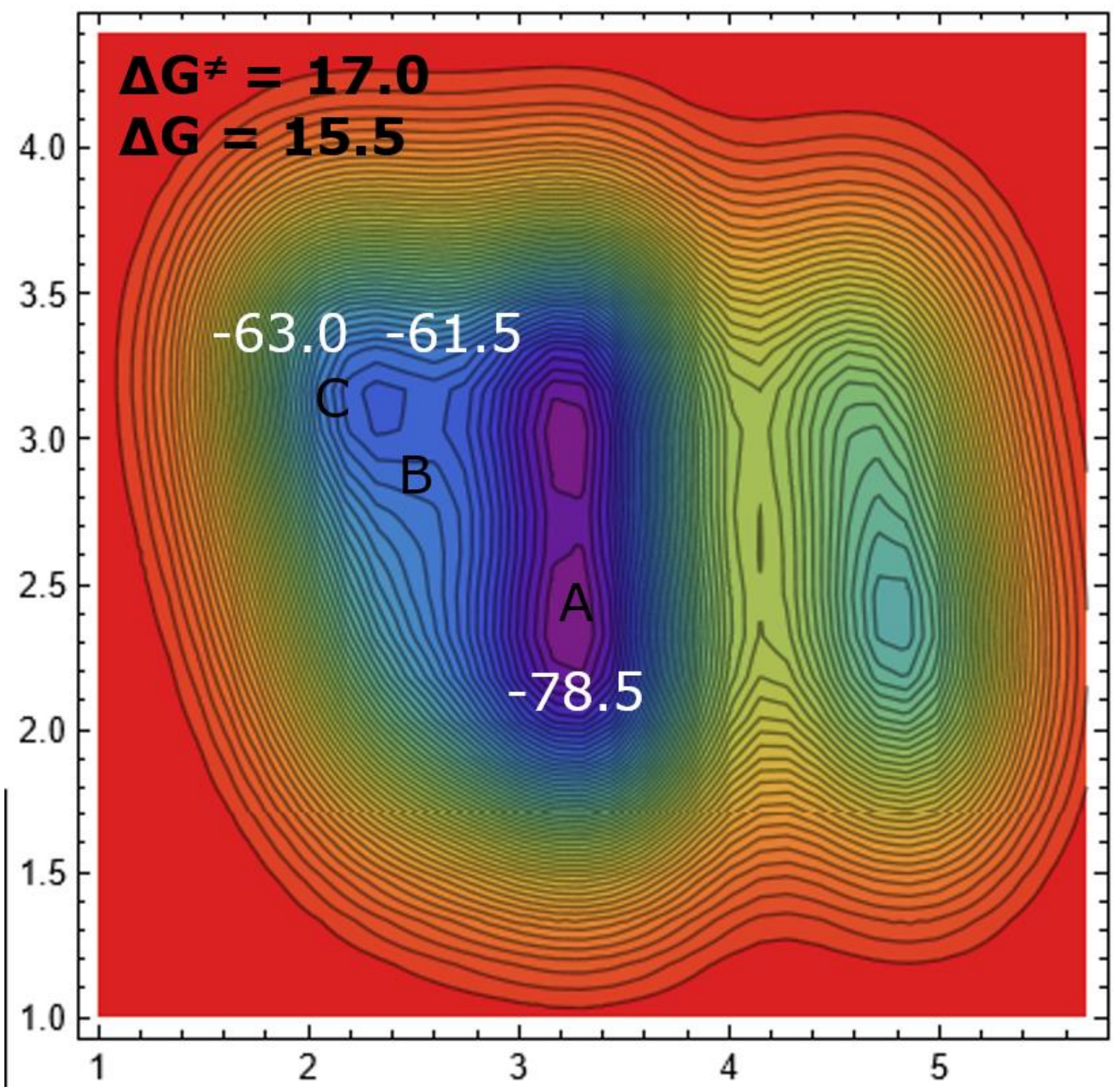

ΔG≠ = 17.0
ΔG = 15.5
-63.0
-61.5
C
B
A
-78.5
4.0
3.5
3.0
2.5
2.0
1.5
1.0
1
2
3
4
5

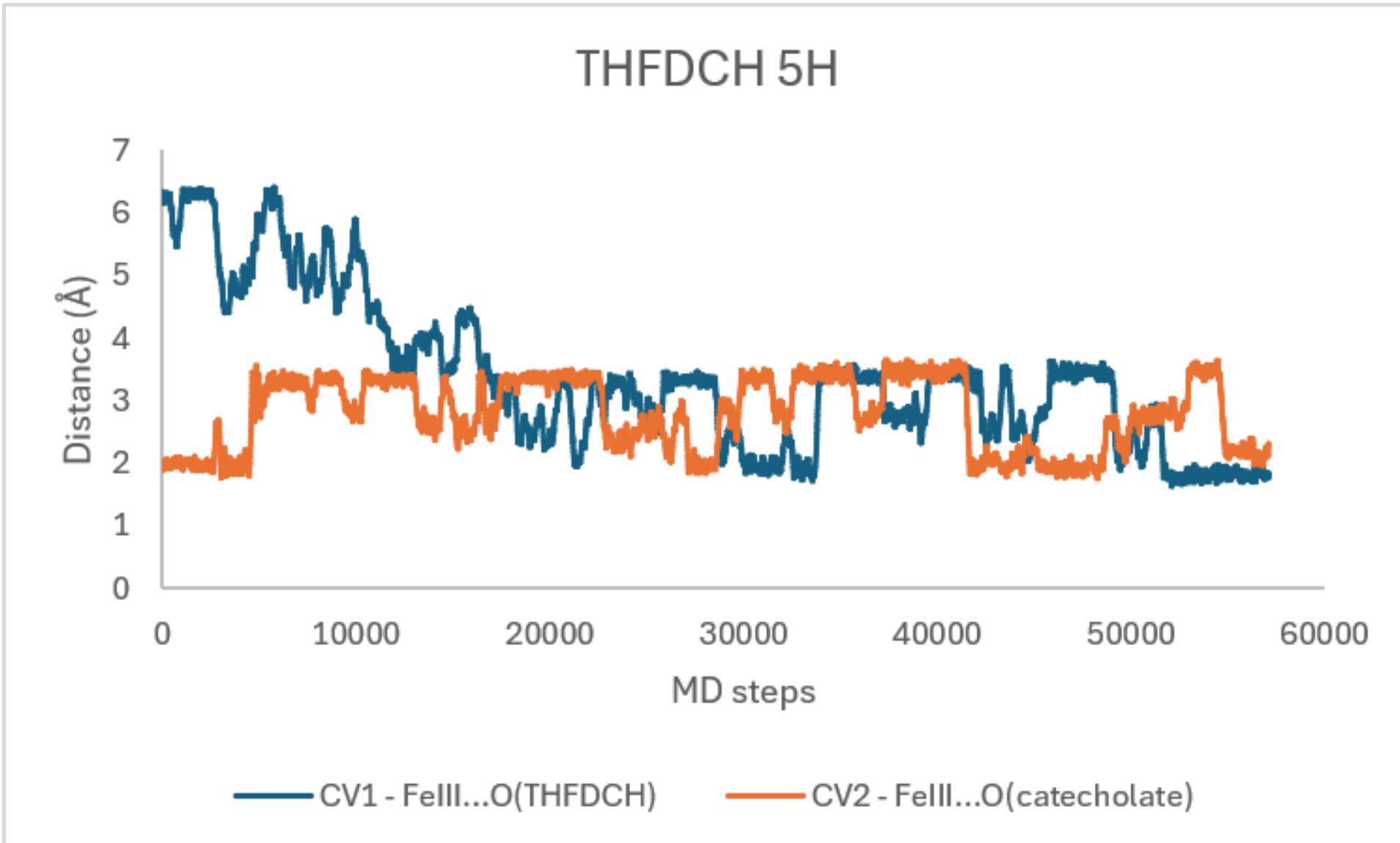
THFDCH 5H
Distance (Å)
MD steps
0
1
2
3
4
5
6
7
0
10000
20000
30000
40000
50000
60000
CV1 - FeIII...O(THFDCH)
CV2 - FeIII...O(catecholate)

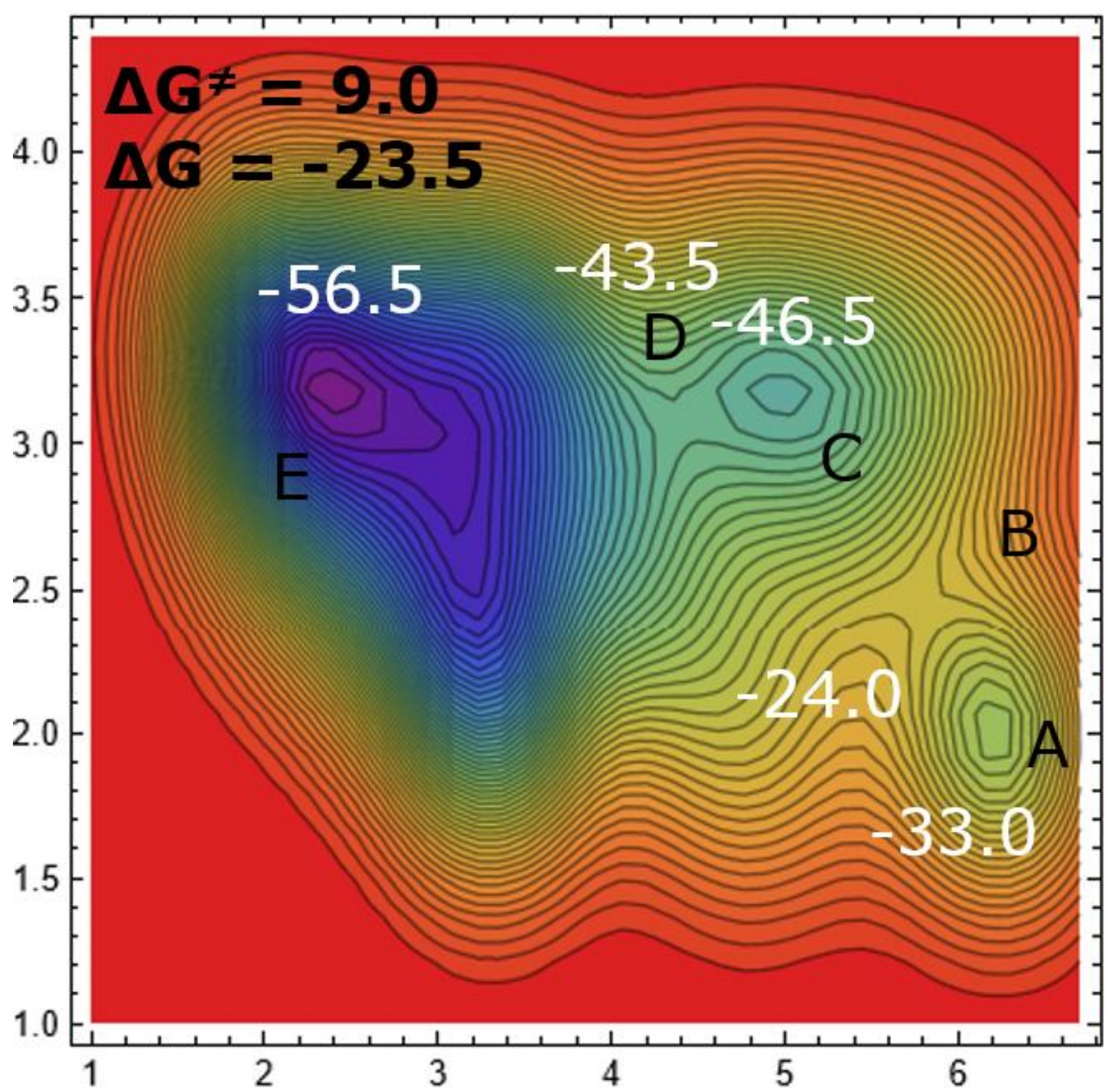
ΔG≠ = 9.0
ΔG = -23.5
-56.5
-43.5
D
-46.5
E
C
B
-24.0
A
-33.0
4.0
3.5
3.0
2.5
2.0
1.5
1.0
1
2
3
4
5
6

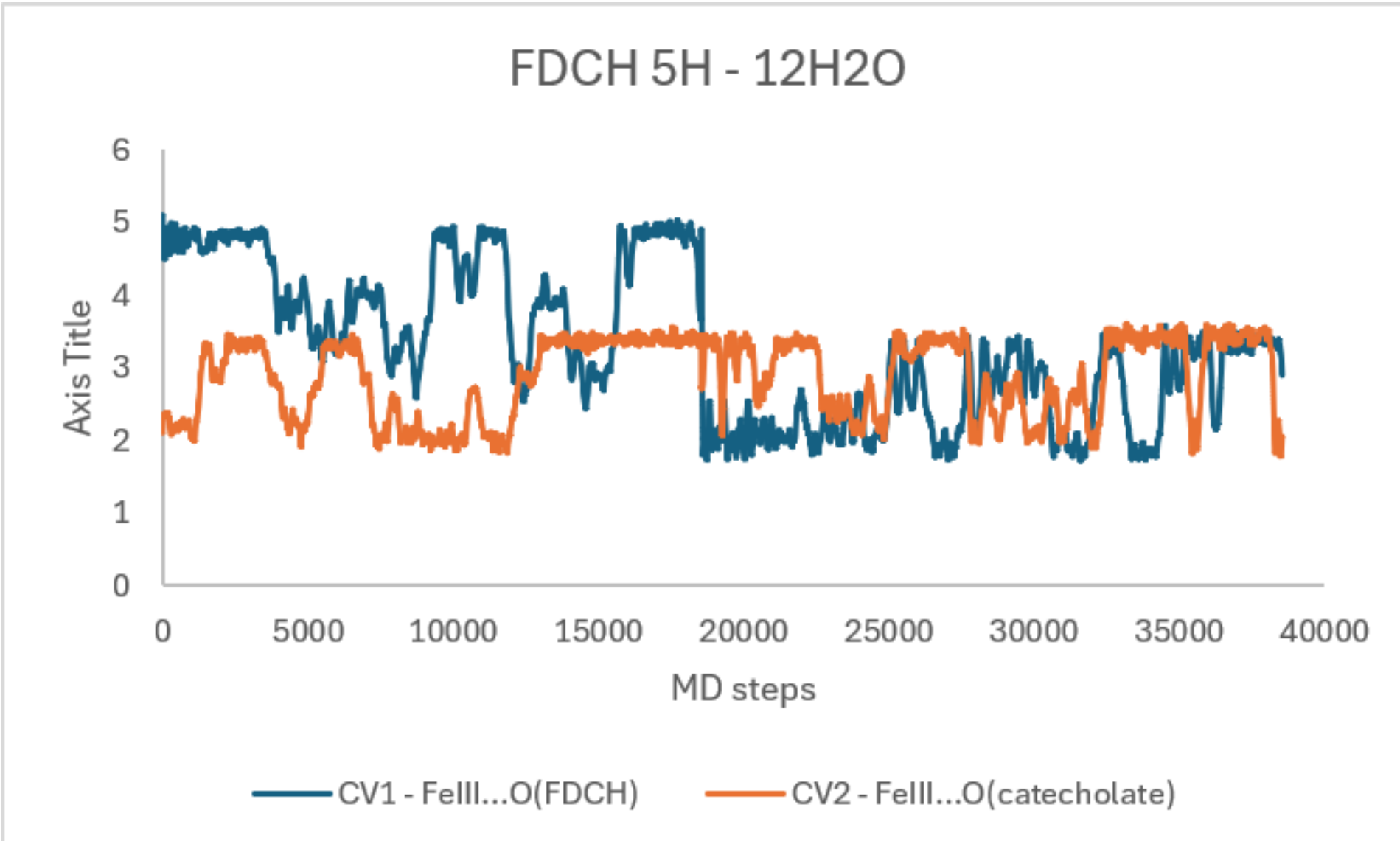

FDCH 5H - 12H2O
Axis Title
MD steps
0
1
2
3
4
5
6
0
5000
10000
15000
20000
25000
30000
35000
40000
CV1 - FeIII...O(FDCH)
CV2 - FeIII...O(catecholate)

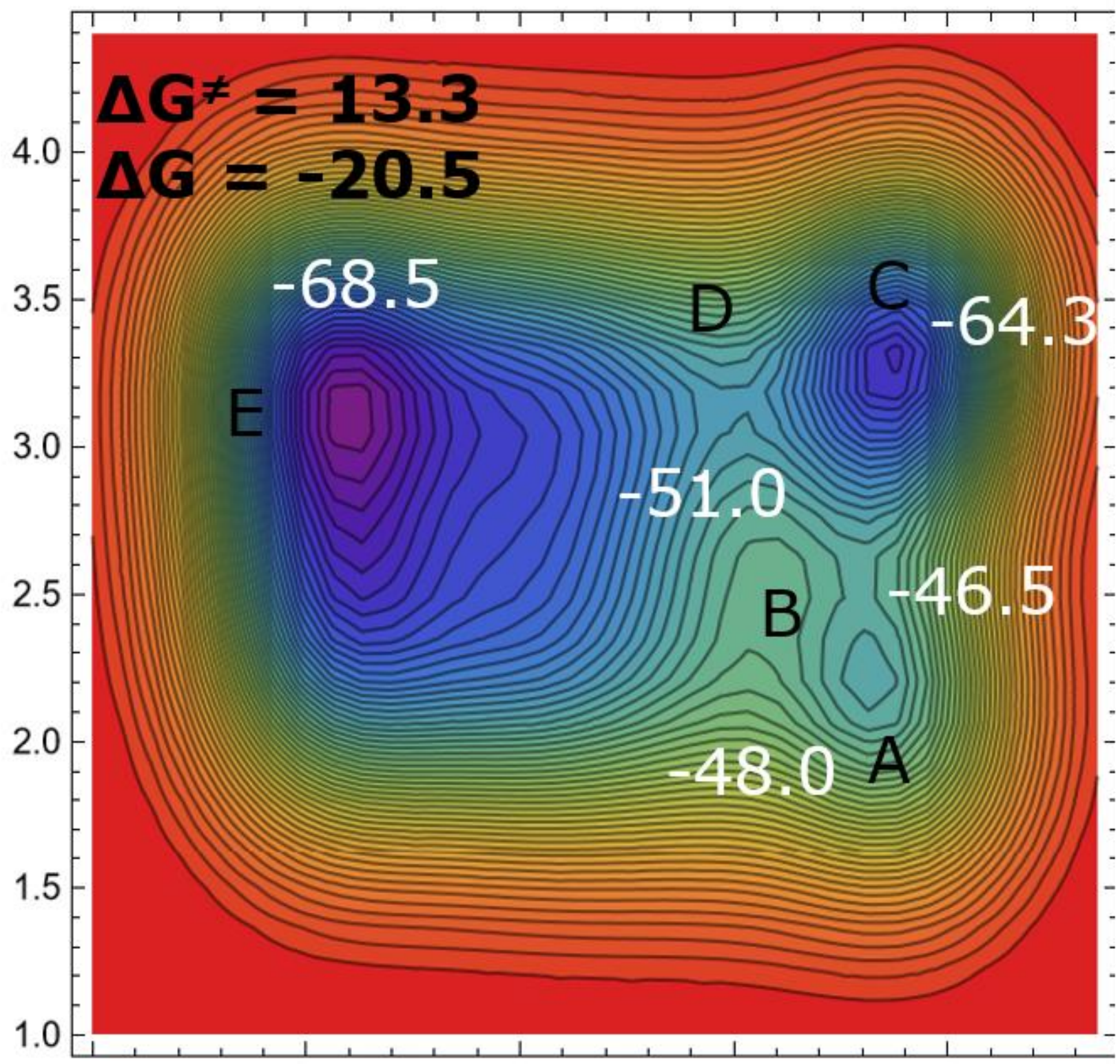

ΔG≠ = 13.3
ΔG = -20.5
-68.5
D
C
-64.3
E
-51.0
-46.5
B
-48.0
A
4.0
3.5
3.0
2.5
2.0
1.5
1.0

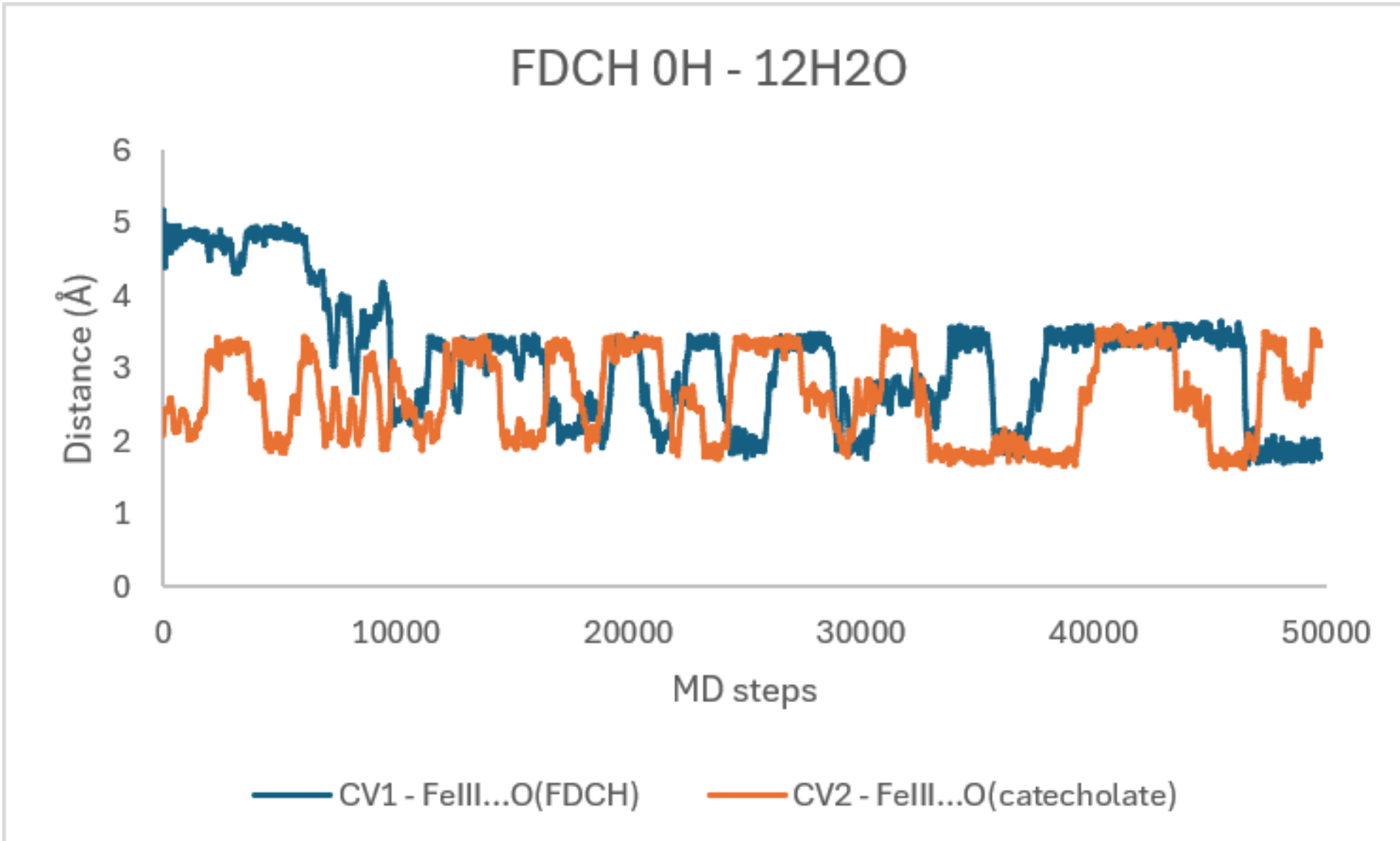
FDCH 0H - 12H2O
Distance (Å)
MD steps
0
1
2
3
4
5
6
0
10000
20000
30000
40000
50000
CV1 - FeIII...O(FDCH)
CV2 - FeIII...O(catecholate)

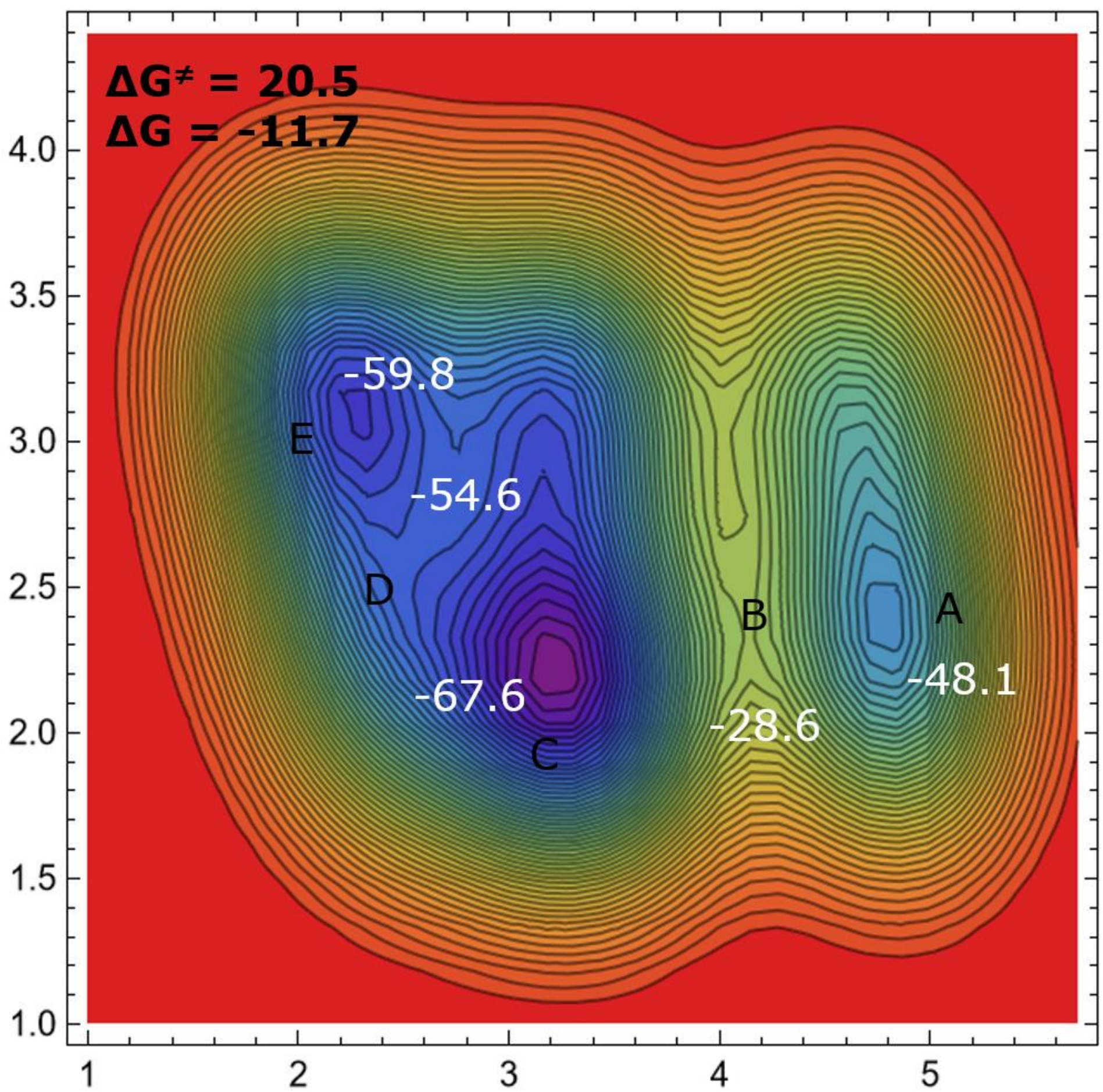
ΔG≠ = 20.5
ΔG = -11.7
-59.8
E
-54.6
D
-67.6
C
B
-28.6
A
-48.1
4.0
3.5
3.0
2.5
2.0
1.5
1.0
1
2
3
4
5

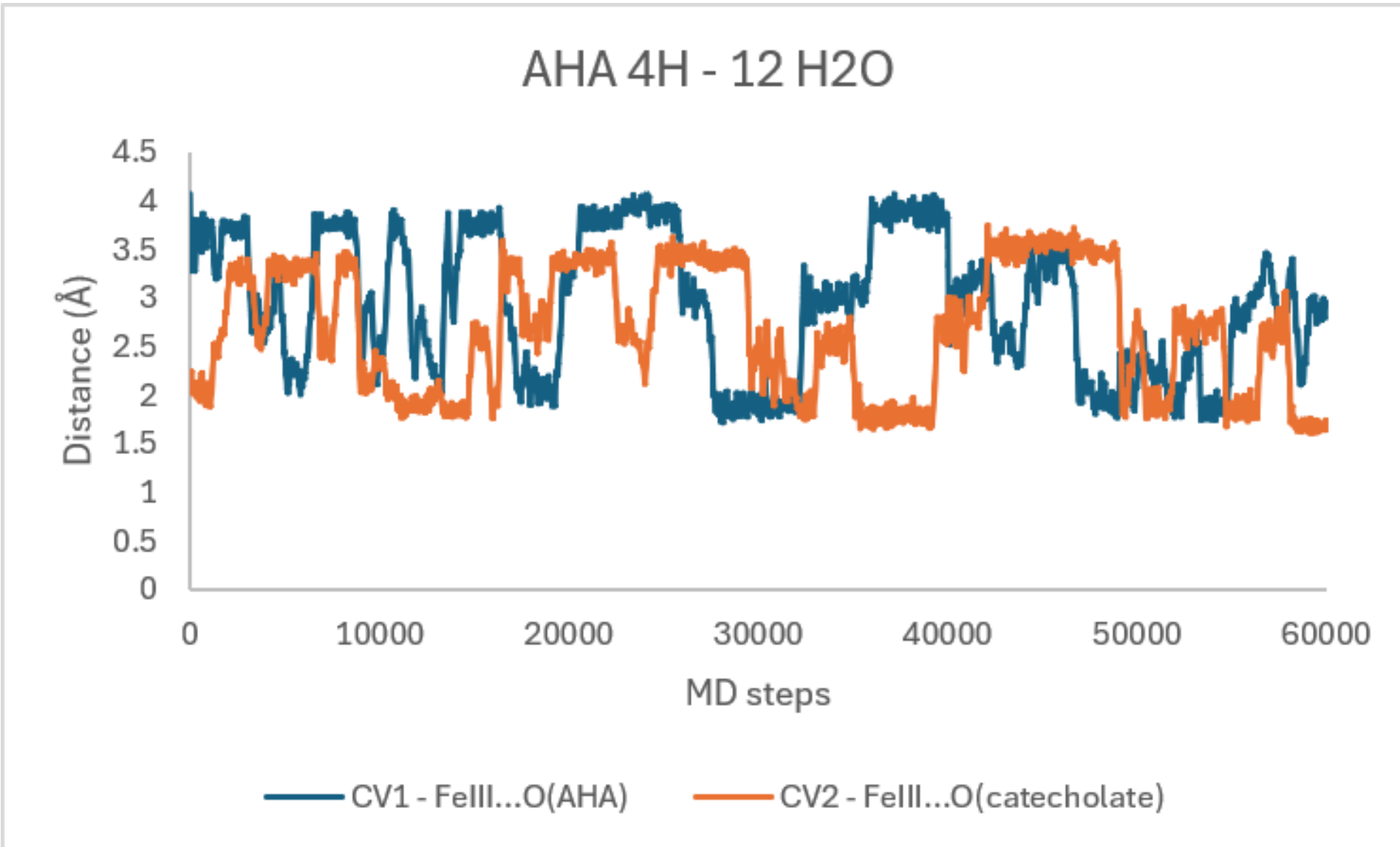
AHA 4H - 12 H2O
Distance (Å)
MD steps
CV1 - FeIII...O(AHA)
CV2 - FeIII...O(catecholate)

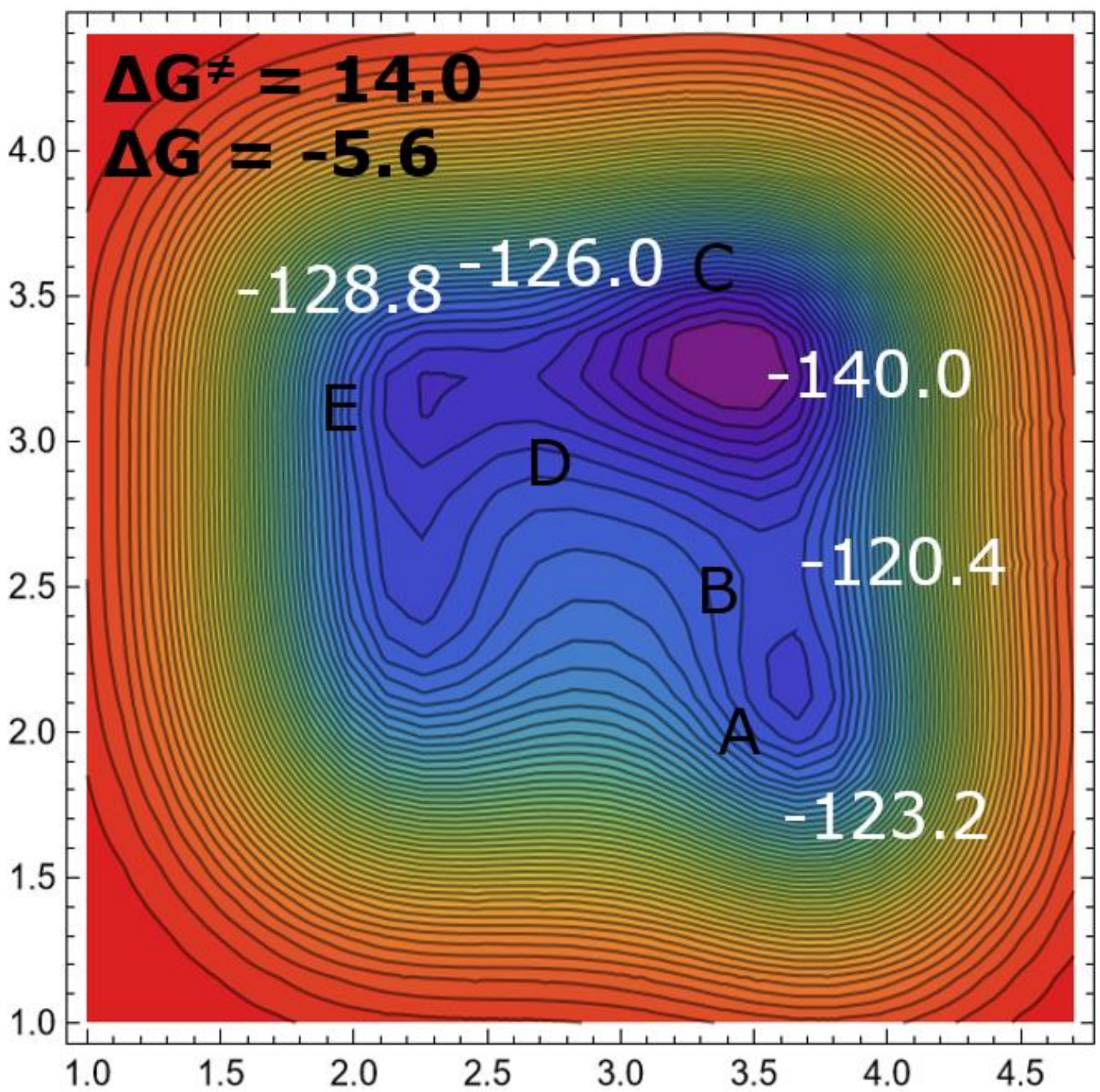
ΔG≠ = 14.0
ΔG = -5.6
-128.8
-126.0
C
-140.0
E
D
-120.4
B
A
-123.2

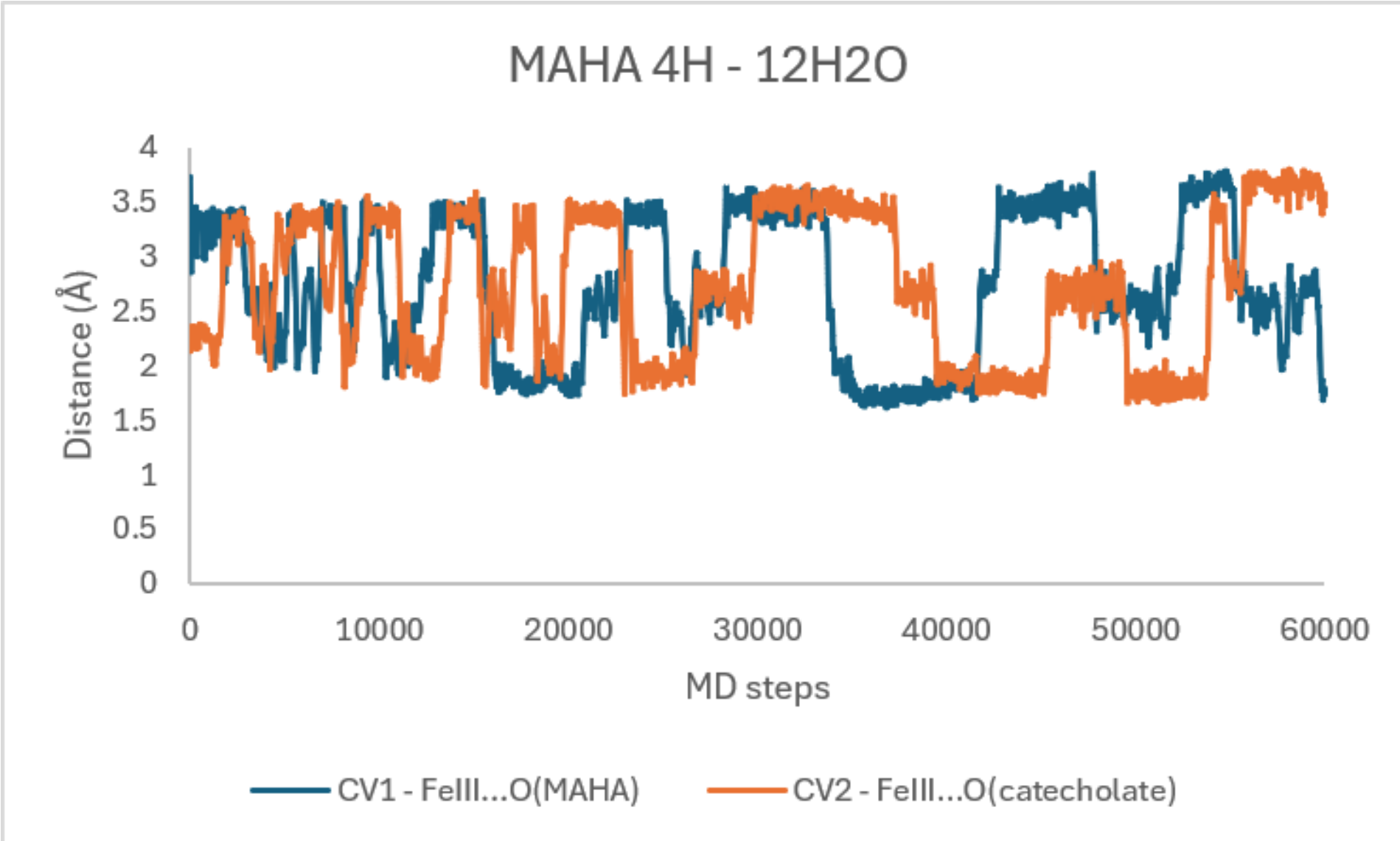
MAHA 4H - 12H2O
Distance (Å)
MD steps
CV1 - FeIII...O(MAHA)
CV2 - FeIII...O(catecholate)

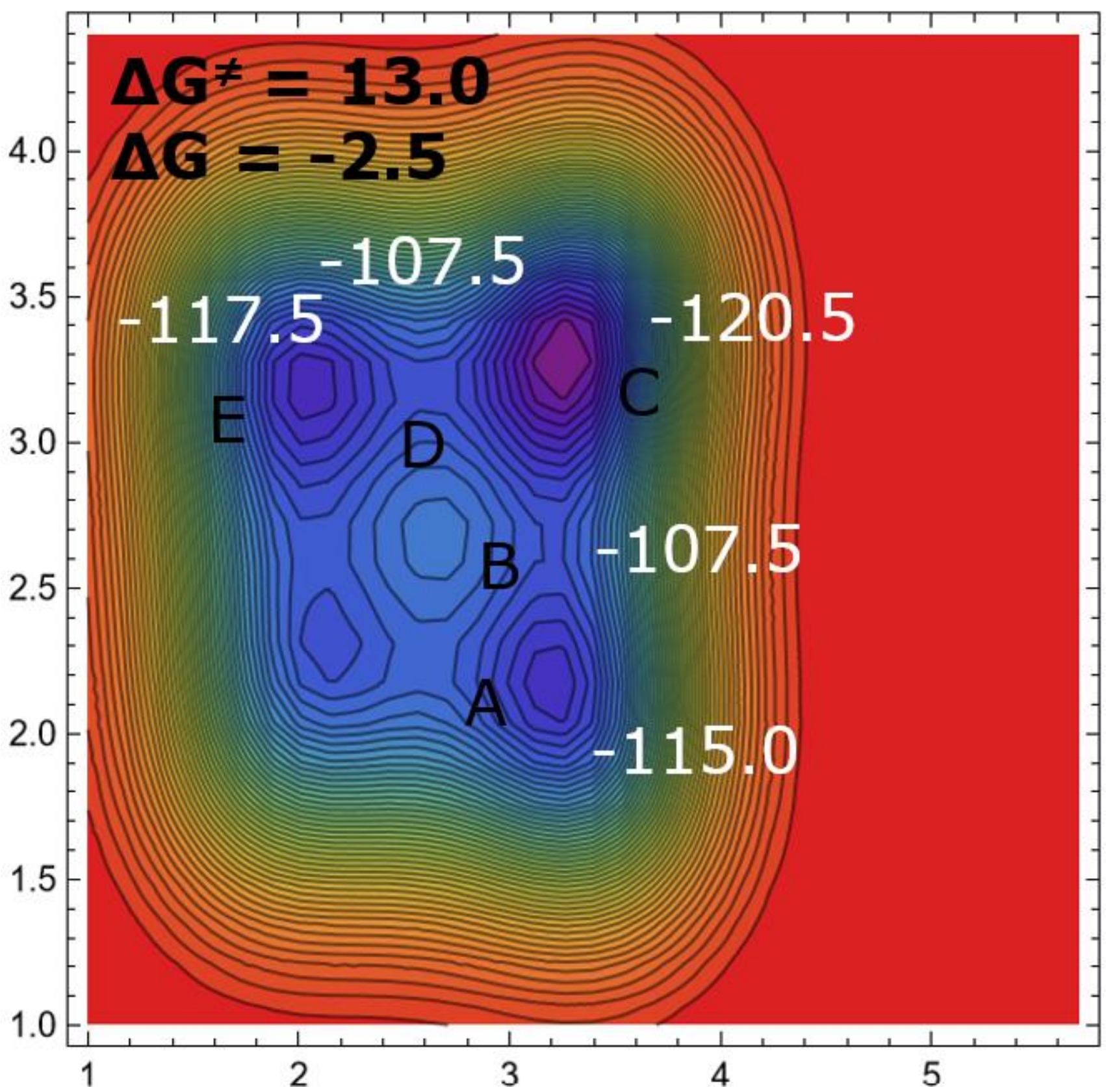
ΔG≠ = 13.0
ΔG = -2.5
-107.5
-117.5
-120.5
E
D
C
B
-107.5
A
-115.0

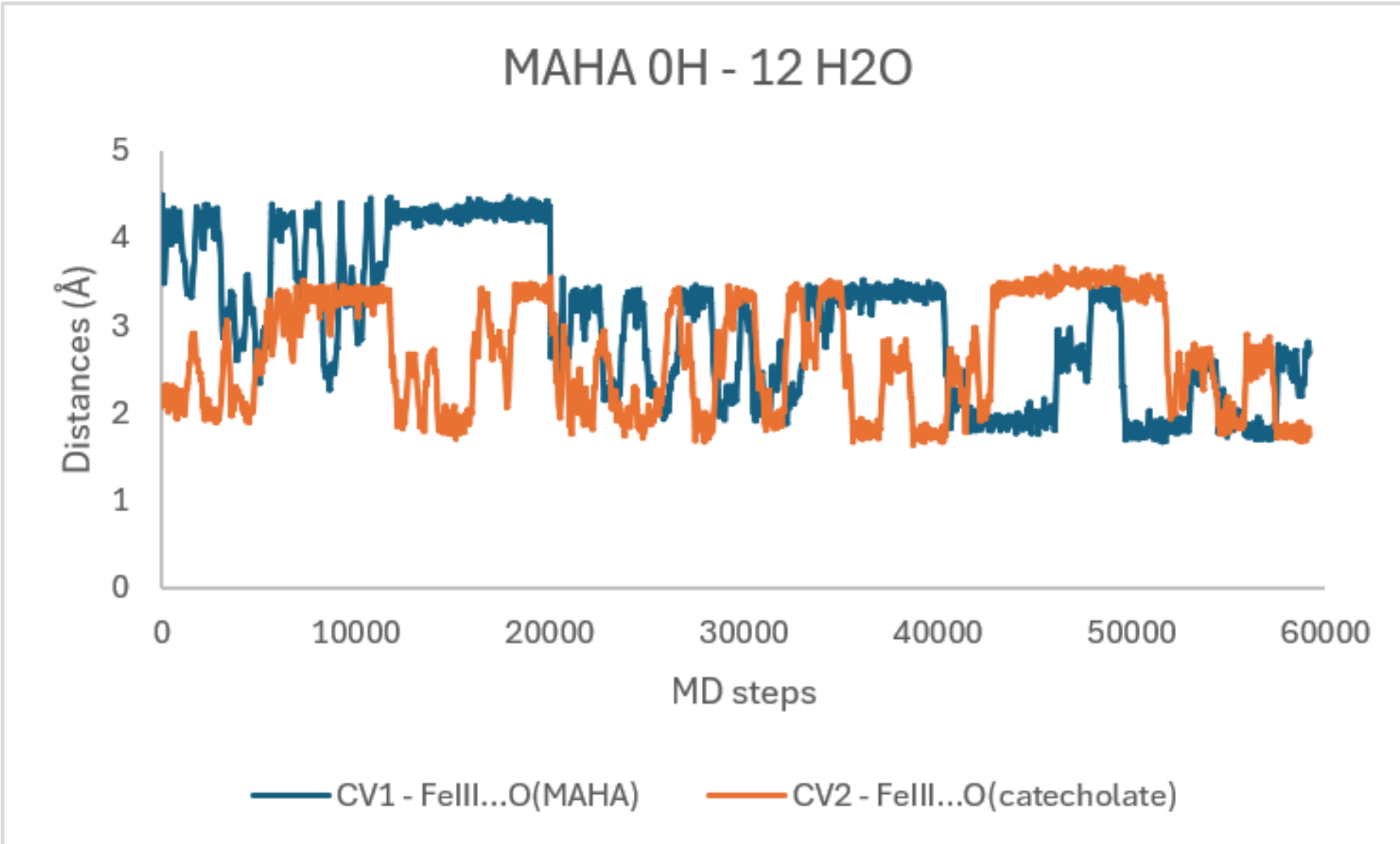
MAHA 0H - 12 H2O
Distances (Å)
0
1
2
3
4
5
0
10000
20000
30000
40000
50000
60000
MD steps
CV1 - FeIII...O(MAHA)
CV2 - FeIII...O(catecholate)

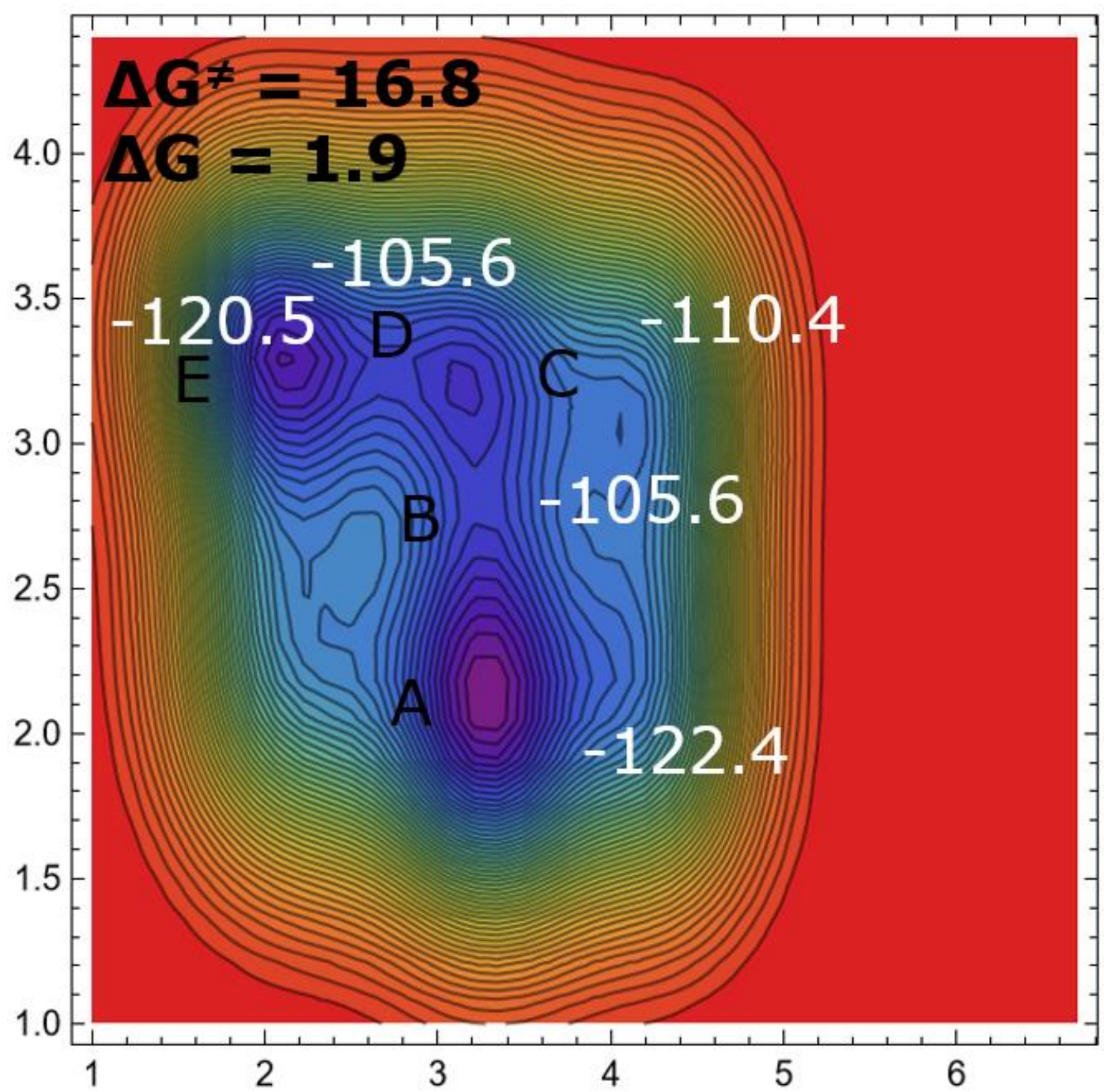
ΔG≠ = 16.8
ΔG = 1.9
-105.6
-120.5
D
E
C
-110.4
B
-105.6
A
-122.4
4.0
3.5
3.0
2.5
2.0
1.5
1.0
1
2
3
4
5
6

Explicit solvation

The reported FESs and the CVs variation in the main text are related to the molecular complexes in isolated-cluster model without explicit solvent in order to track only the ligands ranking. A new set of independent simulations with 12 explicit water molecules in each system at BLYP-D3 DZVP-MOLOPT was calculated. In the protonated systems the protons interact with water oxygens and several proton-transfers from catecholate and ligands to water molecules coupled with the main CVs can be detected (Figure S4 in SI). The aqueous environment plays a critical role in determining hydrogen-bonding patterns, proton-transfer events, and the resulting reaction energetics. The explicit water molecules were included to capture local hydrogen-bonding interactions, solvent-assisted structural rearrangements, and possible proton-transfer events occurring in the immediate vicinity of the reacting complex. Indeed, spontaneous proton-transfer events were observed in several trajectories of the fully protonated systems, highlighting the importance of local solvent organization. These observations indicate that proton motion can couple naturally to the reaction coordinate within the adopted simulation model. Sodium ions were included as counterions to maintain overall charge neutrality in the absence of protons. The simulations were conducted without periodic boundary conditions, and electrostatic interactions were treated using the nonperiodic Poisson solver implemented in CP2K. A sufficiently large simulation cell of 25 Å was employed to eliminate finite-size effects. Because protonation and deprotonation alter the net charge of the system, explicit Na^+ counterions were introduced to maintain charge neutrality where appropriate. The role of the counterions is primarily electrostatic. Nevertheless, we acknowledge that alternative counterion distributions may influence local electrostatic environments and consequently affect quantitative barrier heights, which is again for all the investigated systems. The present model provides only a partial representation of the aqueous environment.

While the 12 water molecules provide an explicit local microsolvation environment and enable local hydrogen-bonding and proton-transfer events, the finite number of water molecules and finite simulation cell may not fully capture long-range solvent fluctuations, ionic-strength effects, or the complete dielectric response of bulk water. Consequently, the calculated free-energy barriers should be interpreted as characterizing the relative behavior within the chosen model system rather than as quantitatively exact predictions under all experimental solution conditions. Nevertheless, because all systems were simulated using an identical solvent representation, the relative trends identified in this work are expected to be more robust than the absolute free-energy values.

Figure S4. The variation of proton…OH2 distances (unbiased) alongside the MTD trajectories with 12 explicit water molecules. The protons (H^+) are bound to the catecholate and the external ligands in the initial optimized geometry and move to the H2O molecules during the simulations.

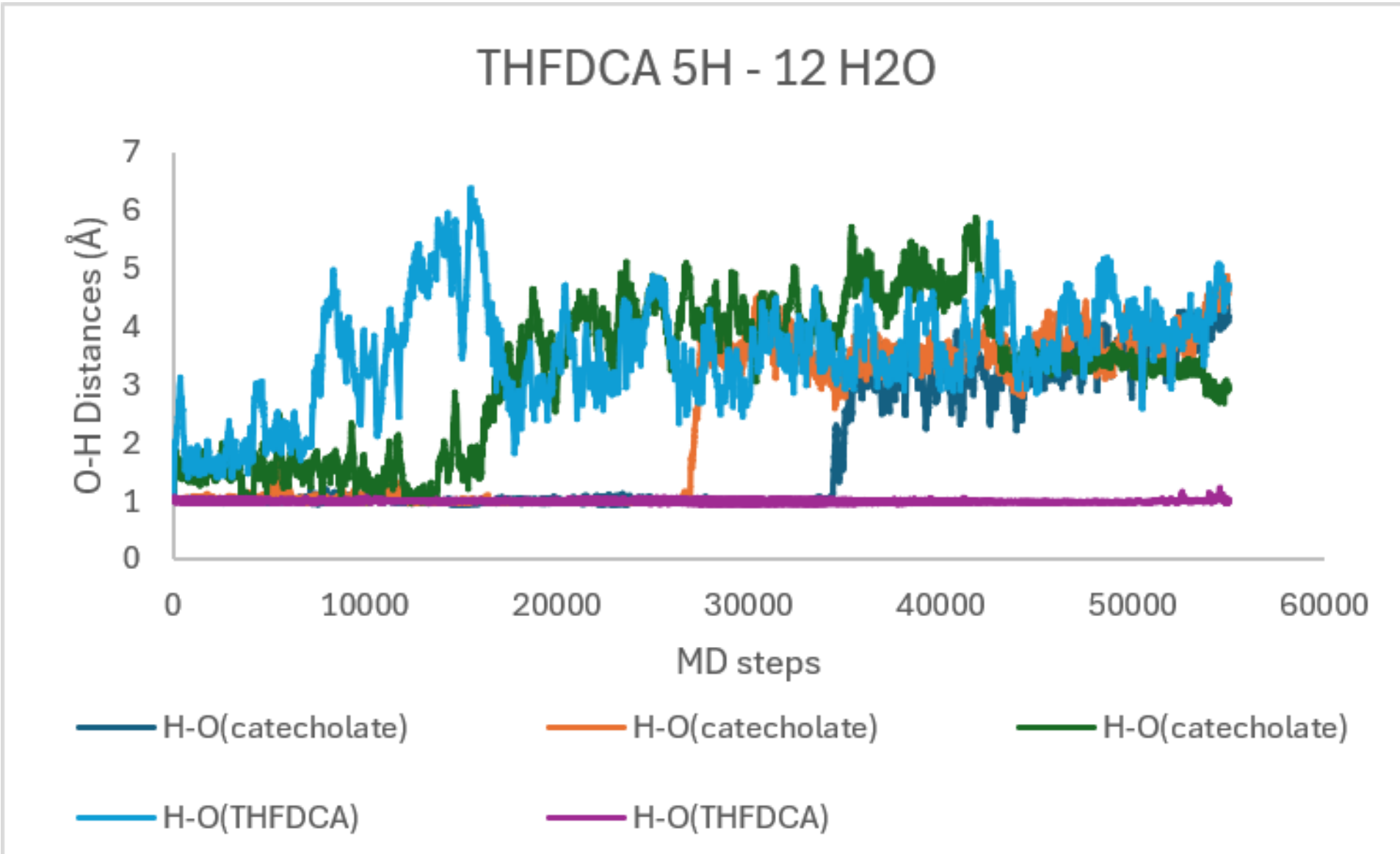

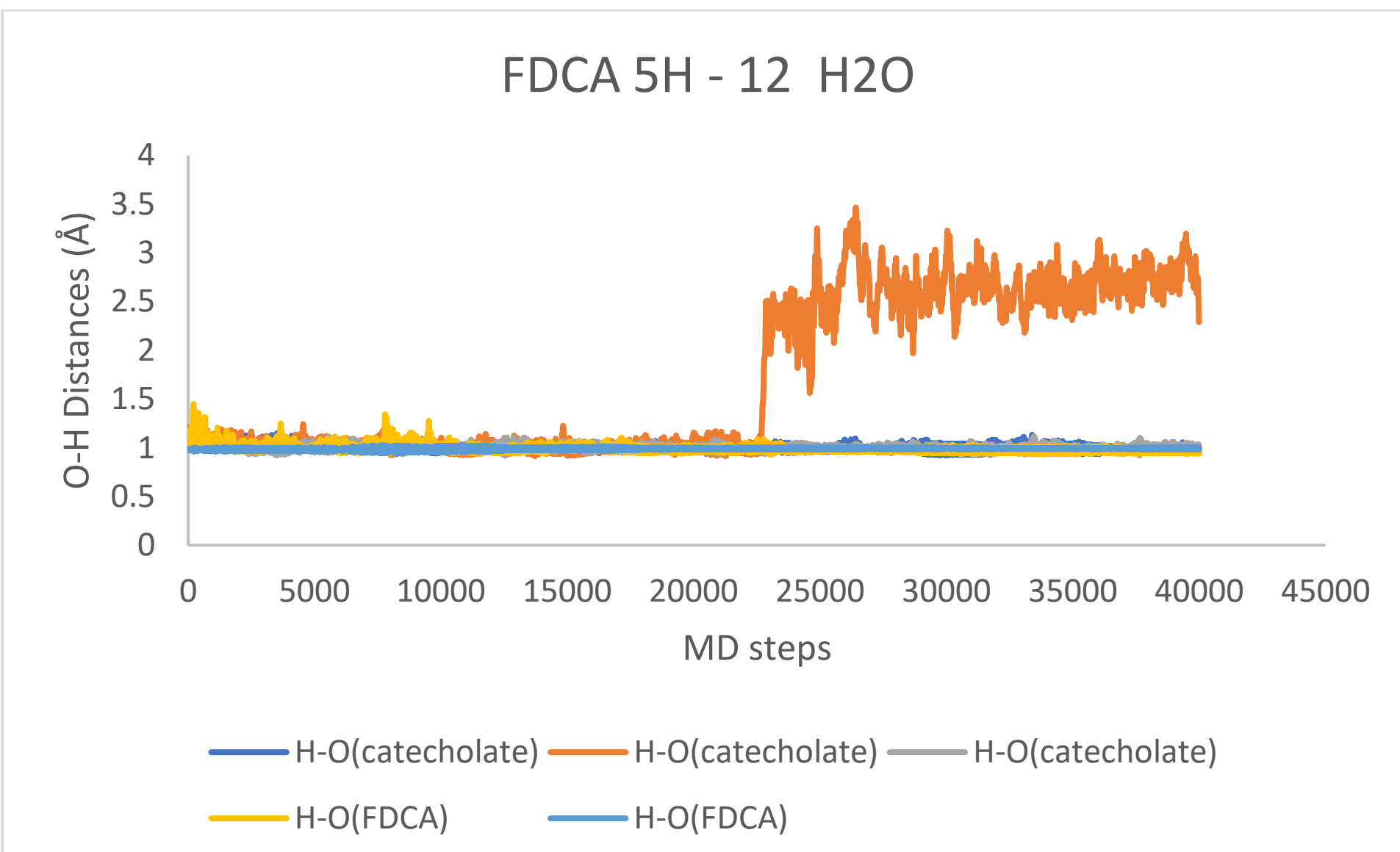

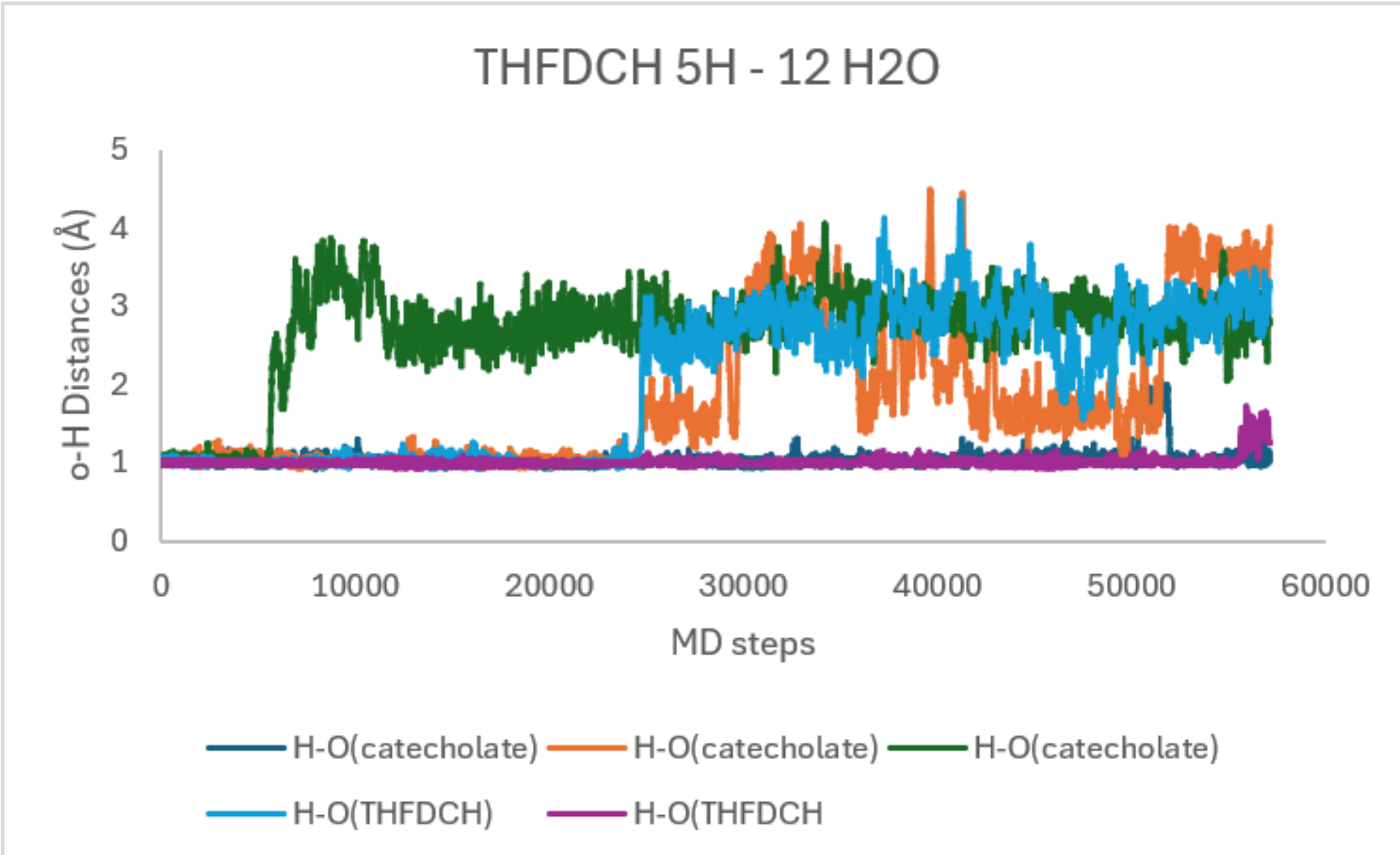

THFDCH 5H - 12 H2O
o-H Distances (Å)
5
4
3
2
1
0
0
10000
20000
30000
40000
50000
60000
MD steps
H-O(catecholate)
H-O(catecholate)
H-O(catecholate)
H-O(THFDCH)
H-O(THFDCH

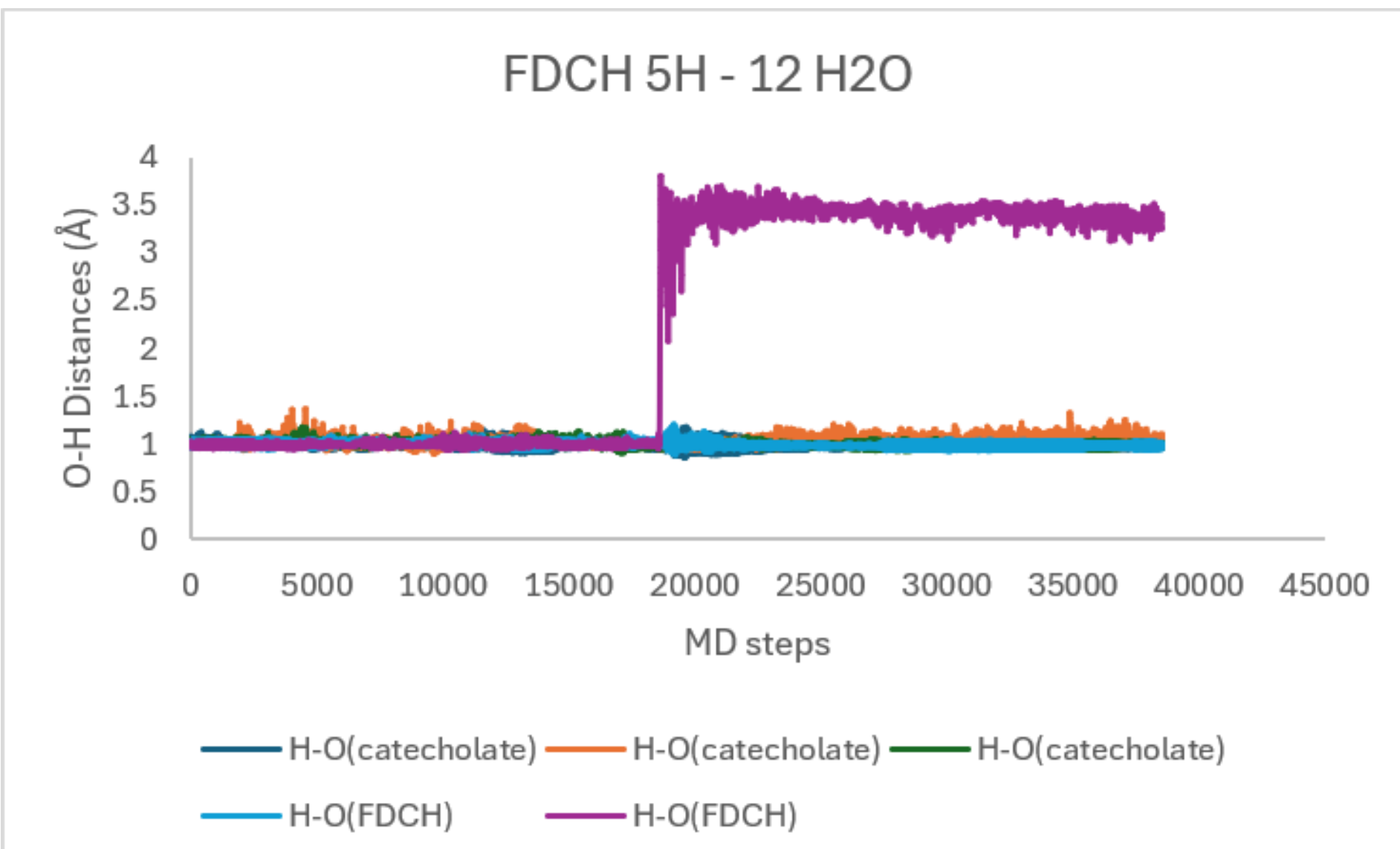

FDCH 5H - 12 H2O
O-H Distances (Å)
4
3.5
3
2.5
2
1.5
1
0.5
0
0
5000
10000
15000
20000
25000
30000
35000
40000
45000
MD steps
H-O(catecholate)
H-O(catecholate)
H-O(catecholate)
H-O(FDCH)
H-O(FDCH)

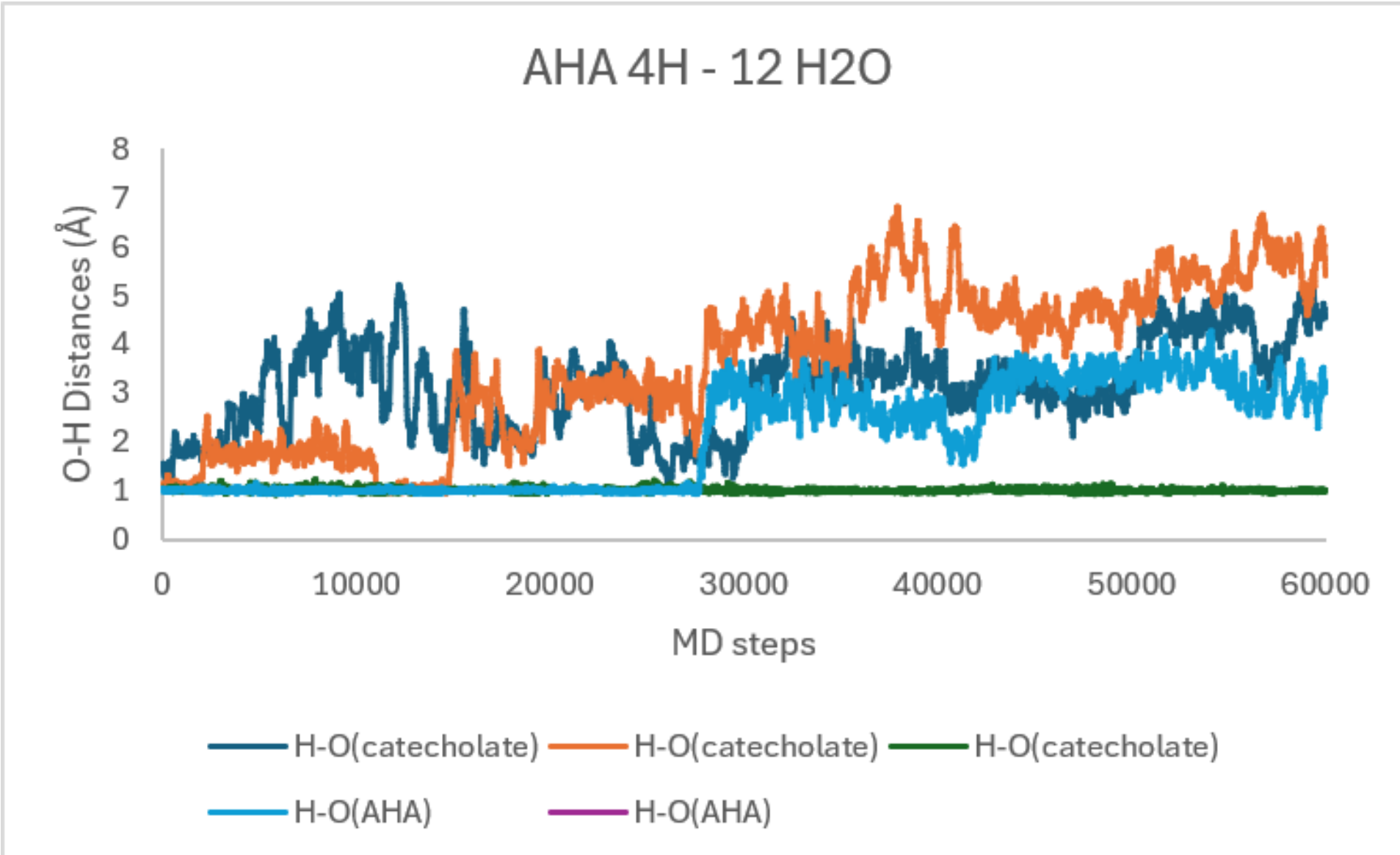
AHA 4H - 12 H2O
O-H Distances (Å)
0
1
2
3
4
5
6
7
8
0
10000
20000
30000
40000
50000
60000
MD steps
H-O(catecholate)
H-O(catecholate)
H-O(catecholate)
H-O(AHA)
H-O(AHA)

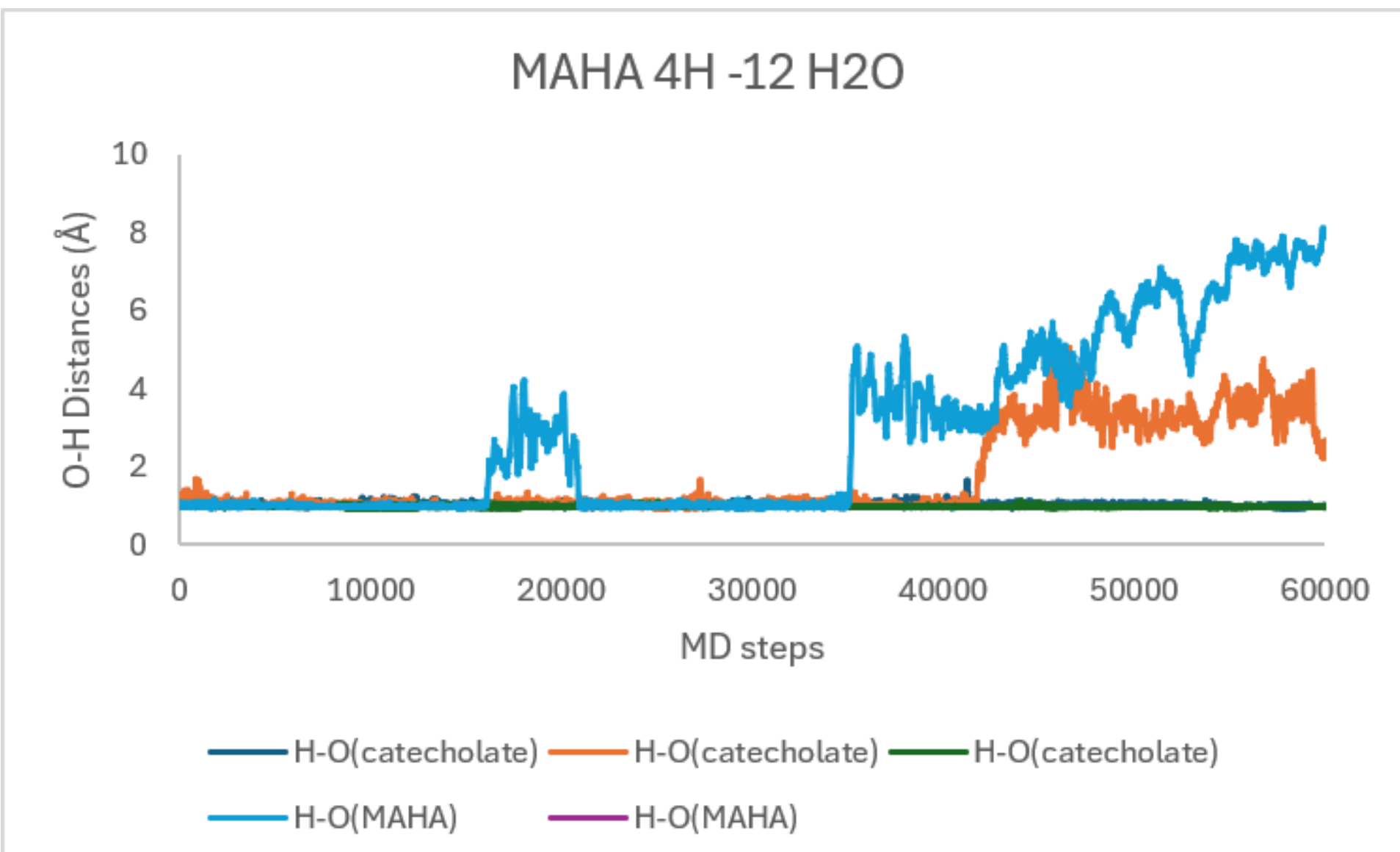
MAHA 4H -12 H2O
O-H Distances (Å)
0
2
4
6
8
10
0
10000
20000
30000
40000
50000
60000
MD steps
H-O(catecholate)
H-O(catecholate)
H-O(catecholate)
H-O(MAHA)
H-O(MAHA)

XYZ coordinates of the considered molecular complexes in the main text optimized at PBE-D3, DZVP-MOLOPT and gas phase

OPT-THFDCA-Five-H

C	0.7466357735	0.7980212418	1.5285137807
C	0.9287016049	1.3397402472	0.2600923082
C	1.2862486606	0.5397292680	-0.8201922634
C	0.9347353350	-0.5667113257	1.7373135518
C	1.4600822940	-0.8289534688	-0.6279750796
C	1.2863627919	-1.3438999915	0.6555387737
C	-3.1216297278	-2.9669603861	2.7369723746
C	-3.0613588821	-4.4925006708	2.8037879825
C	-2.5471974231	-4.8750915640	1.4215830813
C	-3.4745210720	-2.7156942884	1.2675238191
C	-1.0325349275	-4.8581537593	1.3086468770
C	-2.8855640673	-1.4070013223	0.7681638662
C	3.9395205595	-4.6206936701	-0.0364313345
C	2.8793406411	-5.5039304857	0.1657786383
C	5.1860240405	-4.8000584930	0.5243184384
C	5.3910690384	-5.9120643708	1.3390911118
C	4.3528248475	-6.8120591099	1.5527229275
C	3.1033585843	-6.6186623909	0.9726350616
C	0.2822989859	-4.2224398325	-3.3564533907
C	-0.6571391114	-3.8790865920	-2.3885489939
C	-0.0568512709	-4.6384757073	-4.6261501611
C	-2.0085759854	-3.9849982277	-2.7216380180
C	-1.4078982131	-4.7395014359	-4.9468846436
C	-2.3685772483	-4.4147911062	-3.9928366636
H	0.4545145436	1.4318692704	2.3542535545
H	0.7787339692	2.4004656343	0.1061662689
H	0.7990090376	-1.0190270826	2.7107604738
H	1.4108886337	0.9487134489	-1.8134757596
H	-4.0565044114	-4.9190601251	2.9434824889
H	-2.4133203406	-4.8559062043	3.6030534779
H	-2.1472709604	-2.5379460143	2.9684996890
H	-3.8636172244	-2.5304572743	3.4055926686
H	-2.9237703523	-5.8318876491	1.0571056731
H	-4.5569571143	-2.6383669331	1.1306421751
H	4.5153002422	-7.6779284052	2.1816790224
H	2.2914456004	-7.3138345632	1.1409698523
H	0.7190048890	-4.8745544173	-5.3425065453
H	-3.4188023393	-4.4962843705	-4.2426551384
H	-1.7046424411	-5.0662754408	-5.9337770980
H	-2.7501274243	-3.7468250872	-1.9712825712
H	6.3571632046	-6.0717559554	1.7972703615
H	5.9795037613	-4.0897255949	0.3304229900
O	1.5224777721	-2.7213228824	0.7321166144
O	1.7800609831	-1.6841015837	-1.6160450056
O	-3.0096951763	-3.8513088212	0.5301796988
O	-3.3584677373	-0.3613515561	1.1435540884
O	-1.8445604378	-1.4532637359	-0.0563983374
O	-0.3803500044	-3.9580073964	1.8410089632
O	-0.5464095790	-5.8148916502	0.5742722541
O	3.6230090699	-3.5648009962	-0.9091952450
O	1.7091207555	-5.2375690979	-0.4343835482
O	1.6306960652	-4.0942870491	-2.9397536547
O	-0.2332126787	-3.4783409142	-1.1807078990
Fe	1.5883426600	-3.4040746073	-1.0801907237
H	0.4136548187	-5.6185646825	0.2652234963
H	4.0040591977	-2.7281144244	-0.6112364148
H	2.0189373770	-4.9799879435	-2.8981263051
H	-1.5425418571	-2.3618644005	-0.2583416738
H	0.8056243085	-3.2155785663	1.2539398847

OPT-THFDCA-zero-H

C	1.4012845439	1.0242114191	1.6754413814
C	1.4384670466	1.6270178187	0.4233414120
C	1.3988158788	0.8494443663	-0.7458730008
C	1.3092633196	-0.3781851018	1.7701979183
C	1.3431914322	-0.5441210132	-0.6720294227
C	1.2844596280	-1.1605017688	0.6147170987
C	-4.5840695489	-2.1251704816	2.2887853617
C	-5.0100609759	-3.5612633555	2.5617454726
C	-3.8257504834	-4.3303908637	1.9888356792
C	-3.9743906278	-2.2506740517	0.8948908738
C	-2.7039194733	-4.5316229497	3.0511368652
C	-2.9857023512	-1.1422976007	0.5731278472
C	4.2511062227	-3.8910573607	-0.6103426837
C	3.4562583957	-5.0292186492	-0.2866517655
C	5.6423996700	-4.0009324259	-0.6255735947
C	6.2578401636	-5.2181144852	-0.3144664770
C	5.4847333671	-6.3259554839	0.0111561746
C	4.0883723354	-6.2390896514	0.0251132791
C	1.0902968558	-4.6341015030	-3.3003051213
C	-0.0807780739	-4.6322814119	-2.5109845291
C	1.1507573675	-5.4378893193	-4.4387982964
C	-1.1525274793	-5.4433506727	-2.8561022745
C	0.0672005983	-6.2564920439	-4.7766357061
C	-1.0733552861	-6.2600234490	-3.9882384467
H	1.4526518949	1.6236181760	2.5760778689
H	1.5250716515	2.7039170924	0.3433616084
H	1.2143437724	-0.8692123277	2.7348241107
H	1.4475181868	1.3147156442	-1.7231463709
H	-5.9427703830	-3.7969035879	2.0401620326
H	-5.1241593136	-3.7958735152	3.6200181240
H	-3.8145014253	-1.8187609029	3.0005194265
H	-5.3847428133	-1.3878931462	2.3163707653
H	-4.1063163665	-5.3204476364	1.6215150965
H	-4.7695119943	-2.1709966037	0.1391369851
H	5.9607335426	-7.2660651279	0.2586866946
H	3.4717831520	-7.0906969843	0.2899532967
H	2.0192197300	-5.3725918665	-5.0884667556
H	-1.9100538045	-6.8948380168	-4.2476550392
H	0.1188527410	-6.8760891559	-5.6619445049
H	-2.0386633907	-5.4375573258	-2.2317496794
H	7.3388086443	-5.2926726673	-0.3320302681
H	6.2282949361	-3.1307274958	-0.8982477625
O	1.2582728104	-2.4916197556	0.6274308609
O	1.3043116501	-1.3530721772	-1.7138231620
O	-3.4046039878	-3.5576014027	0.8222716917
O	-3.2992296415	0.0215988358	0.9072657729
O	-1.8880425978	-1.4296962168	-0.0191604478
O	-1.5311752936	-4.1759760385	2.7339461974
O	-3.0970874263	-5.0511060907	4.1028652376
O	3.5821944320	-2.7559636202	-0.8499207137
O	2.1417152700	-4.8792730452	-0.3712971587
O	2.1007394338	-3.8524594407	-2.8890466424
O	-0.0824106134	-3.7768860713	-1.4716446919
Fe	1.7253543327	-3.1960507051	-1.1390050332
Na	-1.1330918813	0.6618637211	0.8978714985
Na	3.4745198912	-2.3063887284	1.3684893967
Na	3.4751566589	-5.6635644392	-2.6968145914
Na	-0.9756529452	-1.7599384580	-1.9944121204
Na	-0.9745220831	-3.7848065858	0.6672649603

OPT-FDCA-Five-H

C	3.6747877922	0.3361950360	1.0929060613
C	3.8534841557	0.6241691957	-0.2565998879
C	3.4564816016	-0.2737006193	-1.2425049523
C	3.1000957786	-0.8748567475	1.4766741168
C	2.8680471903	-1.4825435053	-0.8737980355

C	2.7128014601	-1.7528618790	0.4872320962
C	-3.4002368516	-0.6101350367	2.4687082296
C	-2.1327624973	-1.1582660871	2.7794799412
C	-2.0432004276	-2.3367515091	2.0931047442
C	-3.9890629963	-1.4884400089	1.6030748478
C	-0.9384447028	-3.2787832101	1.9276383261
C	-5.2528993783	-1.4228062152	0.8576394638
C	2.8844308948	-6.0013683066	-0.1774595643
C	1.6027546259	-6.1040900826	0.3616796883
C	3.9147242152	-6.8510061803	0.1631286561
C	3.6641181750	-7.8500162006	1.1021106983
C	2.3943506428	-7.9755380382	1.6544999780
C	1.3635110204	-7.1148320985	1.2908012195
C	-0.6363333177	-3.5906390507	-2.7440864854
C	-0.8962120063	-2.7174609074	-1.6905914078
C	-1.4870316991	-3.7557387240	-3.8160025467
C	-2.0765189503	-1.9706647333	-1.7361242184
C	-2.6712192125	-3.0221398159	-3.8372337998
C	-2.9537085826	-2.1347669905	-2.8011401609
H	3.9826282966	1.0467579778	1.8475215954
H	4.3023443983	1.5650524382	-0.5479204208
H	2.9523233006	-1.1324436791	2.5172100719
H	3.5783580825	-0.0528182401	-2.2941803422
H	-1.3611831213	-0.7498935212	3.4119374669
H	-3.8362727319	0.3153884156	2.8084668472
H	2.2016624987	-8.7528821339	2.3827197423
H	-1.2285118050	-4.4407144147	-4.6129020054
H	-3.8718787194	-1.5606951385	-2.8225033937
H	-3.3613503980	-3.1369903154	-4.6614510631
H	-2.2823498256	-1.2778358103	-0.9314961496
H	4.4572802308	-8.5243136456	1.3937527820
H	4.8900222317	-6.7368464965	-0.2920465173
O	2.1722551507	-3.0175988534	0.7406127186
O	2.4447036520	-2.3990632746	-1.7575665597
O	-3.1762891050	-2.5492463382	1.3755889758
O	-6.0737897420	-0.5644806303	1.0552515481
O	-5.4296615642	-2.3730407398	-0.0830002559
O	0.1800978198	-2.9959396127	2.3626984664
O	-1.2660141813	-4.3686671000	1.2919341022
O	2.9990136851	-4.9808212970	-1.1364363631
O	0.6714485465	-5.2315691760	-0.0566466879
O	0.5939738386	-4.2864904918	-2.6305979045
O	-0.0308138182	-2.6397062143	-0.6788489072
Fe	1.4354020521	-3.6700000201	-0.9350190958
H	-0.4421382348	-4.8069338311	0.8617121940
H	-4.6490134087	-2.9434059674	-0.1428586103
H	0.3759817894	-7.2063551705	1.7234032164
H	0.4025729980	-5.2283971947	-2.5141454279
H	1.4506211397	-3.0017677833	1.4465325533
H	3.8447965338	-4.5184149492	-1.0711714318

OPT-FDCA-Zero-H

C	0.7307730214	0.4482722135	1.3036976230
C	0.6686736377	0.9875771585	0.0257045029
C	0.9465871093	0.1915323050	-1.0865830901
C	1.0813583737	-0.8884151830	1.4882951335
C	1.2868197191	-1.1469081096	-0.9096596964
C	1.3568546983	-1.6986444665	0.3881661112
C	-1.3678998982	-2.3366477857	4.1371748596
C	-1.0988657677	-3.7215421417	3.9488607765
C	-1.5010049380	-4.0277463238	2.6770224933
C	-1.8935929258	-1.8714353689	2.9674942689
C	-1.4257615896	-5.2566560977	1.8469656581
C	-2.2179052189	-0.4819268160	2.4640949931
C	3.8762962428	-4.8130096829	-0.4588260444
C	2.8717274576	-5.6929166125	0.0221099073
C	5.1535722534	-4.8698015064	0.0812192394
C	5.4455900519	-5.7740820311	1.1064307819
C	4.4622101024	-6.6261932001	1.5856213391
C	3.1736601715	-6.5911251066	1.0434527518
C	-0.0228357484	-4.6816428770	-3.2959649936
C	-0.9070764421	-4.3207795487	-2.2554137956
C	-0.5314227905	-5.2488453324	-4.4600805581
C	-2.2707726594	-4.5756020605	-2.3790475399
C	-1.9020885575	-5.4774716893	-4.5879446553
C	-2.7660364538	-5.1498777180	-3.5512132145
H	0.4427141670	1.0434715101	2.1608067861
H	0.3617527556	2.0157377201	-0.1110574992
H	1.0940653215	-1.3116543358	2.4864476660
H	0.8722031717	0.5831544437	-2.0942030197
H	-0.7057563634	-4.4293228169	4.6631222990
H	-1.1979075686	-1.7274009083	5.0093941118
H	4.6885512800	-7.3251839013	2.3801895244
H	0.1530258372	-5.5027593155	-5.2604875055
H	-3.8267225207	-5.3412207076	-3.6443097345
H	-2.2885670382	-5.9183953973	-5.4971901755
H	-2.9242960589	-4.3464728912	-1.5444735786
H	6.4414437274	-5.8021694909	1.5281443322
H	5.9159742519	-4.1981532129	-0.2952297128
O	1.6729955328	-3.0015912055	0.4578197667
O	1.5863707741	-1.9863332270	-1.9306255301
O	-1.9881993349	-2.9074374893	2.0772976968
O	-2.1429425631	0.4111740222	3.3147456037
O	-2.4727932698	-0.4147815525	1.2264916523
O	-0.4070053518	-6.0034433687	2.0630634067
O	-2.2671559482	-5.4324527790	0.9456391768
O	3.5036967354	-3.9937614847	-1.4703556900
O	1.6653800435	-5.5969207516	-0.5648592647
O	1.2879272322	-4.4867186414	-3.0705362205
O	-0.3575018318	-3.7969537590	-1.1373518495
Fe	1.5136579938	-3.7617973622	-1.3064521529
Na	-1.4455849269	-1.8399109787	-0.1191603031
H	2.3955529297	-7.2554425354	1.4034898141
Na	2.9274364285	-5.8973471279	-2.7710410526
Na	1.2139199077	-4.4148726765	2.1462341159
Na	3.7873482606	-1.8384799317	-2.0680161442
Na	-0.5844460022	-6.4386157460	-0.2744463752

OPT-THFDCH-Five-H

C	-0.9149158790	-1.7636631887	-0.7236882471
C	-0.7131415250	-1.9756274497	0.6436830243
C	-0.1094978996	-3.1570481980	1.0942740010
C	-0.5159624637	-2.7302808604	-1.6560127718
C	0.2947068027	-4.1114976201	0.1638988233
C	0.0929757305	-3.8987642848	-1.2047799610
C	7.8607259928	-7.2608964734	-3.3277873204
C	7.3971492445	-8.6710386616	-2.9134472628
C	6.2699866758	-8.3549664697	-1.9343216042
C	6.5931550217	-6.3926790793	-3.1556922570
C	5.2552252179	-9.4764444743	-1.7393995138
C	6.0233587536	-5.8785173982	-4.4758374415
H	-1.3837530392	-0.8443222799	-1.0715523310
H	-1.0247185514	-1.2227708245	1.3662657907
H	-0.6655260587	-2.5778759546	-2.7245215607
H	0.0539923277	-3.3346588707	2.1568836430
H	4.0755901928	-8.3823526451	-0.3812904869
H	4.0134639189	-5.8871827946	-3.7887767367
O	0.5117402817	-4.9301707145	-2.0554193703
O	0.8987120131	-5.3273186071	0.5080714860
O	5.5831035943	-7.2261957297	-2.5220458588
O	5.1884527336	-10.5073699692	-2.4368754339
O	6.6868481275	-5.7425078338	-5.5277311692
O	3.4163139769	-10.3001301529	-0.5769107018
O	4.1742850500	-5.1830960063	-5.7012649972
Fe	2.1877849269	-6.0209853930	-1.1994697528
N	4.4212839813	-9.3186821029	-0.6711927008
N	4.7234381897	-5.5039890562	-4.4425386732
H	1.4602235692	-5.2171431055	1.3004068435
O	3.1264725544	-4.4192706056	-1.2327121243
O	3.4778832137	-6.6640563691	-0.0467601850
O	2.5873216992	-6.5337915349	-2.9343127255
O	1.1869742812	-7.8415638108	-1.1360796751

C	4.3342416283	-4.5724654049	-0.6581089419
C	4.5366094701	-5.8048693231	0.0115562615
C	5.3551474304	-3.6085892468	-0.6786675496
C	6.5547699164	-3.8861789487	-0.0128495605
C	6.7429620454	-5.1032722142	0.6671289257
C	5.7336016049	-6.0745404523	0.6845128838
H	5.1961047016	-2.6710081179	-1.2091342111
H	7.3547822109	-3.1466516504	-0.0223049094
H	7.6818136304	-5.2944430563	1.1847866666
H	5.8595721830	-7.0222117909	1.2078928271
C	2.2427597445	-7.8070041524	-3.2513159090
C	1.4397831153	-8.5296645842	-2.3587193668
C	2.6266761152	-8.4234815286	-4.4551873749
C	0.9988408027	-9.8202013058	-2.6134523366
C	2.1838484040	-9.7201573638	-4.7305107115
C	1.3697951943	-10.4170565892	-3.8266689266
H	3.2533076658	-7.8722559728	-5.1551062552
H	2.4850218343	-10.1970394342	-5.6626600744
H	0.4022729751	-10.3527295422	-1.8740072535
H	1.0390465478	-11.4289045681	-4.0534815522
H	0.8745394503	-4.5643003014	-2.8883580067
H	8.2264468032	-7.2086985628	-4.3617923760
H	8.6552980301	-6.8956317052	-2.6641192108
H	6.7872109261	-5.5240847602	-2.5010930758
H	8.1953702823	-9.2731181882	-2.4592118792
H	6.9816786890	-9.2346071694	-3.7595407924
H	6.6739522089	-8.0563931676	-0.9488943702
H	3.6610740003	-10.8531957933	-1.3724436166
H	0.2327237925	-7.8530904758	-0.9360504532
H	5.0084615697	-5.2435009887	-6.2486693063

OPT-THFDCH-zero-H

C	-1.6596517724	-1.6234647775	-0.9343911827
C	-1.2169256840	-1.6931883839	0.3939312174
C	0.0137752794	-2.3170694662	0.6935417753
C	-0.8867023291	-2.1730496503	-1.9759814118
C	0.7991444280	-2.8707965064	-0.3329756507
C	0.3436283553	-2.8054417770	-1.6995091248
C	6.6218897586	-8.5720589621	-5.3665683219
C	6.5000680525	-9.9433495096	-4.6616892385
C	6.9808348275	-9.6820834388	-3.2204130892
C	7.4437029330	-7.6834431884	-4.4144291716
C	5.8822214636	-9.4041071195	-2.1985378164
C	6.6620298858	-6.4560762121	-3.9273611815
H	-2.6098785688	-1.1437865340	-1.1670051431
H	-1.8166691698	-1.2660831055	1.1968455896
H	-1.2280264266	-2.1389065627	-3.0112761559
H	0.3648345333	-2.3906656965	1.7251250103
H	4.3388534239	-10.2642603521	-1.2433372513
H	6.8381279863	-4.7380653979	-2.8828600221
O	1.0842536334	-3.4192027077	-2.6181085370
O	1.9341488022	-3.5591348855	-0.1255813683
O	7.8801270973	-8.5530620145	-3.3114709362
O	5.8200681564	-8.2887748640	-1.5187076004
O	5.4056147645	-6.2593591355	-4.1881026575
O	5.0462784427	-11.5877467624	-2.6122788126
O	8.6315881978	-5.6764687119	-2.7847744603
Fe	2.2049738494	-4.6689705737	-1.7310752728
N	5.0330413705	-10.4168786359	-1.9974387083
N	7.3624060502	-5.5631553230	-3.2200491279
Na	3.8663136023	-3.3190025145	0.9002071531
O	3.8539602242	-3.7039914959	-2.2528049981
O	3.4664262008	-5.8247642758	-0.7367550466
O	2.3154804936	-5.7735199283	-3.3281219683
O	0.6760975304	-5.7510398815	-1.2641628826
C	4.8456752494	-4.0256343401	-1.4099475749
C	4.6397799629	-5.1996466633	-0.6004315069
C	6.0254676719	-3.2707971469	-1.2475460523
C	7.0079553150	-3.6674812589	-0.3147457870
C	6.8073811930	-4.8074878744	0.4731875796
C	5.6282517441	-5.5732098843	0.3357813743
H	6.1614579863	-2.3752771309	-1.8568609015
H	7.9216131908	-3.0835578143	-0.2143317980
H	7.5544550731	-5.0946267999	1.2149551171
H	5.4572153153	-6.4721138549	0.9308625629
C	1.1495371467	-6.4565151155	-3.4740693389
C	0.2810540846	-6.4618869153	-2.3461789087
C	0.8070230750	-7.1793045358	-4.6231820339
C	-0.9040922212	-7.2130700403	-2.3943780390
C	-0.3926009861	-7.9087232416	-4.6642661656
C	-1.2444214315	-7.9276873241	-3.5547404712
H	1.4840674735	-7.1662214960	-5.4785693730
H	-0.6537734199	-8.4662523885	-5.5622870930
H	-1.5371236857	-7.2762228934	-1.5039571177
H	-2.1665278352	-8.5057540071	-3.5827424595
Na	-1.1315931362	-4.6478476528	-0.4316130380
Na	3.9506881745	-7.3066897842	-2.5368653818
Na	7.9249318739	-7.2273580900	-1.2921596861
Na	3.9427202054	-4.5701334481	-4.4111623724
H	5.6408198394	-8.1094867562	-5.5324323899
H	7.1198099816	-8.6649080794	-6.3401547702
H	8.3836260746	-7.3086286299	-4.8465498281
H	7.1591596021	-10.6857354885	-5.1306543296
H	5.4875772775	-10.3662385877	-4.6613180080
H	7.5726359855	-10.5376983281	-2.8526743212

OPT-FDCH-Five-H

C	5.2456620138	-4.0543381941	-3.9474528409
C	5.6696637739	-3.3385412637	-2.8321530652
C	4.8581642021	-3.2834982883	-1.7021733165
C	4.0266840569	-4.7206668725	-3.9490640654
C	3.6371953564	-3.9271627034	-1.7266084333
C	3.1874090685	-4.6665523503	-2.8297223119
C	6.5903563093	-8.2092210753	-4.8347453668
C	7.6405889004	-7.4384352331	-5.3787366422
C	7.9123916326	-6.4544184799	-4.4644574697
C	6.2849440607	-7.6372879783	-3.6287003178
C	8.6897782813	-5.2348514024	-4.6479769426
C	5.1279460702	-7.8590301291	-2.7600840227
C	2.6892481575	-7.0357650385	0.9374307458
C	1.6446720643	-6.5285633791	1.7123796992
C	3.6479333452	-7.8250999914	1.5822853950
C	3.5283596515	-8.0815277714	2.9425169211
C	2.4675731472	-7.5720176285	3.6874567255
C	1.5046157808	-6.7849766091	3.0602177237
C	-1.1814032517	-6.3309296103	-2.0006133177
C	-1.1918179579	-4.9436484538	-2.0998516766
C	-2.3426016922	-7.0582480238	-2.1956086090
C	-2.3667227915	-4.2759834199	-2.3942473690
C	-3.5350229954	-6.3888875572	-2.4829438881
C	-3.5458852973	-4.9987899562	-2.5784373167
H	5.8803155461	-4.1132764639	-4.8233816746
H	6.6290010941	-2.8419959371	-2.8260155453
H	3.7016667689	-5.2987673782	-4.8039774437
H	5.1689738522	-2.7584912041	-0.8080829329
H	9.1800828058	-3.4606664538	-3.7546189358
H	5.5048385574	-6.0966886844	-1.7042525602
H	8.1187663013	-7.5481701435	-6.3378150594
H	6.0830315399	-9.0568361524	-5.2645733004
H	4.4828152060	-8.2010558371	0.9968606047
H	4.2801655702	-8.6889717045	3.4304113015
H	2.3859217442	-7.7831157527	4.7443633993
H	0.6627253005	-6.3812660332	3.6084863285
H	-2.3064452717	-8.1346479934	-2.0927108033
H	-4.4563304191	-6.9460930827	-2.6161210256
H	-2.3410936501	-3.1971521637	-2.4634311277
H	-4.4768574195	-4.4824230760	-2.7874421562
O	2.0138844048	-5.2906347458	-2.7731449243
O	2.7802927963	-3.9213867795	-0.6040363568
O	7.0929213027	-6.5822342285	-3.3857912367
O	8.9879541307	-4.8076403069	-5.7507619764
O	4.2374488512	-8.6369595027	-3.0158966264

O	8.9330999945	-4.8027283636	-2.3169681252
O	5.9808326876	-7.8791364114	-0.8838042414
O	2.7350443485	-6.7429754810	-0.3614249186
O	0.7186720331	-5.7418019014	0.9842374218
O	0.0084997071	-6.9382602466	-1.6035154756
O	-0.0253817024	-4.2358099263	-1.8474199666
Fe	1.4045017450	-5.5000257035	-0.9247929386
N	8.9492487717	-4.4302674320	-3.5271357683
N	5.1162891928	-7.0376604069	-1.5705750183
H	0.4043993489	-5.0145135086	1.5300265048
H	4.1527649027	-6.9748259356	-1.1386321234
H	0.6383424999	-4.3996190194	-2.5890959985
H	0.4273836815	-7.4131016899	-2.3361389450
H	2.4598519896	-3.0246149029	-0.4572978203

OPT-FDCH-zero-H

C	4.7455992715	-3.2844857592	-4.0018447126
C	4.8826514702	-2.4419380623	-2.9046635243
C	4.2051238018	-2.7240599836	-1.7098334780
C	3.9008349031	-4.3996555220	-3.9256061695
C	3.3906711388	-3.8562592612	-1.6032565624
C	3.2097399783	-4.6875314167	-2.7482099651
C	6.2092418852	-7.6697389110	-4.8489719692
C	7.2848593538	-7.0178640064	-5.4864648212
C	7.8575404478	-6.1892957707	-4.5573699927
C	6.1754188029	-7.2058998643	-3.5570087552
C	9.0957149096	-5.4057935890	-4.7813295760
C	5.2440853294	-7.5649153431	-2.5147713097
C	2.4653709005	-7.4764920698	1.0272392713
C	1.4880230348	-6.6766253112	1.6624403025
C	3.1025445332	-8.5029628728	1.7126507709
C	2.7625184835	-8.7648693921	3.0436237704
C	1.7948897605	-7.9909321530	3.6712386826
C	1.1612180546	-6.9496736236	2.9924228489
C	-0.7390426914	-6.5374184249	-1.9924059072
C	-0.8249560664	-5.1263783317	-1.9624130855
C	-1.7550632971	-7.2633376619	-2.6045175097
C	-1.8886914562	-4.4854934365	-2.5893749008
C	-2.8405193786	-6.6125610237	-3.2100410311
C	-2.8964432603	-5.2263003301	-3.2184531935
H	5.2805290586	-3.0777257936	-4.9199387200
H	5.5020155828	-1.5556475952	-2.9757190819
H	3.7892370833	-5.0693832608	-4.7704279740
H	4.2984298129	-2.0724266234	-0.8483007400
H	10.4614242210	-4.1718506841	-4.0249663003
H	4.2708428157	-7.2514079622	-0.8263425621
H	7.6457939855	-7.1185028565	-6.4956905748
H	5.4995352878	-8.3566321018	-5.2798190401
H	3.8560682780	-9.0906613971	1.2013980094
H	3.2528794029	-9.5664400547	3.5790398752
H	1.5283960262	-8.1931488314	4.7005187668
H	0.4104275138	-6.3376797710	3.4750872809
H	-1.6899071456	-8.3445300088	-2.6034203509
H	-3.5964695804	-7.2025843550	-3.7059000989
H	-1.9511083399	-3.4031964034	-2.5327271420
H	-3.6890343546	-4.7017195287	-3.7267415505
O	2.3455012826	-5.6930119332	-2.6150380154
O	2.7687744716	-4.2194638486	-0.4928500506
O	7.1733344220	-6.2925277214	-3.3729187543
O	9.6497029966	-5.5786042432	-5.8851176134
O	4.4380053189	-8.5796185769	-2.7656395391
O	8.9194936420	-4.1867833512	-2.7189311381
O	5.7491840278	-5.8970390688	-0.8538216088
O	2.7662013244	-7.1128672143	-0.2434091714
O	0.9811758741	-5.6682458326	0.9649632226
O	0.3539783191	-7.0839286942	-1.4357609902
O	0.1762960308	-4.4606561918	-1.3677446929
Fe	1.4990373275	-5.7037925654	-0.8195808128
N	9.5559578823	-4.5952037989	-3.8227048839
N	5.1293449459	-6.9672710405	-1.3527568841
Na	4.1784756945	-5.2771357953	0.8947537448
Na	0.9794401929	-3.3994327844	-3.0867607068
Na	5.3546628655	-10.5049221987	-2.8519351982
Na	6.9114113409	-4.2629265020	-1.9929304689
Na	2.2237306562	-8.0001595831	-2.4560447829

OPT-AHA-Five-H

C	1.3894857728	-1.3861202819	-2.2939448277
C	2.7327310617	-1.7452363839	-2.3026428632
C	3.3025237449	-2.4228832155	-1.2291613874
C	0.5886626275	-1.7097734666	-1.2011744656
C	2.5050629514	-2.7322636832	-0.1367471303
C	1.1626364002	-2.3731952087	-0.1370314482
C	4.9087491579	-5.8081658324	1.3269033492
C	5.6327510183	-6.4653025764	2.4572462904
C	1.9094608355	-3.5171035729	4.5008788049
C	2.4969508535	-4.7914153257	4.3296688536
C	2.9252306690	-5.5173921199	5.4391535032
C	2.7876981862	-4.9654492361	6.7106258887
C	2.2151315089	-3.7068125379	6.8774977513
C	1.7689685971	-2.9789653976	5.7772868750
C	-0.1522973963	-6.3641515774	2.3598381373
C	0.5230914346	-6.5059262294	1.1462545475
C	-0.8978944863	-7.3758711406	2.9259798019
C	0.4245858285	-7.7295860339	0.4831758018
C	-0.9743133264	-8.5979723034	2.2594790649
C	-0.3183257466	-8.7633855918	1.0446432740
H	0.9599781476	-0.8654616983	-3.1388130446
H	3.3481500846	-1.5021480637	-3.1591388981
H	-0.4645229603	-1.4649656699	-1.1715068424
H	4.3433677526	-2.7179745360	-1.2295981732
H	5.2142294521	-6.0559402623	3.3816720421
H	6.6880406762	-6.1901552652	2.4030148596
H	5.5354672140	-7.5510543003	2.4629184463
H	3.3566677117	-6.4991274464	5.2929846511
H	3.1230474900	-5.5240800594	7.5743885955
H	1.3191470896	-2.0014067984	5.8895370933
H	2.1087424182	-3.2907521373	7.8705269243
H	-1.4070587981	-7.2136245223	3.8671881761
H	-1.5487449279	-9.4083387357	2.6864691897
H	-0.3854567254	-9.7102643274	0.5242634118
H	0.9363327170	-7.8516587140	-0.4626227570
O	0.4131650882	-2.7841112354	0.9815884649
O	2.9460300497	-3.3705251741	0.9774820669
O	3.4287350939	-6.1255234719	-0.4839992115
O	5.0449999773	-4.5860609023	1.1104438370
O	1.5311392833	-2.9015624471	3.3685389066
O	1.2196273024	-5.4623152617	0.6795603571
O	2.6135293212	-5.2155277464	3.0600453440
O	-0.0452621649	-5.0804204760	2.9251336978
Fe	1.5021739143	-4.1663109718	2.0311059436
N	4.1302248644	-6.5945850282	0.5886246009
H	3.8784643853	-3.8895286680	0.9089541912
H	0.3719055612	-2.0642292906	1.6320173514
H	2.5394927570	-5.8418819576	-0.1081252120
H	0.1781138837	-5.1428339846	3.8708985144
H	3.9711114673	-7.5613150236	0.8337309491

OPT-AHA-Zero-H

C	1.1613557047	0.4823550766	-0.9568509921
C	2.5222346571	0.2682406464	-0.7833656838
C	2.9819743139	-0.9283508815	-0.2243906120
C	0.2411511292	-0.4999148470	-0.5728677725
C	2.0791533021	-1.9235571034	0.1516223115
C	0.6838854238	-1.7063004682	-0.0280059345
C	5.0884419427	-7.5842801982	-0.5211401582
C	6.0001666496	-8.4981291981	-1.2976578380
C	1.7573897900	-3.1639245276	3.9203396045
C	2.4556760372	-4.3559831966	3.6450848505
C	3.4527910096	-4.7931071961	4.5077869062
C	3.7364258493	-4.0691689924	5.6682429038
C	3.0277837180	-2.9122526558	5.9602926181
C	2.0360624296	-2.4587267902	5.0865323418

C	-0.4910748677	-6.1844065229	1.6244372270
C	0.3923136600	-6.3946649394	0.5278492054
C	-1.1079022686	-7.2799533991	2.2274005829
C	0.6086997646	-7.6810586375	0.0395718927
C	-0.8690066997	-8.5704605040	1.7388335875
C	-0.0247885784	-8.7694612700	0.6523033678
H	0.8059620705	1.4106278003	-1.3845148993
H	3.2337643616	1.0300665715	-1.0739933116
H	-0.8246689345	-0.3443389978	-0.6919562621
H	4.0394006702	-1.1074075773	-0.0716797899
H	6.9258929009	-7.9632355936	-1.5249613187
H	5.5234570101	-8.7493424021	-2.2485862586
H	6.2447831358	-9.4213777413	-0.7695234428
H	4.0159685763	-5.6833179451	4.2489514818
H	4.5142181124	-4.4128002382	6.3368932712
H	1.4581286092	-1.5708821165	5.3206566268
H	3.2402502023	-2.3578260317	6.8644424737
H	-1.7598631382	-7.1090571785	3.0752173116
H	-1.3467041708	-9.4175151201	2.2145530101
H	0.1571707880	-9.7700151807	0.2819100615
H	1.2991603107	-7.8194043540	-0.7843023306
O	-0.1291470344	-2.6610202233	0.4278788574
O	2.4336529496	-3.0636881861	0.7398324326
O	3.9282681287	-7.3090069541	1.5088759219
O	4.7186581237	-6.4894319412	-0.9956810127
O	0.8516996639	-2.7709633508	3.0056646064
O	0.9776504759	-5.2848607952	0.0527813555
O	2.0961561292	-5.0121112252	2.5271791225
O	-0.6634522718	-4.9112399466	1.9858488892
Fe	0.9245879295	-3.9286323808	1.5011364207
N	4.7069569829	-8.0224899037	0.6775069369
Na	1.9930200321	-7.2334194635	2.4641032506
Na	-1.2130350855	-4.4128960765	-0.2755688970
Na	3.4003453183	-5.2707342905	0.4285560211
Na	1.1334108583	-0.7383806286	2.3042934754
H	5.0862716501	-8.8889704186	1.0344124991

OPT-MAHA-Five-H

C	1.3292654547	-1.2720846392	-2.2827124886
C	2.6854144368	-1.6203366776	-2.3022350368
C	3.2684364133	-2.3226633144	-1.2406496676
C	0.5302402442	-1.6343749491	-1.1882481141
C	2.4741477838	-2.6752395619	-0.1427158893
C	1.1177560018	-2.3244587168	-0.1367243669
C	5.0601762386	-5.6473521199	1.3904393430
C	5.9515269592	-6.1123979209	2.5121703618
C	3.9334624964	-7.8826075389	1.2456846872
C	1.9232596376	-3.4836732587	4.5223687325
C	2.4905883653	-4.7870508271	4.3521386199
C	2.8921426864	-5.5317604051	5.4752186889
C	2.7451476341	-4.9792125355	6.7529477936
C	2.1961494418	-3.6959632519	6.9195149949
C	1.7825430375	-2.9450970983	5.8134588072
C	-0.2051463798	-6.3181466996	2.3701155735
C	0.4594149243	-6.4835087268	1.1404668611
C	-0.9996379679	-7.3025931840	2.9411979862
C	0.2894185076	-7.7073823004	0.4656966475
C	-1.1395892298	-8.5241808055	2.2649290851
C	-0.5005535978	-8.7138582576	1.0325512109
H	0.8889216495	-0.7314481726	-3.1184586312
H	3.3010111888	-1.3478321828	-3.1589150490
H	-0.5319834425	-1.3979693634	-1.1489827631
H	4.3202040659	-2.6051783641	-1.2511685336
H	4.6303041943	-8.1966774824	2.0291160260
H	3.9944617051	-8.5716368438	0.3934590124
H	2.9108202592	-7.8874097653	1.6535503490
H	5.3543766796	-6.1690663695	3.4374476373
H	6.7285670823	-5.3504448851	2.6523692455
H	6.4199200928	-7.0869238113	2.3265206508
H	3.3063726449	-6.5291467050	5.3297776262
H	3.0512476455	-5.5544187046	7.6256981863
H	1.3525383470	-1.9508600172	5.9278217508
H	2.0822215016	-3.2832850018	7.9210001328
H	-1.4979016424	-7.1194564343	3.8927540904
H	-1.7497542841	-9.3147766122	2.6973670399
H	-0.6174157486	-9.6599957521	0.5046335779
H	0.7922610488	-7.8494913235	-0.4903591316
O	0.3597142796	-2.7811342037	0.9826922719
O	2.9390479545	-3.3374269621	0.9561571950
O	3.5210245010	-6.1281099629	-0.3207685601
O	5.1010438547	-4.4212677007	1.0477239446
O	1.5760352398	-2.8491922019	3.3871101275
O	1.2176724859	-5.4728273153	0.6716809971
O	2.6275592023	-5.2092716460	3.0773999193
O	-0.0439677529	-5.0263313638	2.9457650769
Fe	1.5080648677	-4.1396258652	2.0294873636
N	4.2593420825	-6.5388574733	0.7838371199
H	3.9433652778	-3.8621200246	0.8678364652
H	0.3058233780	-2.0627403845	1.6498679176
H	2.5921332500	-5.8762048949	0.0414775338
H	0.2077653837	-5.1091015386	3.8966651239

OPT-MAHA-Zero-H

C	-0.3013763833	-2.3783386193	-3.0500665555
C	1.0396048230	-2.2975333311	-3.4009736709
C	2.0112188239	-2.8586677457	-2.5710927779
C	-0.6821311625	-3.0229156483	-1.8732321336
C	1.6407845429	-3.4979886969	-1.3947179132
C	0.2786151099	-3.5920212975	-1.0433341167
C	4.9927220418	-6.5759329642	2.1930710703
C	5.6160892361	-7.4427657654	3.2524255184
C	3.7640718156	-5.2460281371	3.8736991101
C	0.7999199735	-3.1273019152	2.9691543940
C	0.2387165766	-4.3743170603	3.3191579337
C	-0.5622602688	-4.4808848759	4.4495763500
C	-0.8222941655	-3.3569011729	5.2356704106
C	-0.2781865397	-2.1278878903	4.8878517425
C	0.5308809530	-2.0124784129	3.7573044952
C	2.8657405287	-8.3868137400	0.0275007277
C	2.0208165545	-7.3234707720	-0.4621005395
C	3.6832098828	-9.0078582449	-0.9540169283
C	1.9707745582	-7.0064133989	-1.8184570843
C	3.6486569949	-8.6532153334	-2.2973880546
C	2.7860651324	-7.6549420901	-2.7507930692
H	-1.0568642028	-1.9412769825	-3.6892122055
H	1.3348485064	-1.8033389222	-4.3166944460
H	-1.7233598232	-3.0872870190	-1.5834800762
H	3.0614283920	-2.8131956279	-2.8337429068
H	4.4108396891	-4.3653322366	3.9565030686
H	4.0407246808	-5.9578068786	4.6511375989
H	2.7313577150	-4.9251803263	4.0212012958
H	6.2914451310	-6.8536974975	3.8798327394
H	6.1914732671	-8.2138068013	2.7403051754
H	4.8742877562	-7.9198716373	3.8934289994
H	-0.9766154753	-5.4482344013	4.7071128000
H	-1.4485625071	-3.4472388858	6.1129646803
H	0.9565663215	-1.0590072562	3.4710988976
H	-0.4817620208	-1.2535905727	5.4916448588
H	4.3562124110	-9.7813449678	-0.6047465281
H	4.2981702966	-9.1641630403	-2.9985881634
H	2.7386858495	-7.3878420353	-3.7978512138
H	1.3153077353	-6.1971029756	-2.1255832045
O	-0.0277644908	-4.2549854568	0.0850306342
O	2.5287272219	-4.0607346499	-0.5442464270
O	3.4227224169	-5.0191751295	1.5609014586
O	5.4503462865	-6.5370240819	1.0533742334
O	1.6004574344	-3.0914792945	1.8938272527
O	1.2912388619	-6.6167060103	0.4383092047
O	0.5431514647	-5.4232887204	2.5223684279
O	2.9066721870	-8.7172881641	1.2739932741
Fe	1.5567798953	-4.7694753928	0.9856295682
N	3.8763478068	-5.8531170812	2.5522280989

Na	1.8340348380	-7.3820794452	2.6134033079
Na	-0.8985350860	-6.1347228488	0.6668024993
Na	4.1022808880	-5.8978091205	-0.7394450755
Na	3.5563429613	-2.7725801466	1.0534877168

XYZ coordinates of the considered molecular complexes including 12H2O explicit molecules optimized at BLYP-D3, DZVP- MOLOPT

OPT-THFDCA-5H

C	-0.0453557369	0.9998514131	1.0890044817
C	0.0439624468	1.4544867101	-0.2260576738
C	0.9750200241	0.9808557827	-1.1529835609
C	0.6297385254	-0.1923786821	1.4139789851
C	1.6331267983	-0.2077100176	-0.8094289420
C	1.4310742690	-0.8262817372	0.4504164918
C	-3.0584477306	-4.5740432195	2.8218481761
C	-3.1010309012	-5.8853256369	2.0344377213
C	-3.3126356324	-5.3866248235	0.5889168413
C	-4.1250128892	-3.7288462376	2.0973022435
C	-1.9735904925	-5.1359320294	-0.0792400474
C	-3.8285694344	-2.2543590045	2.3355420022
C	4.7059083094	-4.1959419747	0.3641924500
C	3.5151666891	-4.8849451985	0.6873187664
C	5.9271436657	-4.4673323050	1.0083314748
C	5.9270024663	-5.3779059359	2.0816922408
C	4.7113188186	-5.9847486458	2.4754195141
C	3.5212202450	-5.7661252330	1.7814197803
C	0.9901489445	-3.8574474569	-3.4825982861
C	-0.1192187724	-3.4848369921	-2.7031224529
C	0.7735830873	-4.3978860427	-4.7552207811
C	-1.4238424484	-3.6161113709	-3.1675570276
C	-0.5361586014	-4.5543433022	-5.2207870646
C	-1.6289412125	-4.1616404030	-4.4396429283
H	-0.8098817316	1.2861592857	1.8092467601
H	-0.5153712348	2.3533456591	-0.5049712004
H	0.4382596168	-0.6883414044	2.3655515954
H	1.0085054656	1.2673565578	-2.2013601093
H	-3.9750379222	-6.4919910322	2.3189789703
H	-2.1817957071	-6.4745879959	2.1541633672
H	-2.0742739093	-4.0925943021	2.7154119253
H	-3.2652225221	-4.6918059186	3.8907368385
H	-3.9162013450	-6.0889551517	-0.0077008443
H	-5.1880933679	-3.9068274938	2.4373518969
H	4.6964848537	-6.6670341852	3.3263553447
H	2.5872093624	-6.2570180816	2.0600377010
H	1.6322907080	-4.7231825946	-5.3391378012
H	-2.6433436561	-4.2765812335	-4.8185385844
H	-0.7013244693	-4.9719121244	-6.2151182642
H	-2.2430331991	-3.2749387052	-2.5410566266
H	6.8718618663	-5.6033937776	2.5885656314
H	6.8645305504	-3.9953481714	0.6817662771
O	1.9339146732	-2.0621982509	0.6316805599
O	2.3433447693	-0.9814149862	-1.7313590642
O	-4.0446324903	-4.1370216178	0.7049158767
O	-4.0648333352	-1.7764543323	3.4471635574
O	-3.1416451978	-1.5386450279	1.4396002036
O	-1.3424404592	-4.0906818098	0.1852321993
O	-1.5843682919	-6.1138461936	-0.8599949542
O	4.6261869613	-3.2584537386	-0.6969017656
O	2.4202523079	-4.6930637647	-0.0672410381
O	2.2217287079	-3.7486392571	-2.9176646715
O	0.1974776437	-3.0204739941	-1.4324957425
Fe	2.2251689389	-3.0433701613	-1.0364227879
H	-0.5565318543	-6.1259053405	-1.1006981631
H	-3.0040745606	-1.9068215916	0.4896382821
H	4.6891168035	-2.3256945377	-0.2961618067
H	-0.4574785197	-3.4462467957	-0.7171161528
H	3.2358231090	-0.5829818636	-2.0689159081
O	4.7142418474	-0.8566770483	0.3879127640
H	5.6656332800	-0.6043169083	0.2992390808
H	4.5956004587	-1.0111365906	1.3440523887
O	0.2110059896	-6.4174538576	2.1784294927
H	0.3595245286	-5.4355941834	2.1702424834
H	0.0580836125	-6.6774590602	1.2533573172
O	-2.8819285707	-1.6140230366	-1.1161025035
H	-2.9816489958	-0.6305868290	-1.0828376977
H	-3.7826011361	-1.9558488693	-1.3823008506
O	-3.0110011707	1.1555852490	-0.5408204897
H	-2.0272727474	1.1620891155	-0.5548298493
H	-3.2078100331	1.1373615238	0.4160921956
O	-3.0394496370	-8.8391087805	0.5391278530
H	-2.0621463732	-8.8407904876	0.4208839842
H	-3.1386837986	-8.8597679268	1.5060107956
O	-0.1786150194	-8.8479556593	0.5166154829
H	0.3998070296	-8.8432124058	1.3008787980
H	0.4204244639	-8.7950786143	-0.2515341345
O	2.7490684315	-6.4098904274	-3.1677589177
H	2.7530161502	-5.4168830039	-3.0929388617
H	3.5636951365	-6.6985629744	-2.7245094441
O	-5.3377403771	-2.4389606690	-1.1370548644
H	-5.3506477979	-1.7473683814	-0.4441009139
H	-5.3001015767	-3.2709484926	-0.6074199051
O	-1.4704626017	-0.5264057999	3.7834158647
H	-2.3995360495	-0.8286109997	3.6645815337
H	-1.3364617944	-0.5805741106	4.7440533903
O	0.8767573747	-6.5208694517	-1.1832188135
H	1.4428771590	-5.8926160616	-0.6438213903
H	1.3863668211	-6.5781109724	-2.0396667481
O	0.7857183715	-3.7289285637	2.5893046302
H	1.5373417730	-3.8290264884	3.1960728195
H	1.1416504844	-3.1456204826	1.8699119006
O	4.6466216075	-0.4406882630	-2.6917940670
H	4.6734308015	0.2121802252	-3.4118153841
H	4.6873333902	-1.3626063617	-3.1403105272
O	4.6528188785	-2.9127140997	-3.5102911888
H	4.7725957798	-3.2734169853	-2.6023040946
H	3.7007582502	-3.1610035434	-3.6623424861

OPT-THFDCA-0H

C	2.4449076456	0.6948166701	2.0829930773
C	2.9449429121	1.1837539687	0.8845202890
C	2.6132336569	0.6625987774	-0.3670309862
C	1.7635349631	-0.5441228884	2.0532494181
C	1.9827756726	-0.6098086536	-0.3826644716
C	1.5610246041	-1.2108574286	0.8413576427
C	-4.2750219010	-3.6489187489	3.0317332425
C	-4.5868195782	-5.1285735074	2.8300785954
C	-4.2111219597	-5.3677193095	1.3757319799
C	-4.6361468883	-3.0624556078	1.6762264044
C	-2.6387382529	-5.6165541587	1.2824687941
C	-4.2118021799	-1.6738015056	1.2841106918
C	4.2752543722	-4.0263758141	0.2734381113
C	3.6980944163	-5.3164993424	0.0208397344
C	5.4362329120	-3.9073667557	1.1234446224
C	5.7240904132	-5.0500999733	1.8876574430

C	5.4278928008	-6.3389960215	1.4506097696
C	4.3036951279	-6.4590539855	0.5629882701
C	0.5360842807	-4.5493696167	-3.3073596914
C	-0.2944160302	-4.9915831634	-2.2383021663
C	0.3890129944	-5.0930554470	-4.5895435110
C	-1.2784251503	-5.9648449704	-2.4916941168
C	-0.5955951792	-6.0702270467	-4.8228874920
C	-1.4250712646	-6.4972354359	-3.7827504441
H	2.7413882657	1.0403997587	3.0676980073
H	3.5262360206	2.1093461307	0.9114807090
H	1.4651672903	-1.0295792733	2.9849548188
H	3.0866929506	0.9458809463	-1.3082131690
H	-5.6967987066	-5.2300062744	2.8784162038
H	-4.2600852282	-5.8615117425	3.5725203872
H	-3.1764813163	-3.5738898029	3.1366637198
H	-4.6185856062	-3.1172977113	3.9197370554
H	-4.6019250910	-6.2264310664	0.8282870342
H	-5.7481610541	-2.9565659631	1.7178572248
H	5.6548768792	-7.2314145752	2.0341187097
H	3.8907538207	-7.4337256211	0.2997992486
H	1.0524658132	-4.7568463624	-5.3878983163
H	-2.1893462262	-7.2510244292	-3.9655005996
H	-0.7060672129	-6.4909595035	-5.8212265831
H	-1.9273758532	-6.3090986329	-1.6801693271
H	6.5038395037	-4.9483750125	2.6564896410
H	5.6218744261	-2.8939942491	1.4968869203
O	1.0211576576	-2.4605146459	0.7573470205
O	1.8108287705	-1.3557584242	-1.4951115534
O	-4.4384548883	-4.1135953741	0.6805976406
O	-4.4506951985	-0.7470433382	2.1004854068
O	-3.3686446988	-1.6012667536	0.2959955594
O	-1.8554724075	-4.6084874623	1.4726222947
O	-2.2629307638	-6.7765975841	0.9604455476
O	3.6629935177	-2.9970787537	-0.3115879335
O	2.5558068331	-5.3187328385	-0.6954773465
O	1.4554015679	-3.6049544340	-2.9850170215
O	-0.0814629274	-4.4297865644	-1.0303282206
Fe	1.7320601402	-3.4432973295	-0.9511197635
Na	-0.0399417414	-6.6252336265	0.2082189701
Na	-0.7848827242	-2.5965366031	2.1808199083
Na	5.5561883708	-1.8520106579	-1.1111412961
Na	-2.2607119955	-3.4964390929	-0.6197296736
Na	0.9454353727	-1.4384790121	-3.6617890781
O	5.5797000403	-0.3530446984	0.6876250493
H	5.5481309799	0.6336355959	0.7151277158
H	5.5523562397	-0.6235804717	1.6255801570
O	1.4422759393	-6.5879815079	2.1021868684
H	1.4442932580	-5.6615103163	2.4570208694
H	2.3570191810	-6.6746873396	1.7689209408
O	-1.3346644890	-1.1125917585	-1.3778098557
H	-2.0580346780	-0.9633526815	-0.7076765827
H	-0.5015829648	-1.1411221348	-0.8711728541
O	0.3733332782	0.8388314914	-3.0117105242
H	0.9751251317	0.8505196338	-2.2376657296
H	-0.5091037441	0.8075011288	-2.5856120744
O	0.5348594385	-9.1448867359	0.1899269771
H	1.4597015963	-9.1924724159	-0.1501933460
H	0.6267279575	-9.1788826514	1.1597866907
O	2.9525077887	-9.1794527708	-1.2005870632
H	3.9252377861	-9.1709271041	-1.1937207841
H	2.7126187838	-9.0804195799	-2.1419354727
O	4.3827414055	-5.5416174452	-4.8147283025
H	5.3490107029	-5.6646431239	-4.6651250367
H	3.9037411579	-6.0219084631	-4.0785574843
O	-4.4293439504	-3.2812070368	-1.9507699879
H	-4.4366818481	-2.3272333713	-1.7325025796
H	-4.5073418890	-3.6843355650	-1.0516822974
O	-1.9450914330	-0.8605335785	3.1119009337
H	-1.8712805011	-0.3293384898	3.9193518257
H	-2.8614478988	-0.6556100259	2.7449578108
O	2.7499360275	-6.7819582537	-3.0179872547
H	2.6824553536	-6.2833609805	-2.1507312620
H	1.9238476999	-6.5323566467	-3.4818981053
O	1.7960099899	-3.9139602107	2.8322802167
H	2.7606770478	-3.9110666658	2.9605014177
H	1.6563667892	-3.3988630196	1.9789798445
O	4.1470923995	-1.1953292749	-2.8662753372
H	4.0134528918	-1.8476429210	-3.6137507327
H	3.3038155155	-1.2738510680	-2.3387952398
O	3.3214206216	-2.9955152149	-4.7190880257
H	3.8437263859	-3.8173276082	-4.9165421958
H	2.6546117408	-3.3575633247	-4.0620959014

OPT-FDCA-5H

C	4.1398849985	0.2023223945	2.0629266224
C	4.5195975481	0.6962666811	0.8154874371
C	4.1555018058	0.0748929514	-0.3837191148
C	3.1256629709	-0.7794753077	2.1137481576
C	3.1130950140	-0.8856813831	-0.3407045489
C	2.6249395280	-1.2956983736	0.9245455896
C	-4.6573267251	-1.7368794728	1.4501610392
C	-3.3588150221	-1.6362367206	2.0257731908
C	-2.6725869967	-2.7777222266	1.6388686519
C	-4.6722415286	-2.9196332112	0.7392236433
C	-1.2940311604	-3.2261055508	1.7260740622
C	-5.5864334435	-3.4508864845	-0.3542076970
C	3.1958161120	-5.1972154561	-0.1894668821
C	1.9271321004	-5.5742049488	0.2672507909
C	4.3425182744	-5.9064983635	0.1596871944
C	4.2651536902	-6.9782079599	1.0636869012
C	2.9950638748	-7.3362408580	1.5574903400
C	1.8339107292	-6.6585460514	1.1519863058
C	-0.4727035268	-3.6823075704	-3.1536839203
C	-1.1613881617	-2.9204941770	-2.1757189247
C	-1.1678656140	-4.3537413261	-4.1600911856
C	-2.5557707867	-2.7888770733	-2.2605660825
C	-2.5680424214	-4.2510371103	-4.1991543670
C	-3.2569724727	-3.4635084429	-3.2652497449
H	4.4150888396	0.6947025842	2.9924802103
H	5.2631110495	1.4983659925	0.7759972468
H	2.7274522771	-1.1427618509	3.0615736863
H	4.4037621834	0.4708254663	-1.3682012799
H	-2.9215983858	-0.8123002402	2.5922556454
H	-5.5700611135	-1.1138612594	1.5321774092
H	2.8921339861	-8.1570246061	2.2668964137
H	-0.6221294037	-4.9401397018	-4.8985198539
H	-4.3490808508	-3.3835497568	-3.3037578634
H	-3.1191717405	-4.7817683672	-4.9734316407
H	-3.0696951691	-2.1677032428	-1.5291970361
H	5.1784231116	-7.5011917175	1.3605188590
H	5.3143791385	-5.5665060472	-0.2092454118
O	1.6860796387	-2.3097226176	0.8552213329
O	2.5780042317	-1.4277278873	-1.4512322569
O	-3.4878231652	-3.5830614691	0.8594723950
O	-5.7183490052	-2.7558943857	-1.3461696339
O	-5.7123229843	-4.8090019581	-0.3772149593
O	-0.4095215112	-2.5086269499	2.2905322207
O	-1.1002744450	-4.3986454124	1.1901869523
O	3.2728911047	-4.0493314112	-0.9536179775
O	0.8718977995	-4.8345589579	-0.1767049400
O	0.9000114008	-3.7162032482	-3.0053429707
O	-0.4181047379	-2.3632772621	-1.2022222937
Fe	1.4061715691	-3.0035930643	-1.1513090728
H	-0.1403031094	-4.5956503334	0.7377070456
H	-5.6774188876	-5.1638977697	0.5392276688
H	0.8642910458	-6.9267663056	1.5773424709
H	4.1903025256	-3.5686729127	-0.7033661356
H	0.8957458992	-2.2737924160	1.5076393719
H	1.3564888593	-4.7205128215	-3.4259191074
O	-0.9472142142	0.4022337134	2.5952541790
H	-0.6371842450	-0.5089233057	2.7478124210

H	-0.9209016472	0.4613605999	1.6049828053
O	-0.1103453471	-6.7222611086	-2.0117552078
H	0.0776920433	-6.0576485806	-1.3001212313
H	-0.9218751847	-6.3836853252	-2.4353027679
O	4.3126009064	-3.0876331854	-3.4660400416
H	4.2489508664	-2.3402333321	-2.8393543031
H	4.3100264246	-2.6841195135	-4.3511244708
O	1.0156845227	0.8482439201	-2.1817832044
H	1.5438438005	0.0249008076	-2.0529128295
H	1.6103962054	1.5632147653	-1.9018053111
O	-0.9608879691	0.2084204022	-0.2004997864
H	-0.3339172243	0.6191018425	-0.8404401443
H	-0.9148326073	-0.7398190012	-0.4584021956
O	-0.1834570911	-6.5182541912	3.5783309228
H	0.4518379159	-5.7741612650	3.6630730212
H	-1.0580895741	-6.0790056643	3.5657918731
O	4.2966206649	-5.8666501559	-3.1760467487
H	4.3242225627	-6.1357607072	-2.2364700732
H	4.3337245481	-4.8741652319	-3.1567821632
O	4.3087323636	-3.8880541877	2.3437660709
H	4.3231355686	-3.6760728308	3.2952552854
H	4.3230739603	-4.8677012752	2.2818791724
O	1.5724480865	-4.1407864035	3.5864519603
H	2.1500931075	-4.1549091170	2.7987856425
H	0.8332454421	-3.5568652083	3.3079560221
O	5.3875904987	-2.9366535773	-0.0370504484
H	5.2384164929	-3.1566265956	0.9200295438
H	5.3083488204	-1.9586484795	-0.0781601373
O	1.7076113209	-5.8802690846	-3.7512528077
H	2.6949853261	-5.9985717983	-3.5316634875
H	1.1351448069	-6.4083944743	-3.1033970377
O	-2.7577391389	-5.0770906595	3.9909706549
H	-2.8859862631	-4.1850429550	3.6195776285
H	-2.6929947785	-4.9384305690	4.9521800920

OPT-FDCA-0H

C	4.2182296127	-1.0213627529	3.0633449278
C	4.1979249384	0.1149172494	2.2383067533
C	4.2098198409	-0.0172260499	0.8464086864
C	4.1888854025	-2.2914562027	2.4822825664
C	4.1959252956	-1.2892522275	0.2128970785
C	4.0675271634	-2.4373777494	1.0850573010
C	-3.4429885811	-0.3415411638	1.4660724857
C	-2.6020436856	-1.2090641529	2.2165378883
C	-3.1055560814	-2.4875766806	2.0810503604
C	-4.4488250056	-1.1165492581	0.9111569543
C	-2.6124693353	-3.8084442472	2.4602234897
C	-5.6913336450	-0.8413474636	-0.1254787515
C	3.0193817944	-6.6083240711	-0.6162251322
C	1.8704059860	-6.9068010568	0.1780006600
C	4.1986747814	-7.3513280418	-0.4241884168
C	4.1866996112	-8.4796315975	0.4173903299
C	3.0004223421	-8.8672064270	1.0631027596
C	1.8565259280	-8.0584909196	0.9706603395
C	0.3135849222	-2.9081499534	-2.3578414624
C	-0.4501013540	-2.7915127109	-1.1594197896
C	-0.2109899161	-2.4071049040	-3.5589339104
C	-1.7169116792	-2.1914914515	-1.1917848428
C	-1.4854573916	-1.8206176878	-3.5812538226
C	-2.2392944840	-1.7130831415	-2.4052971237
H	4.2180408871	-0.9224429482	4.1497030064
H	4.1892673035	1.1166006868	2.6782458249
H	4.1625633868	-3.1847384548	3.1106849004
H	4.2040526305	0.8772731550	0.2109996143
H	-1.7168952965	-0.9456835101	2.7889006931
H	-3.3468463189	0.7336399874	1.3397603197
H	2.9674104978	-9.8174792511	1.6141902294
H	0.4034465614	-2.4488819684	-4.4599157893
H	-3.2300958657	-1.2568396801	-2.4128416099
H	-1.8838303388	-1.4330030668	-4.5178844547
H	-2.2661495981	-2.0689657880	-0.2604983991
H	5.1069959539	-9.0468426797	0.5532484534
H	5.1369836243	-7.0229300119	-0.8857072865
O	3.3518096170	-3.5806371517	0.6368795842
O	4.1913875747	-1.4088817488	-1.1137624257
O	-4.2431921572	-2.4333252129	1.2852048546
O	-5.8236400861	0.3783993098	-0.2778073896
O	-5.8215514855	-1.8750962314	-0.8742144442
O	-1.6171228512	-3.8886180865	3.2561294700
O	-3.1866705839	-4.8438359879	1.9318249044
O	2.9248504508	-5.5601116169	-1.4538552580
O	0.8610708222	-5.9989742837	0.1307475545
O	1.5129605390	-3.5124997395	-2.2530983882
O	0.1366000752	-3.2465500248	-0.0244244554
Fe	1.7604499355	-4.2594583667	-0.4080345498
Na	-0.2060444397	-5.3923493268	2.1799211903
Na	-5.8000409412	-3.8320563798	-0.0407198774
H	0.9449741655	-8.2950394117	1.5189495226
Na	4.2313292169	-3.6627381081	-1.3681746210
Na	1.1124896388	-1.5207120229	1.2773777000
Na	1.5255374756	-5.5519363729	-3.9838805655
O	0.6390903266	-2.4261665011	3.3292687921
H	-0.2725868640	-2.8277932289	3.4037834037
H	1.2333939276	-3.1713158250	3.5673372749
O	-3.3184728744	-4.9042685218	-0.8335918342
H	-3.1636847367	-4.9155218237	0.1484179556
H	-2.8831049910	-4.0774045581	-1.1283536913
O	4.1701040095	-4.5324936730	-4.5675712537
H	4.2507037624	-5.2771296437	-3.9348228334
H	4.2472980208	-3.6986988459	-4.0634478852
O	2.2787115216	0.0821965145	-2.1760227194
H	3.0634231051	-0.4543868973	-1.8126939516
H	2.6084559640	0.9891230282	-2.2957117848
O	0.4435568197	0.0617679085	-0.2291333445
H	1.1002180311	0.1086316790	-0.9915527808
H	-0.4041138099	-0.1479446624	-0.6616321392
O	-0.5923155276	-7.3526155706	3.4304110946
H	-0.4035317603	-8.3011084360	3.4845341958
H	-1.5896963178	-7.2672356387	3.5832646850
O	4.1866076198	-7.1277192809	-3.5731761163
H	4.2313037788	-7.9453016818	-4.0978108775
H	4.1707692574	-7.4173830474	-2.6343522326
O	4.2024158751	-5.5728967721	2.3451342929
H	4.1979423179	-6.4270228783	1.8655567596
H	4.1424280508	-4.8877188210	1.6256887349
O	1.8037304786	-5.0564875151	3.4348241362
H	1.6740999814	-5.6404605705	4.2012416203
H	2.7102513930	-5.2978072080	3.0637367436
O	5.6994658692	-2.5972479699	-2.8606438288
H	6.6666518217	-2.5674625188	-3.0096985661
H	5.4767377718	-1.8556645106	-2.2292921207
O	-0.2095641484	-6.2883004512	-2.5074558030
H	0.1168015348	-6.2325092403	-1.5698266852
H	-1.1355739877	-5.9907885650	-2.4324623131
O	-3.1762433726	-6.8627581949	3.6522112426
H	-3.2040280448	-6.1064575549	2.9748250105
H	-3.3097764548	-6.4139634605	4.5038388981

OPT-THFDCH-5H

C	-1.5221906367	-2.0401041370	-0.7448470496
C	-1.1776960534	-2.1072446499	0.6122219227
C	-0.3600120840	-3.1576623804	1.0658903323
C	-1.1128762664	-3.0112355699	-1.6617428542
C	0.1310097733	-4.0681295090	0.1362480957
C	-0.1558124206	-3.9588097510	-1.2440588751
C	8.6760415842	-7.7282724710	-3.4498417950
C	8.6973079462	-9.0161355499	-2.5706475800
C	7.4383757169	-8.9425242278	-1.7011978735
C	7.2781143500	-7.0824897104	-3.2002820943
C	6.6181235668	-10.2435397582	-1.5472593293
C	6.4723312093	-6.9362806660	-4.4826747091
H	-2.2253618328	-1.2599497693	-1.0683238643

H	-1.4338061039	-1.2922887101	1.2914927422
H	-1.3487077468	-2.9554818284	-2.7232373793
H	-0.0125708466	-3.2095413859	2.0959095059
H	5.6579838476	-9.2938023979	0.0304439491
H	5.7258268267	-4.9558410790	-4.0598716281
O	0.5668361014	-4.7028373587	-2.1072231041
O	1.1281611804	-4.9990791078	0.4316739377
O	6.5237633279	-8.0290909959	-2.3919904793
O	6.6510611313	-11.1663757236	-2.3643602248
O	6.3113736870	-7.8765166859	-5.3003075529
O	4.8164859858	-11.1415116946	-0.1894413452
O	5.2282032824	-5.6371141984	-5.9290725384
Fe	2.2119334486	-5.4835758710	-1.3848288884
N	5.8241161816	-10.1928340805	-0.4393082663
N	5.9703816179	-5.7173779026	-4.7451502686
O	3.4289793160	-3.9160562510	-1.1685540145
O	3.4114288478	-6.1753196613	0.2889518259
O	2.9257557811	-6.1527405068	-2.9996359621
O	1.2913499277	-7.6244467824	-1.4973040878
C	4.1706570294	-3.9421567543	-0.0384965965
C	4.1117865523	-5.0787679733	0.7920969318
C	5.0380362113	-2.9036774974	0.3255308553
C	5.7629902670	-2.9744289834	1.5231760914
C	5.6366499705	-4.0824071626	2.3720205244
C	4.8083708768	-5.1501677249	1.9929536506
H	5.1247921626	-2.0432478870	-0.3355659500
H	6.4019804692	-2.1334412080	1.8002554055
H	6.1759505777	-4.1216300121	3.3175701242
H	4.7207656246	-6.0497529131	2.6008830108
C	2.3331089440	-7.1948867487	-3.5900346602
C	1.4761032254	-8.0195113219	-2.8246403677
C	2.5286414542	-7.5034854016	-4.9499837975
C	0.8779432395	-9.1506498641	-3.3705913564
C	1.9123334745	-8.6304325297	-5.5056031042
C	1.1003563984	-9.4583519827	-4.7199422069
H	3.1696759792	-6.8425699959	-5.5320617273
H	2.0734147451	-8.8677891980	-6.5568527383
H	0.2272084850	-9.7637421205	-2.7480132354
H	0.6280903509	-10.3343308612	-5.1588990613
H	8.7191274572	-8.0020051223	-4.5156402725
H	9.5078521999	-7.0446195149	-3.2171868095
H	7.3419123874	-6.1235664068	-2.6600230290
H	9.6127790122	-9.0676043638	-1.9570469156
H	8.6716358165	-9.9212465368	-3.1981851492
H	7.6653422150	-8.5157614210	-0.7129041027
H	4.4607130243	-11.4451752179	-1.0996932589
H	5.3820791274	-6.5671800761	-6.2668857506
H	0.9129228539	-5.7647913413	1.0582261419
H	0.2681772364	-7.7205244737	-1.2585839352
H	4.1723872566	-6.8635872434	0.1158109448
O	-1.2647499603	-7.6755221290	-1.1202687541
H	-1.3561455067	-7.0869714511	-0.3489626312
H	-1.3637883385	-7.0766141136	-1.9510936051
O	2.3083293968	-2.0922257344	2.1339020669
H	2.2447920424	-1.9043014722	1.1707072846
H	3.2658014986	-2.0825523484	2.2965854787
O	-1.3424730679	-7.6439386865	-5.6422487941
H	-1.2655472222	-7.0578077809	-6.4168812303
H	-2.3003273623	-7.8567760084	-5.6159074126
O	3.6972312095	-12.0431579690	-2.4168826567
H	3.9148241560	-11.2787196961	-3.0297591958
H	2.7266528903	-12.0547635088	-2.3550758223
O	7.5959011794	-5.5439927273	0.1941190806
H	8.5756989346	-5.5111922945	0.2833706140
H	7.2465189987	-4.7530691865	0.6549859641
O	4.3818521811	-9.7829403078	-3.7495682047
H	5.2963473057	-9.5322337094	-3.9824888625
H	3.8168392676	-9.2521387065	-4.3422430465
O	2.2084429465	-1.1992566022	-0.6893650191
H	1.2452093940	-1.1861858398	-0.8440894501
H	2.4968531079	-2.0521393799	-1.0822148102
O	-1.3174251820	-6.1919035829	-3.2505396382
H	-1.2277647001	-6.6821515217	-4.1025822519
H	-0.5072640250	-5.6342916093	-3.1084048520
O	5.5548713993	-7.4021832519	-0.0765260241
H	6.2529961119	-6.7622566689	0.2150971501
H	5.7365087938	-7.4954898873	-1.0644570986
O	0.8458039098	-7.2860777182	1.7098705822
H	1.5643389130	-7.9046262591	1.4109356720
H	0.8343776094	-7.3557747717	2.6782120076
O	2.6836683830	-9.0831059596	0.5084446674
H	3.2639867611	-9.8675986869	0.3861609414
H	2.3622919227	-8.8353036079	-0.3831987922
O	5.3285331504	-3.6457193399	-3.0833633300
H	4.5633411991	-3.7792763181	-2.4526600633
H	5.0692679794	-2.8838040219	-3.6269324362

OPT-THFDCH-0H

C	-1.1229076052	-1.8279916764	-1.3096773263
C	-1.1282242260	-1.7741591391	0.0967127836
C	-1.0468242298	-2.9520800448	0.8595200207
C	-1.0498245944	-3.0865852127	-1.9474876649
C	-0.7956561401	-4.1878331289	0.2349675139
C	-0.8342108033	-4.2547323346	-1.1963398937
C	8.9002397873	-8.3215941075	-4.3729251812
C	8.8987527886	-9.5633116617	-3.4116946472
C	7.5890752881	-9.4147449907	-2.6154409156
C	7.5379132907	-7.6222550593	-4.1148550325
C	6.7147043614	-10.5609729324	-2.0632312717
C	6.7603238801	-7.0814558757	-5.3141224729
H	-1.1678161145	-0.9052845838	-1.8920770147
H	-1.1693904150	-0.7986642085	0.5911420491
H	-1.0343972474	-3.1644962944	-3.0359264654
H	-1.0458695203	-2.9154102994	1.9538998976
H	6.2648486916	-9.0819085401	-0.7257946622
H	6.0542230033	-5.5075658214	-4.1468391484
O	-0.3206019768	-5.3974158207	-1.7573885405
O	-0.2698066546	-5.2416949462	0.8962688790
O	6.6898872691	-8.6734868706	-3.5410817450
O	6.5498702477	-11.7373091305	-2.5284892744
O	6.7144833821	-7.6937032557	-6.4249304529
O	4.7594726934	-10.4348854967	-0.6286217267
O	5.2495844638	-5.3612799205	-5.9789531491
Fe	1.5448748565	-5.7195781297	-0.9084186232
N	5.9483636719	-9.9847367464	-1.0992672380
N	6.0698362219	-5.9544430069	-5.0757848175
O	2.8252867032	-4.3144669951	-0.4625138026
O	2.4897811260	-6.4610466775	0.9376727422
O	2.4339925336	-5.5423973733	-2.7194108636
O	1.5148255217	-7.6865206568	-1.4918789145
C	3.8836291790	-4.6987639258	0.2560048437
C	3.6815963064	-5.8475591223	1.0906789487
C	5.1111233192	-4.0197305860	0.2744224810
C	6.1122834284	-4.4290606741	1.1677976559
C	5.8947977350	-5.5082690947	2.0381409915
C	4.6831653114	-6.2244767037	2.0075924120
H	5.2625443973	-3.1950185862	-0.4218229268
H	7.0558235499	-3.8875504699	1.1954836938
H	6.6771662289	-5.8110315033	2.7325097326
H	4.5311011162	-7.1027321014	2.6358328860
C	2.1637744115	-6.5909191754	-3.4947650359
C	1.7247468046	-7.7783471193	-2.8263264138
C	2.2959552434	-6.5780678197	-4.8939563995
C	1.5310925809	-8.9482539311	-3.5685936494
C	2.0038600854	-7.7378289160	-5.6275973694
C	1.6288281857	-8.9157228894	-4.9677249133
H	2.6466439767	-5.6681185948	-5.3797677547
H	2.0614587191	-7.7234788100	-6.7143832954
H	1.2209314022	-9.8526254816	-3.0509772692
H	1.4016849464	-9.8072914773	-5.5508912011
H	8.9203474282	-8.6393703640	-5.4280689591

H	9.7355316522	-7.6321121339	-4.1872737026
H	7.6449099262	-6.8349725274	-3.3473785534
H	9.7576977735	-9.5706876845	-2.7254750886
H	8.9026290029	-10.5078878021	-3.9787215698
H	7.7557364944	-8.7206565330	-1.7783361458
Na	4.2389323784	-11.8047258619	-2.3430276174
Na	5.3096024447	-6.5438481314	-7.9401888384
Na	2.0947965617	-3.9125001814	2.0898936924
Na	3.4970889448	-8.5199230951	-0.2866491915
O	-2.3155155836	-6.8793273351	0.6891695937
H	-1.5644739859	-6.1838598488	0.8506852445
H	-3.1856413188	-6.4483837617	0.8010733655
O	3.0029719694	-1.7964861649	2.0445449687
H	2.7088489486	-1.7365808262	1.0850490496
H	3.9771141115	-1.7864932253	2.0074386582
O	-1.1706496212	-9.1175456708	-3.0091352114
H	-1.0840758486	-8.2978224984	-3.5851244889
H	-2.1065670240	-9.3838323590	-3.1561878552
O	3.0851689151	-11.7871930812	-4.2466376921
H	3.5905325303	-11.0395635415	-4.6932689136
H	2.1856548374	-11.7730683159	-4.6097812024
O	7.7444289993	-7.5111562757	0.1283176262
H	8.7403529139	-7.4746876282	0.1646216831
H	7.4533349966	-6.6138408929	0.3783211984
O	4.5804438081	-9.7458237101	-5.1380692173
H	5.4325624931	-9.4896621104	-4.7076644757
H	4.0075917615	-8.9576482212	-5.0585967355
O	1.9561437845	-1.7666681567	-0.4271617874
H	0.9989567324	-1.7850693300	-0.6572438369
H	2.4474505876	-1.8309990382	-1.2642641477
O	-1.0970401085	-6.6256057466	-4.1249014692
H	-0.9610163929	-6.0110312026	-3.3488049139
H	-0.2406678351	-6.5671104190	-4.5961021421
O	4.8839252485	-7.2268863302	-1.7834205605
H	4.4519924918	-6.4535570415	-2.1970443309
H	5.3758718964	-7.6895116239	-2.5006485189
O	-0.1143880099	-8.1103569825	2.0581476831
H	0.2737221733	-7.2174719812	1.9660304174
H	-1.0754133303	-7.9377815942	1.9931257775
O	2.6663834414	-8.9440385651	2.0506144122
H	2.5207983322	-7.9693522649	1.9481277354
H	1.7439778306	-9.2641716638	2.0858716549
O	4.5839660839	-3.9912720290	-3.6431368863
H	3.7630122304	-4.4098853162	-3.2867942673
H	4.5966406674	-4.2597341359	-4.5973650697
Na	-1.1070444047	-7.6139578803	-1.1914906090

OPT-FDCH-5H

C	5.2943382612	-3.6235514306	-3.1918404705
C	5.3895465876	-2.8722831541	-2.0142375364
C	4.4986405831	-3.0954896426	-0.9533816977
C	4.3019964888	-4.6019955810	-3.3300599359
C	3.5330819809	-4.0937542731	-1.0797300289
C	3.4182540413	-4.8594422880	-2.2673290536
C	6.3572899045	-7.0062972146	-5.7409060072
C	7.3892107123	-6.0780467903	-6.0674505363
C	7.8720075822	-5.6232172291	-4.8638962689
C	6.3666804584	-7.1538237064	-4.3605222975
C	8.6127106325	-4.3301280842	-4.7348611969
C	5.2193608341	-7.6899136109	-3.5951339611
C	3.0347796190	-7.0890307698	1.4447327378
C	1.9058754860	-6.6773092996	2.2063102436
C	4.2627626341	-7.2495433708	2.1286514272
C	4.3451992391	-6.9952991629	3.5091994105
C	3.2233024266	-6.5500024857	4.2139673123
C	1.9863849678	-6.4025769537	3.5711610228
C	-1.2554545772	-6.6957786039	-1.4615242576
C	-1.2757412976	-5.2863913910	-1.3107812696
C	-2.4678595164	-7.3839939373	-1.6543750434
C	-2.4760728016	-4.5780227545	-1.3511107445
C	-3.6843519168	-6.6779940889	-1.6735140765
C	-3.6879316741	-5.2782427549	-1.5285775869
H	6.0137745612	-3.4370009286	-3.9921414694
H	6.1709515481	-2.1145224478	-1.9229505773
H	4.2028086127	-5.1981663495	-4.2373141869
H	4.5528532323	-2.5041223232	-0.0398679008
H	8.7844506296	-2.7330876909	-3.4464047974
H	4.2988491442	-7.7224171311	-1.7608813081
H	7.5812316273	-5.6242871465	-7.0321128173
H	5.6264344561	-7.4738866520	-6.3959071430
H	5.1411689020	-7.5355698956	1.5473764575
H	5.2846980729	-7.1333185289	4.0505416855
H	3.2921211825	-6.3641409712	5.2968160511
H	1.0947204470	-6.0839563288	4.1113491422
H	-2.4562684600	-8.4690890883	-1.7605610375
H	-4.6451768370	-7.1964624251	-1.7891379005
H	-2.4650982521	-3.4939090805	-1.2421687885
H	-4.6477628856	-4.7474519793	-1.5406862563
O	2.4546329275	-5.7929544386	-2.3407013773
O	2.5915020712	-4.3734686593	-0.1040617090
O	7.4352812466	-6.3778252113	-3.8120023622
O	8.7524079448	-3.6411982028	-5.7623490698
O	4.1710303053	-7.9415126386	-4.2294228989
O	8.7017910922	-4.4202429398	-2.2682042564
O	6.3055473430	-7.6107215542	-1.4088252315
O	2.8858490614	-7.2798219054	0.1239522381
O	0.7060531271	-6.5373277656	1.4943662143
O	-0.0274014418	-7.3039042621	-1.3556928109
O	-0.0318087846	-4.7313538390	-1.0321475739
Fe	1.4600889272	-6.1798667386	-0.7222963907
N	8.7461755257	-3.7524475910	-3.4973510611
N	5.2003816831	-7.8654795360	-2.2373938955
H	0.2247805814	-3.7903729117	-1.4860533028
H	2.9598732000	-4.1169268453	0.8454772949
H	0.4278412061	-7.5047282622	1.2743342914
H	7.7317423344	-4.4707005932	-2.0300189771
H	6.3592228373	-8.4482036738	-0.8770771008
O	3.5832136485	-3.7473919390	2.1457449790
H	3.2676136369	-4.3931677655	2.8102076100
H	4.5754200604	-3.9139518346	2.0716970507
O	6.2396841220	-4.2410350231	1.9620105829
H	6.3213632565	-4.9328987413	2.6420671102
H	6.4410892172	-4.7203602765	1.1201610204
O	1.2909742223	-8.4156982903	-3.9410910253
H	2.2611146431	-8.2678160785	-3.8720102113
H	0.9192303695	-8.1424106109	-3.0790411689
O	0.3661874800	-9.0175857652	0.6948731917
H	1.3155538217	-9.2059987605	0.5713812619
H	0.0894515116	-8.6665809026	-0.1958233693
O	6.3635102450	-0.9502081463	-4.7115486572
H	7.3347101944	-0.8246312626	-4.6697398098
H	6.2365928302	-1.2195916852	-5.6409817807
O	7.5639113184	-5.5648382672	-0.0372924866
H	8.5336102544	-5.4021712317	0.0174396392
H	7.4642591758	-6.3038156632	-0.6776434370
O	6.2889887066	-9.8439164527	0.1614805719
H	6.2664665514	-9.8049254300	1.1324639867
H	6.2939597166	-10.7921437216	-0.0692495429
O	-1.2787798702	-3.4320850968	-3.8762831473
H	-1.2759707262	-2.9563022349	-4.7251246328
H	-1.2591630166	-4.3759688975	-4.1309910249
O	0.8126149928	-2.5935729203	-2.0145139003
H	1.6875742271	-2.4972198381	-1.5950063860
H	1.0122421413	-2.7225495861	-2.9983051437
O	-2.4266382420	-5.9548046012	1.7890112309
H	-1.4487158145	-5.9788855615	1.9082696287
H	-2.5065547900	-5.6365684859	0.8695513213
O	0.7826569570	-5.6291748613	-4.5068276306
H	1.3288923094	-5.6599595470	-3.6799666121
H	0.8329498662	-6.5733763160	-4.7774143289
O	1.6279942664	-3.0106653000	-4.5677496449

H	2.5975796286	-3.0969260872	-4.5132654621
H	1.3124921697	-3.9348490850	-4.7555928840

OPT-FDCH-0H

C	4.9708069558	-3.5612611967	-4.3433115153
C	5.4583454390	-2.8260707004	-3.2538145069
C	4.8953357215	-3.0111398587	-1.9723860344
C	3.9673824893	-4.5248811709	-4.1613112406
C	3.8876675531	-3.9727421797	-1.7723927259
C	3.4556878843	-4.7762258817	-2.8709571223
C	6.6200823901	-7.8451222183	-5.1119624600
C	7.7227030003	-7.0248213317	-5.5518947886
C	7.7522913465	-5.9106863522	-4.7473963399
C	6.1251410502	-7.2213460183	-3.9774660011
C	8.7614334160	-4.8156267826	-4.5189774742
C	5.6915250566	-7.6312454482	-2.6132633620
C	2.3421250625	-6.4547781539	2.0429350861
C	1.2972157381	-5.4874207222	2.1530973094
C	2.9673720743	-6.9140554283	3.2260197267
C	2.6234135910	-6.3878768331	4.4983608205
C	1.6432722300	-5.3871619084	4.5746064855
C	0.9898659952	-4.9443865895	3.4113419926
C	-1.0227947203	-6.1238211991	-1.7665918156
C	-1.0296707170	-4.7092661542	-1.5622353065
C	-2.2431648313	-6.7963774301	-1.9904650052
C	-2.2533343623	-4.0316188198	-1.5145478422
C	-3.4730621635	-6.0973174790	-1.9486396707
C	-3.4753359032	-4.7154112785	-1.6978554606
H	5.4010351119	-3.4161046931	-5.3376877319
H	6.2897367260	-2.1278173778	-3.3940873627
H	3.6001628354	-5.1046501803	-5.0123810045
H	5.2197799425	-2.3980654536	-1.1309737805
H	9.9554180375	-4.1781507865	-3.0642461961
H	7.5187695867	-6.6315833384	-2.0469197717
H	8.4106012103	-7.2313034204	-6.3733493799
H	6.4147765428	-8.8606202189	-5.4345975918
H	3.7372429206	-7.6840392101	3.1315455285
H	3.0996347693	-6.7421502976	5.4204127512
H	1.3856297262	-4.9564342398	5.5444585617
H	0.2385772122	-4.1554059425	3.4612355240
H	-2.2263758010	-7.8736276794	-2.1789623231
H	-4.4276532076	-6.6149761998	-2.0982234838
H	-2.2463710829	-2.9636686857	-1.2949014254
H	-4.4347077395	-4.1882456356	-1.6412467297
O	2.5251450108	-5.6972996196	-2.5879787506
O	3.2643062028	-4.2138510877	-0.5889246509
O	6.6783129578	-5.9621134708	-3.8750713745
O	9.0047950234	-3.9839895272	-5.4058939977
O	4.7805057386	-8.4443441747	-2.2608954172
O	8.9663770779	-5.7208143864	-2.2168218856
O	6.4608288358	-7.2267524255	-0.3729422298
O	2.6685288305	-6.8568866298	0.7886504169
O	0.6541906973	-5.1342565072	1.0232669956
O	0.1731521913	-6.7429128084	-1.5954638615
O	0.1873708262	-4.1229772121	-1.4116930157
Fe	1.5900194133	-5.5165437127	-0.7689194405
N	9.0276977864	-4.6469277672	-3.1529008759
N	6.5551880323	-7.0337591032	-1.7356535265
Na	0.4556592113	-3.1686370267	-3.4995341041
Na	-1.0285137992	-7.1224657639	0.5367754303
Na	7.7735132087	-4.3211489393	-1.0282261723
Na	5.2272082886	-9.1747274817	-0.0420316737
O	4.6591589566	-4.3436732335	2.5718197526
H	4.0634449905	-3.6059471680	2.2676465542
H	4.1323010631	-4.8491899508	3.2251351554
O	3.1566118575	-2.4136607836	1.2619080197
H	3.6454884340	-1.6222923516	0.9839048920
H	3.2134860559	-3.0628657963	0.4650338798
O	1.9054201495	-8.9229922443	-2.0913082599
H	2.8196120694	-8.6219718281	-2.2986184113
H	1.3422127981	-8.1277023123	-2.2175224825
O	0.7729276698	-8.8331005022	0.5745736524
H	1.5153611131	-8.1911201680	0.7378392513
H	1.0124946526	-9.1079933300	-0.3427506430
O	6.5389830153	-3.5170125632	0.7951075840
H	6.4164587601	-3.9691711932	1.6610791257
H	6.4343630077	-2.5760259310	1.0135271569
O	7.8028089217	-6.3788131673	1.5837789183
H	8.7772894458	-6.4248441421	1.7335589424
H	7.6587379504	-6.7286999443	0.6395479443
O	6.4590090360	-8.8804794912	2.0151759751
H	6.8486366447	-7.9695010355	1.9955625167
H	6.4954456354	-9.1517783673	2.9463237753
O	-1.0312707216	-1.3853478161	-3.0020899372
H	-0.9967737652	-1.2154726054	-2.0393530558
H	-1.0194199274	-0.4991284375	-3.4037010545
O	0.7955794185	-1.7403860408	-0.4225773503
H	1.3759398317	-1.8533486626	0.3600575382
H	0.5034391008	-2.6782409795	-0.6376922055
O	-2.1921022290	-5.2181511823	1.5715519022
H	-1.2522018800	-4.8930208049	1.4031377462
H	-2.2483579889	-5.1810515450	2.5430521125
O	0.7447381079	-5.2907150729	-4.4566126726
H	1.4035260852	-5.6300176884	-3.7707890656
H	0.0276145563	-5.9441001917	-4.4437523349
O	2.0683917751	-1.5235344893	-2.8698676888
H	1.7569784937	-1.5012565417	-1.9229500307
H	2.9892215589	-1.8465646354	-2.8235034756
Na	4.7324986522	-5.8019795591	0.5345837678

OPT-AHA-four-H

C	1.0526000724	-2.1283077130	-2.5667514411
C	2.4415638719	-2.3292768501	-2.5625857097
C	3.1262343766	-2.5906870771	-1.3667944746
C	0.3181719878	-2.2014597904	-1.3727065261
C	2.4047513235	-2.6726156662	-0.1621772084
C	0.9936068985	-2.4987332608	-0.1819223538
C	5.2608340079	-6.4535156697	1.6304076370
C	5.6953555790	-7.3925292219	2.7277389490
C	1.5334935833	-2.9568148726	5.0244949740
C	2.4942272383	-4.0025921491	5.0428445546
C	2.9505708923	-4.5360427689	6.2539322594
C	2.4504583333	-4.0344360473	7.4645758296
C	1.5033996588	-2.9990457293	7.4608636068
C	1.0450027801	-2.4578687014	6.2488364192
C	-0.0441212303	-6.2419293895	2.4719521725
C	0.8007417514	-6.3451753302	1.3386291577
C	-0.9611760437	-7.2419442319	2.7985860292
C	0.6464562194	-7.4589572017	0.4945657368
C	-1.0745182378	-8.3652426619	1.9594788444
C	-0.2854622500	-8.4603402289	0.8064645667
H	0.5567886276	-1.9408026653	-3.5288775284
H	2.9703124234	-2.2893962572	-3.5257465664
H	-0.7584456857	-2.0217587450	-1.3617791225
H	4.2048658125	-2.7531562325	-1.3559267838
H	5.3631919860	-6.9865974526	3.6960258792
H	6.7927608861	-7.4359174207	2.7374323073
H	5.2820317040	-8.4021456174	2.6143130682
H	3.6802322427	-5.3451536918	6.2371489344
H	2.7822256852	-4.4304243507	8.4353396825
H	0.3168694263	-1.6474260690	6.2431703695
H	1.1411837083	-2.6284261248	8.4327340538
H	-1.5830153001	-7.1344898624	3.6882162764
H	-1.7909659143	-9.1486202737	2.2013688586
H	-0.4013805487	-9.3126045799	0.1371512243
H	1.2622248316	-7.5192721631	-0.4030466346
O	0.3941196268	-2.6591554892	1.0361726249
O	2.9749466270	-2.9148803585	1.0401207923
O	3.8048453477	-6.1581793028	-0.2229499521
O	5.7324881319	-5.2765057606	1.5497961141
O	1.1250290486	-2.5180515247	3.8125137125
O	1.6908970297	-5.3658899422	1.1284489321

O	2.9244571841	-4.4593363676	3.8194305959
O	0.1292905416	-5.0782446702	3.1750027173
Fe	1.7291466885	-3.7377141355	2.3075186441
N	4.3602477426	-6.9498513130	0.7774029477
H	2.9797113240	-5.7523449942	0.2209719439
H	3.8465539541	-7.8297324530	1.0243932074
H	-0.7203815596	-2.7536855157	1.0484733057
H	-0.4638079784	-4.9671599421	4.0135333864
H	4.3969147556	-4.2977157842	3.8207152787
O	-2.4323423112	-5.3379459252	0.5268845689
H	-2.1681815659	-6.0359376558	1.1555789906
H	-1.7583794169	-5.3981377622	-0.2189752164
O	-2.0244077816	-2.8884968729	1.2525149246
H	-2.0686606366	-2.7149710029	2.2262208087
H	-2.2530761108	-3.8767599176	1.0707642751
O	-1.2922943064	-4.5382583598	5.2398763380
H	-1.5445927854	-3.6333594984	4.8983947696
H	-0.6380663591	-4.3369272426	5.9401228417
O	5.1599981923	-1.4024656207	1.3626830983
H	5.7784147387	-1.9623332783	0.8137303792
H	4.2950269380	-1.9002802352	1.2666927767
O	2.9257504169	-9.0061554303	2.0169530834
H	2.0328062457	-9.0754994101	1.6286888643
H	2.7915253207	-8.3848783596	2.7865134881
O	5.7129293240	-1.7065909785	3.7926141622
H	5.4892025153	-1.4993911869	2.7685278446
H	5.0209760262	-1.2793110388	4.3265805700
O	2.6714161266	-7.2125266152	4.0911863496
H	2.7445378576	-6.2390755080	3.8942112038
H	1.7863676394	-7.3059332622	4.4815927836
O	-0.7132963169	-5.1643133886	-1.4887182720
H	0.1552889284	-5.6009157903	-1.6484032453
H	-0.4807706642	-4.2164956897	-1.5520326660
O	6.4667814095	-3.2640252470	-0.1369102763
H	7.4389174244	-3.3643044296	-0.1309687044
H	6.1166130846	-4.0741241072	0.3217315239
O	5.4372283936	-4.1557498183	3.8255028295
H	5.7235193920	-4.5531309956	2.9168796457
H	5.5856537830	-3.0511340442	3.8406075098
O	-1.4487110414	-2.1975725086	3.8873031359
H	-0.4192269235	-2.1849627838	3.8188082424
H	-1.7210596021	-1.3073605893	4.1644830643
O	1.8607896278	-5.5974151135	-2.5235160717
H	2.7064612092	-6.0693232565	-2.4188928694
H	2.1139505075	-4.6503784300	-2.5103755643

OPT-AHA-zero-H

C	2.3156964256	-0.6500132272	-1.6602398761
C	3.5925340215	-1.0072287999	-1.2097854636
C	3.7694946772	-2.1242162755	-0.3835511212
C	1.1979504819	-1.4089012223	-1.2760565563
C	2.6671981436	-2.9049145823	-0.0062307000
C	1.3610106970	-2.5282475461	-0.4481644281
C	5.1665766760	-7.8867059286	-0.3094722243
C	6.2740611388	-8.8071597588	0.2039742158
C	1.9488924283	-2.7872162966	3.5912886361
C	2.4351948769	-4.0639781923	3.9949290667
C	3.4501112211	-4.1421779717	4.9578975333
C	3.9875376278	-2.9709506707	5.5141763136
C	3.5159117016	-1.7176654653	5.1106838412
C	2.4966559903	-1.6268361837	4.1501068809
C	-0.6968551516	-6.6662018963	1.9367124156
C	0.3684603984	-7.2418722659	1.1663382067
C	-1.6180123870	-7.5145863613	2.5642422184
C	0.4415026004	-8.6491191676	0.9836621709
C	-1.5197544042	-8.9026851868	2.3979593468
C	-0.5146517293	-9.4668802984	1.6017055444
H	2.1869160311	0.2221162144	-2.3016907832
H	4.4600185493	-0.4209057240	-1.5059542878
H	0.1988407699	-1.1482350826	-1.6310506975
H	4.7492348381	-2.4003817516	0.0025772313
H	6.2145532679	-9.8472060612	-0.1575296538
H	6.2478213789	-8.8367725181	1.3055791768
H	7.2772262935	-8.4529129619	-0.0872852642
H	3.8149089015	-5.1242915343	5.2631248109
H	4.7744419128	-3.0439895968	6.2625600001
H	2.1270931436	-0.6587987326	3.8112865626
H	3.9371289872	-0.8087460521	5.5385337541
H	-2.4004702429	-7.0823358944	3.1862610553
H	-2.2362289728	-9.5551579313	2.8952431851
O	0.3289455417	-3.3128151774	-0.0306193174
O	2.7713532415	-4.0265694659	0.7333444044
O	4.5833155427	-6.5378070954	-2.2133992567
O	4.1817900815	-7.5195111837	0.4189665221
O	0.9318081266	-2.7674608890	2.6824756006
O	1.2819482188	-6.3701881543	0.7124362452
O	1.8562920644	-5.1488344381	3.4086282736
O	-0.7027015965	-5.3339889734	2.0920165071
Fe	0.9854551922	-4.4831572147	1.4853742572
N	5.3348752339	-7.4462299120	-1.5649432082
Na	3.0848593208	-5.8028541809	-0.7821823029
H	6.2148690964	-7.7128053341	-2.0308230602
Na	-1.3592157102	-1.8524236663	1.0300317907
Na	-0.5379293687	-4.4827070151	4.2382920816
Na	3.2563487594	-6.5456381686	2.2623972475
O	-3.7292365739	-4.6453074463	3.0713968621
H	-3.7404711508	-5.2918342167	3.7974221852
H	-3.7239556093	-5.1518108141	2.2148284234
O	-3.7228314395	-2.1849995777	1.8519764509
H	-3.7400411270	-1.5131334435	2.5592863007
H	-3.7336830195	-3.0317143399	2.3773863822
O	-1.1029323076	-5.7996014622	6.0462942861
H	-1.0927634676	-5.7731319143	7.0153869856
H	-0.3947674222	-6.4583760155	5.7695648389
O	6.2615206170	-5.9666916999	2.6142030979
H	6.2706630662	-5.8793429655	1.6214010291
H	6.2828634358	-6.9231938431	2.7902118602
O	2.7097182310	-8.8382429590	3.1554036677
H	2.1757044872	-8.8049777093	3.9734874726
H	2.0234011445	-8.9075825824	2.4543201890
O	-0.0433536114	1.1441119949	1.1187182633
H	0.5735713965	1.1351346322	0.3604468725
H	0.5549194420	1.1545124515	1.8885008254
O	0.7373691440	-7.1975808127	4.7430034430
H	1.2545414556	-6.4572664270	4.2876036198
H	0.1408569426	-7.5306103306	4.0346260661
O	-3.7333336704	-5.3196226427	0.4593286709
H	-3.7241305577	-6.0849994538	-0.1424965457
H	-3.7085592642	-4.5234052444	-0.1116768173
O	6.2724761599	-4.9852890458	0.1124680933
H	6.2417660530	-5.0119731407	-0.8620436608
H	6.2718689593	-4.0491054572	0.4098206543
O	6.2724577233	-3.1584777833	2.1740715081
H	6.2561820454	-2.4167233383	2.8034853998
H	6.2649493710	-3.9710090635	2.7284163502
O	-1.1951171958	-1.9951126900	3.8040685593
H	-0.2684123940	-2.1617344312	3.3830863195
H	-1.0613602165	-1.2855002248	4.4528494857
O	-2.0200042202	-3.0081274487	-1.0921217191
H	-1.0738745532	-3.2998581897	-0.9030627838
H	-2.0469261466	-2.7972933014	-2.0382675389
H	1.2553300879	-8.9921769274	0.3430853434
H	-0.4658554747	-10.5515972837	1.4796722234

OPT-MAHA-4H

C	3.4849182595	-1.4343358447	-2.1297563359
C	4.6388199813	-2.2259234238	-2.0545617318
C	4.8303167925	-3.0902963343	-0.9658416966
C	2.4824346861	-1.5219217107	-1.1575943617
C	3.8399784919	-3.1622443628	0.0132801317
C	2.6351656247	-2.4170264158	-0.0892405740
C	5.1920572931	-6.7696609296	1.9226550571

C	5.7725296253	-7.5486073762	3.0690005804
C	3.6061877758	-8.6429477775	1.3872038499
C	2.7118718354	-3.1180070654	4.7396743640
C	3.1173177778	-4.4723876471	4.6855847999
C	3.7418348600	-5.0439948296	5.8076044401
C	3.9420843553	-4.2639770325	6.9542014536
C	3.5409250784	-2.9214677176	6.9818503927
C	2.9236299863	-2.3361962682	5.8692128771
C	-0.4535545125	-5.6915822451	1.8393268441
C	0.4640139694	-6.2667603968	0.9320709076
C	-1.7398466017	-6.1998901445	1.9935397123
C	0.0544593636	-7.3685132080	0.1621421987
C	-2.1477104625	-7.2934738306	1.2123844669
C	-1.2496128106	-7.8698092136	0.3023152313
H	3.3514735813	-0.7624131565	-2.9776665123
H	5.3702630089	-2.1817414590	-2.8654816935
H	1.5667398074	-0.9353250604	-1.2244553255
H	5.7218148399	-3.7098565314	-0.8736491096
H	3.9042767049	-9.0557032374	2.3571582149
H	3.8734718716	-9.3176918770	0.5617622049
H	2.5157945742	-8.4962255278	1.3984434944
H	5.0009590801	-7.7867024183	3.8196766926
H	6.5546677669	-6.9366304540	3.5371123670
H	6.2028727591	-8.4936854418	2.7081380497
H	4.0317259600	-6.0946401989	5.7631009738
H	4.3801341428	-4.6963192309	7.8618621546
H	2.6003835323	-1.2965148657	5.8800165991
H	3.6897141361	-2.3400562163	7.8945964414
H	-2.4093140485	-5.7146654597	2.6998551794
H	-3.1796293575	-7.6569000943	1.2957852865
H	-1.5640213805	-8.7110547547	-0.3150561311
H	0.7514190339	-7.7971806356	-0.5583016883
O	1.6834310604	-2.6396430830	0.8358301555
O	3.9099426093	-3.9343599433	1.1457098794
O	3.8472250678	-6.7068644594	-0.0012353912
O	5.6177863332	-5.5909460265	1.6716069631
O	2.1140133785	-2.6642876539	3.5840077304
O	1.6784418889	-5.6930147342	0.8474746095
O	2.8322712346	-5.1677642172	3.5633951709
O	0.0103231266	-4.5783299173	2.5178119249
Fe	2.0609628240	-4.1610289083	2.0549056444
N	4.2414096396	-7.3491829387	1.1731810559
H	2.9200620252	-6.3208793991	0.2267485673
H	1.4045226438	-1.9052106619	3.7125577994
H	-0.3271171566	-4.6056257551	3.5309081783
H	4.7076629233	-4.6906978818	1.2245219687
O	0.2051986183	-1.0172778103	3.7472139637
H	0.4305891790	-0.0816273679	3.8817730926
H	-0.1812550382	-1.1144538778	2.6796552368
O	-0.8320511163	-2.3509085602	5.8296776334
H	-0.5437122254	-1.7818609651	5.0572695477
H	-1.7661546782	-2.1337484917	5.9811326204
O	3.2394218559	-8.7764144020	4.8691620450
H	3.1330259731	-9.2687079429	5.6975423153
H	2.4080500843	-8.2428670232	4.7801276387
O	1.1759273970	-4.5742675089	-2.0483905790
H	1.7546535273	-4.0536822351	-1.4557342871
H	1.3840412033	-4.2458654218	-2.9412585558
O	4.6065729260	-8.6846126987	-2.0158402658
H	4.1974465772	-7.9202594507	-1.5618315865
H	5.4547512334	-8.7665975129	-1.5369708724
O	-0.7055457252	-4.8217118323	4.8907715339
H	-0.0219277120	-5.4436975330	5.2223433425
H	-0.6587809828	-3.9413477880	5.3804393013
O	-2.2703213855	-3.0636489605	0.7952922128
H	-1.9368698475	-3.6450665537	0.0389734088
H	-3.2088879823	-2.8880649695	0.6005222269
O	-0.4562201905	-1.2565507213	1.4013497648
H	0.3685780862	-1.7347894415	1.0579286896
H	-1.2159577727	-1.8964028685	1.2028376045
O	1.1317549487	-7.0308835881	4.4154920114
H	0.5487293438	-7.2844141704	3.6755436130
H	1.7858678659	-6.3807274657	4.0104387498
O	-1.4423346734	-4.6430689663	-1.1790626545
H	-0.5047516219	-4.5521095802	-1.4997992158
H	-1.4851281005	-5.5573670759	-0.8404875089
O	6.6470308463	-4.3250318906	4.1331583180
H	6.3361117956	-4.6385890388	3.2580329652
H	5.8254903400	-4.2157546777	4.6444343150
O	6.7459715566	-9.0905229969	-0.0278932368
H	7.6855842270	-8.8321381217	-0.0868939184
H	6.7644299057	-10.0620611511	-0.0098326888

OPT-MAHA-0H

C	0.9872786243	-1.4777228859	-2.1145423249
C	2.3837742025	-1.4297121906	-2.0878376900
C	3.0839037510	-2.0842974089	-1.0537177701
C	0.2812006074	-2.1510560842	-1.1036449588
C	2.3951692523	-2.8243059589	-0.0725430745
C	0.9624959151	-2.8127373215	-0.0723763681
C	5.5971445151	-6.0754350153	1.7802550177
C	5.9282885375	-6.4523104918	3.2031639202
C	4.8392489812	-8.3947307855	1.2813415583
C	2.6178503703	-3.5274626521	4.7280940602
C	3.1430215163	-4.8544086340	4.6827722267
C	3.7719159035	-5.3865536607	5.8268064742
C	3.8993085692	-4.6069382534	6.9894333143
C	3.4194360208	-3.2915522198	7.0081217020
C	2.7797677763	-2.7497774513	5.8845856326
C	-0.3494231883	-6.5453697414	2.3101410393
C	0.6349257344	-7.0563592562	1.3901125631
C	-1.5569022781	-7.2569774392	2.4936986467
C	0.4849136302	-8.3471665496	0.8618440650
C	-1.6909681542	-8.5353477185	1.9187792270
C	-0.6701966377	-9.0926643222	1.1408397002
H	0.4451610949	-0.9941736920	-2.9281659179
H	2.9237334736	-0.8953491526	-2.8734779644
H	-0.8084816352	-2.1714536380	-1.1019446662
H	4.1730778192	-1.9932279439	-0.9868742921
H	4.9535203441	-8.5831488569	2.3516507515
H	5.5602014725	-9.0061895297	0.7194212159
H	3.8131253459	-8.6586903524	0.9886211603
H	5.1867433208	-7.1177991993	3.6584990303
H	5.9593266830	-5.5310419586	3.7982600732
H	6.9168188556	-6.9350789619	3.2329824474
H	4.1363482567	-6.4138584724	5.7982764115
H	4.3359268363	-5.0233696287	7.9045387650
H	2.3438898855	-1.7499035749	5.9188133942
H	3.4988018993	-2.7066838470	7.9279747299
H	-2.3319445805	-6.8317763362	3.1319774912
H	-2.6077807807	-9.0977413466	2.0941596135
H	-0.7624074337	-10.1117324941	0.7564584359
H	1.2758409018	-8.7555424906	0.2271755412
O	0.3411944507	-3.4168837923	0.9509227665
O	3.0207348724	-3.5124603024	0.8921709028
O	4.7954389197	-6.6906285246	-0.3531850732
O	5.8690905437	-4.8926550680	1.3541516698
O	1.9477863999	-3.1006176324	3.6322994339
O	1.6780334543	-6.2646104351	1.0822480821
O	2.9219949920	-5.5415123959	3.5466604445
O	-0.0368298030	-5.3946508013	2.9297819352
Fe	1.7238802339	-4.5824386695	2.1553524098
N	5.0601481494	-6.9937142062	0.9617053405
Na	2.2617686435	-6.5428084905	-1.2736065901
Na	3.4165135134	-1.6064258372	2.6731002311
Na	0.6175551750	-5.7204700198	5.3906460150
Na	4.6514190566	-4.4841732045	-0.5348807882
O	1.8614647275	-0.2211579441	1.6028396447
H	1.9204813085	-0.2668962007	0.6312881184
H	0.9089825470	-0.4371788408	1.7783608584
O	0.0899151307	-1.4371512476	4.6915453767

H	-0.4585810682	-1.1838923545	3.9173756674
H	0.7990219782	-1.9851596720	4.2509906053
O	1.0887474817	-7.8668176650	6.0259328062
H	1.6985765587	-8.1304803604	5.2653847166
H	1.4823794175	-8.1930388137	6.8529022012
O	3.4862622557	-4.5709275986	-2.9841342568
H	3.5604242183	-4.8015030452	-3.9340245737
H	3.0312268173	-3.7013870237	-2.9695019312
O	3.6268376223	-8.2665630561	-1.9570437204
H	4.2737105563	-7.8321883849	-1.2859277576
H	3.9349693551	-9.1682796920	-2.1217399234
O	-0.7827330823	-3.9736470664	5.1855870139
H	-0.8150001523	-4.2255058617	4.2341887660
H	-0.5785051768	-2.9979247811	5.1670042890
O	-1.6483145336	-5.0513983400	-0.1116955347
H	-1.0247139378	-4.4519395197	0.3850316187
H	-1.6905197306	-5.8406369307	0.4674963213
O	-0.7108690296	-1.2555015369	2.0245676256
H	-0.4389056601	-2.1668827687	1.6708007042
H	-1.5660776029	-1.0498225367	1.6105139689
O	2.5316217509	-8.1355824061	3.8192801629
H	2.7371046808	-7.1576210897	3.6431795086
H	1.9198658360	-8.3805046633	3.0973666591
O	0.3459868691	-5.4021038225	-1.9636215186
H	0.4923052409	-4.4466644659	-2.0901094488
H	-0.4373747249	-5.4065320322	-1.3353272904
O	5.5030425236	-2.6500276314	2.7599531995
H	6.0718865526	-2.7131128722	3.5431046769
H	5.6313804491	-3.5365051586	2.2847634489
O	8.3339092609	-7.5225938300	1.1147401492
H	9.2795359580	-7.7330959960	1.2005705786
H	8.3266654497	-6.6115051010	0.7700616702
